# Plasma biomarkers for residual feed intake prediction in beef bulls

**DOI:** 10.1093/tas/txag020

**Published:** 2026-02-23

**Authors:** Mauro Venturini, Daniella Heredia, Maria Camila López Duarte, Kamryn Joyce, Martin Ruiz-Moreno, Federico Tarnonsky, Wilmer Cuervo, Nadia Ashrafi, Stewart Graham, William Thatcher, Joao Bittar, Jose Dubeux, Ricardo Chebel, Nicolas DiLorenzo, Angela Gonella

**Affiliations:** Department of Animal Sciences, North Florida Research and Education Center, Institute of Food and Agricultural Sciences, University of Florida, Marianna, FL, 32446, United States; Department of Animal Sciences, North Florida Research and Education Center, Institute of Food and Agricultural Sciences, University of Florida, Marianna, FL, 32446, United States; Department of Animal Sciences, North Florida Research and Education Center, Institute of Food and Agricultural Sciences, University of Florida, Marianna, FL, 32446, United States; Department of Animal Sciences, North Florida Research and Education Center, Institute of Food and Agricultural Sciences, University of Florida, Marianna, FL, 32446, United States; Agronomy Department, North Florida Research and Education Center, Institute of Food and Agricultural Sciences, University of Florida, Marianna, FL, 32446, United States; Department of Animal Sciences, North Florida Research and Education Center, Institute of Food and Agricultural Sciences, University of Florida, Marianna, FL, 32446, United States; Department of Animal Sciences, North Florida Research and Education Center, Institute of Food and Agricultural Sciences, University of Florida, Marianna, FL, 32446, United States; Department of Animal & Range Sciences, Montana State University, Bozeman, Montana, 59717, United States; Metabolomics Department, Corewell Health Research Institute, Royal Oak, MI, 48073, United States; Corewell Health East William Beaumont University Hospital, Royal Oak, MI, 48073, United States; Metabolomics Department, Corewell Health Research Institute, Royal Oak, MI, 48073, United States; Corewell Health East William Beaumont University Hospital, Royal Oak, MI, 48073, United States; Oakland University-William Beaumont School of Medicine, Rochester, MI, 48309, United States; Department of Animal Sciences, Institute of Food and Agricultural Sciences, University of Florida, Gainesville, FL, 32611, United States; Corewell Health East William Beaumont University Hospital, Royal Oak, MI, 48073, United States; Agronomy Department, North Florida Research and Education Center, Institute of Food and Agricultural Sciences, University of Florida, Marianna, FL, 32446, United States; Corewell Health East William Beaumont University Hospital, Royal Oak, MI, 48073, United States; Department of Animal Sciences, North Florida Research and Education Center, Institute of Food and Agricultural Sciences, University of Florida, Marianna, FL, 32446, United States; Department of Animal Sciences, North Florida Research and Education Center, Institute of Food and Agricultural Sciences, University of Florida, Marianna, FL, 32446, United States

**Keywords:** feed efficiency, metabolome, pathway, choline, ceramides

## Abstract

Residual feed intake (RFI) is a measure of feed efficiency (FE) independent of growth and body weight (BW), calculated as the difference between actual and expected feed intake (FI) based on mean metabolic weight (MW) and weight gain. We hypothesized that bulls with contrasting RFI differ in plasma concentration of different compounds. RFI was evaluated in 302 bulls from 3 different ranches. After adaptation, bulls consumed the same diet for 56 d, individual FI was daily recorded, BW was measured every 2 weeks, and blood samples were taken at d 0 and 56. The bulls were ranked as low RFI (LRFI) and high RFI (HRFI), and the top and bottom 30 were used for metabolomic, hormone, and isotope analysis. Multivariate and pathway analysis were conducted with MetaboAnalyst, and univariate analysis was conducted with SAS using Mixed procedures. Additionally, for Biomarker analysis, Receiver Operating Characteristic (ROC) curves were constructed with MetaboAnalyst. Sparse Partial Least Squares–Discriminant Analysis (sPLSDA) showed partial cluster separation evidenced by acceptable R^2^ values. The model showed poor predictive power, reflected by low *Q*^2^ values. For LRFI animals (more feed efficient), the most abundant metabolites (*P* = 0.05) at d 0 were Cer(d18:1/24:1), TG(20:2_32:1), SM (OH) C22:1, SM (OH) C22:2, SM C24:0, TG(20:2_34:3), GCA, Choline, TG(20:2_34:4), and Hex2Cer(d18:1/14:0), while at d 56 were Cer(d18:1/18:0), Cer(d18:1/23:0), TG(14:0_34:2), C16:2, HexCer(d18:1/20:0), C16:1, lysoPCaC16:0, TG(18:3_38:5), C9, SM C24:1, SM C16:0, Carnosine, Cer(d18:2/12:0), SM C18:0, and Cer(d18:1/24:0). Primary bile acid biosynthesis pathway was enriched at d 0 (*P* = 0.008) and sphingomyelin metabolism at d 56 in LRFI (*P* = 0.041). Compounds that were identified as variable importance of projection (VIP) and were also statistically different in the univariate analysis, were used to construct ROC curves to identify their potential as biomarker for RFI. With an Area Under the Curve (AUC) value > 0.7 and p-value < 0.05 as the criteria for diagnostic potential, choline was identified as biomarker of RFI at d 0, and Cer(d18:1/23:0) and TG(18:1_30:0) were identified as biomarkers of RFI at d 56. Although multivariate model showed a poor predictive value, further exploration of individual metabolites could provide insights into the mechanisms contributing to FE differences.

## Teaser

Blood compounds such as choline and certain fatty acids may help explain why some cattle are more feed efficient than others, offering potential biomarkers to guide future selection and breeding programs.

## Introduction

Given the increasing food demand and the scarcity of resources, improving feed conversion into animal products is a priority. Therefore, enhancing feed efficiency (FE) is essential to minimize input costs and improve profitability and sustainability (Nunes et al. 2024), as well as to reduce the environmental impact of animal agriculture (Lam et al. 2018). The most frequent definition of FE is the Feed Conversion Ratio (FCR), which explains the quantity of output produced per unit of dry matter intake (DMI; Connor 2015). However, studies suggested that feed intake (FI) may be adjusted for body weight (BW) and weight gain (WG) to better reflect the animal’s requirements, by subdividing the actual consumption into 2 factors: the expected FI for maintenance and a given production level, and the residual portion (Bezerra et al. 2013). Thus, residual feed intake (RFI) was proposed as a measure of FE, defined as the difference between an animal’s actual FI and its expected feed requirements for maintenance and growth, independent of BW and average daily gain (ADG). Residual feed intake is calculated as the difference between an animal’s actual feed intake and its expected feed intake, with negative values indicating that the animal consumed less feed than predicted (Koch et al. 1963). Significant barriers to the adoption of RFI include the high cost and the long duration required to accurately measure the trait, as specialized equipment is needed to record feed intake and to process the resulting data (Wang et al. 2006; Moore et al. 2009; Cantalapiedra-Hijar et al. 2024).

Therefore, omics technologies—providing large-scale molecular profiling—are being explored to discover RFI-predictive biomarkers (Foroutan et al. 2020; Kasper et al. 2020; Chakraborty et al. 2022). These technologies provide insights into biological pathways or processes at molecular and cellular levels that differ between groups (Goldansaz et al. 2017). Karisa et al. (2014) evaluated the plasma concentration of different metabolites to differentiate steers with divergent RFI. They proposed that metabolic networks and biological pathways associated with significant metabolites such as energy and protein metabolism and metabolism of urea and methane could be potential predictors of RFI with high accuracy. Similarly, isotopic abundance of ^1^³C and ^15^N in tissues is associated with FE (Carmona et al. 2020; Silva et al. 2022) and RFI (Cantalapiedra-Hijar et al. 2022). Metabolic hormones have also been proposed as RFI markers (Richardson et al. 2004; Davis et al. 2012; Bezerra et al. 2013; Foote et al. 2016; Fitzsimons et al. 2017). In addition, reproductive hormones, such as testosterone, have been considered good candidates to assess age at puberty (Kowalski et al. 2017) and the potential relationship with RFI.

Since the metabolome is considered a link between the genome and phenome (Su et al. 2022), our experiment aimed to investigate the plasma concentration of different metabolites, hormones, and ^1^³C and ^15^N isotopes abundance in beef bulls with divergent RFI. Given that RFI-related biomarkers can change over time (Karisa et al. 2014), that normal metabolic changes during puberty influence anabolic processes and feed efficiency (Kowalski et al. 2017), and that the physiological mechanisms underlying RFI—and therefore its biomarkers—are diet-dependent (Jorge-Smeding et al. 2021; Guarnido-Lopez et al. 2022), we collected plasma samples at both the beginning and the end of the performance test. Our hypothesis was that bulls with contrasting RFI will exhibit differences in plasma metabolites and hormone concentrations, as well as in the abundance of ^1^³C and ^15^N.

## Methods

All animal procedures were approved by the Institutional Animal Care and Use Committee (IACUC) from the University of Florida (UF) for the protocol number 202111541.

### Animals and feed efficiency test

**Figure 1:**
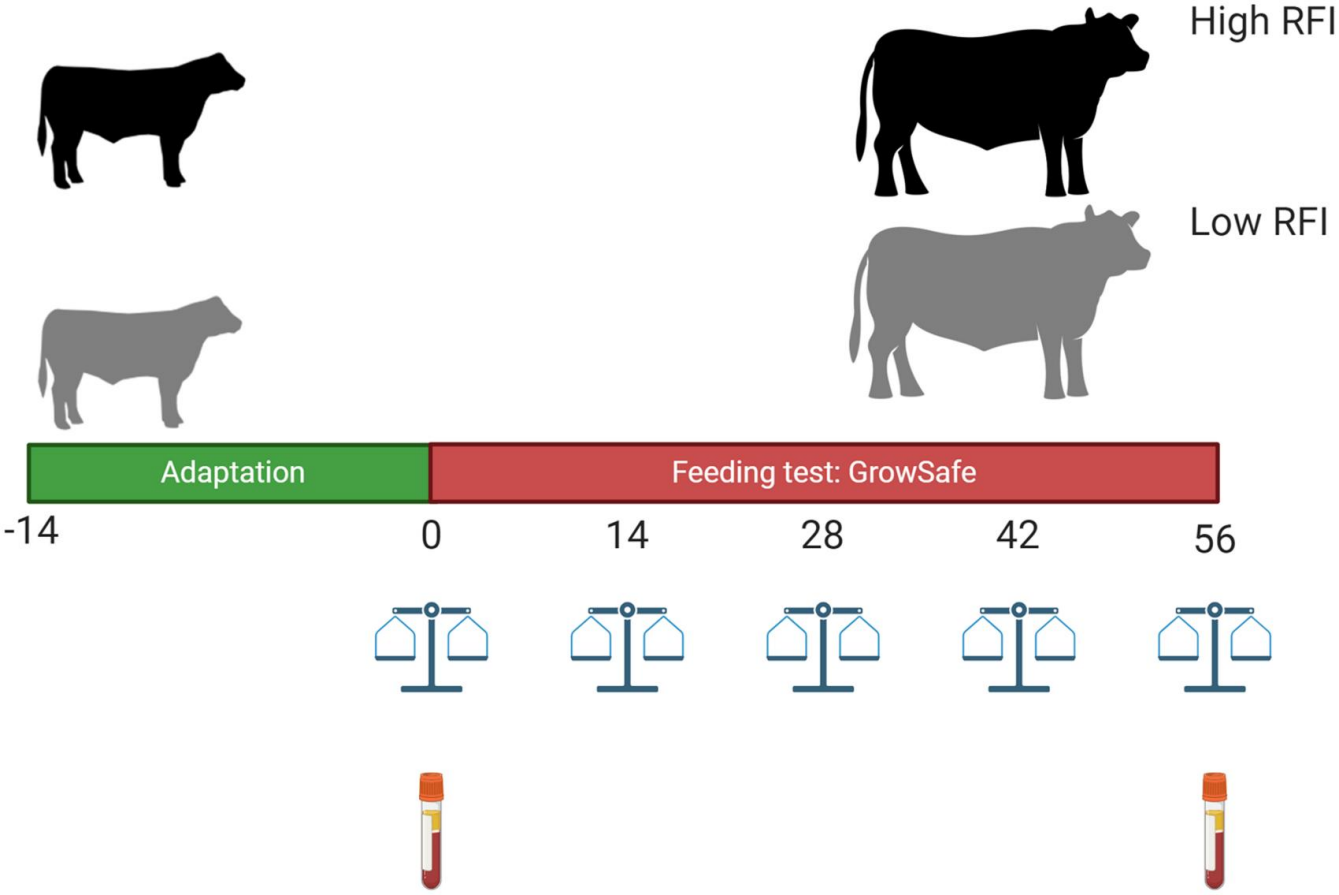
Experimental design for 56 d-feed efficiency test after 14 d-adaptation. Feed efficiency results were utilized to classify the bulls as efficient (Low RFI) or inefficient (High RFI). Blood samples collection for metabolomic, hormone (cortisol and testosterone) and isotopes analysis were conducted on d 0 and d 56.

We used 302 young bulls (238–365 days old) from three ranches: Ranch A (*n* = 103; 43 Angus, 31 SimAngus, 13 Simmental, 11 Charolais, 3 Brahman, 2 Brangus), Ranch B (*n* = 60; Brangus), and Ranch C (*n* = 139; 56 Deseret Red, 50 SimAngus, 33 Brangus). All animals were consigned to the feed efficiency facility at the North Florida Research and Education Center (NFREC) in Marianna, Florida. Ranch A bulls were consigned from August 11 to October 20, 2021; Ranch B from September 8 to November 17, 2021; and Ranch C from October 29, 2021, to January 7, 2022. After arrival, the animals spent 14 d of adaptation (d -14 to d - 0) and then were weighed and randomly allocated into pens equipped with 2 Vytelle Sence® automatic feeder bunks (Vytelle LLC., Lenexa, KS) and wood chips were provided as bedding (Fig. 1). All animals had ad libitum access to water and the same diet for 56 d as a total mixed ration (TMR) to achieve a target rate of gain of 1.89 kg per day. Table 1 contains the ingredients of the diet and chemical composition expressed as dry matter (DM) basis. Individual daily FI was recorded, BW was registered every 2 weeks, and blood samples were taken between 0800 and 1200 h at d 0 and 56 by jugular venipuncture using a 10 mL collection tube containing EDTA-K2E (BD Vacutainer®; Becton Dickinson, Franklin Lakes, NJ).

**Table 1:** Analyzed chemical composition of the ingredients (DM bases) and fed diet to bulls.

	Set up 1 (% of DM)	Set up 2 (% of DM)	Final diet (% of DM)
**Ingredient composition**			
**Corn gluten feed**	35	39	43
**Soybean hulls**	36	39	42
**Cottonseed hulls**	19	12	5
**Bermudagrass hay**	5	5	5
**Suplement¹**	5	5	5
**Chemical composition**			
**DM, %**	90.3	90.4	90.4
**Neg, Mcal/kg of diet DM²**	1.014	1.08	1.17
**Calculated Neg, Mcal/kg of diet DM**	–	–	1.19
**CP, %**	13.4	14.1	14.8
**eNDF^4^, %**	25.0	19.5	14.0
**NDF, %**	N/A	N/A	N/A
**ADF, %**	N/A	N/A	N/A
**Ether extract, %**	2.71	2.81	2.9
**Ca, %**	0.76	0.77	0.77
**P, %**	0.46	0.50	0.54
**S, %**	0.23	0.25	0.27

1 Pellet supplement custom-formulated to provide vitamins (A, D, E) and minerals, and to supply 35 mg of monensin and 10 mg of thiamine per kg of diet DM (Furst-McNess Company, Freeport, IL).

2 Based on diet composition and nutrient content from Nutrient Requirements of Beef Cattle (NASEM, 2016).

3 Calculated based on animal performance according to (Zinn et al. 2007).

4 Effective NDF: Proportion of NDF that is effective in stimulating rumination and is defined as the percent remaining on a 1.18 mm screen after dry sieving.

### Blood samples processing

Blood samples were kept motionless on ice until plasma was separated by centrifugation for 18 min at 3000 × g at 4°C (Allegra X-22R Centrifuge® by Beckman Coulter) and stored in polypropylene vials (1.5 mL) at −80°C (TSX Series® by Thermo Scientific) for metabolomic, hormones, and isotopic analyses. The remaining red blood cell (RBC) pellet was frozen at −20°C for isotopic analysis. Because plasma and RBCs represent matrices with different metabolic turnover rates, variation in isotopic abundance between these fractions can reveal differences in metabolic dynamics; therefore, both plasma and RBC samples were submitted for isotopic analysis.

### Feed efficiency measurement

RFI was calculated as a regression of actual DMI on ADG and mid-metabolic BW (MBW; mid-test BW^**0.75**^), using PROC REG (SAS 9.4 Inst. Inc., Cary, NC), according to the following model: *Y* = *β*_0_ + *β*_1_ X_1_ + *β*_2_ X_2_ + *ε*, where Y is expected DMI, *β*_0_ is the equation intercept, *β*_1_ and *β*_2_ are the coefficients of the equation, X_1_ is the MBW, X_2_ is the ADG, and *ε* is the residual. After the test, bulls that showed any abnormality, disease, or irregularities in behavior patterns were removed from the experimental group. Finally, within each ranch, bulls were ranked according to their RFI values and classified as low (LRFI), medium (MRFI), or high (HRFI). For the metabolomics analyses, we selected the most divergent animals from each location. Specifically, from Ranch A, the 10 lowest- and 10 highest-ranked Angus bulls were chosen. From Ranch B, the 10 lowest- and 10 highest-ranked Brangus bulls were selected. At Ranch C, where three breeds were represented, the 10 lowest- and 10 highest-ranked bulls were selected using a proportional approach to maintain breed representation, resulting in 3 Brangus, 3 SimAngus, and 4 Deseret Red bulls in each RFI group. Therefore, plasma samples from 30 HRFI and 30 LRFI bulls were used to evaluate plasma metabolites, hormones, and natural ^1^³C and ^15^N abundance at d 0 and 56.

### Quantitative metabolomics using liquid chromatography–triple quadrupole mass spectrometry (LC-ESI-MS)

LC-MS grade solvents including acetonitrile, methanol, isopropyl alcohol, and formic acid (≥99.0% purity) were purchased from Fisher Scientific (Hanover Park, IL, USA), while ethanol, pyridine, and phenylisothiocyanate were purchased from Sigma Aldrich (St. Louis, MO, USA). Milli-Q water (EMD Millipore, Billerica, MA, USA) was used for aqueous mobile phases. Plasma samples were processed following the manufacturer’s protocol using the MxP® Quant 500 kit (biocrates Life Sciences, AG, Innsbruck, Austria). After thawing on ice, calibration standards and QCs were reconstituted in water, vortexed, and mixed at 1200 rpm for 15 min. 10 µL each of plasma samples, standards, and phosphate buffer were added to a 96-well plate, dried under nitrogen for 30 min, derivatized with phenylisothiocyanate for 60 min, and dried again for 60 min. Metabolites were extracted in 5 mM ammonium acetate in methanol for 30 min and centrifuged at 500 g for 2 min, then diluted 1:1 with water for LC-MS analysis. For FIA-MS analysis, 10 µL of each sample was mixed with 490 µL of biocrates kit solvent, mixed at 600 rpm for 10 min, and transferred to the LC autosampler. Chromatographic separation was performed using a Waters I-Class UPLC coupled to a Xevo TQ-S MS (Waters Corporation, Milford, MA, USA) equipped with a biocrates C18 column. The mobile phases were water with 0.2% formic acid (A) and acetonitrile with 0.2% formic acid (B), delivered in a gradient flow of 0% to 100% B over 4.5 min at 0.8 mL/min, followed by a high-flow wash and re-equilibration. The total gradient run time for both positive and negative modes was 5.80 min. However, the negative mode acquisition featured a different %B composition specifically between 2.00 and 4.50 min compared to the positive mode. Injection volumes were 5 µL (positive mode) and 15 µL (negative mode). FIA-MS/MS analysis used an isocratic method with 100% methanol containing biocrates FIA additives at a flow rate of 0.03 mL/min and an injection volume of 20 µL. Data was processed using biocrates MetIDQ software. Quality control included triplicate concentrations (low, mid, high) of QC samples provided by the manufacturer. Metabolites were excluded if more than 30% of their values were not detected across groups, as this would prevent reliable imputation. However, no metabolite met this exclusion criterion. A comprehensive listing of biochemical names, abbreviations, and PubChem CID of metabolites, organized by class, is provided in Supplementary Table S1.

### Cortisol and testosterone concentration

Circulating plasma cortisol concentrations were quantified using the automated Immulite® 2000 XPi cortisol Immunoassay kit (Cat. No. L2KCO2, Siemens Healthcare, CA, USA). Briefly, per the manufacturer’s recommendations, a new calibration curve (range of 10 to 500 ng/mL) was run for each new lot number of the cortisol kit. Commercially available tri-level (26.8, 149, and 254 ng/mL) quality control samples (Lyphocheck Immunoassay Plus Control, Cat. No 370, Bio-Rad, Hercules, CA, USA) were run daily before the experimental samples. Quality control samples were considered to pass if the CV was below 10% for each level and the replicates were within the manufacturer’s reported range. Before analysis, samples were thawed at room temperature and ran in duplicates. An internal calculation of quantification was reported for each duplicate, and these values were averaged to determine the CV. All concentrations were reported to have a CV of less than 10%.

Plasma testosterone concentrations were measured using an automated Immulite® 2000 XPi testosterone Immunoassay kit (catalog number L2KTW2, Siemens Healthcare, CA, USA). The kit was adjusted according to the manufacturer’s recommendations for each lot number. It had a calibration range of 20 to 1600 ng/mL (0.7–55 nmol/L) and a sensitivity of 15 ng/mL. To ensure accuracy, commercially available quality control samples (Lyphocheck Immunoassay Plus Control, catalog number 370, Bio-Rad, CA, USA) with tri-level concentrations (26.8, 149, and 254 ng/ml) were run daily before analyzing the samples. The quality control samples were considered acceptable if the CV was below 10% for each level and the replicates fell within the manufacturer’s specified range. Prior to analysis, the samples were thawed to room temperature and assessed in duplicates. The time required to obtain the first results was 65 min. The quantification of each duplicate was internally calculated, and the values were averaged. All reported concentrations had a CV ≤ 10%.

### Natural isotope’s abundance

Frozen red blood cells (RBC) samples were re-suspended with 9 vol. 0.9% NaCl solution and mixed at room temperature for 15 min at 2 Hz on an orbital shaker. The tubes were then centrifuged at 714 x g for 20 min. The saline solution from the centrifuged tubes was discarded after centrifugation. This rinsing procedure was repeated two additional times. After the third rinse, a 500-µL aliquot of the RBC pellet was collected and freeze-dried for isotopic analysis. Plasma samples were also freeze-dried for isotopic analysis. Plasma and RBC samples were analyzed for total C and N using a CHNS analyzer through the Dumas dry combustion method (Vario Microcube, Elementar Americas Inc., Ronkonkoma, NY, USA) coupled to an isotope ratio mass spectrometer (IRMS; IsoPrime 100, Elementar, Americas Inc., Ronkonkoma, NY, USA). Certified standards of L-glutamic acid (USGS40, USGS41; United States Geological Survey) were used for isotope ratio mass spectrometer calibration (IRMS). Isotope ratios were as follows: δ^1^³C of −26.39, + 37.63‰, and δ^15^N of −4.52, 47.57‰ for USGS40 and USGS41, respectively. Calibration of the IRMS was conducted according to Cooket al. (2017), with a precision of ≤ 0.06 ‰ for ^15^N and ≤ 0.08 ‰ for ^13^C.

The isotope ratio for ^13^C/^12^C was calculated as:


$$\delta^{13}C=\left({\;}^{13}C{/}^{12}C\;\mathrm{sample}{-}^{13}C{/}^{12}C\;\mathrm{reference}\right)/\;\left({\;}^{13}C{/}^{12}C\;\mathrm{reference}\times 1000\right)$$

Where δ^1^³C is the C isotope ratio of the sample relative to Pee Dee Belemnite (PDB) standard (‰), ^13^C/^12^C sample is the C isotope ratio of the sample, and ^13^C/^12^C reference is the C isotope ratio of PDB standard.

The isotope ratio for ^15^N/^14^N was calculated as:


$$\delta^{1}{\;}^{5}N=\left({\;}^{15}N{/}^{14}N\;\mathrm{sample}\;{-}^{15}N{/}^{14}N\;\mathrm{reference}\right)/\;\left({\;}^{15}N{/}^{14}N\;\mathrm{reference}\times 1000\right)$$

Where δ^15^N is the N isotope ratio of the sample relative to atmospheric nitrogen (‰), ^15^N/^14^N sample is the N isotope ratio of the sample, and ^15^N/^14^N reference is the N isotope ratio of atmospheric N (standard).

### Statistical analysis

In accordance with established metabolomics pipelines (Goodacre et al. 2007; Chen et al. 2022; Anwardeen et al. 2023), we used a standard workflow in which multivariate analyses reveal global metabolic patterns, univariate analyses identify the specific metabolites driving those patterns, pathway enrichment analyses place those metabolites within their biological context, and ROC curves validate the relevance of selected metabolites as potential biomarkers.

First, multivariate statistics methods were applied to facilitate the visualization of data obtained from the metabolomic analysis of the top and bottom bulls of each group on d 0 and 56 using MetaboAnalyst 5.0® software (Pang et al. 2021). After normalization using Log transformation (base 10) to reduce the impact of large fold changes and stabilize the variance across the dataset, and Pareto scaling (mean-centered and divided by the square root of the standard deviation) to identify dissimilarities between groups and reduce the dominance of high-abundance metabolites while retaining biologically relevant variance, unsupervised Principal Component Analysis (PCA) and supervised classifications, including Partial Least Square—Discriminant Analysis (PLS-DA) and Sparse Partial Least Squares Discriminant Analysis (sPLS-DA) models, were performed. PCA was initially applied to separate the LRFI and HRFI groups and unbiasedly reduce the complexity of multidimensional data sets. General trends and patterns were identified from the principal component (PC) score plots to determine sample clustering, and a loadings plot was used to identify the metabolites primarily responsible for the separation observed between the groups. Following PCA, supervised classification was used to maximize the separation between the two groups. The metabolites, hormones and isotopes were further analyzed based on their Variable Importance in the Projection (VIP) values, which ranked the top 20 compounds for d 0 and d 56, based on their contribution to the discriminant model, and the coefficient of determination (*R*^2^) and the cross-validated Q-squared (*Q*^2^) were reported. After completing the primary analysis, a Pathway analysis was conducted using MetaboAnalyst 5.0® software, to identify metabolites and specific patterns that exhibited notable differences between LRFI and HRFI groups, considering each pathway’s number of Hits, the *P* value, and the False Discovery Rate (FDR) value.

Next, data were also analyzed using univariate analyses following a general complete block design (GCBD) using the procedure MIXED of SAS (SAS 9.4 Inst. Inc., Cary, NC) including 2 RFI groups (LRFI and HRFI), and 3 blocks (ranches: bulls’ consigner). Each animal was considered an experimental unit, and block was considered a random effect. The model (Y_ijk_ = µ + *β*_i_ + *τ*_j_ + *ε*_ijk_) included the effect of the mean (µ), block (*β*), treatment (*τ*), and the experimental error (*ε*). When the residuals did not follow a normal distribution, a transformation was performed using the PROC TRANSREG in SAS, with the λ value estimated as the transformation parameter to meet model assumptions. Significance was declared when *P* values < 0.05.

Finally, to evaluate the predictive performance of the key metabolites identified through multivariate and univariate analyses, based on their statistical significance and contribution to group separation, ROC curve analysis was performed using MetaboAnalyst 5.0®.The results are presented as the Area Under the Curve (AUC) with 95% confidence intervals (CI).

## Results

Table 2 shows the results of the performance test of bulls with differing RFI. Among the initial 302 bulls included in the experiment, 4 animals were removed due to illnesses (*n* = 1) or irregularities in behavior patterns (*n* = 3). The remaining 298 bulls were ranked into three groups based on their RFI: 94 HRFI, 103 LRFI, and 101 medium RFI, determined by standard deviations (SD) from the mean RFI of their respective group (mean ± 0.5 SD). As expected, according to the performance test, initial BW (*P* = 0.41), final BW (*P* = 0.46), and ADG (*P* = 0.42) were similar between groups, but DMI was smaller for the LRFI group (10.8 ± 1.1 kg/d) than for HRFI group (14.5 ± 1.1 kg/d; *P* < .001).

**Table 2:** Performance of bulls classified according to RFI.

	HRFI (*n* = 94)	LRFI (*n* = 103)	*P* value
**Initial BW: day 0 (Kg)**	410 ± 16	411 ± 15.4	0.41
**Final BW: day 56 (Kg)**	504 ± 24	513 ± 20	0.46
**DMI (kg/d)**	14.5 ± 1.1	10.8 ± 1.1	< 0.001
**ADG (kg/d)**	1.55 ± 0.01	1.67 ± 0.08	0.42

BW, Body weight; DMI, Dry matter intake; ADG, Average daily gain; HRFI, High residual feed intake; LRFI, low residual feed intake.

### Multivariate analysis

**Figure 2:**
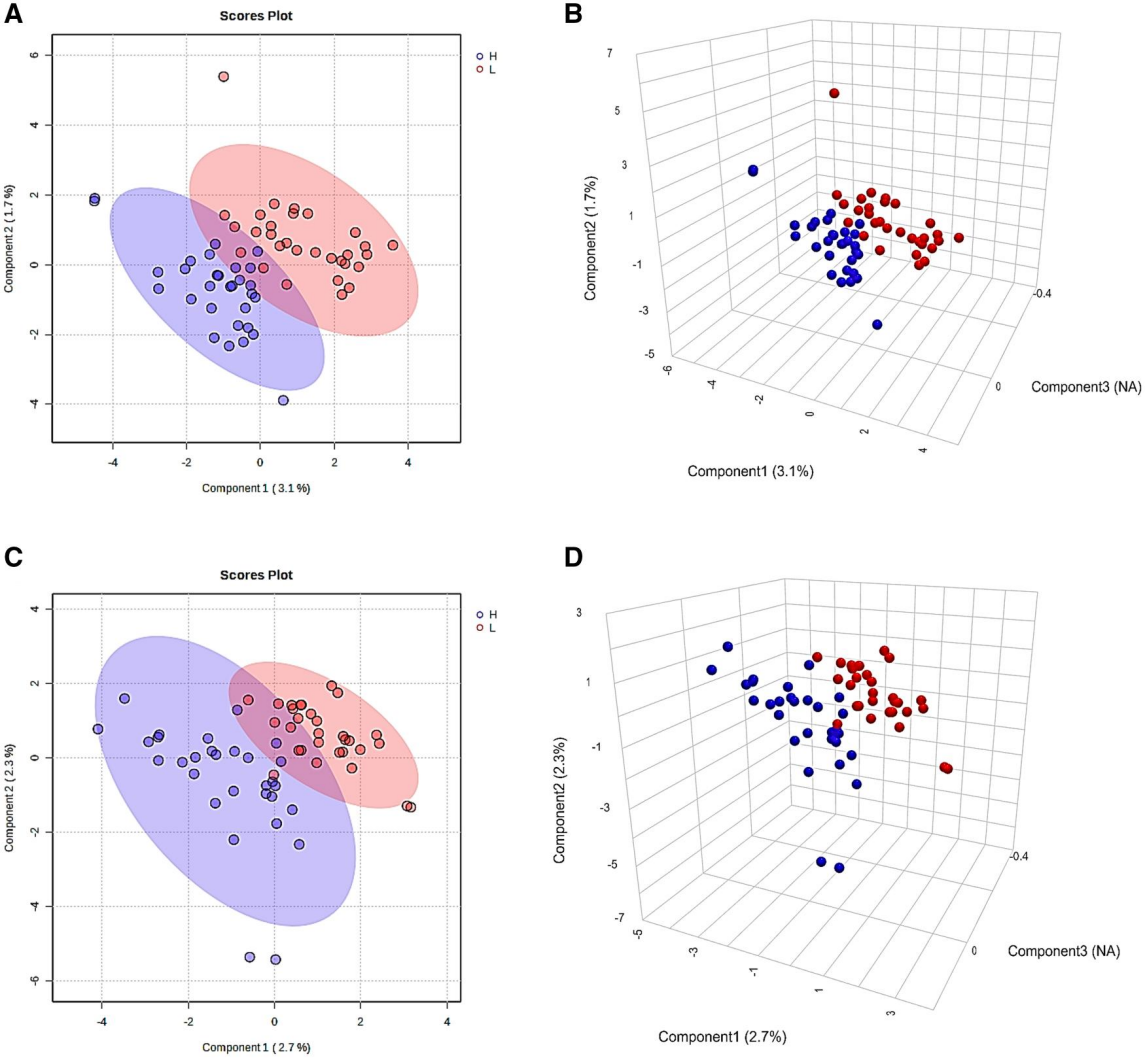
Multivariate analysis of plasma metabolites of LRFI (red circles) and HRFI (blue circles) bulls on d 0 (A, B) and D 56 (C, D). Every circle represents an individual animal. Panels A and C represent sPLS-DA analysis of two components where ellipses represent 95% confidence interval. Panels B and D represents sPLS-DA analysis of three components.

The PCA analysis showed low separable clustering between LRFI and HRFI bulls. In contrast, the sPLS-DA model showed moderate separation between groups, with an overlap or similarity among animals differing in RFI. Figure 2 shows the score plots of the sPLS-DA analysis of plasma metabolites of LRFI and HRFI bulls at d 0 and d 56. According with sPLS-DA cross validation, the quality control metrics indicated high *R*^2^ values at d 0 (*R*^2^ = 0.72 and *R*^2^ = 0.89 for the first and the second component respectively) but low *Q*^2^ values at the same day (*Q*^2^ = 0.007 and *Q*^2^ = 0.05 for the first and the second component respectively). Moreover, at d 56, quality control metrics indicated high *R*^2^ (*R*^2^ = 0.78 and *R*^2^ = 0.90 for the first and the second component), and a negative *Q*^2^ value for the first component (*Q*^2^ = -0.04) and a relative low value for the second component (*Q*^2^ = 0.02). These results indicate that the model had good performance in fitness, but the low *Q*^2^ value indicates poor predictive value.

**Figure 3:**
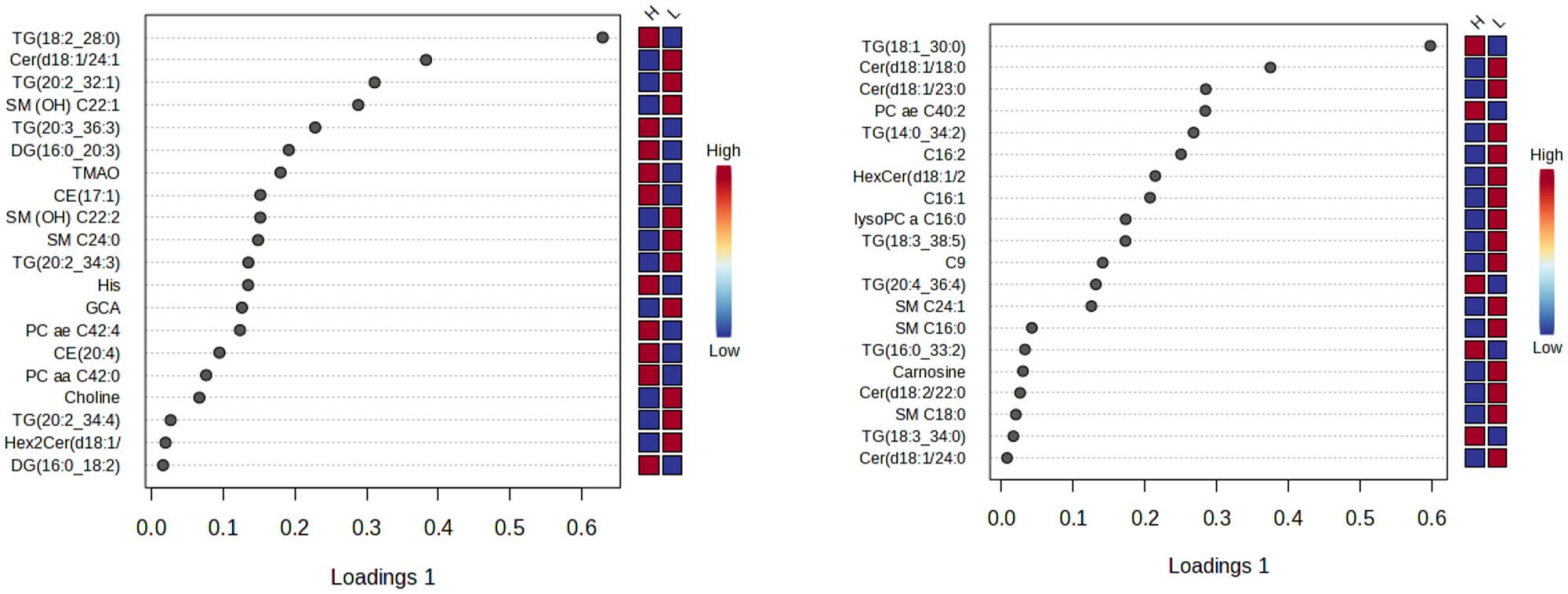
VIP plot obtained from the comparison between LRFI and HRFI groups. The color boxes indicate whether metabolite concentration is increased (red) or decreased (blue). The most discriminating metabolites are presented in descending order of their coefficient scores.

The top 20 greatest VIP plot between LRFI and HRFI bulls at d 0 and d 56 are presented in Fig. 3. As shown by the VIP plot, the most discriminant variables contributing to separating the 2 groups at d 0 were: TG(18:2_28:0), Cer(d18:1/24:1), TG(20:2_32:1), SM(OH)C22:1, TG(20:3_36:3); DG(16:0_20:3), TMAO, ce(17:1), SM(OH)C22:2, SMC24:0, TG(20:2_34:3), His, GCA, PC ae C42:4, ce(20:4), PC aa C42:0, Choline, TG(20:2_34:4), Hex2Cer(18:1/14:0), and DG(16:0_18:2). The top discriminant variables contributing to separating the 2 groups at d 56 were: TG(18:1_30:0), Cer(d18:1/18:0), Cer(d18:1/23:0), PC ae C40:2, TG(14:0_34:2), C16:2, HexCer(d18:1/20:0), C16:1, lysoPC a C16:0, TG(18:3_38:5), C9, TG(20:4_36:4), SM C24:1, SM C16:0, TG(16:0:33:22), Carnosine, SM C18:0, TG(18:3_34:0), and Cer(d18:1/24:0).

**Figure 4:**
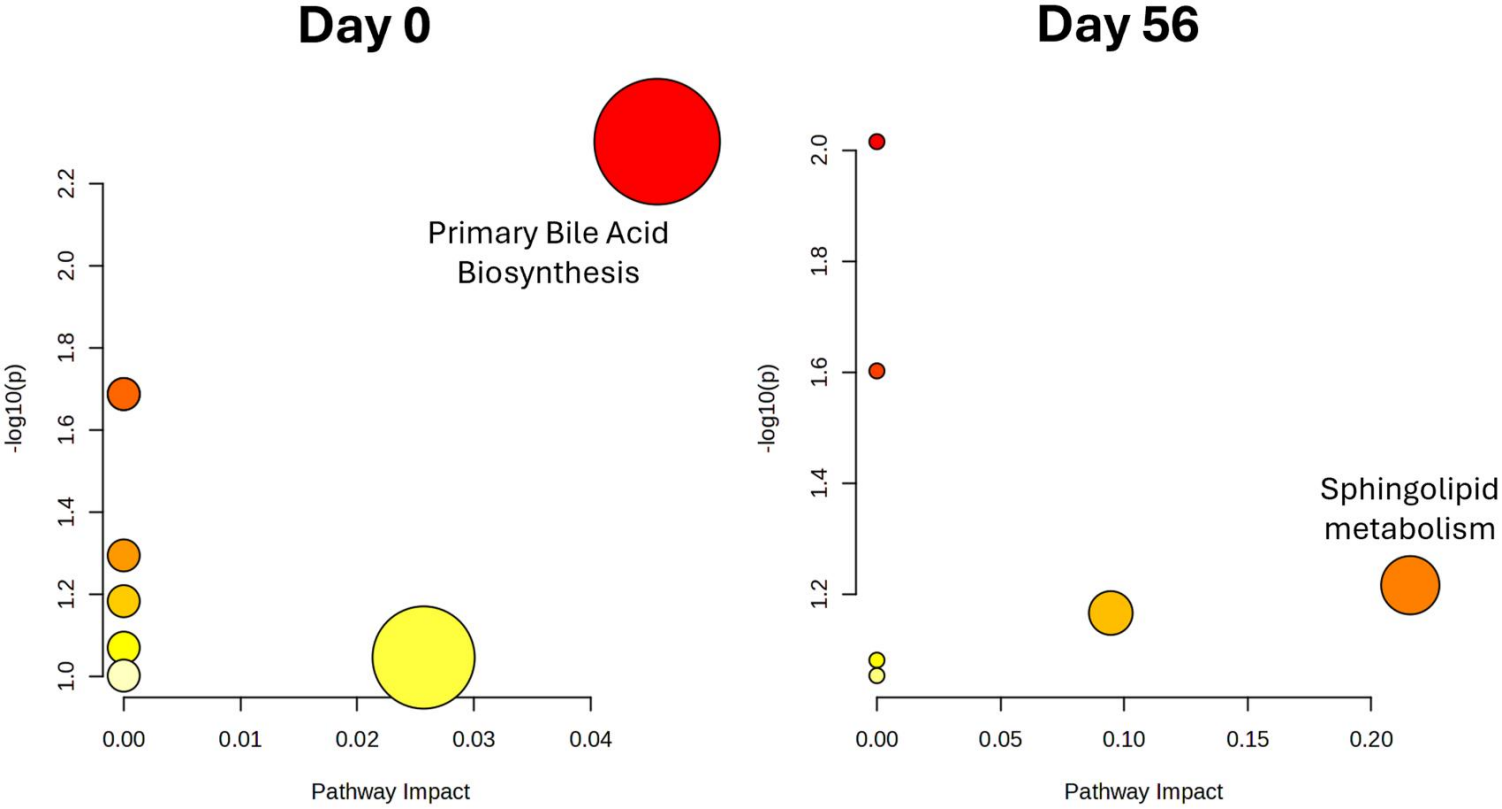
Scatterplot mapping significant metabolites to the *Bos taurus* library among LRFI bulls on day 0 (*P* = 8.54E-03) and day 56 (*P* = 4.11e-02).

Finally, the pathway analysis using *Bos taurus* library, identified 5 significant metabolites pathways at d 0 (Fig. 4), including: primary bile acid (BA) biosynthesis (*P* = 0.003), valine, leucine and isoleucine biosynthesis (*P* = 0.026), taurine and hypotaurine metabolism (*P* = 0.026), glycerolipid metabolism (*P* = 0.052), and pantothenate and CoA biosynthesis (*P* = 0.061). Among these pathways, primary BAs biosynthesis presented the highest impact value (Impact = 0.05), involving Deoxycholic acid glycine conjugate, Lithocholic acid glycine conjugate, 9,12-Hexadecadienoylcarnitine, 2-trans, 4-cis-Decadienoylcarnitine with increased abundance in LRFI group, and 2-trans , 4-cis-Decadienoylcarnitine which was decreased in LRFI group.

While 6 pathways were enriched at d 56 (Fig. 4), including: sphingolipid metabolism (*P* = 0.04), linoleic acid metabolism (*P* = 0.009), alpha-Linoleic acid metabolism (*P* = 0.025), arachidonic acid metabolism (*P* = 0.068), glycerophospholipid metabolism (*P* = 0.068), and fatty acid biosynthesis (*P* = 0.088). Among these pathways, sphingolipid metabolism presented the highest impact value (Impact = 0.27), involving Myristic acid, Creatinine, Taurodeoxycholic acid, 3-Hydroxyglutaric acid, Phosphatidylcholine diacyl C38:0, N-Tetracosanoylsphinganine, Ceramide (d18:1/23:0), Ceramide (18:1/18:0), Ceramide (18:2/14:0) and DG-O(14:0_18:2) with increased concentration in LRFI group. While Tracylglycerol(54:4), Phosphatidylcholine diacyl C42:0, Tracylglycerol(48:1), Tracylglycerol(56:7), Tracylglycerol(56:8) and TG(17:1_38:7), which were less abundant in LRFI group. In Table 3 are presented the metabolites involved in the highlighted pathways at d 0 and 56 for LRFI bulls.

**Table 3:** Metabolites involved in enriched pathways at d 0 and 56 for LRFI bulls.

Pathways highlighted at d 0	Metabolites involved
**Primary Bile Acid Biosynthesis**	Cholesterol
	7α-hydroxycholesterol
	7-ketocholesterol
	7α-hydroxy-4-cholesten-3-1
	Chenodeoxycholic acid
	Cholic acid
**Valine, Leucine, and Isoleucine Biosynthesis**	Pyruvate
	Acetolactate
	α-ketovalerate
	Valine
	α-ketoisovalerate
	Leucine
	α-keto-*β*-methylvalerate
	Isoleucine
**Taurine and Hypotaurine Metabolism**	Cysteine
	Hypotaurine
	Taurine
**Glycerolipid Metabolism**	Glycerol-3-phosphate
	Diacylglycerol
	Triacylglycerol
	Phosphatidic acid
**Pantothenate and CoA Biosynthesis**	3-phosphoglycerate
	Serine
	Glycine
	Threonine

Pathways highlighted at d 56	Metabolites involved
**Sphingolipid Metabolism**	Ceramide
	Sphingosine
	Sphingosine-1-phosphate (S1P)
	Sphingomyelin
**Linoleic Acid Metabolism**	Linoleic acid
	9-hydroxy-octadecenoic acid
	3-hydroxy-octadecenoic acid
	9,10-epoxyoctadecenoic acid
	12,13-epoxyoctadecenoic acid
**Alpha-Linolenic Acid Metabolism**	Alpha-linolenic acid
	Stearidonic acid
	Eicosatetraenoic acid
	Eicosapentaenoic acid
**Glycerophospholipid Metabolism**	Phosphatidylcholine
	Phosphatidylethanolamine
	Phosphatidylserine
	Phosphatidylinositol
**Arachidonic Acid Metabolism**	Arachidonic acid
	Prostaglandin G2
	Prostaglandin H2
	Prostaglandins (PGE2, PGD2, PGF2α)
	Thromboxane A2
	Leukotrienes (LTB4, LTC4, LTD4, LTE4)
	Epoxyeicosatrienoic acids
	Hydroxyeicosatetraenoic acids
**Fatty Acid Biosynthesis**	Acetyl-CoA
	Malonyl-CoA
	Palmitic acid
	Stearic acid
	Oleic acid

### Univariate analysis

**Figure 5:**
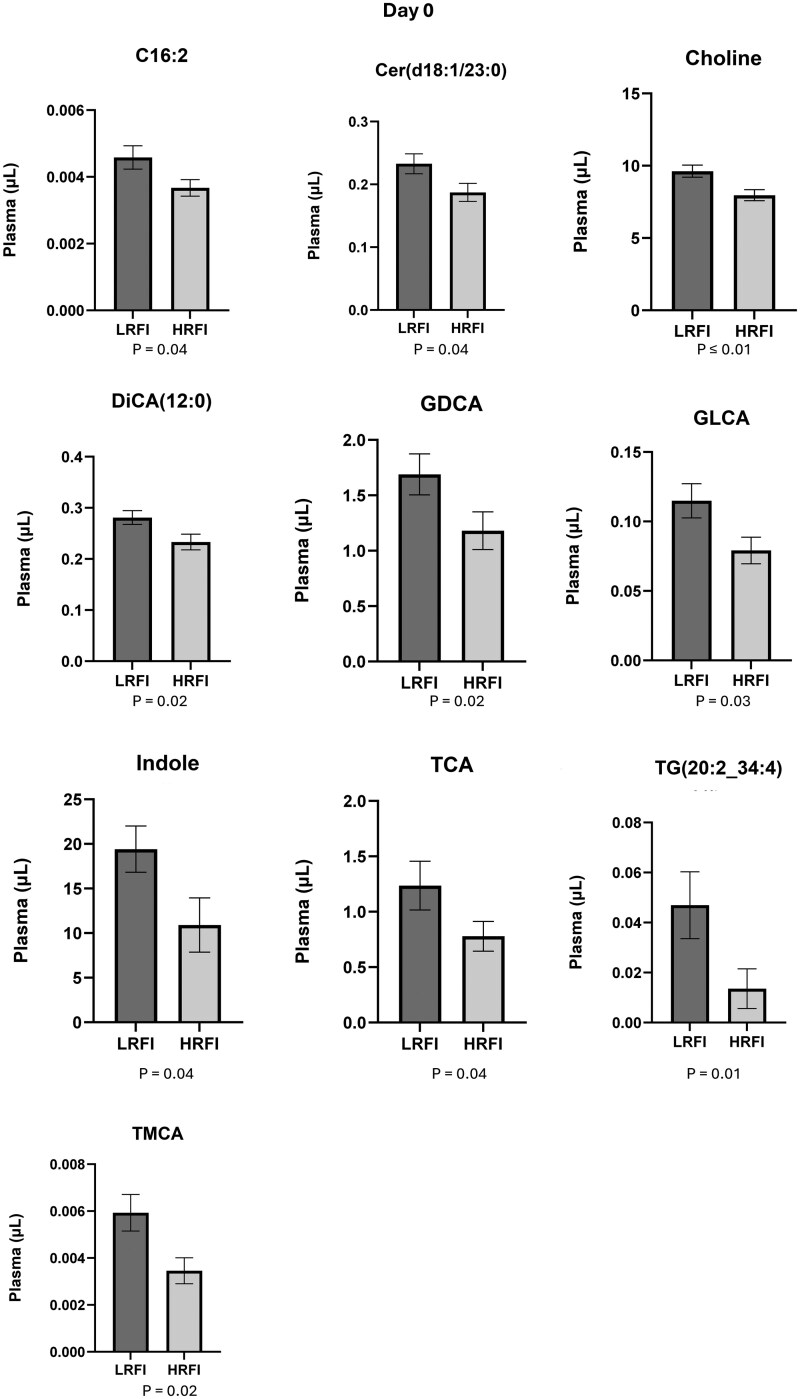
Significant metabolites increased in LRFI compared with HRFI bulls (*P* < .05) at d 0, expressed as mean ± SEM.

**Figure 6:**
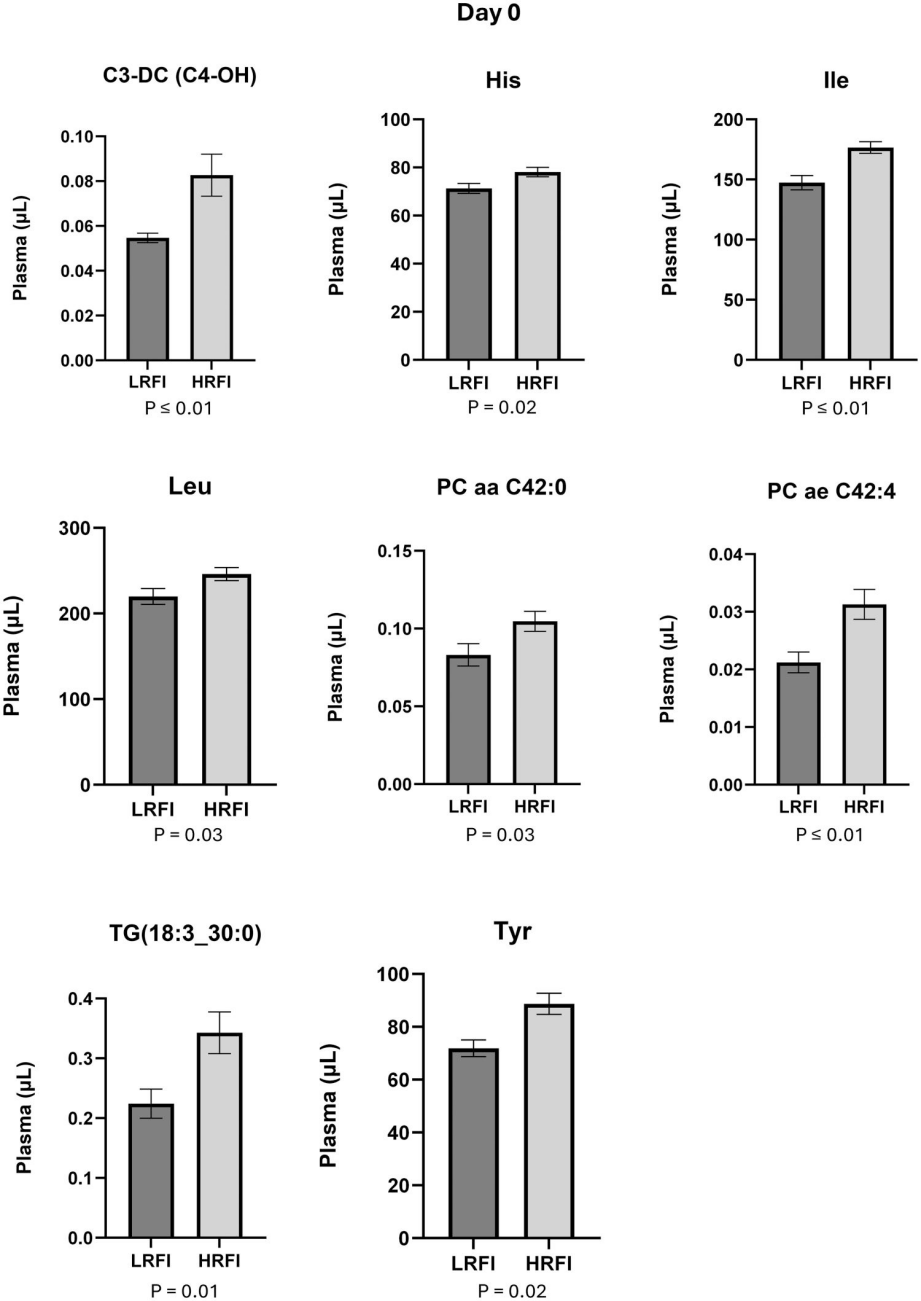
Significant metabolites decreased in LRFI compared with HRFI bulls (*P* < .05) at d 0, expressed as mean ± SEM.

**Figure 7:**
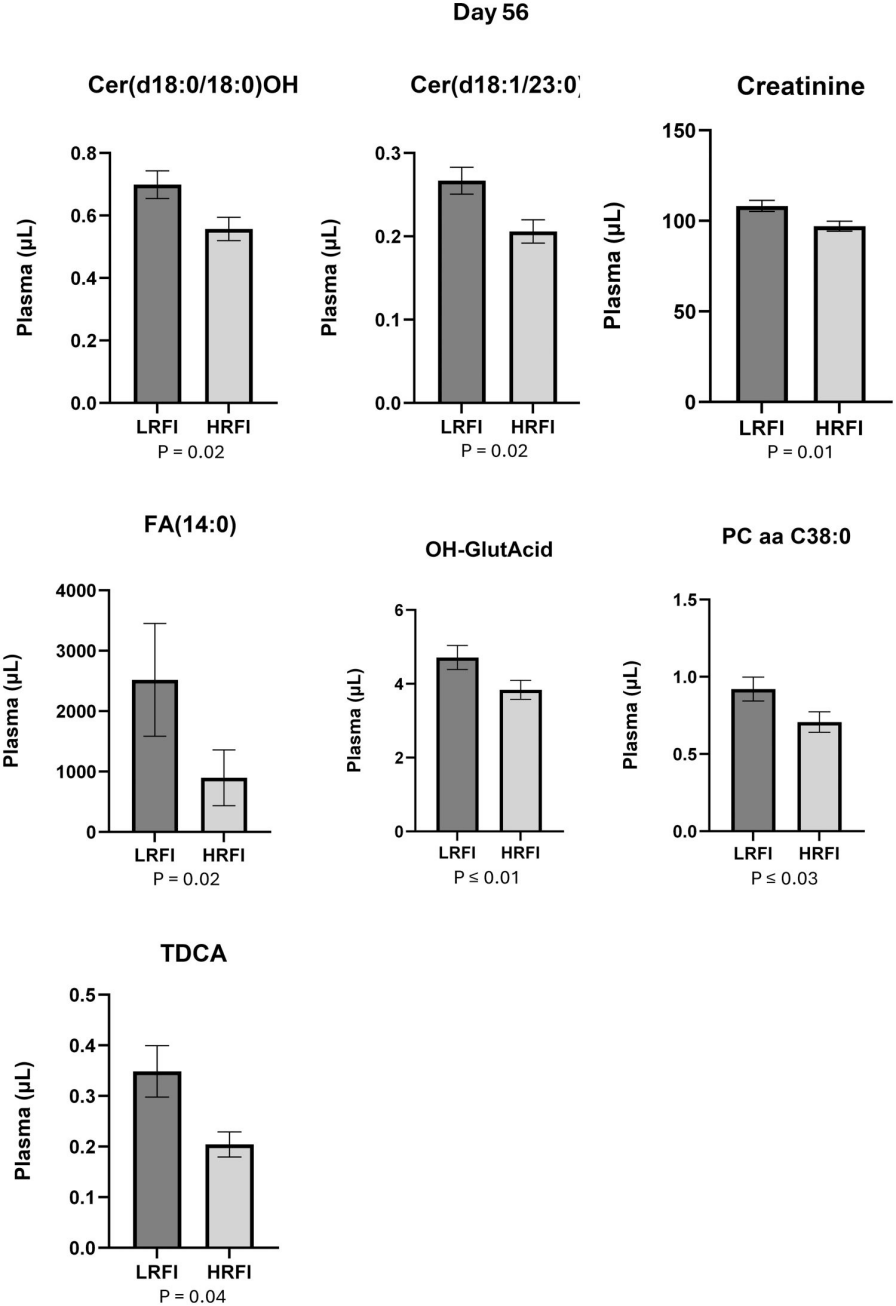
Significant metabolites increased in LRFI compared with HRFI bulls (*P* < .05) at d 56, expressed as mean ± SEM.

**Figure 8:**
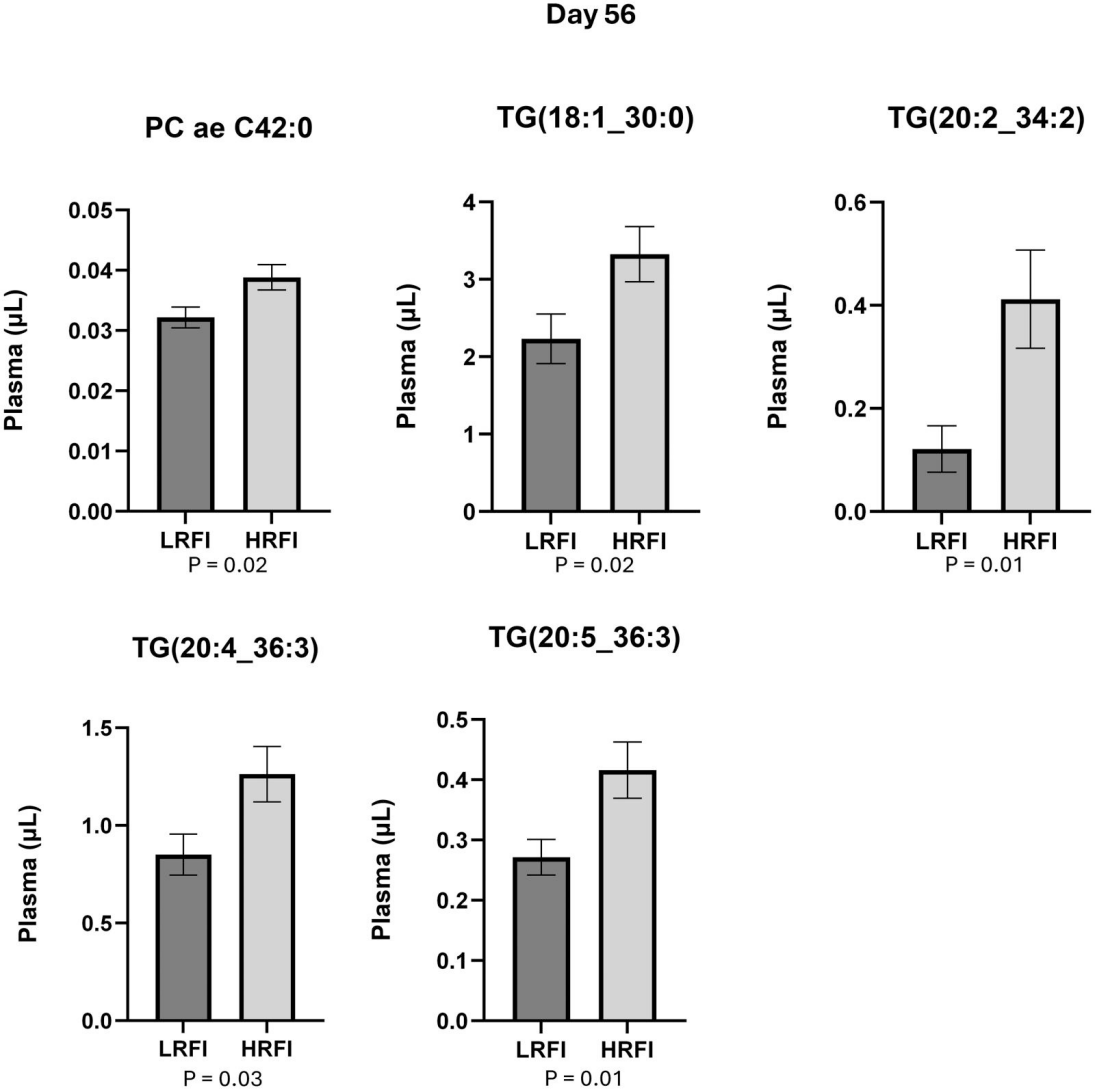
Significant metabolites decreased in LRFI compared with HRFI bulls (*P* < .05) at d 56, expressed as mean ± SEM.

Out of the 628 compounds that were included in the analysis, the plasma concentration of 18 compounds was statistically different (*P* ≤ .05) between LRFI and HRFI bulls at d 0. Ten of them had greater concentrations in LRFI (Fig. 5) and 8 had greater concentration in HRFI (Fig. 6). While at d 56, 12 compounds were statistically different (*P* ≤ .05) between LRFI and HRFI bulls. Seven of them had greater concentration for LRFI (Fig. 7) and 5 had greater concentration for HRFI bulls (Fig. 8).

*Acylcarnitines*: plasma concentration of 40 acylcarnitines are presented in Supplementary Table S2. All the evaluated acylcarnitines were detected in plasma samples and the most abundant were: L-Carnitine (C0; d 0: 12.92 ± 0.34 µM; d 56: 12.64 ± 1.12 µM), and L-Acetylcarnitine (C2; d 0: 7.85 ± 2.89 µM; d 56: 6.30 ± 1.01 µM), and the less abundant were: Pimelylcarnitine (C7-DC; d 0: 0.002 ± 0.00 µM; d 56: 0.002 ± 0.00 µM), and Linolaidylcarnitine (C18:2; d 0: 0.004 ± 0.00 µM; d 56: 0.004 ± 0.00 µM). At d 0, the concentration of 9,12-Hexadecadienoylcarnitine (C16:2; LRFI: 0.004 ± 0.00 µM/ml; HRFI: 0.004 ± 0.00 µM/ml; *P* = 0.04), was significantly increased (Fig. 5) for LRFI group and Malonylcarnitine (C3-DC (C4-OH); LRFI: 0.05 ± 0.00 µM/ml; HRFI: 0.08 ± 0.00 µM/ml; *P* = 0.004), was significantly decreased (Fig. 6) for LRFI group, while no differences were observed at d 56.

*Alkaloids:* the only evaluated compound was Trigonelline, whose plasma concentration is presented in Supplementary Table S3: Trigonelline (d 0: 0.05 ± 0.01 µM; d 56: 0.06 ± 0.01 µM). No statistical differences were observed at both d 0 and 56 (*P* > .05).

*Amine Oxides:* the only evaluated compound was Trimethylamine N-oxide, whose plasma concentration is presented in Supplementary Table S4: Trimethylamine N-oxide (TMAO; d 0: 3.03 ± 0.68 µM; d 56: 2.24 ± 0.47 µM). No statistical differences were observed at both d 0 and 56 (*P* > .05).

*Amino acids*: results of plasma concentration of 20 amino acids are presented in Supplementary Table S5. All the evaluated amino acids were detected in plasma samples and the most abundant were Glycine (Gly; d 0: 414 ± 16.51 µM; d 56: 431 ± 15.06 µM) and Glutamine (Gln; d 0: 367 ± 8.84 µM; d 56: 372 ± 8.94 µM) at both, d 0 and 56. Additionally, the less abundant amino acids in plasma samples were Aspartate (Asp; d 0: 9.53 ± 1.47 µM; d 56: 12.61 ± 1.35 µM), Cysteine (Cys; d 0: 27.11 ± 0.83 µM; d 56: 27.23 ± 0.80 µM), and Methionine (Met; d 0: 29.68 ± 1.34 µM; d 56: 29.78 ± 1.24 µM). At d 0 Histidine (His; LRFI: 71.30 ± 2.08 µM/ml; HRFI: 78.09 ± 1.94 µM/ml; *P* = 0.02), Isoleucine (Ile; LRFI: 147.34 ± 5.9 µM/ml; HRFI: 176.59 ± 4.83 µM/ml; *P* = 0.0003), Leucine (Leu; LRFI: 219 ± 9.29 µM/ml; HRFI: 246 ± 7.61 µM/ml; *P* = 0.03), and L-Tyrosine (Tyr; LRFI: 71.85 ± 3.17 µM/ml; HRFI: 88.69 ± 4.02 µM/ml; *P* = 0.02) were significantly decreased for LRFI compared to the HRFI group (Fig. 6). While no differences were observed at d 56 (*P* > .05).

*Amino acids related compounds*: plasma concentration of 30 amino acids related compounds are presented in Supplementary Table S6. All the evaluated amino acids related compounds were detected in plasma samples and the most abundant in plasma samples were Creatinine (d 0: 104.62 ± 3.23 µM; d 56: 108.22 ± 3.08 µM) and Ornitine (Orn; d 0: 80.28 ± 3.21 µM; d 56: 82.96 ± 3.85 µM), and the less abundant were Phenylalanine betaine (PheAlaBetaine; d 0: 0.004 ± 0.00 µM; d 56: 0.004 ± 0.00 µM) and Lenticin (TrpBetaine d 0: 0.01 ± 0.00 µM; d 56: 0.01 ± 0.00 µM). At d 0 there were no statistical differences between groups. While at d 56 Creatinine (LRFI: 108.22 ± 3.08 µM/ml; HRFI: 97.05 ± 2.76 µM/ml; *P* = 0.01) was increased for LRFI group (Fig. 7).

*Bile acids:* plasma concentration of 14 bile acids is presented in Supplementary Table S7. All the evaluated BAs were detected in plasma samples and the most abundant were Cholic acid (CA; d 0: 8.73 ± 1.13 µM; d 56: 9.01 ± 1.11 µM), and Glycocholic acid (GCA; d 0: 5.40 ± 0.67 µM; d 56: 4.52 ± 0.76 µM), and the less abundant were Glycoursodeoxycholic acid (GUDCA; day 0: 0.001 ± 0.00 µM; d 56: 0.001 ± 0.00 µM), and Glycolithocholic acid 3-sulfate (GLCAS; d 0: 0.004 ± 0.00 µM; d 56: 0.005 ± 0.00 µM). At d 0 Glycodeoxycholic acid (GDCA; LRFI: 1.69 ± 0.19 µM/ml; HRFI: 1.18 ± 0.17 µM/ml; *P* = 0.04), Glucuronic acid (GLCA; LRFI: 0.11 ± 0.01 µM/ml; HRFI: 0.08 ± 0.01 µM/ml; *P* = 0.03), Taurocholic acid (TCA; LRFI: 1.24 ± 0.22 µM/ml; HRFI: 0.78 ± 0.13 µM/ml; *P* = 0.04), and Tauromuricholic acid (TMCA; LRFI: 0.01 ± 0.00 µM/ml; HRFI: 0.003 ± 0.00 µM/ml; *P* = 0.02) were significantly increased for LRFI group (Fig. 5). While Taurodeoxycholic (TDCA; LRFI: 0.35 ± 0.05 µM/ml; HRFI: 0.20 ± 0.02 µM/ml; *P* = 0.04) was increased in LRFI bulls at d 56 (Fig. 7).

*Biogenic Amines:* plasma concentration of 9 biogenic amines is presented in Supplementary Table S8. All the evaluated biogenic amines were detected in plasma samples and the most abundant were beta-Alanine (beta-Ala; d 0: 1.55 ± 0.08 µM; d 56: 1.58 ± 0.08 µM), and Putrescine (d 0: 0.16 ± 0.01 µM; d 56: 0.14 ± 0.01 µM), and the less abundant were Phenylethylamine (PEA; day 0: 0.001 ± 0.00 µM; d 56: 0.001 ± 0.00 µM), and Histamine (d 0: 0.004 ± 0.00 µM; d 56: 0.004 ± 0.00 µM). Neither group was statistically different at both d 0 and 56.

*Carboxylic Acids*: plasma concentration of 7 carboxylic acids is presented in Supplementary Table S9. All the evaluated carboxylic acids were detected in plasma samples and the most were D-Lactic acid (Lac; d 0: 7318 ± 1024 µM; d 56: 6239 ± 795 µM) and Hippuric acid (HipAcid; d 0: 54.02 ± 3.69 µM; d 56: 49.84 ± 3.93 µM), and the less abundant were Tetradecanedioic acid (DiCA (14:0); d 0: 0.04 ± 0.01 µM; d 56: 0.03 ± 0.01 µM) and Dodecanedioic acid (DiCA(12:0); d 0: 0.28 ± 0.01 µM; d 56: 0.24 ± 0.02 µM). At d 0 Dodecanedioic acid (DiCA(12:0); LRFI: 0.28 ± 0.01 µM/ml; HRFI: 0.23 ± 0.02 µM/ml; *P* = 0.02) was significantly increased for LRFI group (Fig. 5). While 3-Hydroxyglutaric acid (OH-Glut-Acid; LRFI: 4.71 ± 0.32 µM/ml; HRFI: 3.84 ± 0.26 µM/ml; *P* = 0.01) was increased in LRFI at d 56 (Fig. 7). When analyzing the significance of the covariate (d 0) on the concentration of all evaluated carboxylic acids, only OH-Glut-Acid (*P* = 0.007) had a significant effect of the covariate suggesting that the initial metabolic state at d 0 influenced the response of this metabolite at d 56.

*Ceramides*: plasma concentration of 28 ceramides are presented in Supplementary Table S10. All the evaluated ceramides were detected in plasma samples and the most abundant ones were: Cer(d18:1/18:0(OH)) (d 0: 0.43 ± 0.02 µM; d 56: 0.46 ± 0.02 µM) and Cer(d18:1/20:0(OH)) (d 0: 0.34 ± 0.03 µM; d 56: 0.36 ± 0.03 µM) and the less abundant were Ceramide (d18:1/26:1) (Cer(d18:1/26:1); d 0: 0.02 ± 0.00 µM; d 56: 0.02 ± 0.00 µM) and Cer(d18:2/18:1) (d 0: 0.02 ± 0.00 µM; d 56: 0.02 ± 0.00 µM). Ceramide (d18:1/23:0) was significantly increased for LRFI group at both d 0 (LRFI: 0.23 ± 0.02 µM/ml; HRFI: 0.19 ± 0.01 µM/ml; *P* = 0.04; Fig. 5) and d 56 (LRFI: 0.27 ± 0.02 µM/ml; HRFI: 0.21 ± 0.01 µM/ml; *P* = 0.02; Fig. 7).

*Cholesterol Esters:* plasma concentration of 22 cholesterol esters is presented in Supplementary Table S11. All the evaluated cholesterol esters were detected in plasma samples and the most abundant were Cholesteryl 1-linoleoate (ce(18:2); d 0: 27164 ± 4087 µM; d 56: 24100 ± 3490 µM), and Cholesteryl 1-g-linolenoate (ce(18:3); d 0: 2593 ± 326 µM; d 56: 2887 ± 385 µM), and the less abundant were Cholesteryl docosadienoate (ce(22:2); day 0: 0.34 ± 0.04 µM; d 56: 0.38 ± 0.04 µM), and Cholesteryl behenate (ce(22:0); d 0: 0.07 ± 0.03 µM; d 56: 0.07 ± 0.03 µM). Neither group was statistically different at both d 0 and 56.

*Cresols:* the only evaluated compound was p-Cresol sulfate, whose plasma concentration is presented in Supplementary Table S12: (p-Cresol-SO4; d 0: 48.21 ± 3.86 µM; d 56: 45.15 ± 4.25 µM). No statistical differences were observed at both d 0 and 56.

*Diacylglycerols:* plasma concentration of 41 diacylglycerols are presented in Supplementary Table S13. All the evaluated diacylglycerols were detected in plasma samples and the most abundant were Diacylglycerol(28:0) (DG(14:0_14:0); d 0: 5.87 ± 0.81 µM; d 56: 6.28 ± 0.76 µM) and Diacylglycerol(32:2) (DG(14:0_18:2); d 0: 3.83 ± 0.5 µM; d 56: 4.02 ± 0.51 µM), and the less abundant were Diacylglycerol(36:0) (DG(16:0_20:0); d 0: 0.003 ± 0.00 µM; d 56: 0.003 ± 0.00 µM) and DG-O(14:0_18:2) (d 0: 0.001 ± 0.00 µM; d 56: 0.001 ± 0.00 µM). No statistical differences were observed at both d 0 and 56.

*Dihydroceramides*: plasma concentration of 8 dihydroceramides are presented in Supplementary Table S14. All the evaluated dihydroceramides were detected in plasma samples and the most abundant were C16-Dihydroceramide (Cer(d18:0/16:0); d 0: 83.46 ± 4.73 µM; d 56: 66.18 ± 5.02 µM) and Cer(d18:0/26:1(OH)) (d 0: 1.55 ± 0.11 µM; d 56: 1.55 ± 0.11 µM), and the less abundant were N-Icosanoylsphinganine (Cer(d18:0/20:0); d 0: 0.01 ± 0.00 µM; d 56: 0.02 ± 0.01 µM) and C18-(Dihydro)ceramide (Cer(d18:0/18:0); d 0: 0.03 ± 0.01 µM; d 56: 0.04 ± 0.005 µM). At d 0, there were no differences between groups for Dihydroceramides concentration. While at d 56, N-Tetracosanoylsphinganine (Cer(d18:0/180(OH)) was greater for LRFI group (LRFI: 0.70 ± 0.04 µM/ml; HRFI: 0.56 ± 0.04 µM/ml; *P* = 0.02; Fig. 7).

*Fatty Acids*: plasma concentration of 12 fatty acids is presented in Supplementary Table S15. All the evaluated fatty acids were detected in plasma samples and the most abundant were Myristic acid (FA(14:0); d 0: 2049 ± 831 µM; d 56: 2517 ± 933 µM) and Dodecanoic acid (FA(12:0); d 0: 810. ± 302 µM; d 56: 988 ± 458 µM), and the less abundant were Eicosapentaenoic acid (EPA; d 0: 0.16 ± 0.02 µM; d 56: 0.13 ± 0.02 µM) and Dihomo-gamma-linolenic acid (FA (20:3); d 0: 0.20 ± 0.04 µM; d 56: 0.32 ± 0.05 µM). At d 0 neither group was statistically different. While at d 56 Myristic acid (FA(14:0); LRFI: 2517 ± 933 µM/ml; HRFI: 897 ± 461 µM/ml; *P* = 0.02; Fig. 7) was increased for LRFI group. When analyzing the significance of the covariate (d 0) on the concentration of all evaluated *Fatty Acids*, only FA(14:0) (*P* = 0.02) had a significant effect of the covariate suggesting that the initial metabolic state at d 0 influenced the response of this metabolite at d 56.

*Glycerophospholipids*: plasma concentration of 88 glycerophospholipids are presented in Supplementary Table S16. All the evaluated glycerophospholipids were detected in plasma samples and the most abundant were: Phosphatidylcholine diacyl C36:2 (PC aa C36:2; day 0: 300 ± 25.71 µM; d 56: 325 ± 27.71 µM), and Phosphatidylcholine diacyl C34:2 (PC aa C34:2; d 0: 295 ± 21 µM; d 56: 273 ± 20 µM), and the less abundant were Phosphatidylcholine diacyl C44:6 (PC ae 44:6; d 0: 0.02 ± 0.00 µM; d 56: 0.02 ± 0.00 µM), and Phosphatidylcholine diacyl C24:0 (PC aa 24:0; d 0: 0.03 ± 0.00 µM; d 56: 0.04 ± 0.00 µM). At d 0 Phosphatidylcholine diacyl C42:4 (PC ae C42:4; LRFI: 0.02 ± 0.00 µM/ml; HRFI: 0.03 ± 0.00 µM/ml; *P* = 0.003) and Phosphatidylcholine diacyl C42:0 (PC aa C42:0; LRFI: 0.08 ± 0.01 µM/ml; HRFI: 0.1 ± 0.01 µM/ml; *P* = 0.03) were decreased for LRFI group (Fig. 6). While at d 56 Phosphatidylcholine diacyl C38:0 (PC aa C38:0; LRFI: 0.92 ± 0.08 µM/ml; HRFI: 0.71 ± 0.07 µM/ml; *P* = 0.03; Fig. 7) was significantly increased, and Phosphatidylcholine diacyl C42:0 (PC ae C42:0; LRFI: 0.03 ± 0.00 µM/ml; HRFI: 0.04 ± 0.00 µM/ml; *P* = 0.02; Fig. 8) was decreased for LRFI group.

*Glycosylceramides:* plasma concentration of 34 glycosylceramides is presented in Supplementary Table S17. All the evaluated glycosylceramides were detected in plasma samples and the most abundant were GlcCer(d18:1/24:1(15Z)) (HexCer(d18:1/24:1); d 0: 0.84. ± 0.08 µM; d 56: 0.69 ± 0.06 µM) and GlcCer(d18:1/22:0) (HexCer(d18:1/22:0); d 0: 0.75 ± 0.05 µM; d 56: 0.62 ± 0.06 µM), and the less abundant were Trihexosylceramide (d18:1/20:0) (Hex3Cer(d18:1_20:0); d 0: 0.004 ± 0.00 µM; d 56: 0.004 ± 0.00 µM) and Galabiosylceramide (d18:1/26:0) (Hex2Cer(d18:1/26:0); d 0: 0.01 ± 0.00 µM; d 56: 0.01 ± 0.00 µM). No statistical differences between RFI groups were observed at both d 0 and 56.

*Hormones:* plasma concentration of 4 hormones are presented in Supplementary Table S18. All the evaluated hormones were detected in plasma samples and the most abundant were Cortisol (d 0: 0.06 ± 0.01 µM; d 56: 0.06 ± 0.01 µM) and Cortisone (d 0: 0.03 ± 0.00 µM; d 56: 0.03 ± 0.00 µM), and the less abundant was Dehydroepiandrosterone sulfate (DHEAS; d 0: 0.02 ± 0.00 µM; d 56: 0.02 ± 0.00 µM). No statistical differences between RFI groups were observed at both d 0 and 56.

*Indole derivates:* plasma concentration of 4 indole derivates are presented in Supplementary Table S19. All the evaluated indole derivates were detected in plasma samples and the most abundant were: Indole (d 0: 19.42 ± 2.60 µM; d 56: 12.22 ± 2.65 µM) and Indoxyl sulfate (Ind-SO4; d 0: 2.97 ± 0.18 µM; d 56: 2.59 ± 0.20 µM), and the less abundant were Indoleacetic acid (3-IAA; d 0: 0.29 ± 0.02 µM; d 56: 0.29 ± 0.02 µM), and Indole-3-propionic acid (3-IPA; day 0: 0.49 ± 0.06 µM; d 56: 0.41 ± 0.06 µM). At d 0 Indole (LRFI: 19.42 ± 2.60 µM/ml; HRFI: 10.91 ± 3.04 µM/ml; *P* = 0.04; Fig. 5) was significantly increased for LRFI group. While no differences were observed at d 56.

*Nucleobases Related compounds:* plasma concentration of 2 nucleobases related compounds are presented in Supplementary Table S20. Both evaluated nucleobases related compounds were detected in plasma samples and their plasma concentration was not different at both d 0 and 56 when comparing LRFI and HRFI bulls.

*Sphingolipids:* plasma concentration of 14 sphingolipids are presented in Supplementary Table S21. All the evaluated sphingolipids were detected in plasma samples and the most abundant were Sphingomyelin (d18:1/16:0) (SM C16:0; d 0: 86.11 ± 3.30 µM; d 56: 91.34 ± 3.91 µM) and Sphingomyelin (D18:1/18:0) (SM C18:0; d 0: 12.87 ± 0.64 µM; d 56: 13.83 ± 0.82 µM), and the less abundant were Sphingomyelin (d18:1/26:1) (SM C26:1; d 0: 0.19 ± 0.03 µM; d 56: 0.26 ± 0.03 µM) and Sphingomyelin (d18:1/26:0) (SM C26:0; d 0: 0.24 ± 0.02 µM; d 56: 0.26 ± 0.02 µM). No statistical differences were observed at both d 0 and 56.

*Sugars:* the only evaluated compound was D-Glucose, whose plasma concentration is presented in Supplementary Table S22: (H1; d 0: 2500 ± 296.52 µM; d 56: 2349 ± 336 µM). No statistical differences were observed at both d 0 and 56.

*Triacylglycerols*: plasma concentration of 242 triacylglycerols are presented in Supplementary Table S23. All the evaluated triacylglycerols were detected in plasma samples and the most abundant were: Tracylglycerol(52:2) (TG(18:1_34:1); d 0: 29.14 ± 4.40 µM; d 56: 33.21 ± 15.15 µM) and Tracylglycerol(56:6) (TG(18:1_38:5); d 0: 27.71 ± 3.62 µM; d 56: 25.89 ± 2.79 µM), and the less abundant were TG(20:1_26:1) (d 0: 0.001 ± 0.00 µM; d 56: 0.001 ± 0.00 µM) and Tracylglycerol(54:6) (TG(22:1_32:5); d 0: 0.002 ± 0.00 µM; d 56: 0.003 ± 0.00 µM). At d 0 Tracylglycerol(54:6) (TG(20:2_34:4); LRFI: 0.05 ± 0.01 µM/ml; HRFI: 0.01 ± 0.01 µM/ml; *P* = 0.01; Fig. 5) was increased and Tracylglycerol(48:3) (TG(18:3_30:0); LRFI: 0.22 ± 0.02 µM/ml; HRFI: 0.34 ± 0.03 µM/ml; *P* = 0.01; Fig. 6) was decreased for LRFI group. While at d 56, Tracylglycerol(48:1) (TG(18:1_30:0); LRFI: 2.23 ± 0.32 µM/ml; HRFI: 3.32 ± 0.36 µM/ml; *P* = 0.02), Tracylglycerol(54:4) (TG(20:2_34:2); LRFI: 0.12 ± 0.04 µM/ml; HRFI: 0.41 ± 0.10 µM/ml; *P* = 0.01), Tracylglycerol(56:7) (TG(20:4_36:3); LRFI: 0.85 ± 0.11 µM/ml; HRFI: 1.26 ± 0.14 µM/ml; *P* = 0.03) and Tracylglycerol(56:8) (TG(20:5_36:3); LRFI: 0.27 ± 0.03 µM/ml; HRFI: 0.42 ± 0.05 µM/ml; *P* = 0.01) where decreased for LRFI group (Fig. 8). When analyzing the significance of the covariate (d 0) on the concentration of all evaluated Triacylglycerols, TG(20:2_34:2) (*P* = 0.012), TG(20:4_36:3) (*P* = 0.033) and TG(20:5_36:3) (*P* = 0.01) presented a significant effect of the covariate on d 0 suggesting that the initial metabolic state on d 0 influenced the response of these metabolites at d 56.

*Vitamins and Cofactors:* the only evaluated compound was Choline, whose plasma concentration is presented in Supplementary Table S24: (Choline; d 0: 9.62 ± 0.42 µM; d 56: 9.69 ± 0.39 µM). At d 0 Choline (LRFI: 9.62 ± 0.42 nM/ml; HRFI: 7.96 ± 0.38 µM/ml; *P* = 0.003) was increased for LRFI group, and no differences were observed at d 56 (Fig. 5).

*Hormones and isotopes:* Finally, no significant difference (*P* > .05) was found between RFI groups for hormone concentration (Cortisol and Testosterone) evaluated by Immunoassay test at both d 0 and 56 (Supplementary Table S25). Notably, cortisol concentration was also evaluated by the metabolomic platform (*Hormones* section) and, no statistical differences were detected among groups. In addition, no significant difference (*P* > .05) was observed in the variation of the isotope’s abundance (^15^N and ^1^³C) between groups on both d 0 and 56 (Supplementary Table S26).

### Biomarker analysis

**Figure 9:**
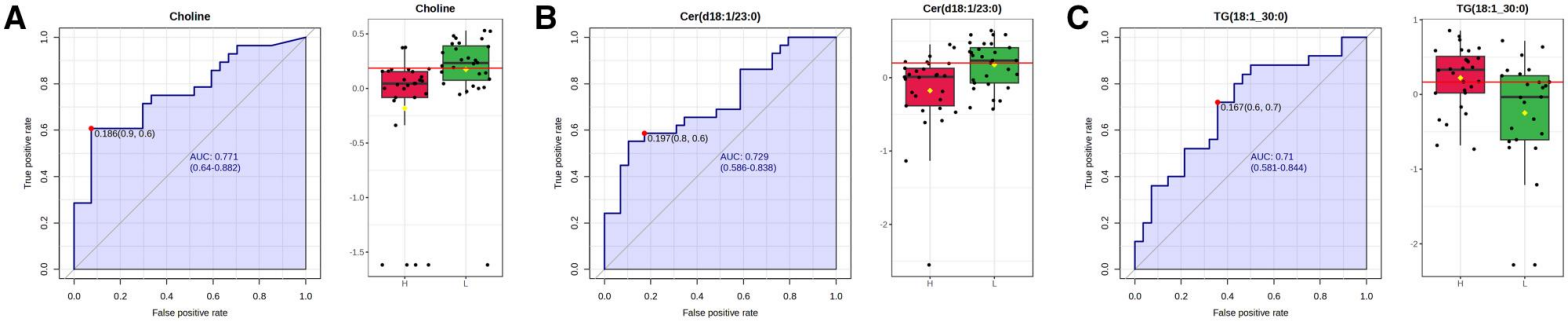
ROC Curve analysis was performed for each potential biomarker candidates for LRFI (green) and HRFI (red) groups. ROC analysis (left panel) and box-whisker plot (right panel) for (a) choline at d 0, (b) Cer(d18:1/23:0) at d 56, and TG(18:1_30:0) at d 56. The box-whisker plots revealed that choline and cer(d18:1/23:0) were increased, while TG(18:1_30:0) was decreased in LRFI animals (*P* < .05).

To evaluate the utility of the individual compound concentration as biomarkers of RFI, we performed ROC curve analysis of the most important metabolites identified by multivariate and univariate analysis. With an AUC value > 0.7 and p-value < 0.05 as the criteria for diagnostic potential, one metabolite, choline, was identified as biomarker of RFI on d 0, and two metabolites, Cer(d18:1/23:0) and TG(18:1_30:0), were identified as biomarkers of RFI on d 56. The individual ROC curve analysis results are presented with the cut-off point, AUC with 95% CI, sensitivity, and specificity values that show how effectively the selected candidate biomarkers can discriminate between the two RFI groups (Fig. 9).

## Discussion

The present study shows that the plasma concentration of certain metabolites differ when comparing bulls with divergent RFI statuses at the beginning and the end of the FE test. This suggests that some metabolites could have the potential to identify feed-efficient (LRFI) and non-efficient (HRFI) bulls and to develop practical tools for early phenotyping on commercial farms (Aboshady et al. 2024). However, the low predictive power of the multivariate models indicates that additional variables and alternative approaches should be explored to improve model performance. According to the sPLS-DA, acceptable coefficients of determination (*R*^2^) were obtained for the first and second components at both d 0 and 56 models, but the predictive ability was poor (*Q*^2^ < 0.05), far below the theoretical maximum of 1 or the empirically acceptable threshold of > 0.4 for biological models (Westerhuis et al. 2008). These findings align with the results of an experiment by Meale et al. (2017), who explored potential biomarkers of RFI and feed conversion efficiency in easily obtainable samples such as blood, hair, and feces in growing Charolais bulls, and found that the predictive models for both traits had poor accuracy (*Q*^2^ < 0.4).

Similarly, Novais et al. (2019) investigated the relationship between serum metabolite levels 21 days before the FE tests and RFI in Nellore bulls. Thay used untargeted metabolomicsand found that their proposed model was overfitted and not predictive (*Q*^2^ of 0.08 and 0.15 for metabolites with negative and positive mode, respectively). While Clemmons et al. (2017) used an untargeted metabolomics approach to determine differences in serum metabolites between purebred Angus steers of low and high RFI during a backgrounding phase, achieving moderate predictive value in the PLS-DA model (*Q*^2^ = 0.41), suggesting that certain metabolites were associated with differing RFI. However, the observed differences in the metabolite abundance may not be generalizable to a broader cattle population, particularly considering breeding variances, the range of RFI among animals (Cantalapiedra-Hijar et al. 2024) and the diet(Aboshady et al. 2024). This underscores the need for caution in interpreting the results. Although some studies have reported association between metabolites and FE, further research is required to validate these findings and develop a reliable plasma metabolic signature for RFI in beef cattle.

Investigating the complex metabolic mechanisms driving RFI in livestock reveals a multifaceted landscape, highlighting both conserved and species-specific pathways emerging as key determinants of FE. Numerous studies have delved into the metabolic underpinnings of RFI in livestock. However, there is still a high variability among the studies where different genes or compounds may affect the RFI status. An experiment performed by Yang et al. (2022), evaluated gene expression in liver samples in Charolais cattle fed a high-concentrate diet with divergent RFI. They found that LRFI cattle had enrichment of pathways related to lipid metabolism, such as fatty acids degradation, BAs biosynthesis, and pentose phosphate pathway. Additionally, pathways related to glucose and lipid metabolism, including tyrosine metabolism, steroid biosynthesis and glycolysis/gluconeogenesis and unsaturated fatty acids biosynthesis, were also enriched in LRFI cattle (Yang et al. 2022). In contrast, other studies had identified amino acid metabolism as the most important pathway to discriminate animals with divergent RFI (Taiwo et al. 2022) and FE (Nunes et al. 2024). Crossbreed steers selected according to their RFI status and fed a high-forage diet showed enrichment of amino acid metabolism in both whole blood transcriptome analysis (Taiwo et al. 2022) and plasma metabolomics (Taiwo et al. 2024). However, their validity as biomarkers is conditional rather than universal. Amino acid profiles are influenced by physiological stage, adiposity, diet characteristics, and metabolic state, indicating that amino acids are context-dependent biomarkers instead of stable indicators across production stages or diets (Aboshady et al. 2024). In our experiment, a high-concentrate diet was utilized, which may explain the absence of a significant abundance of amino acids-related pathways as observed in the previous studies conducted with high-forage diets, coinciding with the findings of Guarnido-Lopez et al. (2022), who reported lower protein turnover when animals were fed corn silage-based diets, supporting the concept of some diet-specific metabolic pathways underlying RFI differences (Jorge-Smeding et al. 2021). These contrasting metabolic networks influencing RFI status illustrate the sophisticated and interconnected roles of an animals’ individual biology, diet, and the wide range of strategies to maximize nutrient efficiency.

Primary BAs biosynthesis (day 0) and Sphingolipid metabolism (day 56) were two pathways enriched and with significant impact scores in plasma samples in LRFI bulls. Primary BAs are synthesized in the liver from cholesterol (Chiang 2004) and play a crucial role in lipid digestion and absorption. Beyond their digestive function, BAs function as signaling molecules, influencing various metabolic processes, including energy homeostasis, glucose metabolism, and lipid metabolism (Vítek and Haluzík 2016). While Sphingolipids participate in cell signaling and membrane structure by the generation of intermediates that regulate diverse biological functions such as cell growth, differentiation, and apoptosis (McFadden and Rico 2019). The differential regulation of metabolic pathways between LRFI and HRFI bulls suggests a complex interplay of factors influencing bovine physiology. BAs emerge as key regulators of glucose and lipid metabolism. By modulating glycogen synthesis, inhibiting gluconeogenesis, and influencing cholesterol homeostasis, BAs play a central role in energy balance (Chiang 2013; Gómez et al. 2022). Furthermore, alterations in the BAs pathway occur due to the influence on lipid and glucose metabolism, driven by the need to maintain metabolic homeostasis (Chiang 2013). Understanding the intricate mechanisms underlying these metabolic differences provides valuable insights into bovine physiology and potential targets for future research and breeding strategies.

According to univariate analysis, choline emerged as a key metabolite associated with RFI in our study. It was consistently identified in both univariate and multivariate analysis, with higher plasma concentrations in LRFI bulls at d 0. Choline’s multifaceted roles in animal physiology are well-documented. It serves as a precursor for cell membrane lipids (Gregg et al. 2023) and aids in lipid transport in the blood (Jayaprakash et al. 2016). By playing an important role in the metabolism of fat, it breaks fat down for use as an energy source, reducing the risk of fatty liver (Elsawy et al. 2014). Additionally, choline is a precursor of phosphatidylcholine, which has been associated with lower oxidative stress in the liver and linked to reduced maintenance requirements in LRFI steers, due to a lower protein and lipid turnover and more efficient energy use (Aboshady et al. 2024). Moreover, as a methyl donor, choline participates in DNA methylation process through the 1 carbon cycle metabolism (Holdorf et al. 2023). These diverse functions position choline as a potential hub metabolite linking various aspects of feed efficiency and RFI (Gregg et al. 2023). However, the relationship between choline and feed efficiency is not straightforward. In contrast to our results, Martin et al. (2021) reported lower plasmatic concentration of choline in efficient mid-lactation multiparous dairy cows, suggesting a potential differential metabolism of choline between animals with contrasting feed efficiency. Additionally, Li et al. (2021) identified a positive association between plasma choline concentration and daily DMI in crossbred beef cattle, indicating a potential role of choline in regulating feed intake and overall feed efficiency. Intriguingly, evidence from human and rodent studies suggests that choline may influence molecules that affect satiety signals in the hypothalamus (Brown et al. 2023), leading to decreased consumption of calories (Elsawy et al. 2014). This conflicting finding highlights the complex nature of choline’s influence on RFI and the need for further research to elucidate the underlying mechanisms.

The concentration of triglyceride also presented significative differences when comparing LRFI with HRFI bulls. On d 0, LRFI bulls showed increased concentration of TG(20:2_34:4), and a decreased concentration of TG(18:3_30:0). While by d 56, LRFI bulls exhibited a decrease concentration of TG(18:1_30:0), TG(20:2_34:2), TG(20:4_36:3), TG(20:5_36:3). Triglycerides serve as an energy reserve stored within the adipocyte cells (Vernon and Houseknecht 2000). Muscle tissue can utilize plasma triglycerides as an extra muscular fuel source, following their transformation into non-esterified fatty acids (Hocquette et al. 1998). Richardson et al. (2004), who observed higher plasma triglycerides levels in LRFI Angus growing steers, suggested increased lipogenesis and a higher metabolic rate in muscle tissue in the more efficient animals. Similarly, Jorge-Smeding et al. (2021) found a negative association between total triglycerides and RFI when Charolais yearling bulls were fed a high-forage diet, and Souza et al. (2019) reported a tendency for more efficient Nellore lactating cows to have higher triglyceride concentrations. However, other researchers failed to consistently identify significant differences in triglyceride concentrations between HRFI and LRFI animals, when controlling variables such as parity groups in dairy cows (Martin et al. 2021). Given these contrasting results and the potential existence of a RFI x Diet interaction affecting triglyceride concentrations (Jorge-Smeding et al. 2021), it is uncertain whether these metabolites can be reliably used as predictors of FE.

Finally, Cer(d18:1/23:0) [known as Sphingomyelin SM(D18:1/23:0)] was also highlighted as key metabolite whose plasmatic concentration was enriched at d 0 and 56 for LRFI bulls and was detected by multivariate analysis as a VIP compound, due to its contribution in differing RFI groups. Furthermore, sphingolipid metabolism was pointed out by the pathway analysis, emphasizing its relevance at d 56. This sphingolipid is found in animal cell membranes, particularly within the myelin sheath. In the nucleus it is the primary phospholipid linked to chromatin, and in plasma, is abundant in lipoprotein fractions (Mughram et al. 2024). Additionally, Cer(d18:1/23:0) is important in ruminant erythrocytes, where replaces phosphatidylcholine, interacting with proteins and cholesterol, being in plasma membranes vital for transferrin internalization, facilitating iron uptake by cells (Christie and Han 2010). Widmann et al. (2015), highlighted the potential role of sphingomyelin in energy metabolism and feed efficiency after performing a network approach to detect genes or gene-gene interactions that are likely participating in the modulation of feed efficiency in cattle population generated by crossing dairy and beef cattle. Ceramides synthesis can be stimulated by an increase in the fatty acids level and facilitate transport across membranes, enhancing their swift conversion into acyl-coenzyme A (Summers et al. 2019). Furthermore, they promote the incorporation of fatty acids into triglycerides, enabling their storage in lipid droplets and inhibiting the uptake of glucose and amino acids, thus leading to preferential utilization of fatty acids for energy. These observations underscore the significance of ceramides as regulators of glucose homeostasis, as confirmed by previous research (Summers et al. 2019). Owing to its role in the energetic metabolism, Sphingomyelin, and some precursors such as Ceramides, emerge as crucial players in the context of the anabolic efficiency that characterizes LRFI animals, becoming potential biomarkers to prematurely identify them.

The identification of different biomarkers at d 0 and d 56 is biologically expected because metabolite profiles naturally change as animals progress through different stages of growth and development. Shifts in age, diet, metabolic demands, and tissue accretion modify circulating metabolite concentrations and pathway activity, generating time-specific metabolic signatures. Previous studies in cattle and other species have shown that many metabolites are highly sensitive to developmental stage, whereas only a small subset remains stable over time (Karisa et al. 2014). Therefore, the distinct pathways identified at each sampling point likely reflect normal metabolic adjustments occurring as animals grow, rather than inconsistencies or limitations in the analytical approach. Most of the metabolites that exhibited significantly higher concentrations at d 0 and d 56 were associated with lipid transport, oxidation, and metabolism. Specifically, TMCA, diacylglycerols, and TAGs contributed to the pathways highlighted at d 0, whereas ceramides and phosphatidylcholine contributed to the pathways highlighted at d 56. These results align with prior research emphasizing the importance of lipid metabolism, oxidation, and transport in determining feed efficiency in livestock (Hayes et al. 2013; Artegoitia et al. 2019; Su et al. 2022). Collectively, these findings suggest that LRFI animals possess enhanced metabolic capacity, enabling them to harness energy and nutrients more efficiently from the ingested feed.

## Conclusions

We concluded that the multivariate model constructed using plasma metabolomics had limited predictive ability for residual feed intake (RFI) when using plasma samples collected at both the beginning (d 0) and the end (d 56) of the test. However, several individual metabolites differed significantly between LRFI and HRFI bulls at both time points. Notably, ceramides, particularly Cer(d18:1/23:0), emerged as potential contributors to physiological mechanisms that enhance energy utilization and may therefore be linked to improved feed efficiency. Additionally, plasma choline concentrations were higher in LRFI bulls and showed promising predictive value. Because these findings contrast with some previously published studies, further research is warranted to clarify the biological roles of choline, ceramides, and other metabolites in shaping the RFI phenotype.

## Supplementary Material

txag020_Supplementary_Data

## Data Availability

Not applicable.

## List of abbreviations

ADF: acid detergent fiber
ADG: average daily gain
AUC: Area Under the Curve
BA: Bile Acid
BRI: Beaumont Research Institute
BW: body weight
CI: confidence interval
Cov: covariate
CP: crude protein
CV: coefficient of variation
δ: isotope ratio
DM: dry matter
DMI: dry matter intake
ESI: electrospray ionization
FCR: feed conversion ratio
FDR: False Discovery Rate
FE: feed efficiency
FI: feed intake
GCBD: general complete block design
HRFI: high RFI
IACUC: Institutional Animal Care and Use Committee
IRMS: isotope ratio mass spectrometer
LRFI: low RFI
µM: micromolar
MBW: mid-metabolic BW
Mcal: megacalories
MW: metabolic weight
N: nitrogen
NDF: neutral detergent fiber
NFREC: North Florida Research and Education Center
P: phosphorus
PC: principal component
PCA: Principal Component Analysis
PDB: Pee Dee Belemnite
PLS-DA: Partial Least Square—Discriminant Analysis
Q²: cross-validated Q-squared
R²: coefficient of determination
RBC: red blood cells
RFI: Residual Feed Intake
ROC: Receiver Operating Characteristic
S: sulfur
sPLSDA: Sparse Partial Least Squares–Discriminant Analysis
TCA: Citrate cycle
TMR: total mixed ration
UF: University of Florida
WDA: weight per day of age
WG: weight gain
VIP: variable importance of projection
