# Supplementary material for: Plasma biomarkers for residual feed intake prediction in beef bulls
Source: Transl Anim Sci. 2026 Feb 23;10:txag020. doi: 10.1093/tas/txag020 (PMC12986784; doi:10.1093/tas/txag020)
Supplement: txag020_Supplementary_Data [file txag020_supplementary_data.zip › ST2 to ST26.docx]

**Supplementary Table S2.**Biochemical abbreviation and Plasma concentration (µM) of Acylcarnitines in low (LRFI) and high (HRFI) Residual Feed Intake bulls on Day 0 and 56 of the feed efficiency trial (Mean ± SEM).

|  |  |  | **Day 0** |  |  |  |  |  | **Day 56** |  |  |
| --- | --- | --- | --- | --- | --- | --- | --- | --- | --- | --- | --- |
|  |  | **LRFI** |  | **HRFI** |  |  |  | **LRFI** |  | **HRFI** |  |
| **COMPOUND** | **Mean** | **SEM** | **Mean** | **SEM** | ***P*** |  | **Mean** | **SEM** | **Mean** | **SEM** | ***P*** |
| **C0** | 12.92 | 0.34 | 13.14 | 0.54 | > 0.05 |  | 12.64 | 1.12 | 14.20 | 0.79 | > 0.05 |
| **C2** | 7.85 | 2.89 | 4.35 | 0.29 | > 0.05 |  | 6.30 | 1.01 | 6.41 | 0.99 | > 0.05 |
| **C3** | 0.26 | 0.03 | 0.32 | 0.03 | > 0.05 |  | 0.23 | 0.04 | 0.29 | 0.03 | > 0.05 |
| **C3-DC (C4-OH)** | 0.05 | 0.00 | 0.08 | 0.01 | 0.004 |  | 0.08 | 0.01 | 0.08 | 0.01 | > 0.05 |
| **C3-OH** | 0.27 | 0.02 | 0.26 | 0.02 | > 0.05 |  | 0.26 | 0.04 | 0.34 | 0.04 | > 0.05 |
| **C3:1** | 0.02 | 0.00 | 0.02 | 0.00 | > 0.05 |  | 0.04 | 0.00 | 0.04 | 0.01 | > 0.05 |
| **C4** | 0.26 | 0.02 | 0.28 | 0.02 | > 0.05 |  | 0.24 | 0.03 | 0.28 | 0.02 | > 0.05 |
| **C4:1** | 0.01 | 0.00 | 0.01 | 0.00 | > 0.05 |  | 0.04 | 0.01 | 0.04 | 0.01 | > 0.05 |
| **C5** | 0.13 | 0.01 | 0.16 | 0.01 | > 0.05 |  | 0.10 | 0.01 | 0.09 | 0.01 | > 0.05 |
| **C5-DC (C6-OH)** | 0.02 | 0.00 | 0.02 | 0.00 | > 0.05 |  | 0.01 | 0.00 | 0.01 | 0.00 | > 0.05 |
| **C5-M-DC** | 0.04 | 0.00 | 0.04 | 0.00 | > 0.05 |  | 0.07 | 0.01 | 0.07 | 0.00 | > 0.05 |
| **C5-OH (C3-DC-M)** | 0.06 | 0.00 | 0.06 | 0.00 | > 0.05 |  | 0.06 | 0.00 | 0.06 | 0.00 | > 0.05 |
| **C5:1** | 0.04 | 0.00 | 0.05 | 0.00 | > 0.05 |  | 0.09 | 0.01 | 0.08 | 0.01 | > 0.05 |
| **C5:1-DC** | 0.01 | 0.01 | 0.02 | 0.01 | > 0.05 |  | 0.11 | 0.01 | 0.12 | 0.02 | > 0.05 |
| **C6 (C4:1-DC)** | 0.04 | 0.00 | 0.04 | 0.00 | > 0.05 |  | 0.09 | 0.01 | 0.09 | 0.01 | > 0.05 |
| **C6:1** | 0.02 | 0.00 | 0.02 | 0.00 | > 0.05 |  | 0.01 | 0.00 | 0.01 | 0.00 | > 0.05 |
| **C7-DC** | 0.00 | 0.00 | 0.00 | 0.00 | > 0.05 |  | 0.00 | 0.00 | 0.00 | 0.00 | > 0.05 |
| **C8** | 0.01 | 0.00 | 0.01 | 0.00 | > 0.05 |  | 0.02 | 0.00 | 0.02 | 0.00 | > 0.05 |
| **C9** | 0.01 | 0.00 | 0.01 | 0.00 | > 0.05 |  | 0.03 | 0.00 | 0.02 | 0.00 | > 0.05 |
| **C10** | 0.04 | 0.00 | 0.04 | 0.00 | > 0.05 |  | 0.05 | 0.00 | 0.05 | 0.00 | > 0.05 |
| **C10:1** | 0.04 | 0.00 | 0.03 | 0.00 | > 0.05 |  | 0.03 | 0.00 | 0.03 | 0.00 | > 0.05 |
| **C10:2** | 0.08 | 0.00 | 0.08 | 0.00 | > 0.05 |  | 0.06 | 0.00 | 0.06 | 0.00 | > 0.05 |
| **C12** | 0.03 | 0.00 | 0.03 | 0.00 | > 0.05 |  | 0.04 | 0.00 | 0.04 | 0.00 | > 0.05 |
| **C12-DC** | 0.64 | 0.02 | 0.65 | 0.02 | > 0.05 |  | 0.73 | 0.03 | 0.73 | 0.02 | > 0.05 |
| **C12:1** | 0.02 | 0.00 | 0.02 | 0.00 | > 0.05 |  | 0.02 | 0.00 | 0.02 | 0.00 | > 0.05 |
| **C14** | 0.02 | 0.00 | 0.02 | 0.00 | > 0.05 |  | 0.03 | 0.00 | 0.03 | 0.00 | > 0.05 |
| **C14:1** | 0.02 | 0.00 | 0.02 | 0.00 | > 0.05 |  | 0.02 | 0.00 | 0.02 | 0.00 | > 0.05 |
| **C14:1-OH** | 0.01 | 0.00 | 0.01 | 0.00 | > 0.05 |  | 0.01 | 0.00 | 0.01 | 0.00 | > 0.05 |
| **C14:2** | 0.01 | 0.00 | 0.01 | 0.00 | > 0.05 |  | 0.01 | 0.00 | 0.01 | 0.00 | > 0.05 |
| **C14:2-OH** | 0.01 | 0.00 | 0.01 | 0.00 | > 0.05 |  | 0.01 | 0.00 | 0.01 | 0.00 | > 0.05 |
| **C16** | 0.01 | 0.00 | 0.01 | 0.00 | > 0.05 |  | 0.02 | 0.00 | 0.02 | 0.00 | > 0.05 |
| **C16-OH** | 0.01 | 0.00 | 0.01 | 0.00 | > 0.05 |  | 0.01 | 0.00 | 0.01 | 0.00 | > 0.05 |
| **C16:1** | 0.01 | 0.00 | 0.01 | 0.00 | > 0.05 |  | 0.01 | 0.00 | 0.01 | 0.00 | > 0.05 |
| **C16:1-OH** | 0.01 | 0.00 | 0.01 | 0.00 | > 0.05 |  | 0.01 | 0.00 | 0.01 | 0.00 | > 0.05 |
| **C16:2** | 0.00 | 0.00 | 0.00 | 0.00 | 0.04 |  | 0.01 | 0.00 | 0.00 | 0.00 | > 0.05 |
| **C16:2-OH** | 0.01 | 0.00 | 0.01 | 0.00 | > 0.05 |  | 0.01 | 0.00 | 0.01 | 0.00 | > 0.05 |
| **C18** | 0.03 | 0.00 | 0.02 | 0.00 | > 0.05 |  | 0.03 | 0.00 | 0.03 | 0.00 | > 0.05 |
| **C18:1** | 0.03 | 0.00 | 0.03 | 0.00 | > 0.05 |  | 0.03 | 0.00 | 0.03 | 0.00 | > 0.05 |
| **C18:1-OH** | 0.04 | 0.00 | 0.04 | 0.00 | > 0.05 |  | 0.02 | 0.00 | 0.02 | 0.00 | > 0.05 |
| **C18:2** | 0.00 | 0.00 | 0.00 | 0.00 | > 0.05 |  | 0.00 | 0.00 | 0.00 | 0.00 | > 0.05 |

**Supplementary Table S3.**Biochemical abbreviation and Plasma concentration (µM) of Alkaloids in low (LRFI) and high (HRFI) Residual Feed Intake bulls on Day 0 and 56 of the feed efficiency trial (Mean ± SEM).

|  |  |  | **Day 0** |  | |  | |  | |  | |  | | **Day 56** | |  | |  | |
| --- | --- | --- | --- | --- | --- | --- | --- | --- | --- | --- | --- | --- | --- | --- | --- | --- | --- | --- | --- |
|  |  | **LRFI** |  | **HRFI** | |  | |  | |  | | **LRFI** | |  | | **HRFI** | |  | |
| **COMPOUND** | **Mean** | **SEM** | **Mean** | **SEM** | | ***P*** | |  | | **Mean** | | **SEM** | | **Mean** | | **SEM** | | ***P*** | |
| **Trigonelline** | 0.05 | 0.01 | 0.07 | 0.02 | > 0.05 | |  | | 0.06 | | 0.01 | | 0.06 | | 0.01 | | > 0.05 | |  |

**Supplementary Table S4.**Biochemical abbreviation and Plasma concentration (µM) of Amine Oxides in low (LRFI) and high (HRFI) Residual Feed Intake bulls on Day 0 and 56 of the feed efficiency trial (Mean ± SEM).

|  |  |  | **Day 0** |  | |  | |  | |  | |  | | **Day 56** | |  | |  | |
| --- | --- | --- | --- | --- | --- | --- | --- | --- | --- | --- | --- | --- | --- | --- | --- | --- | --- | --- | --- |
|  |  | **LRFI** |  | **HRFI** | |  | |  | |  | | **LRFI** | |  | | **HRFI** | |  | |
| **COMPOUND** | **Mean** | **SEM** | **Mean** | **SEM** | | ***P*** | |  | | **Mean** | | **SEM** | | **Mean** | | **SEM** | | ***P*** | |
| **TMAO** | 3.03 | 0.68 | 2.95 | 0.48 | > 0.05 | |  | | 2.24 | | 0.47 | | 2.13 | | 0.50 | | > 0.05 | |  |

**Supplementary Table S5.**Biochemical abbreviation and Plasma concentration (µM) of Amino acids in low (LRFI) and high (HRFI) Residual Feed Intake bulls on Day 0 and 56 of the feed efficiency trial (Mean ± SEM).

|  |  |  | **Day 0** |  | |  | |  | |  | |  | | **Day 56** | |  | |  | |
| --- | --- | --- | --- | --- | --- | --- | --- | --- | --- | --- | --- | --- | --- | --- | --- | --- | --- | --- | --- |
|  |  | **LRFI** |  | **HRFI** | |  | |  | |  | | **LRFI** | |  | | **HRFI** | |  | |
| **COMPOUND** | **Mean** | **SEM** | **Mean** | **SEM** | | ***P*** | |  | | **Mean** | | **SEM** | | **Mean** | | **SEM** | | ***P*** | |
| **Ala** | 250.00 | 12.54 | 268.43 | 8.05 | > 0.05 | |  | | 251.04 | | 11.37 | | 254.48 | | 12.27 | | > 0.05 | |  |
| **Arg** | 96.37 | 2.96 | 101.24 | 3.01 | > 0.05 | |  | | 102.69 | | 3.06 | | 100.58 | | 3.26 | | > 0.05 | |  |
| **Asn** | 36.36 | 1.68 | 39.82 | 1.31 | > 0.05 | |  | | 39.60 | | 1.59 | | 39.63 | | 1.35 | | > 0.05 | |  |
| **Asp** | 9.53 | 1.47 | 11.11 | 0.87 | > 0.05 | |  | | 12.61 | | 1.35 | | 11.60 | | 1.60 | | > 0.05 | |  |
| **Cys** | 27.11 | 0.83 | 28.59 | 0.75 | > 0.05 | |  | | 27.23 | | 0.80 | | 27.43 | | 0.84 | | > 0.05 | |  |
| **Gln** | 367.52 | 8.84 | 373.63 | 8.85 | > 0.05 | |  | | 372.33 | | 8.94 | | 376.61 | | 10.00 | | > 0.05 | |  |
| **Glu** | 104.56 | 5.77 | 94.34 | 5.31 | > 0.05 | |  | | 92.30 | | 5.23 | | 90.19 | | 5.44 | | > 0.05 | |  |
| **Gly** | 413.96 | 16.51 | 449.43 | 16.98 | > 0.05 | |  | | 431.21 | | 15.06 | | 421.66 | | 16.81 | | > 0.05 | |  |
| **His** | 71.30 | 2.08 | 78.09 | 1.94 | 0.02 | |  | | 79.34 | | 2.41 | | 76.04 | | 3.12 | | > 0.05 | |  |
| **Ile** | 147.34 | 5.96 | 176.59 | 4.83 | 0.0003 | |  | | 157.25 | | 5.83 | | 168.08 | | 6.38 | | > 0.05 | |  |
| **Leu** | 219.79 | 9.29 | 246.00 | 7.61 | 0.03 | |  | | 231.17 | | 10.42 | | 231.37 | | 8.42 | | > 0.05 | |  |
| **Lys** | 103.42 | 3.83 | 111.22 | 3.44 | > 0.05 | |  | | 110.03 | | 4.88 | | 111.80 | | 3.55 | | > 0.05 | |  |
| **Met** | 29.68 | 1.34 | 32.33 | 1.34 | > 0.05 | |  | | 29.78 | | 1.24 | | 30.56 | | 1.04 | | > 0.05 | |  |
| **Phe** | 64.56 | 2.36 | 69.76 | 2.73 | > 0.05 | |  | | 67.83 | | 3.04 | | 73.28 | | 2.86 | | > 0.05 | |  |
| **Pro** | 87.06 | 2.71 | 89.82 | 2.25 | > 0.05 | |  | | 88.65 | | 2.63 | | 89.22 | | 2.57 | | > 0.05 | |  |
| **Ser** | 86.22 | 5.05 | 93.07 | 4.08 | > 0.05 | |  | | 87.54 | | 4.41 | | 87.08 | | 3.69 | | > 0.05 | |  |
| **Thr** | 82.23 | 3.52 | 91.24 | 3.32 | > 0.05 | |  | | 86.65 | | 3.38 | | 83.70 | | 3.54 | | > 0.05 | |  |
| **Trp** | 55.39 | 2.09 | 59.32 | 1.81 | > 0.05 | |  | | 59.83 | | 2.97 | | 54.91 | | 2.34 | | > 0.05 | |  |
| **Tyr** | 71.85 | 3.17 | 88.69 | 4.02 | 0.02 | |  | | 90.58 | | 4.70 | | 83.40 | | 4.35 | | > 0.05 | |  |
| **Val** | 236.87 | 12.37 | 269.46 | 14.22 | > 0.05 | |  | | 262.33 | | 13.16 | | 262.86 | | 15.16 | | > 0.05 | |  |

**Supplementary Table S6.**Biochemical abbreviation and Plasma concentration (µM) of Amino acids Related in low (LRFI) and high (HRFI) Residual Feed Intake bulls on Day 0 and 56 of the feed efficiency trial (Mean ± SEM).

|  |  |  | **Day 0** |  | |  | |  | |  | |  | | **Day 56** | |  | |  | |
| --- | --- | --- | --- | --- | --- | --- | --- | --- | --- | --- | --- | --- | --- | --- | --- | --- | --- | --- | --- |
|  |  | **LRFI** |  | **HRFI** | |  | |  | |  | | **LRFI** | |  | | **HRFI** | |  | |
| **COMPOUND** | **Mean** | **SEM** | **Mean** | **SEM** | | ***P*** | |  | | **Mean** | | **SEM** | | **Mean** | | **SEM** | | ***P*** | |
| **1-Met-His** | 4.98 | 0.27 | 4.59 | 0.25 | > 0.05 | |  | | 5.36 | | 0.32 | | 4.68 | | 0.24 | | > 0.05 | |  |
| **3-Met-His** | 4.28 | 0.20 | 4.06 | 0.17 | > 0.05 | |  | | 4.72 | | 0.20 | | 4.34 | | 0.19 | | > 0.05 | |  |
| **5-AVA** | 0.42 | 0.04 | 0.50 | 0.06 | > 0.05 | |  | | 0.45 | | 0.04 | | 0.47 | | 0.05 | | > 0.05 | |  |
| **AABA** | 6.27 | 0.35 | 6.45 | 0.42 | > 0.05 | |  | | 6.59 | | 0.48 | | 6.63 | | 0.42 | | > 0.05 | |  |
| **Ac-Orn** | 2.36 | 0.27 | 2.50 | 0.35 | > 0.05 | |  | | 2.06 | | 0.27 | | 2.40 | | 0.22 | | > 0.05 | |  |
| **ADMA** | 1.23 | 0.03 | 1.29 | 0.04 | > 0.05 | |  | | 1.34 | | 0.03 | | 1.24 | | 0.03 | | > 0.05 | |  |
| **alpha-AAA** | 1.47 | 0.08 | 1.35 | 0.06 | > 0.05 | |  | | 1.41 | | 0.07 | | 1.30 | | 0.05 | | > 0.05 | |  |
| **Anserine** | 0.36 | 0.02 | 0.33 | 0.02 | > 0.05 | |  | | 0.34 | | 0.01 | | 0.31 | | 0.02 | | > 0.05 | |  |
| **BABA** | 0.07 | 0.01 | 0.07 | 0.01 | > 0.05 | |  | | 0.07 | | 0.01 | | 0.07 | | 0.01 | | > 0.05 | |  |
| **Betaine** | 53.82 | 4.01 | 47.41 | 4.13 | > 0.05 | |  | | 58.25 | | 4.04 | | 56.66 | | 4.29 | | > 0.05 | |  |
| **c4-OH-Pro** | 0.01 | 0.00 | 0.02 | 0.00 | > 0.05 | |  | | 0.01 | | 0.00 | | 0.01 | | 0.00 | | > 0.05 | |  |
| **Carnosine** | 17.67 | 1.03 | 16.73 | 0.85 | > 0.05 | |  | | 16.46 | | 0.76 | | 15.02 | | 0.94 | | > 0.05 | |  |
| **Cit** | 65.39 | 3.13 | 69.19 | 4.33 | > 0.05 | |  | | 67.89 | | 4.06 | | 72.47 | | 4.52 | | > 0.05 | |  |
| **Creatinine** | 104.62 | 3.23 | 99.83 | 2.55 | > 0.05 | |  | | 108.22 | | 3.08 | | 97.05 | | 2.76 | | 0.01 | |  |
| **Cystine** | 13.38 | 0.76 | 15.12 | 0.84 | > 0.05 | |  | | 12.74 | | 0.83 | | 13.73 | | 0.66 | | > 0.05 | |  |
| **DOPA** | 0.02 | 0.00 | 0.02 | 0.00 | > 0.05 | |  | | 0.02 | | 0.00 | | 0.03 | | 0.00 | | > 0.05 | |  |
| **HArg** | 2.75 | 0.11 | 2.75 | 0.10 | > 0.05 | |  | | 2.71 | | 0.12 | | 2.86 | | 0.13 | | > 0.05 | |  |
| **HCys** | 3.45 | 0.14 | 3.89 | 0.17 | > 0.05 | |  | | 3.56 | | 0.18 | | 3.33 | | 0.16 | | > 0.05 | |  |
| **Kynurenine** | 4.92 | 0.24 | 5.13 | 0.24 | > 0.05 | |  | | 5.25 | | 0.31 | | 5.33 | | 0.17 | | > 0.05 | |  |
| **Met-SO** | 1.63 | 0.09 | 1.64 | 0.11 | > 0.05 | |  | | 1.55 | | 0.11 | | 1.72 | | 0.09 | | > 0.05 | |  |
| **Nitro-Tyr** | 0.46 | 0.02 | 0.44 | 0.02 | > 0.05 | |  | | 0.44 | | 0.02 | | 0.45 | | 0.02 | | > 0.05 | |  |
| **Orn** | 80.28 | 3.21 | 81.48 | 3.74 | > 0.05 | |  | | 82.96 | | 3.85 | | 84.95 | | 3.02 | | > 0.05 | |  |
| **PAG** | 4.36 | 0.42 | 4.86 | 0.54 | > 0.05 | |  | | 5.24 | | 0.53 | | 5.44 | | 0.68 | | > 0.05 | |  |
| **PheAlaBetaine** | 0.00 | 0.00 | 0.00 | 0.00 | > 0.05 | |  | | 0.00 | | 0.00 | | 0.00 | | 0.00 | | > 0.05 | |  |
| **ProBetaine** | 0.36 | 0.03 | 0.34 | 0.03 | > 0.05 | |  | | 0.42 | | 0.05 | | 0.40 | | 0.03 | | > 0.05 | |  |
| **Sarcosine** | 2.95 | 0.12 | 2.96 | 0.11 | > 0.05 | |  | | 3.07 | | 0.12 | | 3.19 | | 0.09 | | > 0.05 | |  |
| **SDMA** | 0.57 | 0.03 | 0.54 | 0.03 | > 0.05 | |  | | 0.58 | | 0.02 | | 0.55 | | 0.02 | | > 0.05 | |  |
| **t4-OH-Pro** | 31.85 | 1.39 | 27.86 | 1.09 | > 0.05 | |  | | 31.02 | | 1.10 | | 28.74 | | 1.30 | | > 0.05 | |  |
| **Taurine** | 32.55 | 2.47 | 35.16 | 2.59 | > 0.05 | |  | | 32.79 | | 2.16 | | 37.55 | | 2.54 | | > 0.05 | |  |
| **TrpBetaine** | 0.01 | 0.00 | 0.01 | 0.00 | > 0.05 | |  | | 0.01 | | 0.00 | | 0.01 | | 0.00 | | > 0.05 | |  |

**Supplementary Table S7.**Biochemical abbreviation and Plasma concentration (µM) of Bile Acids in low (LRFI) and high (HRFI) Residual Feed Intake bulls on Day 0 and 56 of the feed efficiency trial (Mean ± SEM).

|  |  |  | **Day 0** |  | |  | |  | |  | |  | | **Day 56** | |  | |  | |
| --- | --- | --- | --- | --- | --- | --- | --- | --- | --- | --- | --- | --- | --- | --- | --- | --- | --- | --- | --- |
|  |  | **LRFI** |  | **HRFI** | |  | |  | |  | | **LRFI** | |  | | **HRFI** | |  | |
| **COMPOUND** | **Mean** | **SEM** | **Mean** | **SEM** | | ***P*** | |  | | **Mean** | | **SEM** | | **Mean** | | **SEM** | | ***P*** | |
| **CA** | 8.73 | 1.13 | 9.04 | 0.99 | > 0.05 | |  | | 9.01 | | 1.11 | | 10.64 | | 0.97 | | > 0.05 | |  |
| **CDCA** | 0.73 | 0.14 | 1.01 | 0.23 | > 0.05 | |  | | 1.17 | | 0.26 | | 1.15 | | 0.18 | | > 0.05 | |  |
| **DCA** | 1.52 | 0.29 | 1.51 | 0.24 | > 0.05 | |  | | 1.94 | | 0.34 | | 1.99 | | 0.27 | | > 0.05 | |  |
| **GCA** | 5.40 | 0.67 | 3.84 | 0.62 | > 0.05 | |  | | 4.52 | | 0.76 | | 4.82 | | 0.76 | | > 0.05 | |  |
| **GCDCA** | 0.55 | 0.05 | 0.48 | 0.07 | > 0.05 | |  | | 0.61 | | 0.09 | | 0.54 | | 0.07 | | > 0.05 | |  |
| **GDCA** | 1.69 | 0.19 | 1.18 | 0.17 | 0.04 | |  | | 1.49 | | 0.22 | | 1.14 | | 0.14 | | > 0.05 | |  |
| **GLCA** | 0.11 | 0.01 | 0.08 | 0.01 | 0.03 | |  | | 0.10 | | 0.01 | | 0.09 | | 0.01 | | > 0.05 | |  |
| **GLCAS** | 0.00 | 0.00 | 0.00 | 0.00 | > 0.05 | |  | | 0.01 | | 0.00 | | 0.00 | | 0.00 | | > 0.05 | |  |
| **GUDCA** | 0.00 | 0.00 | 0.00 | 0.00 | > 0.05 | |  | | 0.00 | | 0.00 | | 0.00 | | 0.00 | | > 0.05 | |  |
| **TCA** | 1.24 | 0.22 | 0.78 | 0.13 | 0.04 | |  | | 0.96 | | 0.18 | | 0.85 | | 0.13 | | > 0.05 | |  |
| **TCDCA** | 0.21 | 0.02 | 0.25 | 0.04 | > 0.05 | |  | | 0.17 | | 0.02 | | 0.19 | | 0.03 | | > 0.05 | |  |
| **TDCA** | 0.33 | 0.05 | 0.23 | 0.04 | > 0.05 | |  | | 0.35 | | 0.05 | | 0.20 | | 0.02 | | 0.04 | |  |
| **TLCA** | 0.04 | 0.01 | 0.03 | 0.00 | > 0.05 | |  | | 0.04 | | 0.01 | | 0.03 | | 0.00 | | > 0.05 | |  |
| **TMCA** | 0.01 | 0.00 | 0.00 | 0.00 | 0.02 | |  | | 0.01 | | 0.00 | | 0.00 | | 0.00 | | > 0.05 | |  |

**Supplementary Table S8.**Biochemical abbreviation and Plasma concentration (µM) of Biogenic Amines in low (LRFI) and high (HRFI) Residual Feed Intake bulls on Day 0 and 56 of the feed efficiency trial (Mean ± SEM).

|  |  |  | **Day 0** |  | |  | |  | |  | |  | | **Day 56** | |  | |  | |
| --- | --- | --- | --- | --- | --- | --- | --- | --- | --- | --- | --- | --- | --- | --- | --- | --- | --- | --- | --- |
|  |  | **LRFI** |  | **HRFI** | |  | |  | |  | | **LRFI** | |  | | **HRFI** | |  | |
| **COMPOUND** | **Mean** | **SEM** | **Mean** | **SEM** | | ***P*** | |  | | **Mean** | | **SEM** | | **Mean** | | **SEM** | | ***P*** | |
| **beta-Ala** | 1.55 | 0.08 | 1.53 | 0.07 | > 0.05 | |  | | 1.58 | | 0.08 | | 1.73 | | 0.06 | | > 0.05 | |  |
| **Dopamine** | 0.09 | 0.02 | 0.10 | 0.02 | > 0.05 | |  | | 0.11 | | 0.02 | | 0.11 | | 0.02 | | > 0.05 | |  |
| **GABA** | 0.13 | 0.01 | 0.12 | 0.01 | > 0.05 | |  | | 0.13 | | 0.01 | | 0.12 | | 0.01 | | > 0.05 | |  |
| **Histamine** | 0.00 | 0.00 | 0.00 | 0.00 | > 0.05 | |  | | 0.00 | | 0.00 | | 0.00 | | 0.00 | | > 0.05 | |  |
| **PEA** | 0.00 | 0.00 | 0.00 | 0.00 | > 0.05 | |  | | 0.00 | | 0.00 | | 0.00 | | 0.00 | | > 0.05 | |  |
| **Putrescine** | 0.16 | 0.01 | 0.14 | 0.01 | > 0.05 | |  | | 0.14 | | 0.01 | | 0.13 | | 0.01 | | > 0.05 | |  |
| **Serotonin** | 0.13 | 0.01 | 0.14 | 0.01 | > 0.05 | |  | | 0.14 | | 0.01 | | 0.12 | | 0.01 | | > 0.05 | |  |
| **Spermidine** | 0.02 | 0.00 | 0.02 | 0.00 | > 0.05 | |  | | 0.02 | | 0.00 | | 0.03 | | 0.00 | | > 0.05 | |  |
| **Spermine** | 0.05 | 0.01 | 0.05 | 0.01 | > 0.05 | |  | | 0.06 | | 0.01 | | 0.06 | | 0.01 | | > 0.05 | |  |

**Supplementary Table S9.**Biochemical abbreviation and Plasma concentration (µM) of Carboxylic Acids in low (LRFI) and high (HRFI) Residual Feed Intake bulls on Day 0 and 56 of the feed efficiency trial (Mean ± SEM).

|  | |  | |  | | **Day 0** | |  | |  | |  | |  | |  | | **Day 56** | |  | |  |
| --- | --- | --- | --- | --- | --- | --- | --- | --- | --- | --- | --- | --- | --- | --- | --- | --- | --- | --- | --- | --- | --- | --- |
|  | |  | | **LRFI** | |  | | **HRFI** | |  | |  | |  | | **LRFI** | |  | | **HRFI** | |  |
| **COMPOUND** | | **Mean** | | **SEM** | | **Mean** | | **SEM** | | ***P*** | |  | | **Mean** | | **SEM** | | **Mean** | | **SEM** | | ***P*** |
| **AconAcid** | 15.24 | | 1.20 | | 12.90 | | 1.17 | | > 0.05 | |  | | 12.47 | | 1.19 | | 10.87 | | 0.89 | | > 0.05 | |
| **DiCA(12:0)** | 0.28 | | 0.01 | | 0.23 | | 0.02 | | 0.02 | |  | | 0.24 | | 0.02 | | 0.25 | | 0.02 | | > 0.05 | |
| **DiCA(14:0)** | 0.04 | | 0.01 | | 0.04 | | 0.01 | | > 0.05 | |  | | 0.03 | | 0.01 | | 0.04 | | 0.01 | | > 0.05 | |
| **HipAcid** | 54.02 | | 3.69 | | 45.72 | | 3.66 | | > 0.05 | |  | | 49.84 | | 3.93 | | 47.12 | | 3.16 | | > 0.05 | |
| **Lac** | 7319 | | 1025 | | 6255 | | 734 | | > 0.05 | |  | | 6240 | | 796 | | 5394 | | 681 | | > 0.05 | |
| **OH-GlutAcid** | 4.79 | | 0.35 | | 4.42 | | 0.34 | | > 0.05 | |  | | 4.71 | | 0.32 | | 3.84 | | 0.26 | | 0.01 | |
| **Suc** | 9.53 | | 1.22 | | 7.64 | | 0.90 | | > 0.05 | |  | | 7.88 | | 0.94 | | 8.35 | | 0.84 | | > 0.05 | |

**Supplementary Table S10.**Biochemical abbreviation and Plasma concentration (µM) of Ceramides in low (LRFI) and high (HRFI) Residual Feed Intake bulls on Day 0 and 56 of the feed efficiency trial (Mean ± SEM).

|  |  |  | **Day 0** |  | |  | |  | |  |  | | | **Day 56** |  | |  | |  |
| --- | --- | --- | --- | --- | --- | --- | --- | --- | --- | --- | --- | --- | --- | --- | --- | --- | --- | --- | --- |
|  |  | **LRFI** |  | **HRFI** | |  | |  | |  | **LRFI** | | |  | **HRFI** | |  | |  |
| **COMPOUND** | **Mean** | **SEM** | **Mean** | **SEM** | | ***P*** | |  | | **Mean** | **SEM** | | | **Mean** | **SEM** | | ***P*** | |  |
| **Cer(d16:1/18:0)** | 0.05 | 0.00 | 0.05 | 0.00 | > 0.05 | |  | | 0.05 | | | 0.00 | 0.05 | | | 0.00 | | > 0.05 | |
| **Cer(d16:1/20:0)** | 0.08 | 0.00 | 0.09 | 0.00 | > 0.05 | |  | | 0.09 | | | 0.00 | 0.08 | | | 0.00 | | > 0.05 | |
| **Cer(d16:1/22:0)** | 0.06 | 0.00 | 0.06 | 0.00 | > 0.05 | |  | | 0.06 | | | 0.00 | 0.05 | | | 0.00 | | > 0.05 | |
| **Cer(d16:1/23:0)** | 0.06 | 0.01 | 0.05 | 0.01 | > 0.05 | |  | | 0.06 | | | 0.00 | 0.05 | | | 0.00 | | > 0.05 | |
| **Cer(d16:1/24:0)** | 0.09 | 0.00 | 0.10 | 0.01 | > 0.05 | |  | | 0.10 | | | 0.01 | 0.10 | | | 0.00 | | > 0.05 | |
| **Cer(d18:1/14:0)** | 0.07 | 0.00 | 0.07 | 0.00 | > 0.05 | |  | | 0.07 | | | 0.00 | 0.07 | | | 0.00 | | > 0.05 | |
| **Cer(d18:1/16:0)** | 0.20 | 0.02 | 0.21 | 0.03 | > 0.05 | |  | | 0.21 | | | 0.02 | 0.21 | | | 0.02 | | > 0.05 | |
| **Cer(d18:1/18:0(OH))** | 0.43 | 0.02 | 0.45 | 0.02 | > 0.05 | |  | | 0.46 | | | 0.02 | 0.45 | | | 0.02 | | > 0.05 | |
| **Cer(d18:1/18:0)** | 0.06 | 0.01 | 0.06 | 0.01 | > 0.05 | |  | | 0.08 | | | 0.01 | 0.06 | | | 0.01 | | > 0.05 | |
| **Cer(d18:1/18:1)** | 0.03 | 0.00 | 0.03 | 0.00 | > 0.05 | |  | | 0.03 | | | 0.00 | 0.04 | | | 0.00 | | > 0.05 | |
| **Cer(d18:1/20:0(OH))** | 0.34 | 0.03 | 0.37 | 0.03 | > 0.05 | |  | | 0.36 | | | 0.03 | 0.36 | | | 0.04 | | > 0.05 | |
| **Cer(d18:1/20:0)** | 0.02 | 0.00 | 0.02 | 0.00 | > 0.05 | |  | | 0.02 | | | 0.00 | 0.02 | | | 0.00 | | > 0.05 | |
| **Cer(d18:1/22:0)** | 0.08 | 0.01 | 0.09 | 0.01 | > 0.05 | |  | | 0.09 | | | 0.01 | 0.08 | | | 0.01 | | > 0.05 | |
| **Cer(d18:1/23:0)** | 0.23 | 0.02 | 0.19 | 0.01 | 0.04 | |  | | 0.27 | | | 0.02 | 0.21 | | | 0.01 | | 0.02 | |
| **Cer(d18:1/24:0)** | 0.20 | 0.01 | 0.17 | 0.01 | > 0.05 | |  | | 0.21 | | | 0.01 | 0.18 | | | 0.01 | | > 0.05 | |
| **Cer(d18:1/24:1)** | 0.09 | 0.01 | 0.07 | 0.01 | > 0.05 | |  | | 0.11 | | | 0.01 | 0.09 | | | 0.01 | | > 0.05 | |
| **Cer(d18:1/25:0)** | 0.09 | 0.01 | 0.09 | 0.01 | > 0.05 | |  | | 0.09 | | | 0.01 | 0.08 | | | 0.01 | | > 0.05 | |
| **Cer(d18:1/26:0)** | 0.04 | 0.00 | 0.05 | 0.00 | > 0.05 | |  | | 0.05 | | | 0.00 | 0.04 | | | 0.00 | | > 0.05 | |
| **Cer(d18:1/26:1)** | 0.02 | 0.00 | 0.02 | 0.00 | > 0.05 | |  | | 0.02 | | | 0.00 | 0.02 | | | 0.00 | | > 0.05 | |
| **Cer(d18:2/14:0)** | 0.02 | 0.00 | 0.02 | 0.00 | > 0.05 | |  | | 0.02 | | | 0.00 | 0.02 | | | 0.00 | | > 0.05 | |
| **Cer(d18:2/16:0)** | 0.04 | 0.00 | 0.04 | 0.00 | > 0.05 | |  | | 0.04 | | | 0.01 | 0.04 | | | 0.00 | | > 0.05 | |
| **Cer(d18:2/18:0)** | 0.04 | 0.01 | 0.05 | 0.00 | > 0.05 | |  | | 0.04 | | | 0.00 | 0.04 | | | 0.00 | | > 0.05 | |
| **Cer(d18:2/18:1)** | 0.02 | 0.00 | 0.02 | 0.00 | > 0.05 | |  | | 0.02 | | | 0.00 | 0.02 | | | 0.00 | | > 0.05 | |
| **Cer(d18:2/20:0)** | 0.04 | 0.00 | 0.05 | 0.00 | > 0.05 | |  | | 0.04 | | | 0.00 | 0.04 | | | 0.00 | | > 0.05 | |
| **Cer(d18:2/22:0)** | 0.03 | 0.00 | 0.03 | 0.00 | > 0.05 | |  | | 0.03 | | | 0.00 | 0.02 | | | 0.00 | | > 0.05 | |
| **Cer(d18:2/23:0)** | 0.02 | 0.00 | 0.03 | 0.00 | > 0.05 | |  | | 0.03 | | | 0.00 | 0.03 | | | 0.00 | | > 0.05 | |
| **Cer(d18:2/24:0)** | 0.05 | 0.01 | 0.06 | 0.01 | > 0.05 | |  | | 0.05 | | | 0.01 | 0.05 | | | 0.01 | | > 0.05 | |
| **Cer(d18:2/24:1)** | 0.04 | 0.00 | 0.04 | 0.00 | > 0.05 | |  | | 0.04 | | | 0.00 | 0.04 | | | 0.00 | | > 0.05 | |

**Supplementary Table S11.**Biochemical abbreviation and Plasma concentration (µM) of Cholesterol Esters in low (LRFI) and high (HRFI) Residual Feed Intake bulls on Day 0 and 56 of the feed efficiency trial (Mean ± SEM).

|  | |  |  | | **Day 0** | |  | |  | |  | |  |  | | **Day 56** | |  | |  | |  |
| --- | --- | --- | --- | --- | --- | --- | --- | --- | --- | --- | --- | --- | --- | --- | --- | --- | --- | --- | --- | --- | --- | --- |
|  | |  | **LRFI** | |  | | **HRFI** | |  | |  | |  | **LRFI** | |  | | **HRFI** | |  | |  |
| **COMPOUND** | | **Mean** | **SEM** | | **Mean** | | **SEM** | | ***P*** | |  | | **Mean** | **SEM** | | **Mean** | | **SEM** | | ***P*** | |  |
| **CE(14:0)** | 67.56 | | | 8.59 | | 62.64 | | 7.60 | | > 0.05 | |  | 69.03 | | 8.89 | | 73.12 | | 8.57 | | > 0.05 | |
| **CE(14:1)** | 0.93 | | | 0.12 | | 0.94 | | 0.12 | | > 0.05 | |  | 1.02 | | 0.12 | | 1.06 | | 0.13 | | > 0.05 | |
| **CE(15:0)** | 6.20 | | | 0.85 | | 6.39 | | 0.80 | | > 0.05 | |  | 6.39 | | 0.74 | | 6.36 | | 0.73 | | > 0.05 | |
| **CE(15:1)** | 1.28 | | | 0.19 | | 1.29 | | 0.18 | | > 0.05 | |  | 1.24 | | 0.16 | | 1.47 | | 0.21 | | > 0.05 | |
| **CE(16:0)** | 405.76 | | | 54.15 | | 440.00 | | 55.44 | | > 0.05 | |  | 439.37 | | 47.35 | | 443.22 | | 58.72 | | > 0.05 | |
| **CE(16:1)** | 224.78 | | | 30.08 | | 243.91 | | 30.19 | | > 0.05 | |  | 256.41 | | 30.91 | | 271.60 | | 34.83 | | > 0.05 | |
| **CE(17:0)** | 56.36 | | | 8.58 | | 52.26 | | 7.66 | | > 0.05 | |  | 53.04 | | 6.87 | | 51.63 | | 7.62 | | > 0.05 | |
| **CE(17:1)** | 45.00 | | | 5.90 | | 54.97 | | 7.36 | | > 0.05 | |  | 56.70 | | 6.96 | | 58.50 | | 7.27 | | > 0.05 | |
| **CE(18:0)** | 43.30 | | | 5.78 | | 45.41 | | 6.20 | | > 0.05 | |  | 44.73 | | 5.50 | | 51.10 | | 6.68 | | > 0.05 | |
| **CE(18:1)** | 1438.0 | | | 202.4 | | 1425.3 | | 215.0 | | > 0.05 | |  | 1415.3 | | 189.09 | | 1569.4 | | 237.23 | | > 0.05 | |
| **CE(18:2)** | 27164 | | | 4087 | | 24468 | | 3858 | | > 0.05 | |  | 24100 | | 3490 | | 24869 | | 3786 | | > 0.05 | |
| **CE(18:3)** | 2594 | | | 326 | | 2814 | | 426 | | > 0.05 | |  | 2887 | | 385 | | 3292 | | 489 | | > 0.05 | |
| **CE(20:0)** | 35.91 | | | 6.59 | | 30.12 | | 6.67 | | > 0.05 | |  | 34.18 | | 6.03 | | 33.43 | | 7.55 | | > 0.05 | |
| **CE(20:1)** | 14.46 | | | 2.53 | | 13.16 | | 2.66 | | > 0.05 | |  | 14.49 | | 2.21 | | 12.78 | | 2.59 | | > 0.05 | |
| **CE(20:3)** | 92.13 | | | 13.01 | | 107.29 | | 14.32 | | > 0.05 | |  | 103.98 | | 11.39 | | 121.81 | | 15.54 | | > 0.05 | |
| **CE(20:4)** | 567.35 | | | 54.39 | | 720.10 | | 96.36 | | > 0.05 | |  | 752.07 | | 86.07 | | 798.41 | | 111.73 | | > 0.05 | |
| **CE(20:5)** | 297.38 | | | 43.05 | | 332.25 | | 46.33 | | > 0.05 | |  | 327.94 | | 31.62 | | 326.79 | | 43.82 | | > 0.05 | |
| **CE(22:0)** | 0.07 | | | 0.03 | | 0.05 | | 0.02 | | > 0.05 | |  | 0.07 | | 0.03 | | 0.09 | | 0.03 | | > 0.05 | |
| **CE(22:1)** | 1.09 | | | 0.12 | | 1.12 | | 0.12 | | > 0.05 | |  | 1.22 | | 0.10 | | 1.25 | | 0.14 | | > 0.05 | |
| **CE(22:2)** | 0.34 | | | 0.04 | | 0.37 | | 0.05 | | > 0.05 | |  | 0.38 | | 0.04 | | 0.35 | | 0.04 | | > 0.05 | |
| **CE(22:5)** | 3.35 | | | 0.73 | | 2.56 | | 0.71 | | > 0.05 | |  | 4.71 | | 1.12 | | 4.27 | | 1.13 | | > 0.05 | |
| **CE(22:6)** | 39.55 | | | 4.39 | | 36.22 | | 3.56 | | > 0.05 | |  | 41.84 | | 4.55 | | 39.41 | | 4.41 | | > 0.05 | |

**Supplementary Table S12.**Biochemical abbreviation and Plasma concentration (µM) of Cresols in low (LRFI) and high (HRFI) Residual Feed Intake bulls on Day 0 and 56 of the feed efficiency trial (Mean ± SEM).

|  |  |  | **Day 0** |  | |  | |  | |  |  | | **Day 56** | |  | | |  |  |
| --- | --- | --- | --- | --- | --- | --- | --- | --- | --- | --- | --- | --- | --- | --- | --- | --- | --- | --- | --- |
|  |  | **LRFI** |  | **HRFI** | |  | |  | |  | **LRFI** | |  | | **HRFI** | | |  |  |
| **COMPOUND** | **Mean** | **SEM** | **Mean** | **SEM** | | ***P*** | |  | | **Mean** | **SEM** | | **Mean** | | **SEM** | | | ***P*** |  |
| **p-Cresol-SO4** | 48.21 | 3.86 | 47.86 | 4.11 | > 0.05 | |  | | 45.15 | | | 4.25 | | 41.51 | | 3.83 | > 0.05 | | |

**Supplementary Table S13.**Biochemical abbreviation and Plasma concentration (µM) of Diacylglycerols in low (LRFI) and high (HRFI) Residual Feed Intake bulls on Day 0 and 56 of the feed efficiency trial (Mean ± SEM).

|  |  |  | **Day 0** |  | |  | |  | |  |  | | **Day 56** | | |  |  | | |
| --- | --- | --- | --- | --- | --- | --- | --- | --- | --- | --- | --- | --- | --- | --- | --- | --- | --- | --- | --- |
|  |  | **LRFI** |  | **HRFI** | |  | |  | |  | **LRFI** | |  | | | **HRFI** |  | | |
| **COMPOUND** | **Mean** | **SEM** | **Mean** | **SEM** | | ***P*** | |  | | **Mean** | **SEM** | | **Mean** | | | **SEM** | ***P*** | | |
| **DG(14:0_14:0)** | 5.87 | 0.81 | 6.27 | 0.80 | > 0.05 | |  | | 6.28 | | | 0.76 | | 6.78 | 0.85 | | | > 0.05 |  |
| **DG(14:0_18:1)** | 1.71 | 0.24 | 1.72 | 0.22 | > 0.05 | |  | | 1.82 | | | 0.23 | | 1.94 | 0.24 | | | > 0.05 |  |
| **DG(14:0_18:2)** | 3.83 | 0.50 | 3.68 | 0.47 | > 0.05 | |  | | 4.02 | | | 0.51 | | 3.87 | 0.44 | | | > 0.05 |  |
| **DG(14:0_20:0)** | 0.02 | 0.00 | 0.02 | 0.00 | > 0.05 | |  | | 0.02 | | | 0.00 | | 0.01 | 0.00 | | | > 0.05 |  |
| **DG(14:1_18:1)** | 0.15 | 0.02 | 0.18 | 0.02 | > 0.05 | |  | | 0.20 | | | 0.02 | | 0.25 | 0.03 | | | > 0.05 |  |
| **DG(14:1_20:2)** | 0.03 | 0.01 | 0.02 | 0.01 | > 0.05 | |  | | 0.03 | | | 0.01 | | 0.04 | 0.01 | | | > 0.05 |  |
| **DG(16:0_16:0)** | 0.09 | 0.04 | 0.07 | 0.04 | > 0.05 | |  | | 0.14 | | | 0.05 | | 0.26 | 0.06 | | | > 0.05 |  |
| **DG(16:0_16:1)** | 2.64 | 0.47 | 3.47 | 0.56 | > 0.05 | |  | | 2.39 | | | 0.37 | | 2.12 | 0.41 | | | > 0.05 |  |
| **DG(16:0_18:1)** | 1.91 | 0.61 | 1.70 | 0.41 | > 0.05 | |  | | 2.27 | | | 0.51 | | 1.50 | 0.47 | | | > 0.05 |  |
| **DG(16:0_18:2)** | 2.49 | 0.34 | 3.27 | 0.38 | > 0.05 | |  | | 3.25 | | | 0.43 | | 2.65 | 0.32 | | | > 0.05 |  |
| **DG(16:0_20:0)** | 0.00 | 0.00 | 0.00 | 0.00 | > 0.05 | |  | | 0.00 | | | 0.00 | | 0.00 | 0.00 | | | > 0.05 |  |
| **DG(16:0_20:3)** | 0.47 | 0.08 | 0.56 | 0.07 | > 0.05 | |  | | 0.39 | | | 0.04 | | 0.38 | 0.06 | | | > 0.05 |  |
| **DG(16:0_20:4)** | 1.25 | 0.21 | 1.16 | 0.13 | > 0.05 | |  | | 1.08 | | | 0.18 | | 0.83 | 0.16 | | | > 0.05 |  |
| **DG(16:1_18:0)** | 0.30 | 0.04 | 0.33 | 0.04 | > 0.05 | |  | | 0.29 | | | 0.04 | | 0.27 | 0.04 | | | > 0.05 |  |
| **DG(16:1_18:1)** | 0.73 | 0.15 | 1.13 | 0.20 | > 0.05 | |  | | 0.73 | | | 0.16 | | 0.74 | 0.16 | | | > 0.05 |  |
| **DG(16:1_18:2)** | 2.61 | 0.37 | 3.29 | 0.43 | > 0.05 | |  | | 2.41 | | | 0.30 | | 2.16 | 0.29 | | | > 0.05 |  |
| **DG(17:0_17:1)** | 0.12 | 0.01 | 0.16 | 0.02 | > 0.05 | |  | | 0.12 | | | 0.01 | | 0.13 | 0.02 | | | > 0.05 |  |
| **DG(17:0_18:1)** | 0.41 | 0.05 | 0.37 | 0.04 | > 0.05 | |  | | 0.45 | | | 0.05 | | 0.38 | 0.05 | | | > 0.05 |  |
| **DG(18:0_20:4)** | 0.54 | 0.07 | 0.63 | 0.08 | > 0.05 | |  | | 0.63 | | | 0.09 | | 0.49 | 0.07 | | | > 0.05 |  |
| **DG(18:1_18:1)** | 0.47 | 0.06 | 0.55 | 0.07 | > 0.05 | |  | | 0.53 | | | 0.07 | | 0.42 | 0.07 | | | > 0.05 |  |
| **DG(18:1_18:2)** | 0.89 | 0.13 | 1.01 | 0.15 | > 0.05 | |  | | 0.84 | | | 0.10 | | 0.69 | 0.10 | | | > 0.05 |  |
| **DG(18:1_18:3)** | 0.42 | 0.10 | 0.49 | 0.10 | > 0.05 | |  | | 0.37 | | | 0.10 | | 0.27 | 0.08 | | | > 0.05 |  |
| **DG(18:1_18:4)** | 0.36 | 0.06 | 0.48 | 0.06 | > 0.05 | |  | | 0.44 | | | 0.07 | | 0.31 | 0.04 | | | > 0.05 |  |
| **DG(18:1_20:1)** | 0.04 | 0.00 | 0.04 | 0.00 | > 0.05 | |  | | 0.03 | | | 0.00 | | 0.04 | 0.00 | | | > 0.05 |  |
| **DG(18:1_20:2)** | 0.64 | 0.07 | 0.80 | 0.10 | > 0.05 | |  | | 0.64 | | | 0.09 | | 0.51 | 0.07 | | | > 0.05 |  |
| **DG(18:1_20:3)** | 0.12 | 0.02 | 0.10 | 0.01 | > 0.05 | |  | | 0.09 | | | 0.01 | | 0.10 | 0.01 | | | > 0.05 |  |
| **DG(18:1_20:4)** | 0.42 | 0.06 | 0.47 | 0.07 | > 0.05 | |  | | 0.38 | | | 0.06 | | 0.32 | 0.04 | | | > 0.05 |  |
| **DG(18:1_22:5)** | 0.02 | 0.00 | 0.02 | 0.00 | > 0.05 | |  | | 0.01 | | | 0.00 | | 0.01 | 0.00 | | | > 0.05 |  |
| **DG(18:1_22:6)** | 0.16 | 0.03 | 0.19 | 0.04 | > 0.05 | |  | | 0.13 | | | 0.03 | | 0.12 | 0.03 | | | > 0.05 |  |
| **DG(18:2_18:2)** | 3.12 | 0.39 | 3.48 | 0.40 | > 0.05 | |  | | 3.22 | | | 0.52 | | 3.18 | 0.49 | | | > 0.05 |  |
| **DG(18:2_18:3)** | 1.72 | 0.23 | 1.84 | 0.24 | > 0.05 | |  | | 1.52 | | | 0.22 | | 1.18 | 0.16 | | | > 0.05 |  |
| **DG(18:2_18:4)** | 0.95 | 0.16 | 1.14 | 0.15 | > 0.05 | |  | | 0.84 | | | 0.13 | | 0.73 | 0.12 | | | > 0.05 |  |
| **DG(18:2_20:0)** | 0.71 | 0.09 | 0.72 | 0.11 | > 0.05 | |  | | 0.79 | | | 0.10 | | 0.64 | 0.07 | | | > 0.05 |  |
| **DG(18:2_20:4)** | 1.38 | 0.19 | 1.47 | 0.19 | > 0.05 | |  | | 1.02 | | | 0.11 | | 1.04 | 0.16 | | | > 0.05 |  |
| **DG(18:3_18:3)** | 3.69 | 0.53 | 4.54 | 0.52 | > 0.05 | |  | | 3.30 | | | 0.37 | | 3.54 | 0.50 | | | > 0.05 |  |
| **DG(18:3_20:2)** | 0.01 | 0.00 | 0.01 | 0.00 | > 0.05 | |  | | 0.01 | | | 0.00 | | 0.00 | 0.00 | | | > 0.05 |  |
| **DG(21:0_22:6)** | 0.00 | 0.00 | 0.01 | 0.00 | > 0.05 | |  | | 0.01 | | | 0.00 | | 0.00 | 0.00 | | | > 0.05 |  |
| **DG(22:1_22:2)** | 0.03 | 0.01 | 0.03 | 0.01 | > 0.05 | |  | | 0.04 | | | 0.01 | | 0.05 | 0.01 | | | > 0.05 |  |
| **DG-O(14:0_18:2)** | 0.00 | 0.00 | 0.00 | 0.00 | > 0.05 | |  | | 0.00 | | | 0.00 | | 0.00 | 0.00 | | | > 0.05 |  |
| **DG-O(16:0_20:4)** | 0.72 | 0.05 | 0.62 | 0.06 | > 0.05 | |  | | 0.59 | | | 0.06 | | 0.62 | 0.05 | | | > 0.05 |  |
| **DG-O(18:2_18:2)** | 0.24 | 0.02 | 0.23 | 0.02 | > 0.05 | |  | | 0.23 | | | 0.02 | | 0.25 | 0.02 | | | > 0.05 |  |

**Supplementary Table S14.**Biochemical abbreviation and Plasma concentration (µM) of Dihydroceramides in low (LRFI) and high (HRFI) Residual Feed Intake bulls on Day 0 and 56 of the feed efficiency trial (Mean ± SEM).

|  |  |  | **Day 0** |  | |  | |  | |  |  | | **Day 56** | |  | | |  | |
| --- | --- | --- | --- | --- | --- | --- | --- | --- | --- | --- | --- | --- | --- | --- | --- | --- | --- | --- | --- |
|  |  | **LRFI** |  | **HRFI** | |  | |  | |  | **LRFI** | |  | | **HRFI** | | |  | |
| **COMPOUND** | **Mean** | **SEM** | **Mean** | **SEM** | | ***P*** | |  | | **Mean** | **SEM** | | **Mean** | | **SEM** | | | ***P*** | |
| **Cer(d18:0/16:0)** | 83.46 | 4.73 | 82.53 | 4.41 | > 0.05 | |  | | 66.18 | | | 5.02 | | 67.81 | | 5.52 | > 0.05 | |  |
| **Cer(d18:0/18:0(OH))** | 0.62 | 0.04 | 0.60 | 0.04 | > 0.05 | |  | | 0.70 | | | 0.04 | | 0.56 | | 0.04 | 0.02 | |  |
| **Cer(d18:0/18:0)** | 0.03 | 0.01 | 0.04 | 0.01 | > 0.05 | |  | | 0.04 | | | 0.01 | | 0.03 | | 0.01 | > 0.05 | |  |
| **Cer(d18:0/20:0)** | 0.01 | 0.00 | 0.01 | 0.00 | > 0.05 | |  | | 0.02 | | | 0.01 | | 0.02 | | 0.01 | > 0.05 | |  |
| **Cer(d18:0/22:0)** | 0.14 | 0.01 | 0.15 | 0.01 | > 0.05 | |  | | 0.15 | | | 0.01 | | 0.13 | | 0.01 | > 0.05 | |  |
| **Cer(d18:0/24:0)** | 0.30 | 0.03 | 0.31 | 0.02 | > 0.05 | |  | | 0.32 | | | 0.03 | | 0.28 | | 0.02 | > 0.05 | |  |
| **Cer(d18:0/24:1)** | 0.38 | 0.03 | 0.38 | 0.02 | > 0.05 | |  | | 0.39 | | | 0.02 | | 0.37 | | 0.03 | > 0.05 | |  |
| **Cer(d18:0/26:1(OH))** | 1.55 | 0.11 | 1.68 | 0.10 | > 0.05 | |  | | 1.55 | | | 0.11 | | 1.56 | | 0.11 | > 0.05 | |  |

**Supplementary Table S15.**Biochemical abbreviation and Plasma concentration (µM) of Fatty Acids in low (LRFI) and high (HRFI) Residual Feed Intake bulls on Day 0 and 56 of the feed efficiency trial (Mean ± SEM).

|  |  |  | **Day 0** | |  | |  | |  | | |  |  | | **Day 56** | |  | |  | |  |
| --- | --- | --- | --- | --- | --- | --- | --- | --- | --- | --- | --- | --- | --- | --- | --- | --- | --- | --- | --- | --- | --- |
|  |  | **LRFI** |  | | **HRFI** | |  | |  | | |  | **LRFI** | |  | | **HRFI** | |  | |  |
| **COMPOUND** | **Mean** | **SEM** | **Mean** | | **SEM** | | ***P*** | |  | | | **Mean** | **SEM** | | **Mean** | | **SEM** | | ***P*** | |  |
| **AA** | 0.57 | 0.13 | | 0.63 | | 0.13 | | > 0.05 | |  | 0.97 | | | 0.13 | | 0.91 | | 0.14 | | > 0.05 | |
| **DHA** | 0.70 | 0.05 | | 0.65 | | 0.06 | | > 0.05 | |  | 0.58 | | | 0.04 | | 0.58 | | 0.05 | | > 0.05 | |
| **EPA** | 0.16 | 0.02 | | 0.15 | | 0.02 | | > 0.05 | |  | 0.13 | | | 0.02 | | 0.14 | | 0.02 | | > 0.05 | |
| **FA(12:0)** | 810.55 | 302.79 | | 884.21 | | 443.5 | | > 0.05 | |  | 988.88 | | | 458.47 | | 391.63 | | 113.21 | | > 0.05 | |
| **FA(14:0)** | 2049.60 | 831.84 | | 1090.7 | | 224.9 | | > 0.05 | |  | 2517.62 | | | 933.76 | | 897.20 | | 461.27 | | 0.02 | |
| **FA(16:0)** | 196.97 | 4.13 | | 196.48 | | 4.15 | | > 0.05 | |  | 201.62 | | | 4.05 | | 198.15 | | 5.17 | | > 0.05 | |
| **FA(18:0)** | 151.96 | 4.08 | | 157.68 | | 5.65 | | > 0.05 | |  | 169.55 | | | 5.89 | | 159.04 | | 6.19 | | > 0.05 | |
| **FA(18:1)** | 52.92 | 4.64 | | 45.49 | | 3.50 | | > 0.05 | |  | 40.49 | | | 4.48 | | 41.81 | | 4.75 | | > 0.05 | |
| **FA(18:2)** | 8.03 | 0.44 | | 7.78 | | 0.40 | | > 0.05 | |  | 8.26 | | | 0.49 | | 8.02 | | 0.52 | | > 0.05 | |
| **FA(20:1)** | 0.78 | 0.08 | | 0.87 | | 0.11 | | > 0.05 | |  | 0.87 | | | 0.09 | | 0.88 | | 0.09 | | > 0.05 | |
| **FA(20:2)** | 0.67 | 0.09 | | 0.79 | | 0.08 | | > 0.05 | |  | 0.62 | | | 0.07 | | 0.70 | | 0.10 | | > 0.05 | |
| **FA(20:3)** | 0.20 | 0.04 | | 0.28 | | 0.05 | | > 0.05 | |  | 0.32 | | | 0.05 | | 0.33 | | 0.05 | | > 0.05 | |

**Supplementary Table S16.**Biochemical abbreviation and Plasma concentration (µM) of Glycerophospholipids in low (LRFI) and high (HRFI) Residual Feed Intake bulls on Day 0 and 56 of the feed efficiency trial (Mean ± SEM).

|  |  |  | **Day 0** |  | |  | |  | |  | |  | | **Day 56** | |  | |  | |
| --- | --- | --- | --- | --- | --- | --- | --- | --- | --- | --- | --- | --- | --- | --- | --- | --- | --- | --- | --- |
|  |  | **LRFI** |  | **HRFI** | |  | |  | |  | | **LRFI** | |  | | **HRFI** | |  | |
| **COMPOUND** | **Mean** | **SEM** | **Mean** | **SEM** | | ***P*** | |  | | **Mean** | | **SEM** | | **Mean** | | **SEM** | | ***P*** | |
| **lysoPC a C14:0** | 3.10 | 0.13 | 3.21 | 0.14 | > 0.05 | |  | | 3.09 | | 0.12 | | 3.34 | | 0.14 | | > 0.05 | |  |
| **lysoPC a C16:0** | 22.65 | 1.51 | 21.75 | 1.60 | > 0.05 | |  | | 24.22 | | 1.48 | | 22.63 | | 1.80 | | > 0.05 | |  |
| **lysoPC a C16:1** | 1.14 | 0.06 | 1.18 | 0.05 | > 0.05 | |  | | 1.25 | | 0.06 | | 1.22 | | 0.07 | | > 0.05 | |  |
| **lysoPC a C17:0** | 3.52 | 0.27 | 4.12 | 0.37 | > 0.05 | |  | | 4.18 | | 0.32 | | 4.39 | | 0.35 | | > 0.05 | |  |
| **lysoPC a C18:0** | 27.11 | 2.00 | 26.06 | 2.05 | > 0.05 | |  | | 29.82 | | 2.06 | | 28.01 | | 2.56 | | > 0.05 | |  |
| **lysoPC a C18:1** | 10.12 | 0.66 | 9.83 | 0.60 | > 0.05 | |  | | 10.92 | | 0.75 | | 10.89 | | 0.83 | | > 0.05 | |  |
| **lysoPC a C18:2** | 25.80 | 1.90 | 23.33 | 1.94 | > 0.05 | |  | | 27.21 | | 2.43 | | 25.72 | | 2.69 | | > 0.05 | |  |
| **lysoPC a C20:3** | 1.05 | 0.06 | 1.04 | 0.06 | > 0.05 | |  | | 1.01 | | 0.06 | | 0.98 | | 0.07 | | > 0.05 | |  |
| **lysoPC a C20:4** | 1.32 | 0.09 | 1.16 | 0.06 | > 0.05 | |  | | 1.37 | | 0.08 | | 1.33 | | 0.11 | | > 0.05 | |  |
| **lysoPC a C24:0** | 0.13 | 0.01 | 0.11 | 0.01 | > 0.05 | |  | | 0.11 | | 0.01 | | 0.11 | | 0.01 | | > 0.05 | |  |
| **lysoPC a C26:0** | 0.13 | 0.01 | 0.13 | 0.01 | > 0.05 | |  | | 0.15 | | 0.01 | | 0.18 | | 0.01 | | > 0.05 | |  |
| **lysoPC a C26:1** | 0.12 | 0.01 | 0.13 | 0.01 | > 0.05 | |  | | 0.16 | | 0.01 | | 0.16 | | 0.01 | | > 0.05 | |  |
| **lysoPC a C28:0** | 0.48 | 0.04 | 0.45 | 0.04 | > 0.05 | |  | | 0.44 | | 0.04 | | 0.41 | | 0.03 | | > 0.05 | |  |
| **lysoPC a C28:1** | 0.37 | 0.02 | 0.34 | 0.03 | > 0.05 | |  | | 0.37 | | 0.02 | | 0.41 | | 0.03 | | > 0.05 | |  |
| **PC aa C24:0** | 0.03 | 0.00 | 0.03 | 0.00 | > 0.05 | |  | | 0.04 | | 0.00 | | 0.03 | | 0.00 | | > 0.05 | |  |
| **PC aa C26:0** | 0.26 | 0.01 | 0.25 | 0.01 | > 0.05 | |  | | 0.26 | | 0.01 | | 0.26 | | 0.01 | | > 0.05 | |  |
| **PC aa C28:1** | 1.40 | 0.08 | 1.31 | 0.12 | > 0.05 | |  | | 1.51 | | 0.08 | | 1.52 | | 0.11 | | > 0.05 | |  |
| **PC aa C30:0** | 2.68 | 0.11 | 2.56 | 0.17 | > 0.05 | |  | | 3.01 | | 0.15 | | 2.93 | | 0.17 | | > 0.05 | |  |
| **PC aa C32:0** | 11.78 | 0.75 | 10.73 | 0.80 | > 0.05 | |  | | 11.37 | | 0.67 | | 10.93 | | 0.78 | | > 0.05 | |  |
| **PC aa C32:1** | 6.60 | 0.45 | 6.60 | 0.55 | > 0.05 | |  | | 6.84 | | 0.59 | | 6.92 | | 0.50 | | > 0.05 | |  |
| **PC aa C32:2** | 11.28 | 1.20 | 9.87 | 1.20 | > 0.05 | |  | | 10.50 | | 1.06 | | 11.03 | | 1.21 | | > 0.05 | |  |
| **PC aa C32:3** | 82.46 | 9.04 | 72.62 | 11.47 | > 0.05 | |  | | 68.53 | | 7.71 | | 80.02 | | 9.89 | | > 0.05 | |  |
| **PC aa C34:1** | 71.99 | 5.29 | 65.64 | 5.57 | > 0.05 | |  | | 72.83 | | 5.62 | | 73.25 | | 5.75 | | > 0.05 | |  |
| **PC aa C34:2** | 294.90 | 20.90 | 253.09 | 24.69 | > 0.05 | |  | | 272.68 | | 20.06 | | 265.36 | | 22.13 | | > 0.05 | |  |
| **PC aa C34:3** | 21.04 | 1.60 | 20.11 | 2.00 | > 0.05 | |  | | 20.66 | | 1.76 | | 19.90 | | 1.77 | | > 0.05 | |  |
| **PC aa C34:4** | 7.12 | 0.69 | 7.12 | 0.94 | > 0.05 | |  | | 7.35 | | 0.74 | | 7.39 | | 0.76 | | > 0.05 | |  |
| **PC aa C36:0** | 1.03 | 0.20 | 1.19 | 0.16 | > 0.05 | |  | | 1.42 | | 0.30 | | 0.65 | | 0.14 | | > 0.05 | |  |
| **PC aa C36:1** | 153.92 | 12.52 | 132.51 | 14.31 | > 0.05 | |  | | 146.40 | | 12.70 | | 156.95 | | 13.76 | | > 0.05 | |  |
| **PC aa C36:2** | 300.62 | 25.71 | 262.30 | 21.77 | > 0.05 | |  | | 324.85 | | 27.71 | | 278.48 | | 24.53 | | > 0.05 | |  |
| **PC aa C36:3** | 123.45 | 9.24 | 127.81 | 11.04 | > 0.05 | |  | | 135.48 | | 11.77 | | 125.28 | | 10.21 | | > 0.05 | |  |
| **PC aa C36:4** | 32.85 | 2.61 | 30.64 | 3.13 | > 0.05 | |  | | 35.13 | | 3.13 | | 28.73 | | 2.06 | | > 0.05 | |  |
| **PC aa C36:5** | 5.31 | 0.39 | 4.71 | 0.45 | > 0.05 | |  | | 5.00 | | 0.36 | | 4.89 | | 0.40 | | > 0.05 | |  |
| **PC aa C36:6** | 2.92 | 0.25 | 2.49 | 0.26 | > 0.05 | |  | | 2.87 | | 0.22 | | 3.17 | | 0.32 | | > 0.05 | |  |
| **PC aa C38:0** | 0.79 | 0.06 | 0.69 | 0.06 | > 0.05 | |  | | 0.92 | | 0.08 | | 0.71 | | 0.07 | | 0.03 | |  |
| **PC aa C38:3** | 34.06 | 2.77 | 32.82 | 3.18 | > 0.05 | |  | | 35.45 | | 3.15 | | 38.20 | | 3.08 | | > 0.05 | |  |
| **PC aa C38:4** | 40.59 | 2.75 | 39.33 | 3.29 | > 0.05 | |  | | 48.65 | | 3.77 | | 41.51 | | 3.23 | | > 0.05 | |  |
| **PC aa C38:5** | 13.30 | 0.82 | 11.99 | 0.86 | > 0.05 | |  | | 13.14 | | 0.91 | | 12.10 | | 0.85 | | > 0.05 | |  |
| **PC aa C38:6** | 3.41 | 0.18 | 3.15 | 0.23 | > 0.05 | |  | | 3.32 | | 0.23 | | 3.38 | | 0.24 | | > 0.05 | |  |
| **PC aa C40:1** | 0.02 | 0.00 | 0.03 | 0.00 | > 0.05 | |  | | 0.03 | | 0.00 | | 0.02 | | 0.00 | | > 0.05 | |  |
| **PC aa C40:2** | 0.24 | 0.03 | 0.21 | 0.03 | > 0.05 | |  | | 0.22 | | 0.03 | | 0.25 | | 0.02 | | > 0.05 | |  |
| **PC aa C40:3** | 0.51 | 0.05 | 0.57 | 0.05 | > 0.05 | |  | | 0.57 | | 0.07 | | 0.58 | | 0.05 | | > 0.05 | |  |
| **PC aa C40:4** | 6.35 | 0.41 | 6.46 | 0.38 | > 0.05 | |  | | 7.38 | | 0.53 | | 7.33 | | 0.50 | | > 0.05 | |  |
| **PC aa C40:5** | 13.27 | 0.91 | 12.02 | 0.76 | > 0.05 | |  | | 14.81 | | 0.93 | | 12.71 | | 0.87 | | > 0.05 | |  |
| **PC aa C40:6** | 4.99 | 0.35 | 4.37 | 0.30 | > 0.05 | |  | | 5.62 | | 0.41 | | 4.69 | | 0.36 | | > 0.05 | |  |
| **PC aa C42:0** | 0.08 | 0.01 | 0.10 | 0.01 | 0.03 | |  | | 0.09 | | 0.01 | | 0.10 | | 0.01 | | > 0.05 | |  |
| **PC aa C42:1** | 0.15 | 0.01 | 0.15 | 0.01 | > 0.05 | |  | | 0.17 | | 0.01 | | 0.15 | | 0.01 | | > 0.05 | |  |
| **PC aa C42:2** | 0.40 | 0.03 | 0.36 | 0.03 | > 0.05 | |  | | 0.45 | | 0.04 | | 0.39 | | 0.03 | | > 0.05 | |  |
| **PC aa C42:4** | 0.13 | 0.01 | 0.12 | 0.01 | > 0.05 | |  | | 0.13 | | 0.01 | | 0.13 | | 0.01 | | > 0.05 | |  |
| **PC aa C42:5** | 0.22 | 0.02 | 0.19 | 0.02 | > 0.05 | |  | | 0.23 | | 0.02 | | 0.20 | | 0.02 | | > 0.05 | |  |
| **PC aa C42:6** | 0.29 | 0.03 | 0.30 | 0.04 | > 0.05 | |  | | 0.34 | | 0.04 | | 0.37 | | 0.03 | | > 0.05 | |  |
| **PC ae C30:0** | 0.62 | 0.03 | 0.61 | 0.03 | > 0.05 | |  | | 0.66 | | 0.03 | | 0.68 | | 0.04 | | > 0.05 | |  |
| **PC ae C30:1** | 1.43 | 0.16 | 2.06 | 0.26 | > 0.05 | |  | | 1.96 | | 0.19 | | 2.04 | | 0.24 | | > 0.05 | |  |
| **PC ae C30:2** | 0.30 | 0.02 | 0.33 | 0.03 | > 0.05 | |  | | 0.37 | | 0.02 | | 0.37 | | 0.03 | | > 0.05 | |  |
| **PC ae C32:1** | 5.00 | 0.42 | 4.69 | 0.51 | > 0.05 | |  | | 5.19 | | 0.43 | | 5.45 | | 0.43 | | > 0.05 | |  |
| **PC ae C32:2** | 7.52 | 0.63 | 7.66 | 0.96 | > 0.05 | |  | | 9.77 | | 0.88 | | 9.27 | | 0.86 | | > 0.05 | |  |
| **PC ae C34:0** | 6.55 | 0.55 | 5.26 | 0.51 | > 0.05 | |  | | 6.46 | | 0.44 | | 6.55 | | 0.50 | | > 0.05 | |  |
| **PC ae C34:1** | 18.43 | 1.25 | 16.08 | 1.34 | > 0.05 | |  | | 18.14 | | 1.37 | | 18.22 | | 1.37 | | > 0.05 | |  |
| **PC ae C34:2** | 35.39 | 3.02 | 30.13 | 3.25 | > 0.05 | |  | | 33.27 | | 2.63 | | 34.47 | | 2.63 | | > 0.05 | |  |
| **PC ae C34:3** | 47.55 | 5.34 | 42.89 | 5.95 | > 0.05 | |  | | 45.55 | | 4.85 | | 47.57 | | 4.54 | | > 0.05 | |  |
| **PC ae C36:0** | 3.41 | 0.32 | 3.08 | 0.33 | > 0.05 | |  | | 3.53 | | 0.27 | | 3.48 | | 0.30 | | > 0.05 | |  |
| **PC ae C36:1** | 26.07 | 2.11 | 22.98 | 2.02 | > 0.05 | |  | | 27.41 | | 2.51 | | 26.54 | | 2.27 | | > 0.05 | |  |
| **PC ae C36:2** | 95.31 | 9.11 | 82.77 | 8.71 | > 0.05 | |  | | 86.50 | | 6.82 | | 96.37 | | 8.61 | | > 0.05 | |  |
| **PC ae C36:3** | 15.46 | 1.21 | 13.36 | 1.29 | > 0.05 | |  | | 15.43 | | 1.16 | | 14.14 | | 1.03 | | > 0.05 | |  |
| **PC ae C36:4** | 8.81 | 0.71 | 8.86 | 0.96 | > 0.05 | |  | | 10.06 | | 0.86 | | 9.24 | | 0.67 | | > 0.05 | |  |
| **PC ae C36:5** | 6.22 | 0.51 | 5.86 | 0.58 | > 0.05 | |  | | 6.48 | | 0.53 | | 6.23 | | 0.44 | | > 0.05 | |  |
| **PC ae C38:0** | 0.87 | 0.07 | 0.71 | 0.07 | > 0.05 | |  | | 0.84 | | 0.07 | | 0.84 | | 0.07 | | > 0.05 | |  |
| **PC ae C38:1** | 3.05 | 0.31 | 3.12 | 0.44 | > 0.05 | |  | | 3.63 | | 0.28 | | 3.60 | | 0.28 | | > 0.05 | |  |
| **PC ae C38:2** | 10.01 | 0.84 | 9.59 | 0.98 | > 0.05 | |  | | 10.08 | | 0.85 | | 10.11 | | 0.89 | | > 0.05 | |  |
| **PC ae C38:3** | 7.28 | 0.62 | 7.24 | 0.65 | > 0.05 | |  | | 7.85 | | 0.70 | | 7.96 | | 0.66 | | > 0.05 | |  |
| **PC ae C38:4** | 7.95 | 0.62 | 7.11 | 0.59 | > 0.05 | |  | | 7.51 | | 0.51 | | 7.71 | | 0.58 | | > 0.05 | |  |
| **PC ae C38:5** | 5.13 | 0.40 | 4.43 | 0.38 | > 0.05 | |  | | 5.18 | | 0.42 | | 4.84 | | 0.32 | | > 0.05 | |  |
| **PC ae C38:6** | 3.44 | 0.26 | 3.05 | 0.26 | > 0.05 | |  | | 3.26 | | 0.24 | | 3.66 | | 0.32 | | > 0.05 | |  |
| **PC ae C40:1** | 0.18 | 0.02 | 0.14 | 0.02 | > 0.05 | |  | | 0.17 | | 0.03 | | 0.19 | | 0.04 | | > 0.05 | |  |
| **PC ae C40:2** | 1.00 | 0.06 | 0.96 | 0.07 | > 0.05 | |  | | 0.99 | | 0.06 | | 1.08 | | 0.07 | | > 0.05 | |  |
| **PC ae C40:3** | 1.31 | 0.11 | 1.37 | 0.10 | > 0.05 | |  | | 1.37 | | 0.09 | | 1.49 | | 0.11 | | > 0.05 | |  |
| **PC ae C40:4** | 1.77 | 0.16 | 1.91 | 0.13 | > 0.05 | |  | | 2.03 | | 0.14 | | 1.91 | | 0.12 | | > 0.05 | |  |
| **PC ae C40:5** | 3.25 | 0.21 | 2.87 | 0.22 | > 0.05 | |  | | 3.26 | | 0.19 | | 3.25 | | 0.23 | | > 0.05 | |  |
| **PC ae C40:6** | 1.37 | 0.08 | 1.31 | 0.11 | > 0.05 | |  | | 1.38 | | 0.10 | | 1.34 | | 0.10 | | > 0.05 | |  |
| **PC ae C42:0** | 0.03 | 0.00 | 0.04 | 0.00 | > 0.05 | |  | | 0.03 | | 0.00 | | 0.04 | | 0.00 | | 0.02 | |  |
| **PC ae C42:1** | 0.17 | 0.01 | 0.18 | 0.01 | > 0.05 | |  | | 0.18 | | 0.01 | | 0.17 | | 0.01 | | > 0.05 | |  |
| **PC ae C42:2** | 0.23 | 0.02 | 0.20 | 0.02 | > 0.05 | |  | | 0.25 | | 0.02 | | 0.24 | | 0.02 | | > 0.05 | |  |
| **PC ae C42:3** | 0.17 | 0.02 | 0.14 | 0.01 | > 0.05 | |  | | 0.19 | | 0.02 | | 0.18 | | 0.01 | | > 0.05 | |  |
| **PC ae C42:4** | 0.02 | 0.00 | 0.03 | 0.00 | 0.003 | |  | | 0.02 | | 0.00 | | 0.02 | | 0.00 | | > 0.05 | |  |
| **PC ae C42:5** | 0.23 | 0.07 | 0.32 | 0.09 | > 0.05 | |  | | 0.28 | | 0.09 | | 0.36 | | 0.09 | | > 0.05 | |  |
| **PC ae C44:3** | 0.13 | 0.01 | 0.12 | 0.01 | > 0.05 | |  | | 0.15 | | 0.01 | | 0.14 | | 0.01 | | > 0.05 | |  |
| **PC ae C44:4** | 0.10 | 0.01 | 0.10 | 0.01 | > 0.05 | |  | | 0.09 | | 0.02 | | 0.07 | | 0.01 | | > 0.05 | |  |
| **PC ae C44:5** | 0.10 | 0.01 | 0.10 | 0.01 | > 0.05 | |  | | 0.11 | | 0.01 | | 0.11 | | 0.01 | | > 0.05 | |  |
| **PC ae C44:6** | 0.02 | 0.00 | 0.02 | 0.00 | > 0.05 | |  | | 0.02 | | 0.00 | | 0.02 | | 0.00 | | > 0.05 | |  |

**Supplementary Table S17.**Biochemical abbreviation and Plasma concentration (µM) of Glycosylceramides in low (LRFI) and high (HRFI) Residual Feed Intake bulls on Day 0 and 56 of the feed efficiency trial (Mean ± SEM).

|  |  |  | **Day 0** |  | |  | |  | |  |  | | **Day 56** | | |  |  | |  |
| --- | --- | --- | --- | --- | --- | --- | --- | --- | --- | --- | --- | --- | --- | --- | --- | --- | --- | --- | --- |
|  |  | **LRFI** |  | **HRFI** | |  | |  | |  | **LRFI** | |  | | | **HRFI** |  | |  |
| **COMPOUND** | **Mean** | **SEM** | **Mean** | **SEM** | | ***P*** | |  | | **Mean** | **SEM** | | **Mean** | | | **SEM** | ***P*** | |  |
| **Hex2Cer(d18:1/14:0)** | 0.06 | 0.01 | 0.06 | 0.01 | > 0.05 | |  | | 0.06 | | | 0.01 | | 0.06 | 0.01 | | | > 0.05 | |
| **Hex2Cer(d18:1/16:0)** | 0.48 | 0.04 | 0.51 | 0.04 | > 0.05 | |  | | 0.51 | | | 0.04 | | 0.48 | 0.05 | | | > 0.05 | |
| **Hex2Cer(d18:1/18:0)** | 0.07 | 0.01 | 0.06 | 0.01 | > 0.05 | |  | | 0.06 | | | 0.01 | | 0.05 | 0.01 | | | > 0.05 | |
| **Hex2Cer(d18:1/20:0)** | 0.01 | 0.00 | 0.01 | 0.00 | > 0.05 | |  | | 0.01 | | | 0.00 | | 0.01 | 0.00 | | | > 0.05 | |
| **Hex2Cer(d18:1/22:0)** | 0.02 | 0.00 | 0.02 | 0.00 | > 0.05 | |  | | 0.02 | | | 0.00 | | 0.02 | 0.00 | | | > 0.05 | |
| **Hex2Cer(d18:1/24:0)** | 0.03 | 0.00 | 0.03 | 0.00 | > 0.05 | |  | | 0.03 | | | 0.00 | | 0.03 | 0.00 | | | > 0.05 | |
| **Hex2Cer(d18:1/24:1)** | 0.02 | 0.00 | 0.03 | 0.00 | > 0.05 | |  | | 0.03 | | | 0.00 | | 0.02 | 0.00 | | | > 0.05 | |
| **Hex2Cer(d18:1/26:0)** | 0.01 | 0.00 | 0.01 | 0.00 | > 0.05 | |  | | 0.01 | | | 0.00 | | 0.01 | 0.00 | | | > 0.05 | |
| **Hex2Cer(d18:1/26:1)** | 0.01 | 0.00 | 0.01 | 0.00 | > 0.05 | |  | | 0.01 | | | 0.00 | | 0.01 | 0.00 | | | > 0.05 | |
| **Hex3Cer(d18:1/16:0)** | 0.19 | 0.02 | 0.16 | 0.02 | > 0.05 | |  | | 0.21 | | | 0.02 | | 0.18 | 0.02 | | | > 0.05 | |
| **Hex3Cer(d18:1/18:0)** | 0.04 | 0.00 | 0.04 | 0.00 | > 0.05 | |  | | 0.03 | | | 0.00 | | 0.04 | 0.00 | | | > 0.05 | |
| **Hex3Cer(d18:1/24:1)** | 0.05 | 0.01 | 0.05 | 0.00 | > 0.05 | |  | | 0.05 | | | 0.00 | | 0.05 | 0.00 | | | > 0.05 | |
| **Hex3Cer(d18:1/26:1)** | 0.01 | 0.00 | 0.01 | 0.00 | > 0.05 | |  | | 0.01 | | | 0.00 | | 0.01 | 0.00 | | | > 0.05 | |
| **Hex3Cer(d18:1_20:0)** | 0.00 | 0.00 | 0.00 | 0.00 | > 0.05 | |  | | 0.00 | | | 0.00 | | 0.00 | 0.00 | | | > 0.05 | |
| **Hex3Cer(d18:1_22:0)** | 0.02 | 0.00 | 0.02 | 0.00 | > 0.05 | |  | | 0.02 | | | 0.00 | | 0.02 | 0.00 | | | > 0.05 | |
| **HexCer(d16:1/22:0)** | 0.04 | 0.00 | 0.04 | 0.00 | > 0.05 | |  | | 0.04 | | | 0.00 | | 0.04 | 0.00 | | | > 0.05 | |
| **HexCer(d16:1/24:0)** | 0.03 | 0.00 | 0.03 | 0.00 | > 0.05 | |  | | 0.03 | | | 0.00 | | 0.03 | 0.00 | | | > 0.05 | |
| **HexCer(d18:1/14:0)** | 0.02 | 0.00 | 0.03 | 0.00 | > 0.05 | |  | | 0.02 | | | 0.00 | | 0.03 | 0.00 | | | > 0.05 | |
| **HexCer(d18:1/16:0)** | 0.25 | 0.02 | 0.26 | 0.03 | > 0.05 | |  | | 0.27 | | | 0.03 | | 0.24 | 0.03 | | | > 0.05 | |
| **HexCer(d18:1/18:0)** | 0.06 | 0.01 | 0.07 | 0.01 | > 0.05 | |  | | 0.05 | | | 0.01 | | 0.05 | 0.01 | | | > 0.05 | |
| **HexCer(d18:1/18:1)** | 0.17 | 0.01 | 0.17 | 0.01 | > 0.05 | |  | | 0.19 | | | 0.01 | | 0.19 | 0.01 | | | > 0.05 | |
| **HexCer(d18:1/20:0)** | 0.20 | 0.01 | 0.18 | 0.01 | > 0.05 | |  | | 0.21 | | | 0.01 | | 0.21 | 0.01 | | | > 0.05 | |
| **HexCer(d18:1/22:0)** | 0.75 | 0.05 | 0.68 | 0.05 | > 0.05 | |  | | 0.62 | | | 0.06 | | 0.62 | 0.05 | | | > 0.05 | |
| **HexCer(d18:1/23:0)** | 0.35 | 0.03 | 0.29 | 0.03 | > 0.05 | |  | | 0.33 | | | 0.03 | | 0.30 | 0.03 | | | > 0.05 | |
| **HexCer(d18:1/24:0)** | 0.19 | 0.02 | 0.19 | 0.02 | > 0.05 | |  | | 0.16 | | | 0.02 | | 0.19 | 0.01 | | | > 0.05 | |
| **HexCer(d18:1/24:1)** | 0.84 | 0.08 | 0.75 | 0.06 | > 0.05 | |  | | 0.69 | | | 0.06 | | 0.62 | 0.05 | | | > 0.05 | |
| **HexCer(d18:1/26:0)** | 0.07 | 0.01 | 0.07 | 0.00 | > 0.05 | |  | | 0.07 | | | 0.00 | | 0.07 | 0.00 | | | > 0.05 | |
| **HexCer(d18:1/26:1)** | 0.10 | 0.01 | 0.09 | 0.01 | > 0.05 | |  | | 0.10 | | | 0.00 | | 0.09 | 0.01 | | | > 0.05 | |
| **HexCer(d18:2/16:0)** | 0.01 | 0.00 | 0.01 | 0.00 | > 0.05 | |  | | 0.01 | | | 0.00 | | 0.01 | 0.00 | | | > 0.05 | |
| **HexCer(d18:2/18:0)** | 0.03 | 0.00 | 0.03 | 0.00 | > 0.05 | |  | | 0.03 | | | 0.00 | | 0.02 | 0.00 | | | > 0.05 | |
| **HexCer(d18:2/20:0)** | 0.02 | 0.00 | 0.02 | 0.00 | > 0.05 | |  | | 0.02 | | | 0.00 | | 0.02 | 0.00 | | | > 0.05 | |
| **HexCer(d18:2/22:0)** | 0.21 | 0.02 | 0.20 | 0.02 | > 0.05 | |  | | 0.22 | | | 0.01 | | 0.21 | 0.01 | | | > 0.05 | |
| **HexCer(d18:2/23:0)** | 0.14 | 0.01 | 0.12 | 0.01 | > 0.05 | |  | | 0.13 | | | 0.01 | | 0.14 | 0.01 | | | > 0.05 | |
| **HexCer(d18:2/24:0)** | 0.18 | 0.01 | 0.17 | 0.02 | > 0.05 | |  | | 0.16 | | | 0.01 | | 0.16 | 0.01 | | | > 0.05 | |

**Supplementary Table S18.**Biochemical abbreviation and Plasma concentration (µM) of Hormones in low (LRFI) and high (HRFI) Residual Feed Intake bulls on Day 0 and 56 of the feed efficiency trial (Mean ± SEM).

|  |  |  | **Day 0** |  | |  | |  | |  |  | | | **Day 56** |  | | |  |  |
| --- | --- | --- | --- | --- | --- | --- | --- | --- | --- | --- | --- | --- | --- | --- | --- | --- | --- | --- | --- |
|  |  | **LRFI** |  | **HRFI** | |  | |  | |  | **LRFI** | | |  | **HRFI** | | |  |  |
| **COMPOUND** | **Mean** | **SEM** | **Mean** | **SEM** | | ***P*** | |  | | **Mean** | **SEM** | | | **Mean** | **SEM** | | | ***P*** |  |
| **AbsAcid** | 0.01 | 0.00 | 0.02 | 0.00 | > 0.05 | |  | | 0.02 | | | 0.00 | 0.02 | | | 0.00 | > 0.05 | | |
| **Cortisol** | 0.06 | 0.01 | 0.06 | 0.01 | > 0.05 | |  | | 0.06 | | | 0.01 | 0.06 | | | 0.01 | > 0.05 | | |
| **Cortisone** | 0.03 | 0.00 | 0.03 | 0.00 | > 0.05 | |  | | 0.03 | | | 0.00 | 0.04 | | | 0.00 | > 0.05 | | |
| **DHEAS** | 0.02 | 0.00 | 0.02 | 0.00 | > 0.05 | |  | | 0.02 | | | 0.00 | 0.02 | | | 0.00 | > 0.05 | | |

**Supplementary Table S19.**Biochemical abbreviation and Plasma concentration (µM) of Indoles in low (LRFI) and high (HRFI) Residual Feed Intake bulls on Day 0 and 56 of the feed efficiency trial (Mean ± SEM).

|  |  |  | **Day 0** |  | |  | |  | |  |  | | | **Day 56** | |  | |  |  |
| --- | --- | --- | --- | --- | --- | --- | --- | --- | --- | --- | --- | --- | --- | --- | --- | --- | --- | --- | --- |
|  |  | **LRFI** |  | **HRFI** | |  | |  | |  | **LRFI** | | |  | | **HRFI** | |  |  |
| **COMPOUND** | **Mean** | **SEM** | **Mean** | **SEM** | | ***P*** | |  | | **Mean** | **SEM** | | | **Mean** | | **SEM** | | ***P*** |  |
| **3-IAA** | 0.29 | 0.02 | 0.30 | 0.03 | > 0.05 | |  | | 0.29 | | | 0.02 | 0.30 | | 0.02 | | > 0.05 | | |
| **3-IPA** | 0.49 | 0.06 | 0.43 | 0.05 | > 0.05 | |  | | 0.41 | | | 0.06 | 0.44 | | 0.07 | | > 0.05 | | |
| **Ind-SO4** | 2.97 | 0.18 | 3.07 | 0.22 | > 0.05 | |  | | 2.59 | | | 0.20 | 2.69 | | 0.17 | | > 0.05 | | |
| **Indole** | 19.42 | 2.60 | 10.91 | 3.04 | 0.04 | |  | | 12.22 | | | 2.65 | 16.61 | | 4.09 | | > 0.05 | | |

**Supplementary Table S20.**Biochemical abbreviation and Plasma concentration (µM) of Nucleobases Related compounds in low (LRFI) and high (HRFI) Residual Feed Intake bulls on Day 0 and 56 of the feed efficiency trial (Mean ± SEM).

|  |  |  | **Day 0** |  | |  | |  | |  | |  | **Day 56** | |  | |  | |  |
| --- | --- | --- | --- | --- | --- | --- | --- | --- | --- | --- | --- | --- | --- | --- | --- | --- | --- | --- | --- |
|  |  | **LRFI** |  | **HRFI** | |  | |  | |  | | **LRFI** |  | | **HRFI** | |  | |  |
| **COMPOUND** | **Mean** | **SEM** | **Mean** | **SEM** | | ***P*** | |  | | **Mean** | | **SEM** | **Mean** | | **SEM** | | ***P*** | |  |
| **Hypoxanthine** | 0.02 | 0.00 | 0.02 | 0.00 | > 0.05 | |  | | 0.02 | | 0.00 | | | 0.02 | | 0.00 | | > 0.05 | |
| **Xanthine** | 0.07 | 0.00 | 0.07 | 0.00 | > 0.05 | |  | | 0.07 | | 0.00 | | | 0.07 | | 0.00 | | > 0.05 | |

**Supplementary Table S21.**Biochemical abbreviation and Plasma concentration (µM) of Sphingolipids in low (LRFI) and high (HRFI) Residual Feed Intake bulls on Day 0 and 56 of the feed efficiency trial (Mean ± SEM).

|  |  |  | **Day 0** |  | |  | |  | |  | |  | | **Day 56** | |  | |  | |
| --- | --- | --- | --- | --- | --- | --- | --- | --- | --- | --- | --- | --- | --- | --- | --- | --- | --- | --- | --- |
|  |  | **LRFI** |  | **HRFI** | |  | |  | |  | | **LRFI** | |  | | **HRFI** | |  | |
| **COMPOUND** | **Mean** | **SEM** | **Mean** | **SEM** | | ***P*** | |  | | **Mean** | | **SEM** | | **Mean** | | **SEM** | | ***P*** | |
| **SM (OH) C14:1** | 11.74 | 0.46 | 11.14 | 0.61 | > 0.05 | |  | | 11.88 | | 0.46 | | 10.61 | | 0.65 | | > 0.05 | |  |
| **SM (OH) C16:1** | 11.97 | 0.82 | 12.27 | 0.83 | > 0.05 | |  | | 12.12 | | 0.67 | | 11.72 | | 0.89 | | > 0.05 | |  |
| **SM (OH) C22:1** | 10.79 | 0.97 | 8.63 | 1.00 | > 0.05 | |  | | 10.84 | | 0.99 | | 8.87 | | 0.91 | | > 0.05 | |  |
| **SM (OH) C22:2** | 3.95 | 0.32 | 3.23 | 0.28 | > 0.05 | |  | | 4.09 | | 0.36 | | 3.32 | | 0.33 | | > 0.05 | |  |
| **SM (OH) C24:1** | 0.97 | 0.10 | 1.01 | 0.09 | > 0.05 | |  | | 0.94 | | 0.07 | | 0.94 | | 0.10 | | > 0.05 | |  |
| **SM C16:0** | 86.11 | 3.30 | 84.00 | 5.02 | > 0.05 | |  | | 91.34 | | 3.91 | | 81.37 | | 5.15 | | > 0.05 | |  |
| **SM C16:1** | 10.30 | 0.58 | 10.38 | 0.65 | > 0.05 | |  | | 11.10 | | 0.61 | | 10.01 | | 0.70 | | > 0.05 | |  |
| **SM C18:0** | 12.87 | 0.64 | 11.69 | 0.67 | > 0.05 | |  | | 13.83 | | 0.82 | | 11.97 | | 0.88 | | > 0.05 | |  |
| **SM C18:1** | 8.46 | 0.65 | 8.65 | 0.74 | > 0.05 | |  | | 8.65 | | 0.70 | | 8.13 | | 0.83 | | > 0.05 | |  |
| **SM C20:2** | 0.34 | 0.04 | 0.40 | 0.04 | > 0.05 | |  | | 0.32 | | 0.03 | | 0.36 | | 0.03 | | > 0.05 | |  |
| **SM C24:0** | 12.18 | 1.10 | 9.80 | 0.82 | > 0.05 | |  | | 11.85 | | 1.04 | | 10.37 | | 0.98 | | > 0.05 | |  |
| **SM C24:1** | 9.27 | 0.85 | 7.56 | 0.80 | > 0.05 | |  | | 9.02 | | 0.82 | | 7.04 | | 0.68 | | > 0.05 | |  |
| **SM C26:0** | 0.24 | 0.02 | 0.29 | 0.04 | > 0.05 | |  | | 0.26 | | 0.02 | | 0.26 | | 0.04 | | > 0.05 | |  |
| **SM C26:1** | 0.19 | 0.03 | 0.24 | 0.03 | > 0.05 | |  | | 0.26 | | 0.03 | | 0.28 | | 0.03 | | > 0.05 | |  |

**Supplementary Table S22.**Biochemical abbreviation and Plasma concentration (µM) of Sugars in low (LRFI) and high (HRFI) Residual Feed Intake bulls on Day 0 and 56 of the feed efficiency trial (Mean ± SEM).

|  |  |  | **Day 0** |  | |  | |  | |  | |  | | **Day 56** |  | | |  | |
| --- | --- | --- | --- | --- | --- | --- | --- | --- | --- | --- | --- | --- | --- | --- | --- | --- | --- | --- | --- |
|  |  | **LRFI** |  | **HRFI** | |  | |  | |  | | **LRFI** | |  | **HRFI** | | |  | |
| **COMPOUND** | **Mean** | **SEM** | **Mean** | **SEM** | | ***P*** | |  | | **Mean** | | **SEM** | | **Mean** | **SEM** | | | ***P*** | |
| **H1** | 2500.9 | 296.5 | 2491.9 | 305.4 | > 0.05 | |  | | 2349.4 | | 335.8 | | 2492.5 | | | 448.5 | > 0.05 | |  |

**Supplementary Table S23.**Biochemical abbreviation and Plasma concentration (µM) of Triacylglycerols in low (LRFI) and high (HRFI) Residual Feed Intake bulls on Day 0 and 56 of the feed efficiency trial (Mean ± SEM).

|  |  |  | **Day 0** |  | |  | |  | |  | |  | **Day 56** | | |  |  | | |
| --- | --- | --- | --- | --- | --- | --- | --- | --- | --- | --- | --- | --- | --- | --- | --- | --- | --- | --- | --- |
|  |  | **LRFI** |  | **HRFI** | |  | |  | |  | | **LRFI** |  | | | **HRFI** |  | | |
| **COMPOUND** | **Mean** | **SEM** | **Mean** | **SEM** | | ***P*** | |  | | **Mean** | | **SEM** | **Mean** | | | **SEM** | ***P*** | | |
| **TG(14:0_32:2)** | 0.71 | 0.08 | 0.71 | 0.08 | > 0.05 | |  | | 0.83 | | 0.09 | | | 0.94 | 0.12 | | | > 0.05 |  |
| **TG(14:0_34:0)** | 1.32 | 0.15 | 1.36 | 0.15 | > 0.05 | |  | | 1.62 | | 0.16 | | | 1.55 | 0.14 | | | > 0.05 |  |
| **TG(14:0_34:1)** | 1.77 | 0.23 | 1.84 | 0.20 | > 0.05 | |  | | 1.95 | | 0.27 | | | 2.15 | 0.27 | | | > 0.05 |  |
| **TG(14:0_34:2)** | 0.83 | 0.09 | 0.86 | 0.07 | > 0.05 | |  | | 1.00 | | 0.09 | | | 0.81 | 0.10 | | | > 0.05 |  |
| **TG(14:0_34:3)** | 0.09 | 0.03 | 0.07 | 0.04 | > 0.05 | |  | | 0.15 | | 0.06 | | | 0.28 | 0.07 | | | > 0.05 |  |
| **TG(14:0_35:1)** | 0.42 | 0.04 | 0.51 | 0.05 | > 0.05 | |  | | 0.57 | | 0.05 | | | 0.59 | 0.06 | | | > 0.05 |  |
| **TG(14:0_35:2)** | 0.34 | 0.04 | 0.33 | 0.03 | > 0.05 | |  | | 0.32 | | 0.04 | | | 0.30 | 0.03 | | | > 0.05 |  |
| **TG(14:0_36:1)** | 1.57 | 0.19 | 1.56 | 0.18 | > 0.05 | |  | | 1.94 | | 0.22 | | | 1.92 | 0.21 | | | > 0.05 |  |
| **TG(14:0_36:2)** | 1.33 | 0.14 | 1.37 | 0.17 | > 0.05 | |  | | 1.26 | | 0.15 | | | 1.49 | 0.16 | | | > 0.05 |  |
| **TG(14:0_36:3)** | 0.66 | 0.08 | 0.67 | 0.08 | > 0.05 | |  | | 0.67 | | 0.10 | | | 1.00 | 0.11 | | | > 0.05 |  |
| **TG(14:0_36:4)** | 0.59 | 0.06 | 0.53 | 0.06 | > 0.05 | |  | | 0.70 | | 0.08 | | | 0.59 | 0.07 | | | > 0.05 |  |
| **TG(14:0_38:4)** | 0.50 | 0.05 | 0.52 | 0.04 | > 0.05 | |  | | 0.57 | | 0.04 | | | 0.58 | 0.06 | | | > 0.05 |  |
| **TG(14:0_38:5)** | 0.59 | 0.21 | 0.51 | 0.24 | > 0.05 | |  | | 1.37 | | 0.36 | | | 1.61 | 0.35 | | | > 0.05 |  |
| **TG(14:0_40:5)** | 1.40 | 0.10 | 1.66 | 0.11 | > 0.05 | |  | | 1.55 | | 0.13 | | | 1.64 | 0.12 | | | > 0.05 |  |
| **TG(16:0_28:1)** | 0.28 | 0.03 | 0.30 | 0.04 | > 0.05 | |  | | 0.43 | | 0.05 | | | 0.40 | 0.04 | | | > 0.05 |  |
| **TG(16:0_28:2)** | 0.20 | 0.03 | 0.25 | 0.03 | > 0.05 | |  | | 0.24 | | 0.03 | | | 0.23 | 0.03 | | | > 0.05 |  |
| **TG(16:0_30:2)** | 0.30 | 0.04 | 0.37 | 0.03 | > 0.05 | |  | | 0.33 | | 0.04 | | | 0.41 | 0.07 | | | > 0.05 |  |
| **TG(16:0_32:0)** | 4.27 | 0.57 | 5.16 | 0.63 | > 0.05 | |  | | 5.69 | | 0.63 | | | 6.38 | 0.61 | | | > 0.05 |  |
| **TG(16:0_32:1)** | 2.42 | 0.24 | 2.55 | 0.27 | > 0.05 | |  | | 2.97 | | 0.34 | | | 3.11 | 0.33 | | | > 0.05 |  |
| **TG(16:0_32:2)** | 0.97 | 0.10 | 0.76 | 0.09 | > 0.05 | |  | | 1.25 | | 0.13 | | | 1.13 | 0.12 | | | > 0.05 |  |
| **TG(16:0_32:3)** | 0.13 | 0.05 | 0.13 | 0.05 | > 0.05 | |  | | 0.20 | | 0.05 | | | 0.09 | 0.04 | | | > 0.05 |  |
| **TG(16:0_33:1)** | 4.22 | 0.51 | 4.29 | 0.59 | > 0.05 | |  | | 5.08 | | 0.62 | | | 4.81 | 0.41 | | | > 0.05 |  |
| **TG(16:0_33:2)** | 1.44 | 0.19 | 1.22 | 0.14 | > 0.05 | |  | | 1.73 | | 0.21 | | | 1.98 | 0.22 | | | > 0.05 |  |
| **TG(16:0_34:0)** | 5.61 | 0.76 | 8.03 | 1.26 | > 0.05 | |  | | 7.13 | | 0.78 | | | 7.06 | 0.67 | | | > 0.05 |  |
| **TG(16:0_34:1)** | 11.82 | 1.57 | 15.47 | 2.08 | > 0.05 | |  | | 12.45 | | 1.78 | | | 13.77 | 1.68 | | | > 0.05 |  |
| **TG(16:0_34:2)** | 6.65 | 0.71 | 6.60 | 0.98 | > 0.05 | |  | | 6.96 | | 0.88 | | | 7.85 | 0.80 | | | > 0.05 |  |
| **TG(16:0_34:3)** | 1.68 | 0.21 | 1.75 | 0.26 | > 0.05 | |  | | 1.58 | | 0.20 | | | 1.60 | 0.22 | | | > 0.05 |  |
| **TG(16:0_34:4)** | 0.09 | 0.04 | 0.21 | 0.06 | > 0.05 | |  | | 0.16 | | 0.05 | | | 0.09 | 0.04 | | | > 0.05 |  |
| **TG(16:0_35:1)** | 3.11 | 0.34 | 3.46 | 0.48 | > 0.05 | |  | | 2.96 | | 0.36 | | | 3.42 | 0.34 | | | > 0.05 |  |
| **TG(16:0_35:2)** | 1.71 | 0.22 | 2.28 | 0.31 | > 0.05 | |  | | 1.96 | | 0.23 | | | 2.19 | 0.24 | | | > 0.05 |  |
| **TG(16:0_35:3)** | 0.68 | 0.08 | 0.61 | 0.06 | > 0.05 | |  | | 0.57 | | 0.06 | | | 0.62 | 0.06 | | | > 0.05 |  |
| **TG(16:0_36:2)** | 16.93 | 2.05 | 20.41 | 2.90 | > 0.05 | |  | | 20.64 | | 2.77 | | | 16.20 | 1.90 | | | > 0.05 |  |
| **TG(16:0_36:3)** | 9.12 | 1.31 | 8.85 | 1.39 | > 0.05 | |  | | 8.14 | | 1.18 | | | 8.05 | 1.01 | | | > 0.05 |  |
| **TG(16:0_36:4)** | 2.64 | 0.39 | 2.97 | 0.52 | > 0.05 | |  | | 3.11 | | 0.50 | | | 3.34 | 0.40 | | | > 0.05 |  |
| **TG(16:0_36:5)** | 0.84 | 0.14 | 0.86 | 0.18 | > 0.05 | |  | | 1.01 | | 0.17 | | | 0.87 | 0.17 | | | > 0.05 |  |
| **TG(16:0_36:6)** | 0.55 | 0.06 | 0.54 | 0.07 | > 0.05 | |  | | 0.54 | | 0.06 | | | 0.60 | 0.07 | | | > 0.05 |  |
| **TG(16:0_37:3)** | 0.33 | 0.04 | 0.43 | 0.05 | > 0.05 | |  | | 0.43 | | 0.06 | | | 0.45 | 0.04 | | | > 0.05 |  |
| **TG(16:0_38:1)** | 0.50 | 0.06 | 0.67 | 0.07 | > 0.05 | |  | | 0.58 | | 0.05 | | | 0.62 | 0.06 | | | > 0.05 |  |
| **TG(16:0_38:2)** | 0.53 | 0.07 | 0.49 | 0.07 | > 0.05 | |  | | 0.58 | | 0.07 | | | 0.66 | 0.07 | | | > 0.05 |  |
| **TG(16:0_38:3)** | 0.25 | 0.09 | 0.22 | 0.09 | > 0.05 | |  | | 0.30 | | 0.09 | | | 0.21 | 0.08 | | | > 0.05 |  |
| **TG(16:0_38:4)** | 0.62 | 0.09 | 0.68 | 0.11 | > 0.05 | |  | | 0.76 | | 0.11 | | | 0.71 | 0.11 | | | > 0.05 |  |
| **TG(16:0_38:5)** | 0.89 | 0.15 | 1.30 | 0.19 | > 0.05 | |  | | 1.28 | | 0.18 | | | 1.08 | 0.16 | | | > 0.05 |  |
| **TG(16:0_38:6)** | 0.54 | 0.10 | 0.69 | 0.12 | > 0.05 | |  | | 0.77 | | 0.11 | | | 0.73 | 0.10 | | | > 0.05 |  |
| **TG(16:0_38:7)** | 0.94 | 0.13 | 1.14 | 0.16 | > 0.05 | |  | | 1.11 | | 0.18 | | | 0.98 | 0.12 | | | > 0.05 |  |
| **TG(16:0_40:6)** | 0.45 | 0.06 | 0.38 | 0.05 | > 0.05 | |  | | 0.51 | | 0.05 | | | 0.57 | 0.09 | | | > 0.05 |  |
| **TG(16:0_40:7)** | 0.60 | 0.08 | 0.59 | 0.06 | > 0.05 | |  | | 0.48 | | 0.06 | | | 0.69 | 0.08 | | | > 0.05 |  |
| **TG(16:0_40:8)** | 0.44 | 0.06 | 0.45 | 0.04 | > 0.05 | |  | | 0.48 | | 0.05 | | | 0.50 | 0.05 | | | > 0.05 |  |
| **TG(16:1_28:0)** | 0.30 | 0.02 | 0.36 | 0.03 | > 0.05 | |  | | 0.31 | | 0.04 | | | 0.24 | 0.04 | | | > 0.05 |  |
| **TG(16:1_30:1)** | 0.27 | 0.03 | 0.27 | 0.03 | > 0.05 | |  | | 0.27 | | 0.04 | | | 0.28 | 0.04 | | | > 0.05 |  |
| **TG(16:1_32:0)** | 1.20 | 0.16 | 1.45 | 0.20 | > 0.05 | |  | | 1.60 | | 0.25 | | | 1.35 | 0.16 | | | > 0.05 |  |
| **TG(16:1_32:1)** | 0.38 | 0.12 | 0.57 | 0.13 | > 0.05 | |  | | 0.59 | | 0.11 | | | 0.60 | 0.13 | | | > 0.05 |  |
| **TG(16:1_32:2)** | 0.35 | 0.06 | 0.46 | 0.08 | > 0.05 | |  | | 0.41 | | 0.08 | | | 0.45 | 0.07 | | | > 0.05 |  |
| **TG(16:1_33:1)** | 0.57 | 0.09 | 0.72 | 0.09 | > 0.05 | |  | | 0.69 | | 0.09 | | | 0.66 | 0.11 | | | > 0.05 |  |
| **TG(16:1_34:0)** | 2.13 | 0.34 | 2.21 | 0.29 | > 0.05 | |  | | 2.58 | | 0.30 | | | 2.44 | 0.34 | | | > 0.05 |  |
| **TG(16:1_34:1)** | 2.80 | 0.34 | 2.41 | 0.30 | > 0.05 | |  | | 3.11 | | 0.41 | | | 2.77 | 0.42 | | | > 0.05 |  |
| **TG(16:1_34:2)** | 1.24 | 0.19 | 1.51 | 0.21 | > 0.05 | |  | | 1.53 | | 0.25 | | | 1.59 | 0.28 | | | > 0.05 |  |
| **TG(16:1_34:3)** | 0.47 | 0.06 | 0.53 | 0.09 | > 0.05 | |  | | 0.50 | | 0.09 | | | 0.53 | 0.09 | | | > 0.05 |  |
| **TG(16:1_36:1)** | 1.88 | 0.29 | 1.68 | 0.21 | > 0.05 | |  | | 2.33 | | 0.33 | | | 1.95 | 0.27 | | | > 0.05 |  |
| **TG(16:1_36:2)** | 1.77 | 0.27 | 1.85 | 0.26 | > 0.05 | |  | | 2.02 | | 0.29 | | | 2.45 | 0.39 | | | > 0.05 |  |
| **TG(16:1_36:3)** | 1.08 | 0.13 | 1.11 | 0.15 | > 0.05 | |  | | 1.33 | | 0.19 | | | 1.16 | 0.16 | | | > 0.05 |  |
| **TG(16:1_36:4)** | 0.22 | 0.08 | 0.39 | 0.15 | > 0.05 | |  | | 0.42 | | 0.14 | | | 0.45 | 0.15 | | | > 0.05 |  |
| **TG(16:1_36:5)** | 0.19 | 0.08 | 0.37 | 0.12 | > 0.05 | |  | | 0.56 | | 0.14 | | | 0.46 | 0.15 | | | > 0.05 |  |
| **TG(16:1_38:3)** | 0.23 | 0.02 | 0.25 | 0.02 | > 0.05 | |  | | 0.18 | | 0.02 | | | 0.23 | 0.02 | | | > 0.05 |  |
| **TG(16:1_38:4)** | 0.44 | 0.05 | 0.48 | 0.05 | > 0.05 | |  | | 0.53 | | 0.06 | | | 0.50 | 0.05 | | | > 0.05 |  |
| **TG(16:1_38:5)** | 0.43 | 0.19 | 0.78 | 0.25 | > 0.05 | |  | | 0.84 | | 0.23 | | | 0.91 | 0.26 | | | > 0.05 |  |
| **TG(17:0_32:1)** | 0.59 | 0.08 | 0.77 | 0.08 | > 0.05 | |  | | 0.69 | | 0.08 | | | 0.78 | 0.09 | | | > 0.05 |  |
| **TG(17:0_34:1)** | 3.01 | 0.41 | 3.11 | 0.34 | > 0.05 | |  | | 3.13 | | 0.31 | | | 3.05 | 0.38 | | | > 0.05 |  |
| **TG(17:0_34:2)** | 3.96 | 0.48 | 3.99 | 0.57 | > 0.05 | |  | | 4.85 | | 0.63 | | | 4.27 | 0.60 | | | > 0.05 |  |
| **TG(17:0_34:3)** | 0.80 | 0.15 | 0.99 | 0.17 | > 0.05 | |  | | 0.82 | | 0.14 | | | 1.07 | 0.15 | | | > 0.05 |  |
| **TG(17:0_36:3)** | 2.38 | 0.29 | 2.46 | 0.32 | > 0.05 | |  | | 2.21 | | 0.30 | | | 2.89 | 0.38 | | | > 0.05 |  |
| **TG(17:0_36:4)** | 0.59 | 0.10 | 0.58 | 0.09 | > 0.05 | |  | | 0.65 | | 0.08 | | | 0.64 | 0.09 | | | > 0.05 |  |
| **TG(17:1_32:1)** | 0.39 | 0.05 | 0.44 | 0.04 | > 0.05 | |  | | 0.40 | | 0.04 | | | 0.37 | 0.04 | | | > 0.05 |  |
| **TG(17:1_34:1)** | 0.93 | 0.11 | 0.98 | 0.12 | > 0.05 | |  | | 0.75 | | 0.09 | | | 0.89 | 0.11 | | | > 0.05 |  |
| **TG(17:1_34:2)** | 0.53 | 0.07 | 0.60 | 0.08 | > 0.05 | |  | | 0.49 | | 0.07 | | | 0.64 | 0.07 | | | > 0.05 |  |
| **TG(17:1_34:3)** | 0.15 | 0.05 | 0.16 | 0.06 | > 0.05 | |  | | 0.18 | | 0.06 | | | 0.19 | 0.05 | | | > 0.05 |  |
| **TG(17:1_36:3)** | 0.90 | 0.10 | 0.97 | 0.10 | > 0.05 | |  | | 0.68 | | 0.09 | | | 0.79 | 0.10 | | | > 0.05 |  |
| **TG(17:1_36:4)** | 0.54 | 0.17 | 0.41 | 0.15 | > 0.05 | |  | | 0.51 | | 0.16 | | | 0.58 | 0.14 | | | > 0.05 |  |
| **TG(17:1_36:5)** | 0.94 | 0.13 | 0.91 | 0.09 | > 0.05 | |  | | 0.98 | | 0.13 | | | 0.94 | 0.14 | | | > 0.05 |  |
| **TG(17:1_38:5)** | 0.07 | 0.01 | 0.07 | 0.01 | > 0.05 | |  | | 0.08 | | 0.01 | | | 0.08 | 0.01 | | | > 0.05 |  |
| **TG(17:1_38:6)** | 0.09 | 0.01 | 0.08 | 0.01 | > 0.05 | |  | | 0.09 | | 0.01 | | | 0.10 | 0.01 | | | > 0.05 |  |
| **TG(17:1_38:7)** | 0.11 | 0.01 | 0.11 | 0.01 | > 0.05 | |  | | 0.09 | | 0.01 | | | 0.11 | 0.01 | | | > 0.05 |  |
| **TG(17:2_34:2)** | 0.27 | 0.02 | 0.30 | 0.03 | > 0.05 | |  | | 0.27 | | 0.03 | | | 0.28 | 0.03 | | | > 0.05 |  |
| **TG(17:2_34:3)** | 0.82 | 0.07 | 0.73 | 0.07 | > 0.05 | |  | | 0.79 | | 0.08 | | | 0.91 | 0.07 | | | > 0.05 |  |
| **TG(17:2_36:2)** | 0.41 | 0.05 | 0.34 | 0.04 | > 0.05 | |  | | 0.30 | | 0.04 | | | 0.35 | 0.05 | | | > 0.05 |  |
| **TG(17:2_36:3)** | 0.83 | 0.10 | 0.71 | 0.08 | > 0.05 | |  | | 0.71 | | 0.10 | | | 0.74 | 0.10 | | | > 0.05 |  |
| **TG(17:2_36:4)** | 0.69 | 0.07 | 0.61 | 0.06 | > 0.05 | |  | | 0.54 | | 0.06 | | | 0.60 | 0.05 | | | > 0.05 |  |
| **TG(17:2_38:5)** | 0.59 | 0.06 | 0.55 | 0.07 | > 0.05 | |  | | 0.46 | | 0.05 | | | 0.60 | 0.06 | | | > 0.05 |  |
| **TG(17:2_38:6)** | 0.32 | 0.03 | 0.33 | 0.04 | > 0.05 | |  | | 0.28 | | 0.03 | | | 0.35 | 0.03 | | | > 0.05 |  |
| **TG(17:2_38:7)** | 0.05 | 0.01 | 0.05 | 0.01 | > 0.05 | |  | | 0.04 | | 0.01 | | | 0.04 | 0.01 | | | > 0.05 |  |
| **TG(18:0_30:0)** | 2.23 | 0.25 | 2.08 | 0.21 | > 0.05 | |  | | 2.43 | | 0.36 | | | 2.03 | 0.23 | | | > 0.05 |  |
| **TG(18:0_30:1)** | 0.69 | 0.10 | 0.64 | 0.08 | > 0.05 | |  | | 0.71 | | 0.10 | | | 0.77 | 0.09 | | | > 0.05 |  |
| **TG(18:0_32:0)** | 7.72 | 0.57 | 8.27 | 0.72 | > 0.05 | |  | | 7.40 | | 0.80 | | | 7.10 | 0.66 | | | > 0.05 |  |
| **TG(18:0_32:1)** | 3.82 | 0.32 | 3.52 | 0.39 | > 0.05 | |  | | 2.98 | | 0.38 | | | 3.91 | 0.42 | | | > 0.05 |  |
| **TG(18:0_32:2)** | 1.24 | 0.13 | 1.22 | 0.15 | > 0.05 | |  | | 1.29 | | 0.18 | | | 1.27 | 0.13 | | | > 0.05 |  |
| **TG(18:0_34:2)** | 11.49 | 1.11 | 10.61 | 1.00 | > 0.05 | |  | | 10.19 | | 1.15 | | | 12.12 | 1.22 | | | > 0.05 |  |
| **TG(18:0_34:3)** | 1.65 | 0.18 | 1.47 | 0.19 | > 0.05 | |  | | 1.67 | | 0.23 | | | 1.75 | 0.17 | | | > 0.05 |  |
| **TG(18:0_36:1)** | 11.92 | 1.64 | 10.89 | 1.56 | > 0.05 | |  | | 10.87 | | 1.46 | | | 12.69 | 1.32 | | | > 0.05 |  |
| **TG(18:0_36:2)** | 21.02 | 2.84 | 16.72 | 2.92 | > 0.05 | |  | | 20.49 | | 2.98 | | | 20.59 | 2.30 | | | > 0.05 |  |
| **TG(18:0_36:3)** | 7.30 | 0.91 | 6.94 | 1.10 | > 0.05 | |  | | 7.49 | | 1.14 | | | 8.89 | 0.95 | | | > 0.05 |  |
| **TG(18:0_36:4)** | 2.69 | 0.28 | 2.56 | 0.38 | > 0.05 | |  | | 2.48 | | 0.33 | | | 2.41 | 0.32 | | | > 0.05 |  |
| **TG(18:0_36:5)** | 1.07 | 0.34 | 0.54 | 0.25 | > 0.05 | |  | | 1.15 | | 0.34 | | | 1.77 | 0.38 | | | > 0.05 |  |
| **TG(18:0_38:6)** | 1.04 | 0.31 | 0.74 | 0.25 | > 0.05 | |  | | 1.19 | | 0.37 | | | 1.70 | 0.42 | | | > 0.05 |  |
| **TG(18:0_38:7)** | 1.01 | 0.33 | 0.83 | 0.28 | > 0.05 | |  | | 1.20 | | 0.33 | | | 1.77 | 0.51 | | | > 0.05 |  |
| **TG(18:1_26:0)** | 0.21 | 0.02 | 0.23 | 0.03 | > 0.05 | |  | | 0.23 | | 0.03 | | | 0.22 | 0.02 | | | > 0.05 |  |
| **TG(18:1_28:1)** | 0.44 | 0.06 | 0.51 | 0.06 | > 0.05 | |  | | 0.51 | | 0.05 | | | 0.56 | 0.06 | | | > 0.05 |  |
| **TG(18:1_30:0)** | 2.71 | 0.43 | 1.99 | 0.30 | > 0.05 | |  | | 2.23 | | 0.32 | | | 3.32 | 0.36 | | | 0.02 |  |
| **TG(18:1_30:1)** | 0.60 | 0.07 | 0.56 | 0.08 | > 0.05 | |  | | 0.70 | | 0.10 | | | 0.74 | 0.10 | | | > 0.05 |  |
| **TG(18:1_30:2)** | 0.38 | 0.05 | 0.40 | 0.05 | > 0.05 | |  | | 0.39 | | 0.05 | | | 0.48 | 0.05 | | | > 0.05 |  |
| **TG(18:1_31:0)** | 2.97 | 0.40 | 2.88 | 0.43 | > 0.05 | |  | | 2.90 | | 0.36 | | | 3.86 | 0.45 | | | > 0.05 |  |
| **TG(18:1_32:0)** | 11.12 | 1.62 | 9.05 | 1.39 | > 0.05 | |  | | 12.42 | | 1.78 | | | 14.24 | 1.66 | | | > 0.05 |  |
| **TG(18:1_32:1)** | 3.57 | 0.48 | 3.08 | 0.53 | > 0.05 | |  | | 4.29 | | 0.55 | | | 5.09 | 0.71 | | | > 0.05 |  |
| **TG(18:1_32:2)** | 1.02 | 0.13 | 0.80 | 0.11 | > 0.05 | |  | | 1.02 | | 0.15 | | | 1.11 | 0.13 | | | > 0.05 |  |
| **TG(18:1_32:3)** | 0.08 | 0.03 | 0.08 | 0.03 | > 0.05 | |  | | 0.11 | | 0.05 | | | 0.13 | 0.05 | | | > 0.05 |  |
| **TG(18:1_33:0)** | 8.18 | 1.30 | 8.19 | 1.23 | > 0.05 | |  | | 8.28 | | 1.13 | | | 10.05 | 1.23 | | | > 0.05 |  |
| **TG(18:1_33:1)** | 6.99 | 1.01 | 6.68 | 1.26 | > 0.05 | |  | | 7.64 | | 1.28 | | | 8.25 | 1.30 | | | > 0.05 |  |
| **TG(18:1_33:2)** | 1.55 | 0.23 | 1.91 | 0.30 | > 0.05 | |  | | 1.68 | | 0.24 | | | 1.83 | 0.24 | | | > 0.05 |  |
| **TG(18:1_33:3)** | 0.20 | 0.06 | 0.10 | 0.05 | > 0.05 | |  | | 0.15 | | 0.06 | | | 0.23 | 0.06 | | | > 0.05 |  |
| **TG(18:1_34:1)** | 29.14 | 4.40 | 23.49 | 4.11 | > 0.05 | |  | | 33.21 | | 5.15 | | | 32.35 | 4.65 | | | > 0.05 |  |
| **TG(18:1_34:2)** | 8.34 | 1.27 | 7.87 | 1.21 | > 0.05 | |  | | 10.84 | | 1.64 | | | 11.95 | 1.62 | | | > 0.05 |  |
| **TG(18:1_34:3)** | 1.68 | 0.23 | 1.55 | 0.23 | > 0.05 | |  | | 1.57 | | 0.24 | | | 2.11 | 0.32 | | | > 0.05 |  |
| **TG(18:1_34:4)** | 0.65 | 0.07 | 0.72 | 0.07 | > 0.05 | |  | | 0.76 | | 0.08 | | | 0.65 | 0.08 | | | > 0.05 |  |
| **TG(18:1_35:2)** | 1.13 | 0.32 | 1.03 | 0.33 | > 0.05 | |  | | 1.49 | | 0.40 | | | 1.97 | 0.40 | | | > 0.05 |  |
| **TG(18:1_35:3)** | 0.54 | 0.08 | 0.50 | 0.06 | > 0.05 | |  | | 0.61 | | 0.08 | | | 0.58 | 0.09 | | | > 0.05 |  |
| **TG(18:1_36:0)** | 8.97 | 1.39 | 6.73 | 0.93 | > 0.05 | |  | | 8.86 | | 1.19 | | | 10.67 | 1.33 | | | > 0.05 |  |
| **TG(18:1_36:1)** | 20.91 | 3.31 | 16.97 | 3.21 | > 0.05 | |  | | 26.05 | | 4.01 | | | 26.65 | 3.69 | | | > 0.05 |  |
| **TG(18:1_36:2)** | 13.36 | 1.93 | 12.91 | 2.33 | > 0.05 | |  | | 15.38 | | 2.45 | | | 18.80 | 2.76 | | | > 0.05 |  |
| **TG(18:1_36:3)** | 5.73 | 1.02 | 6.17 | 1.21 | > 0.05 | |  | | 6.56 | | 1.11 | | | 7.12 | 1.06 | | | > 0.05 |  |
| **TG(18:1_36:4)** | 2.45 | 0.29 | 1.99 | 0.28 | > 0.05 | |  | | 2.92 | | 0.32 | | | 3.39 | 0.35 | | | > 0.05 |  |
| **TG(18:1_36:5)** | 8.19 | 0.97 | 7.14 | 0.82 | > 0.05 | |  | | 6.94 | | 0.59 | | | 9.10 | 0.96 | | | > 0.05 |  |
| **TG(18:1_36:6)** | 0.46 | 0.13 | 0.28 | 0.09 | > 0.05 | |  | | 0.32 | | 0.14 | | | 0.61 | 0.17 | | | > 0.05 |  |
| **TG(18:1_38:5)** | 27.71 | 3.62 | 24.96 | 3.38 | > 0.05 | |  | | 25.89 | | 2.79 | | | 28.44 | 3.38 | | | > 0.05 |  |
| **TG(18:1_38:6)** | 4.63 | 0.75 | 4.76 | 0.61 | > 0.05 | |  | | 4.54 | | 0.47 | | | 6.11 | 0.92 | | | > 0.05 |  |
| **TG(18:1_38:7)** | 0.46 | 0.07 | 0.40 | 0.08 | > 0.05 | |  | | 0.38 | | 0.06 | | | 0.52 | 0.07 | | | > 0.05 |  |
| **TG(18:2_28:0)** | 0.32 | 0.05 | 0.36 | 0.03 | > 0.05 | |  | | 0.38 | | 0.04 | | | 0.48 | 0.05 | | | > 0.05 |  |
| **TG(18:2_30:0)** | 1.03 | 0.13 | 1.00 | 0.10 | > 0.05 | |  | | 1.08 | | 0.13 | | | 1.09 | 0.11 | | | > 0.05 |  |
| **TG(18:2_30:1)** | 0.35 | 0.05 | 0.40 | 0.04 | > 0.05 | |  | | 0.42 | | 0.05 | | | 0.43 | 0.05 | | | > 0.05 |  |
| **TG(18:2_31:0)** | 1.39 | 0.16 | 1.41 | 0.14 | > 0.05 | |  | | 1.67 | | 0.18 | | | 1.99 | 0.25 | | | > 0.05 |  |
| **TG(18:2_32:0)** | 4.02 | 0.50 | 3.92 | 0.34 | > 0.05 | |  | | 4.26 | | 0.40 | | | 4.26 | 0.35 | | | > 0.05 |  |
| **TG(18:2_32:1)** | 1.25 | 0.16 | 1.28 | 0.13 | > 0.05 | |  | | 1.41 | | 0.17 | | | 1.43 | 0.14 | | | > 0.05 |  |
| **TG(18:2_32:2)** | 0.53 | 0.06 | 0.55 | 0.06 | > 0.05 | |  | | 0.57 | | 0.06 | | | 0.61 | 0.07 | | | > 0.05 |  |
| **TG(18:2_33:0)** | 3.06 | 0.41 | 3.57 | 0.41 | > 0.05 | |  | | 3.15 | | 0.32 | | | 3.32 | 0.42 | | | > 0.05 |  |
| **TG(18:2_33:1)** | 1.43 | 0.20 | 1.59 | 0.18 | > 0.05 | |  | | 1.54 | | 0.18 | | | 2.00 | 0.25 | | | > 0.05 |  |
| **TG(18:2_33:2)** | 0.27 | 0.08 | 0.22 | 0.08 | > 0.05 | |  | | 0.18 | | 0.06 | | | 0.16 | 0.08 | | | > 0.05 |  |
| **TG(18:2_34:0)** | 8.04 | 0.96 | 7.54 | 0.74 | > 0.05 | |  | | 7.27 | | 0.71 | | | 8.56 | 0.93 | | | > 0.05 |  |
| **TG(18:2_34:1)** | 6.61 | 0.81 | 7.11 | 0.64 | > 0.05 | |  | | 7.15 | | 0.72 | | | 7.86 | 0.79 | | | > 0.05 |  |
| **TG(18:2_34:2)** | 3.08 | 0.38 | 3.68 | 0.44 | > 0.05 | |  | | 3.36 | | 0.36 | | | 4.82 | 0.63 | | | > 0.05 |  |
| **TG(18:2_34:3)** | 0.22 | 0.08 | 0.22 | 0.06 | > 0.05 | |  | | 0.31 | | 0.11 | | | 0.26 | 0.08 | | | > 0.05 |  |
| **TG(18:2_34:4)** | 0.28 | 0.03 | 0.31 | 0.03 | > 0.05 | |  | | 0.28 | | 0.03 | | | 0.33 | 0.04 | | | > 0.05 |  |
| **TG(18:2_35:1)** | 1.16 | 0.16 | 1.45 | 0.14 | > 0.05 | |  | | 1.25 | | 0.15 | | | 1.59 | 0.19 | | | > 0.05 |  |
| **TG(18:2_35:2)** | 0.29 | 0.11 | 0.38 | 0.12 | > 0.05 | |  | | 0.48 | | 0.15 | | | 0.43 | 0.15 | | | > 0.05 |  |
| **TG(18:2_35:3)** | 0.42 | 0.05 | 0.37 | 0.06 | > 0.05 | |  | | 0.36 | | 0.05 | | | 0.39 | 0.06 | | | > 0.05 |  |
| **TG(18:2_36:0)** | 3.15 | 0.41 | 2.95 | 0.36 | > 0.05 | |  | | 3.45 | | 0.41 | | | 3.64 | 0.34 | | | > 0.05 |  |
| **TG(18:2_36:1)** | 4.81 | 0.55 | 4.95 | 0.43 | > 0.05 | |  | | 5.09 | | 0.39 | | | 5.59 | 0.58 | | | > 0.05 |  |
| **TG(18:2_36:2)** | 4.55 | 0.62 | 5.03 | 0.49 | > 0.05 | |  | | 4.64 | | 0.45 | | | 5.36 | 0.58 | | | > 0.05 |  |
| **TG(18:2_36:3)** | 1.85 | 0.25 | 1.99 | 0.28 | > 0.05 | |  | | 2.24 | | 0.31 | | | 2.63 | 0.34 | | | > 0.05 |  |
| **TG(18:2_36:4)** | 1.00 | 0.18 | 1.30 | 0.20 | > 0.05 | |  | | 0.96 | | 0.12 | | | 1.17 | 0.16 | | | > 0.05 |  |
| **TG(18:2_36:5)** | 1.16 | 0.12 | 1.32 | 0.14 | > 0.05 | |  | | 1.14 | | 0.09 | | | 1.04 | 0.11 | | | > 0.05 |  |
| **TG(18:2_38:4)** | 1.26 | 0.15 | 1.37 | 0.16 | > 0.05 | |  | | 1.35 | | 0.17 | | | 1.40 | 0.19 | | | > 0.05 |  |
| **TG(18:2_38:5)** | 2.16 | 0.48 | 2.84 | 0.52 | > 0.05 | |  | | 2.15 | | 0.48 | | | 1.62 | 0.50 | | | > 0.05 |  |
| **TG(18:2_38:6)** | 0.76 | 0.09 | 0.77 | 0.08 | > 0.05 | |  | | 0.70 | | 0.10 | | | 0.67 | 0.08 | | | > 0.05 |  |
| **TG(18:3_30:0)** | 0.22 | 0.02 | 0.34 | 0.03 | 0.01 | |  | | 0.29 | | 0.03 | | | 0.30 | 0.04 | | | > 0.05 |  |
| **TG(18:3_32:0)** | 0.72 | 0.07 | 0.72 | 0.07 | > 0.05 | |  | | 0.76 | | 0.10 | | | 0.68 | 0.06 | | | > 0.05 |  |
| **TG(18:3_32:1)** | 0.15 | 0.04 | 0.23 | 0.06 | > 0.05 | |  | | 0.15 | | 0.05 | | | 0.10 | 0.04 | | | > 0.05 |  |
| **TG(18:3_33:2)** | 0.01 | 0.00 | 0.02 | 0.00 | > 0.05 | |  | | 0.01 | | 0.00 | | | 0.01 | 0.00 | | | > 0.05 |  |
| **TG(18:3_34:0)** | 1.57 | 0.18 | 1.32 | 0.14 | > 0.05 | |  | | 1.21 | | 0.16 | | | 1.42 | 0.14 | | | > 0.05 |  |
| **TG(18:3_34:1)** | 1.37 | 0.17 | 1.49 | 0.15 | > 0.05 | |  | | 1.47 | | 0.18 | | | 1.16 | 0.13 | | | > 0.05 |  |
| **TG(18:3_34:2)** | 0.66 | 0.09 | 0.72 | 0.09 | > 0.05 | |  | | 0.76 | | 0.08 | | | 0.78 | 0.08 | | | > 0.05 |  |
| **TG(18:3_34:3)** | 0.15 | 0.06 | 0.24 | 0.07 | > 0.05 | |  | | 0.13 | | 0.05 | | | 0.16 | 0.06 | | | > 0.05 |  |
| **TG(18:3_35:2)** | 0.05 | 0.02 | 0.07 | 0.02 | > 0.05 | |  | | 0.04 | | 0.01 | | | 0.04 | 0.01 | | | > 0.05 |  |
| **TG(18:3_36:1)** | 0.47 | 0.14 | 0.44 | 0.13 | > 0.05 | |  | | 0.37 | | 0.12 | | | 0.31 | 0.11 | | | > 0.05 |  |
| **TG(18:3_36:2)** | 0.76 | 0.09 | 1.01 | 0.11 | > 0.05 | |  | | 0.92 | | 0.09 | | | 0.94 | 0.10 | | | > 0.05 |  |
| **TG(18:3_36:3)** | 0.56 | 0.06 | 0.51 | 0.07 | > 0.05 | |  | | 0.49 | | 0.06 | | | 0.64 | 0.07 | | | > 0.05 |  |
| **TG(18:3_36:4)** | 0.75 | 0.08 | 1.18 | 0.17 | > 0.05 | |  | | 0.90 | | 0.11 | | | 0.85 | 0.12 | | | > 0.05 |  |
| **TG(18:3_38:5)** | 3.69 | 0.55 | 7.05 | 1.32 | > 0.05 | |  | | 5.89 | | 1.05 | | | 3.74 | 0.64 | | | > 0.05 |  |
| **TG(18:3_38:6)** | 0.08 | 0.01 | 0.17 | 0.04 | > 0.05 | |  | | 0.11 | | 0.02 | | | 0.11 | 0.03 | | | > 0.05 |  |
| **TG(20:0_32:3)** | 0.11 | 0.01 | 0.11 | 0.01 | > 0.05 | |  | | 0.10 | | 0.01 | | | 0.11 | 0.01 | | | > 0.05 |  |
| **TG(20:0_32:4)** | 0.08 | 0.02 | 0.11 | 0.03 | > 0.05 | |  | | 0.07 | | 0.03 | | | 0.09 | 0.03 | | | > 0.05 |  |
| **TG(20:0_34:1)** | 0.63 | 0.10 | 0.75 | 0.11 | > 0.05 | |  | | 0.54 | | 0.11 | | | 0.78 | 0.10 | | | > 0.05 |  |
| **TG(20:1_24:3)** | 0.13 | 0.02 | 0.16 | 0.03 | > 0.05 | |  | | 0.16 | | 0.03 | | | 0.15 | 0.03 | | | > 0.05 |  |
| **TG(20:1_26:1)** | 0.00 | 0.00 | 0.00 | 0.00 | > 0.05 | |  | | 0.00 | | 0.00 | | | 0.00 | 0.00 | | | > 0.05 |  |
| **TG(20:1_30:1)** | 0.22 | 0.01 | 0.22 | 0.02 | > 0.05 | |  | | 0.22 | | 0.02 | | | 0.22 | 0.01 | | | > 0.05 |  |
| **TG(20:1_32:0)** | 0.17 | 0.02 | 0.17 | 0.01 | > 0.05 | |  | | 0.20 | | 0.02 | | | 0.21 | 0.01 | | | > 0.05 |  |
| **TG(20:1_32:1)** | 0.17 | 0.05 | 0.11 | 0.05 | > 0.05 | |  | | 0.15 | | 0.05 | | | 0.26 | 0.06 | | | > 0.05 |  |
| **TG(20:1_32:2)** | 0.03 | 0.01 | 0.03 | 0.01 | > 0.05 | |  | | 0.03 | | 0.01 | | | 0.04 | 0.01 | | | > 0.05 |  |
| **TG(20:1_32:3)** | 0.03 | 0.01 | 0.02 | 0.00 | > 0.05 | |  | | 0.03 | | 0.00 | | | 0.03 | 0.01 | | | > 0.05 |  |
| **TG(20:1_34:0)** | 0.37 | 0.04 | 0.41 | 0.04 | > 0.05 | |  | | 0.45 | | 0.03 | | | 0.45 | 0.04 | | | > 0.05 |  |
| **TG(20:1_34:1)** | 0.42 | 0.05 | 0.49 | 0.06 | > 0.05 | |  | | 0.53 | | 0.06 | | | 0.63 | 0.06 | | | > 0.05 |  |
| **TG(20:1_34:2)** | 0.45 | 0.04 | 0.49 | 0.04 | > 0.05 | |  | | 0.46 | | 0.03 | | | 0.40 | 0.03 | | | > 0.05 |  |
| **TG(20:1_34:3)** | 0.04 | 0.01 | 0.03 | 0.01 | > 0.05 | |  | | 0.04 | | 0.01 | | | 0.06 | 0.01 | | | > 0.05 |  |
| **TG(20:2_32:0)** | 0.27 | 0.05 | 0.30 | 0.04 | > 0.05 | |  | | 0.28 | | 0.05 | | | 0.33 | 0.05 | | | > 0.05 |  |
| **TG(20:2_32:1)** | 0.04 | 0.01 | 0.02 | 0.01 | > 0.05 | |  | | 0.04 | | 0.01 | | | 0.05 | 0.01 | | | > 0.05 |  |
| **TG(20:2_34:1)** | 0.10 | 0.04 | 0.08 | 0.03 | > 0.05 | |  | | 0.09 | | 0.03 | | | 0.10 | 0.04 | | | > 0.05 |  |
| **TG(20:2_34:2)** | 0.33 | 0.09 | 0.13 | 0.06 | 0.01 | |  | | 0.12 | | 0.04 | | | 0.41 | 0.10 | | | 0.01 |  |
| **TG(20:2_34:3)** | 0.06 | 0.02 | 0.02 | 0.01 | > 0.05 | |  | | 0.03 | | 0.01 | | | 0.06 | 0.01 | | | > 0.05 |  |
| **TG(20:2_34:4)** | 0.05 | 0.01 | 0.01 | 0.01 | 0.01 | |  | | 0.03 | | 0.01 | | | 0.07 | 0.01 | | | > 0.05 |  |
| **TG(20:2_36:5)** | 0.22 | 0.02 | 0.24 | 0.02 | > 0.05 | |  | | 0.24 | | 0.02 | | | 0.20 | 0.02 | | | > 0.05 |  |
| **TG(20:3_32:0)** | 0.43 | 0.11 | 0.37 | 0.08 | > 0.05 | |  | | 0.30 | | 0.08 | | | 0.60 | 0.11 | | | > 0.05 |  |
| **TG(20:3_32:1)** | 0.15 | 0.02 | 0.14 | 0.01 | > 0.05 | |  | | 0.14 | | 0.02 | | | 0.16 | 0.02 | | | > 0.05 |  |
| **TG(20:3_32:2)** | 0.04 | 0.01 | 0.02 | 0.01 | > 0.05 | |  | | 0.03 | | 0.01 | | | 0.05 | 0.01 | | | > 0.05 |  |
| **TG(20:3_34:0)** | 0.60 | 0.08 | 0.63 | 0.06 | > 0.05 | |  | | 0.52 | | 0.06 | | | 0.56 | 0.07 | | | > 0.05 |  |
| **TG(20:3_34:1)** | 0.50 | 0.05 | 0.55 | 0.06 | > 0.05 | |  | | 0.51 | | 0.07 | | | 0.57 | 0.06 | | | > 0.05 |  |
| **TG(20:3_34:2)** | 0.63 | 0.07 | 0.57 | 0.06 | > 0.05 | |  | | 0.56 | | 0.07 | | | 0.63 | 0.10 | | | > 0.05 |  |
| **TG(20:3_34:3)** | 0.05 | 0.02 | 0.02 | 0.01 | > 0.05 | |  | | 0.05 | | 0.02 | | | 0.07 | 0.02 | | | > 0.05 |  |
| **TG(20:3_36:3)** | 0.60 | 0.07 | 0.72 | 0.06 | > 0.05 | |  | | 0.61 | | 0.06 | | | 0.79 | 0.09 | | | > 0.05 |  |
| **TG(20:3_36:4)** | 0.95 | 0.11 | 1.00 | 0.10 | > 0.05 | |  | | 1.06 | | 0.10 | | | 1.13 | 0.11 | | | > 0.05 |  |
| **TG(20:3_36:5)** | 0.05 | 0.01 | 0.04 | 0.01 | > 0.05 | |  | | 0.05 | | 0.01 | | | 0.08 | 0.01 | | | > 0.05 |  |
| **TG(20:4_30:0)** | 0.13 | 0.02 | 0.13 | 0.02 | > 0.05 | |  | | 0.12 | | 0.01 | | | 0.13 | 0.02 | | | > 0.05 |  |
| **TG(20:4_32:0)** | 0.31 | 0.04 | 0.35 | 0.04 | > 0.05 | |  | | 0.35 | | 0.04 | | | 0.35 | 0.03 | | | > 0.05 |  |
| **TG(20:4_32:1)** | 0.09 | 0.03 | 0.04 | 0.02 | > 0.05 | |  | | 0.09 | | 0.03 | | | 0.15 | 0.04 | | | > 0.05 |  |
| **TG(20:4_32:2)** | 0.12 | 0.03 | 0.07 | 0.03 | > 0.05 | |  | | 0.07 | | 0.02 | | | 0.12 | 0.03 | | | > 0.05 |  |
| **TG(20:4_33:2)** | 0.14 | 0.04 | 0.05 | 0.03 | > 0.05 | |  | | 0.07 | | 0.03 | | | 0.15 | 0.03 | | | > 0.05 |  |
| **TG(20:4_34:0)** | 0.13 | 0.05 | 0.07 | 0.03 | > 0.05 | |  | | 0.11 | | 0.04 | | | 0.31 | 0.09 | | | > 0.05 |  |
| **TG(20:4_34:1)** | 0.41 | 0.11 | 0.17 | 0.07 | > 0.05 | |  | | 0.21 | | 0.08 | | | 0.42 | 0.11 | | | > 0.05 |  |
| **TG(20:4_34:2)** | 1.40 | 0.18 | 1.05 | 0.15 | > 0.05 | |  | | 1.05 | | 0.12 | | | 1.50 | 0.17 | | | > 0.05 |  |
| **TG(20:4_34:3)** | 0.09 | 0.03 | 0.06 | 0.03 | > 0.05 | |  | | 0.09 | | 0.04 | | | 0.13 | 0.03 | | | > 0.05 |  |
| **TG(20:4_35:3)** | 0.06 | 0.00 | 0.06 | 0.00 | > 0.05 | |  | | 0.06 | | 0.01 | | | 0.05 | 0.01 | | | > 0.05 |  |
| **TG(20:4_36:2)** | 4.16 | 0.67 | 3.80 | 0.73 | > 0.05 | |  | | 4.53 | | 0.72 | | | 5.02 | 0.67 | | | > 0.05 |  |
| **TG(20:4_36:3)** | 0.87 | 0.12 | 0.91 | 0.12 | 0.03 | |  | | 0.85 | | 0.11 | | | 1.26 | 0.14 | | | 0.03 |  |
| **TG(20:4_36:4)** | 0.60 | 0.08 | 0.49 | 0.07 | > 0.05 | |  | | 0.57 | | 0.08 | | | 0.84 | 0.11 | | | > 0.05 |  |
| **TG(20:4_36:5)** | 0.42 | 0.13 | 0.29 | 0.12 | > 0.05 | |  | | 0.27 | | 0.10 | | | 0.58 | 0.13 | | | > 0.05 |  |
| **TG(20:5_34:0)** | 0.23 | 0.03 | 0.21 | 0.02 | > 0.05 | |  | | 0.21 | | 0.02 | | | 0.24 | 0.03 | | | > 0.05 |  |
| **TG(20:5_34:1)** | 0.39 | 0.06 | 0.45 | 0.06 | > 0.05 | |  | | 0.37 | | 0.05 | | | 0.41 | 0.05 | | | > 0.05 |  |
| **TG(20:5_34:2)** | 0.14 | 0.05 | 0.08 | 0.03 | > 0.05 | |  | | 0.10 | | 0.04 | | | 0.15 | 0.04 | | | > 0.05 |  |
| **TG(20:5_36:2)** | 0.47 | 0.06 | 0.48 | 0.04 | > 0.05 | |  | | 0.50 | | 0.06 | | | 0.52 | 0.06 | | | > 0.05 |  |
| **TG(20:5_36:3)** | 0.31 | 0.04 | 0.27 | 0.05 | 0.01 | |  | | 0.27 | | 0.03 | | | 0.42 | 0.05 | | | 0.01 |  |
| **TG(22:0_32:4)** | 0.06 | 0.01 | 0.04 | 0.01 | > 0.05 | |  | | 0.05 | | 0.01 | | | 0.06 | 0.01 | | | > 0.05 |  |
| **TG(22:1_32:5)** | 0.00 | 0.00 | 0.00 | 0.00 | > 0.05 | |  | | 0.00 | | 0.00 | | | 0.00 | 0.00 | | | > 0.05 |  |
| **TG(22:2_32:4)** | 0.01 | 0.00 | 0.01 | 0.00 | > 0.05 | |  | | 0.01 | | 0.00 | | | 0.02 | 0.00 | | | > 0.05 |  |
| **TG(22:3_30:2)** | 0.01 | 0.00 | 0.01 | 0.00 | > 0.05 | |  | | 0.01 | | 0.00 | | | 0.01 | 0.00 | | | > 0.05 |  |
| **TG(22:4_32:0)** | 0.20 | 0.02 | 0.16 | 0.02 | > 0.05 | |  | | 0.16 | | 0.02 | | | 0.18 | 0.02 | | | > 0.05 |  |
| **TG(22:4_32:2)** | 0.13 | 0.02 | 0.17 | 0.01 | > 0.05 | |  | | 0.17 | | 0.02 | | | 0.15 | 0.02 | | | > 0.05 |  |
| **TG(22:4_34:2)** | 0.40 | 0.05 | 0.38 | 0.04 | > 0.05 | |  | | 0.44 | | 0.05 | | | 0.50 | 0.05 | | | > 0.05 |  |
| **TG(22:5_32:0)** | 0.36 | 0.04 | 0.28 | 0.03 | > 0.05 | |  | | 0.35 | | 0.04 | | | 0.41 | 0.05 | | | > 0.05 |  |
| **TG(22:5_32:1)** | 0.16 | 0.02 | 0.17 | 0.02 | > 0.05 | |  | | 0.16 | | 0.02 | | | 0.19 | 0.02 | | | > 0.05 |  |
| **TG(22:5_34:1)** | 0.28 | 0.09 | 0.12 | 0.07 | > 0.05 | |  | | 0.20 | | 0.07 | | | 0.33 | 0.10 | | | > 0.05 |  |
| **TG(22:5_34:2)** | 0.32 | 0.11 | 0.17 | 0.10 | > 0.05 | |  | | 0.31 | | 0.10 | | | 0.57 | 0.13 | | | > 0.05 |  |
| **TG(22:5_34:3)** | 0.04 | 0.01 | 0.03 | 0.01 | > 0.05 | |  | | 0.03 | | 0.01 | | | 0.04 | 0.01 | | | > 0.05 |  |
| **TG(22:6_32:0)** | 0.08 | 0.02 | 0.12 | 0.04 | > 0.05 | |  | | 0.10 | | 0.03 | | | 0.15 | 0.04 | | | > 0.05 |  |
| **TG(22:6_32:1)** | 0.16 | 0.01 | 0.17 | 0.02 | > 0.05 | |  | | 0.17 | | 0.02 | | | 0.20 | 0.02 | | | > 0.05 |  |
| **TG(22:6_34:1)** | 0.49 | 0.06 | 0.50 | 0.06 | > 0.05 | |  | | 0.58 | | 0.06 | | | 0.58 | 0.06 | | | > 0.05 |  |
| **TG(22:6_34:2)** | 0.75 | 0.22 | 0.73 | 0.22 | > 0.05 | |  | | 0.58 | | 0.20 | | | 0.74 | 0.23 | | | > 0.05 |  |
| **TG(22:6_34:3)** | 0.23 | 0.02 | 0.26 | 0.02 | > 0.05 | |  | | 0.24 | | 0.03 | | | 0.25 | 0.02 | | | > 0.05 |  |

**Supplementary Table S24.**Biochemical abbreviation and Plasma concentration (µM) of Vitamins and Cofactors in low (LRFI) and high (HRFI) Residual Feed Intake bulls on Day 0 and 56 of the feed efficiency trial (Mean ± SEM).

|  |  |  | **Day 0** |  | |  | |  | |  | |  | **Day 56** | |  | | |  | |
| --- | --- | --- | --- | --- | --- | --- | --- | --- | --- | --- | --- | --- | --- | --- | --- | --- | --- | --- | --- |
|  |  | **LRFI** |  | **HRFI** | |  | |  | |  | | **LRFI** |  | | **HRFI** | | |  | |
| **COMPOUND** | **Mean** | **SEM** | **Mean** | **SEM** | | ***P*** | |  | | **Mean** | | **SEM** | **Mean** | | **SEM** | | | ***P*** | |
| **Choline** | 9.62 | 0.42 | 7.96 | 0.38 | 0.003 | |  | | 9.69 | | 0.39 | | | 8.97 | | 0.52 | > 0.05 | |  |

**Supplementary Table S25.**Biochemical abbreviation and Plasma concentration (nM) of Hormones (others) in low (LRFI) and high (HRFI) Residual Feed Intake bulls on Day 0 and 56 of the feed efficiency trial (Mean ± SEM).

|  |  |  | **Day 0** |  | |  | |  | |  | |  | | **Day 56** | |  | |  | |
| --- | --- | --- | --- | --- | --- | --- | --- | --- | --- | --- | --- | --- | --- | --- | --- | --- | --- | --- | --- |
|  |  | **LRFI** |  | **HRFI** | |  | |  | |  | | **LRFI** | |  | | **HRFI** | |  | |
| **COMPOUND** | **Mean** | **SEM** | **Mean** | **SEM** | | ***P*** | |  | | **Mean** | | **SEM** | | **Mean** | | **SEM** | | ***P*** | |
| **COR** | 32.70 | 3.62 | 28.88 | 3.16 | > 0.05 | |  | | 30.85 | | 3.29 | | 30.02 | | 2.97 | | > 0.05 | |  |
| **TEST** | 13.29 | 0.85 | 11.12 | 1.20 | > 0.05 | |  | | 11.71 | | 1.04 | | 11.75 | | 1.07 | | > 0.05 | |  |

**Supplementary Table S26.**Biochemical abbreviation and Plasma and RBC ratio (δ) of Isotopes in low (LRFI) and high (HRFI) Residual Feed Intake bulls on Day 0 and 56 of the feed efficiency trial (Mean ± SEM).

|  |  |  | **Day 0** |  | |  | |  | |  | |  | | **Day 56** |  | | |  |  |
| --- | --- | --- | --- | --- | --- | --- | --- | --- | --- | --- | --- | --- | --- | --- | --- | --- | --- | --- | --- |
|  |  | **LRFI** |  | **HRFI** | |  | |  | |  | | **LRFI** | |  | **HRFI** | | |  |  |
| **COMPOUND** | **Mean** | **SEM** | **Mean** | **SEM** | | ***P*** | |  | | **Mean** | | **SEM** | | **Mean** | **SEM** | | | ***P*** |  |
| **¹⁵N (Plasma)** | 6.04 | 0.14 | 6.07 | 0.09 | > 0.05 | |  | | 6.01 | | 0.12 | | 6.05 | | | 0.08 | > 0.05 | | |
| **¹³C (Plasma)** | -17.02 | 0.24 | -17.04 | 0.16 | > 0.05 | |  | | -17.31 | | 0.18 | | -17.38 | | | 0.10 | > 0.05 | | |
| **¹⁵N (RBC)** | 4.54 | 0.08 | 4.30 | 0.07 | > 0.05 | |  | | 4.80 | | 0.11 | | 4.69 | | | 0.06 | > 0.05 | | |
| **¹³C (RBC** | -25.02 | 0.09 | -25.92 | 0.08 | > 0.05 | |  | | -16.39 | | 0.27 | | -16.46 | | | 0.18 | > 0.05 | | |
