# Supplementary material for: Plasma biomarkers for residual feed intake prediction in beef bulls
Source: Transl Anim Sci. 2026 Feb 23;10:txag020. doi: 10.1093/tas/txag020 (PMC12986784; doi:10.1093/tas/txag020)
Supplement: txag020_Supplementary_Data [file txag020_supplementary_data.zip › Supplementary Table S1_Venturini et al. 2025.docx]

**Plasma Molecular Biomarkers for Residual Feed Intake Prediction in Beef Bulls**

Mauro Venturini, Daniella Heredia, Maria Camila López Duarte, Kamryn Joyce, Georgia Dubeux, Martin Ruiz-Moreno, Federico Tarnosky, Nadia Ashrafi, Stewart F. Graham, William W Thatcher, Jose Dubeux, Nicolas DiLorenzo, Ricardo Chebel, Joao Jabur Bittar, Angela Gonella

**Supplementary Table S1.** Biochemical name, abbreviation and PubChem Compound ID of each compound quantified in plasma samples of high and low Residual Feed Intake bulls on day 0 and 56.

| **Metabolite** | **Abbreviation** | **Bio ID** | **CID** |
| --- | --- | --- | --- |
| ***Acylcarnitines*** |  |  |  |
| L-Carnitine | C0 | HMDB0000062 | 281 |
| L-Acetylcarnitine | C2 | HMDB0000201 | 139247 |
| Propionylcarnitine | C3 | HMDB0000824 | 57449547 |
| Malonylcarnitine | C3-DC (C4-OH) | HMDB0002095 | 22833583 |
| Hydroxypropionylcarnitine | C3-OH | HMDB0013125 | 14795060 |
| Propenoylcarnitine | C3:1 | HMDB0013124 | 300 |
| Isobutyryl-L-carnitine | C4 | HMDB0000736 | 6432018 |
| Butenylcarnitine | C4:1 | HMDB0013126 | 6511 |
| 2-Methylbutyroylcarnitine | C5 | HMDB0000378 | 129675571 |
| Glutarylcarnitine | C5-DC (C6-OH) | HMDB0013130 | 71317118 |
| 3-Methylglutarylcarnitine | C5-M-DC | HMDB0000552 | 128145 |
| Hydroxyvalerylcarnitine | C5-OH (C3-DC-M) | HMDB0013132 | 118796870 |
| Tiglylcarnitine | C5:1 | HMDB0002366 | 638122 |
| Glutaconylcarnitine | C5:1-DC | HMDB0013129 | 53481620 |
| Hexanoylcarnitine | C6 (C4:1-DC) | HMDB0000705 | 6426853 |
| 2-Hexenoylcarnitine | C6:1 | HMDB0013161 | 5282707 |
| Pimelylcarnitine | C7-DC | HMDB0013328 | 91825640 |
| Octanoylcarnitine | C8 | HMDB0000791 | 71332814 |
| 2,6 Dimethylheptanoyl carnitine | C9 | HMDB0006320 | 53477823 |
| Decanoylcarnitine | C10 | HMDB0000651 | 71336865 |
| 9-Decenoylcarnitine | C10:1 | HMDB0013205 | 5282724 |
| 2-trans,4-cis-Decadienoylcarnitine | C10:2 | HMDB0013325 | 101087484 |
| Dodecanoylcarnitine | C12 | HMDB0002250 | 85668474 |
| Dodecanedioylcarnitine | C12-DC | HMDB0013327 | 53481673 |
| trans-2-Dodecenoylcarnitine | C12:1 | HMDB0013326 | 5282729 |
| Tetradecanoylcarnitine | C14 | HMDB0005066 | 101087485 |
| cis-5-Tetradecenoylcarnitine | C14:1 | HMDB0002014 | 88490686 |
| 3-Hydroxy-cis-5-tetradecenoylcarnitine | C14:1-OH | HMDB0013330 | 53481777 |
| 3, 5-Tetradecadiencarnitine | C14:2 | HMDB0013331 | 91749539 |
|  | C14:2-OH |  |  |
| Palmitoylcarnitine | C16 | HMDB0000222 | 71345955 |
| 3-Hydroxyhexadecanoylcarnitine | C16-OH | HMDB0013336 | 71464553 |
| trans-Hexadec-2-enoyl carnitine | C16:1 | HMDB0006317 | 11680418 |
| 3-Hydroxy-9-hexadecenoylcarnitine | C16:1-OH | HMDB0013333 | 53481779 |
| 9,12-Hexadecadienoylcarnitine | C16:2 | HMDB0013334 | 91749542 |
| 3-Hydroxyhexadecadienoylcarnitine | C16:2-OH | HMDB0013335 | 53481689 |
| Stearoylcarnitine | C18 | HMDB0000848 | 14940714 |
| Oleoylcarnitine | C18:1 | HMDB0005065 | 445639 |
| 3-Hydroxy-11Z-octadecenoylcarnitine | C18:1-OH | HMDB0013339 | 156908021 |
| Linoelaidylcarnitine | C18:2 | HMDB0006461 | 5280450 |
| ***Alkaloids*** |  |  |  |
| Trigonelline | Trigonelline | HMDB0000875 | 5570 |
| ***Amine Oxides*** |  |  |  |
| Trimethylamine N-oxide | TMAO | HMDB0000925 | 1145 |
| ***Aminoacids*** |  |  |  |
| Alanine | Ala | HMDB0000161 | 5950 |
| L-arginine | Arg | HMDB0000517 | 6322 |
| L-asparagine | Asn | HMDB0000168 | 6267 |
| L-aspartic acid | Asp | HMDB0000191 | 5960 |
| L-cysteine | Cys | HMDB0000574 | 582 |
| L-glutamine | Gln | HMDB0000641 | 5961 |
| L-glutamic acid | Glu | HMDB0000148 | 33032 |
| Glycine | Gly | HMDB0000123 | 750 |
| L-histidine | His | HMDB0000177 | 6274 |
| L-Isoleucine | Ile | HMDB0000172 | 16130199 |
| L-leucine | Leu | HMDB0000687 | 6106 |
| L-Lysine | Lys | HMDB0000182 | 5962 |
| L-methionine | Met | HMDB0000696 | 6137 |
| L-phenylalanine | Phe | HMDB0000159 | 6140 |
| L-Proline | Pro | HMDB0000162 | 145742 |
| L-Serine | Ser | HMDB0000187 | 5951 |
| L-serine | Thr | HMDB0000167 | 6288 |
| L-tryptophan | Trp | HMDB0000929 | 6305 |
| L-tyrosine | Tyr | HMDB0000158 | 6057 |
| L-valine | Val | HMDB0000883 | 6287 |
| ***Aminoacids Related*** |  |  |  |
| 1-Methylhistidine | 1-Met-His | HMDB0000001 | 92105 |
| Methylhistidine | 3-Met-His | HMDB0000479 | 90638 |
| 5-Aminopentanoic acid | 5-AVA | HMDB0003355 | 100955340 |
| L-alpha-Aminobutyric acid | AABA | HMDB0000452 | 6657 |
| N2-Acetylornithine | Ac-Orn | HMDB0003357 | 439232 |
| Asymmetric dimethylarginine | ADMA | HMDB0001539 | 123831 |
| Aminoadipic acid | alpha-AAA | HMDB0000510 | 469 |
| Anserine | Anserine | HMDB0000194 | 112072 |
| 3-Aminobutanoic acid | BABA | HMDB0031654 | 10932 |
| Betaine | Betaine | HMDB0000043 | 247 |
| cis-4-Hydroxyproline | c4-OH-Pro | HMDB0240251 | 440015 |
| Carnosine | Carnosine | HMDB0000033 | 439224 |
| Citrulline | Cit | HMDB0000904 | 10048368 |
| Creatinine | Creatinine | HMDB0000562 | 588 |
| L-Cystine | Cystine | HMDB0000192 | 67678 |
| DOPA | DOPA | HMDB0000181 | 6047 |
| Homo-L-arginine | HArg | HMDB0000670 | 232 |
| Homocysteine | HCys | HMDB0000742 | 594 |
| Kynurenine | Kynurenine | HMDB0000684 | 846 |
| Methionine sulfoxide | Met-SO | HMDB0002005 | 158980 |
| 3-Nitrotyrosine | Nitro-Tyr | HMDB0001904 | 91867833 |
| Ornithine | Orn | HMDB0000214 | 25246315 |
| Phenylacetylglycine | PAG | HMDB0000821 | 16132166 |
| Phenylalanine betaine | PheAlaBetaine | HMDB0240552 |  |
| Proline betaine | ProBetaine | HMDB0004827 | 115244 |
| Sarcosine | Sarcosine | HMDB0000271 | 1088 |
| Symmetric dimethylarginine | SDMA | HMDB0003334 | 169148 |
| 4-Hydroxyproline | t4-OH-Pro | HMDB0000725 | 825 |
| Taurine | Taurine | HMDB0000251 |  |
| Lenticin | TrpBetaine | HMDB0061115 | 1123 |
| ***Bile Acids*** |  |  |  |
| Cholic acid | CA | HMDB0000619 | 5460341 |
| Chenodeoxycholic acid | CDCA | HMDB0000518 | 10133 |
| Deoxycholic acid | DCA | HMDB0000626 | 517326 |
| Glycocholic acid | GCA | HMDB0000138 | 16197348 |
| Chenodeoxycholic acid glycine conjugate | GCDCA | HMDB0000637 | 12544 |
| Deoxycholic acid glycine conjugate | GDCA | HMDB0000631 | 3035026 |
| Lithocholic acid glycine conjugate | GLCA | HMDB0000698 | 94715 |
| Sulfolithocholylglycine | GLCAS | HMDB0002639 | 72222 |
| Glycoursodeoxycholic acid | GUDCA | HMDB0000708 | 12310288 |
| Taurocholic acid | TCA | HMDB0000036 | 6421 |
| Taurochenodesoxycholic acid | TCDCA | HMDB0000951 | 387316 |
| Taurodeoxycholic acid | TDCA | HMDB0000896 | 137701658 |
| Lithocholyltaurine | TLCA | HMDB0000722 | 439763 |
| Tauro-b-muricholic acid | TMCA | HMDB0000932 | 16746649 |
| ***Biogenic Amines*** |  |  |  |
| beta-Alanine | beta-Ala | HMDB0000056 | 239 |
| Dopamine | Dopamine | HMDB0000073 | 681 |
| gamma-Aminobutyric acid | GABA | HMDB0000112 | 119 |
| Histamine | Histamine | HMDB0000870 | 774 |
| Phenylethylamine | PEA | HMDB0012275 | 6054 |
| Putrescine | Putrescine | HMDB0001414 | 1045 |
| Serotonin | Serotonin | HMDB0000259 | 5202 |
| Spermidine | Spermidine | HMDB0001257 | 1102 |
| Spermine | Spermine | HMDB0001256 | 1103 |
| ***Carboxylic Acids*** |  |  |  |
| cis-Aconitic acid | AconAcid | HMDB0000072 | 643757 |
| Dodecanedioic acid | DiCA(12:0) | HMDB0000623 | 12736 |
| Tetradecanedioic acid | DiCA(14:0) | HMDB0000872 | 13185 |
| Hippuric acid | HipAcid | HMDB0000714 | 464 |
| Lactic acid | Lac | HMDB0000190 | 62358 |
| 3-Hydroxyglutaric acid | OH-GlutAcid | HMDB0000428 | 33032 |
| Succinic acid | Suc | HMDB0000254 | 1110 |
| ***Ceramides*** |  |  |  |
|  | Cer(d16:1/18:0) | HMDB0341516 | 10582678 |
|  | Cer(d16:1/20:0) | HMDB0341517 | 70698937 |
| Ceramide (D16:1/22:0) | Cer(d16:1/22:0) | HMDB0240682 | 10627306 |
| Sphingomyelin(D16:1/23:0) | Cer(d16:1/23:0) | HMDB0240619 | 11273482 |
|  | Cer(d16:1/24:0) | HMDB0341518 | 10627700 |
| Ceramide (d18:1/14:0) | Cer(d18:1/14:0) | HMDB0011773 | 5282310 |
| Ceramide (d18:1/16:0) | Cer(d18:1/16:0) | HMDB0004949 | 5283564 |
|  | Cer(d18:1/18:0(OH)) |  |  |
| Ceramide (d18:1/18:0) | Cer(d18:1/18:0) | HMDB0004950 | 5283565 |
| Ceramide (d18:1/18:1) | Cer(d18:1/18:1) | HMDB0004948 | 154573065 |
|  | Cer(d18:1/20:0(OH)) |  |  |
| Ceramide (d18:1/20:0) | Cer(d18:1/20:0) | HMDB0004951 | 5283566 |
| Ceramide (d18:1/22:0) | Cer(d18:1/22:0) | HMDB0004952 | 5283567 |
| Ceramide (d18:1/23:0) | Cer(d18:1/23:0) | HMDB0000950 | 52931115 |
| Ceramide (d18:1/24:0) | Cer(d18:1/24:0) | HMDB0004956 | 5283571 |
| Ceramide (d18:1/24:1) | Cer(d18:1/24:1) | HMDB0004953 | 90659794 |
| Ceramide (d18:1/25:0) | Cer(d18:1/25:0) | HMDB0004957 | 9547202 |
| Ceramide (d18:1/26:0) | Cer(d18:1/26:0) | HMDB0004955 | 5283570 |
| Ceramide (d18:1/26:1) | Cer(d18:1/26:1) | HMDB04954 | 154573078 |
|  | Cer(d18:2/14:0) | HMDB0341546 | 52931116 |
|  | Cer(d18:2/16:0) |  | 52931118 |
|  | Cer(d18:2/18:0) |  | 132282053 |
|  | Cer(d18:2/18:1) | HMDB0341547 | 52931119 |
|  | Cer(d18:2/20:0) |  | 52931120 |
|  | Cer(d18:2/22:0) | HMDB0341519 | 52931123 |
|  | Cer(d18:2/23:0) |  | 52931124 |
|  | Cer(d18:2/24:0) | HMDB0341520 | 165415641 |
| Ceramide (d18:2/24:1) | Cer(d18:2/24:1) | HMDB0240680 | 156960926 |
| ***Cholesterol Esters*** |  |  |  |
| 1-Myristoyl-cholesterol | CE(14:0) | HMDB0006725 | 99486 |
| cholest-5-en-3b-yl (9Z-tetradecenoate) | CE(14:1) | HMDB0062458 | 24779601 |
| Cholesterol 1-pentadecanoate | CE(15:0) | HMDB0060057 | 24779606 |
| 1-Pentadecanoyl-cholesterol | CE(15:1) | HMDB0060056 | 124202106 |
| (3beta)-Cholest-5-en-3-ol hexadecanoate | CE(16:0) | HMDB0000885 | 246520 |
| 1-Palmitoleoyl-cholesterol | CE(16:1) | HMDB0000658 | 13828666 |
| Cholesteryl 1-heptadecanoic acid | CE(17:0) | HMDB0060059 | 24779605 |
| cholesteryl (9Z)-heptadecenoate | CE(17:1) | HMDB0060060 | 24779603 |
| Cholesterol stearate | CE(18:0) | HMDB0062461 | 118246 |
| Cholesteroyl-oleate | CE(18:1) | HMDB0000918 | 5283632 |
| Cholesteryl 1-linoleoate | CE(18:2) | HMDB0000610 | 5287939 |
| Cholesteryl 1-g-linolenoate | CE(18:3) | HMDB0010369 | 6436907 |
| Cholest-5-en-3b-yl eicosanoate | CE(20:0) | HMDB0062459 | 16061336 |
| Cholesteryl eicosenoate | CE(20:1) | HMDB0005193 | 16061337 |
| Cholesteryl eicosatrienoate | CE(20:3) | HMDB0006736 | 14274976 |
| Cholesterol arachidonate | CE(20:4) | HMDB0006726 | 6479222 |
| Cholesteryl eicosapentaenoate | CE(20:5) | HMDB0006731 | 156997831 |
| Cholesteryl behenate | CE(22:0) | HMDB0006727 | 16061339 |
| cholest-5-en-3b-yl (13Z-docosenoate) | CE(22:1) | HMDB0062456 | 16219158 |
| Cholesteryl docosadienoate | CE(22:2) | HMDB0006737 | 16061340 |
| Cholesteryl 1-osbondoate | CE(22:5) | HMDB0010374 | 42608400 |
| Cholesteryl docosahexaenoate | CE(22:6) | HMDB0006733 | 14274978 |
| ***Cresols*** |  |  |  |
| p-Cresol sulfate | p-Cresol-SO4 | HMDB0011635 | 4615423 |
| ***Diacylglycerols*** |  |  |  |
| Diacylglycerol(28:0) | DG(14:0_14:0) | HMDB0007008 | 89298 |
| Diacylglycerol(32:1) | DG(14:0_18:1) | HMDB0007015 | 14275341 |
| Diacylglycerol(32:2) | DG(14:0_18:2) | HMDB0007016 | 14275352 |
| Diacylglycerol(34:0) | DG(14:0_20:0) | HMDB0007020 | 53477955 |
| Diacylglycerol(32:2) | DG(14:1_18:1) | HMDB0007044 | 53477979 |
| Diacylglycerol(34:3) | DG(14:1_20:2) | HMDB0007051 | 53477986 |
| Diacylglycerol(32:0) | DG(16:0_16:0) | HMDB0007098 | 99931 |
| Diacylglycerol(32:1) | DG(16:0_16:1) | HMDB0007099 | 9543678 |
| Diacylglycerol(34:1) | DG(16:0_18:1) | HMDB0007101 | 6440177 |
| Diacylglycerol(34:2) | DG(16:0_18:2) | HMDB0007103 | 9543695 |
| Diacylglycerol(36:0) | DG(16:0_20:0) | HMDB0007107 | 9543710 |
| Diacylglycerol(36:3) | DG(16:0_20:3) | HMDB0007111 | 9543728 |
| Diacylglycerol(36:4) | DG(16:0_20:4) | HMDB0007112 | 9543736 |
| Diacylglycerol(34:1) | DG(16:1_18:0) | HMDB0007129 | 9543691 |
| Diacylglycerol(34:2) | DG(16:1_18:1) | HMDB0007131 | 9543694 |
| Diacylglycerol(34:3) | DG(16:1_18:2) | HMDB0007132 | 9543699 |
| DG(17:0/17:1/0:0)[iso2] | DG(17:0_17:1) |  | 9543686 |
| DG(17:0/18:1/0:0)[iso2] | DG(17:0_18:1) |  | 9543698 |
| Diacylglycerol(38:4) | DG(18:0_20:4) | HMDB0007170 | 6438587 |
| Diacylglycerol(36:2) | DG(18:1_18:1) | HMDB0007188 | 9543716 |
| Diacylglycerol(36:3) | DG(18:1_18:2) | HMDB0007219 | 9543722 |
| Diacylglycerol(36:4) | DG(18:1_18:3) | HMDB0007221 | 14275405 |
| Diacylglycerol(36:5) | DG(18:1_18:4) | HMDB0007334 | 53478163 |
| Diacylglycerol(38:2) | DG(18:1_20:1) | HMDB0007224 | 9543756 |
| Diacylglycerol(38:3) | DG(18:1_20:2) | HMDB0007225 | 9543766 |
| Diacylglycerol(38:4) | DG(18:1_20:3) | HMDB0007227 | 9543776 |
| Diacylglycerol(38:5) | DG(18:1_20:4) | HMDB0007228 | 9543786 |
| Diacylglycerol(40:6) | DG(18:1_22:5) | HMDB0007236 | 9543880 |
| Diacylglycerol(40:7) | DG(18:1_22:6) | HMDB0007237 | 9543890 |
| Diacylglycerol(36:4) | DG(18:2_18:2) | HMDB0007248 | 6438297 |
| Diacylglycerol(36:5) | DG(18:2_18:3) | HMDB0007250 | 14275407 |
| Diacylglycerol(36:6) | DG(18:2_18:4) | HMDB0007335 | 53478164 |
| Diacylglycerol(38:2) | DG(18:2_20:0) | HMDB0007252 | 9543755 |
| Diacylglycerol(38:6) | DG(18:2_20:4) | HMDB0007257 | 9543796 |
| Diacylglycerol(36:6) | DG(18:3_18:3) | HMDB0007278 | 53478121 |
| Diacylglycerol(38:5) | DG(18:3_20:2) | HMDB0007283 | 9543784 |
| DG(21:0/22:6/0:0)[iso2] | DG(21:0_22:6) |  | 9543955 |
| Diacylglycerol(44:3) | DG(22:1_22:2) | HMDB0007639 | 9543948 |
|  | DG-O(14:0_18:2) |  | 164232790 |
|  | DG-O(16:0_20:4) |  | 164504764 |
|  | DG-O(18:2_18:2) |  | 164358162 |
| ***Dihydroceramides*** |  |  |  |
| C16-Dihydroceramide | Cer(d18:0/16:0) | HMDB0011760 | 5283572 |
|  | Cer(d18:0/18:0(OH)) |  |  |
| C18-(Dihydro)ceramide | Cer(d18:0/18:0) | HMDB0011761 | 5283573 |
| N-Icosanoylsphinganine | Cer(d18:0/20:0) | HMDB0011764 | 5283574 |
| N-Docosanoyldihydrosphingosine | Cer(d18:0/22:0) | HMDB0011765 | 5283575 |
| N-(Tetracosanoyl)-dihydrosphingosine | Cer(d18:0/24:0) | HMDB0011768 | 5283577 |
| N-Nervonoylsphinganine | Cer(d18:0/24:1) | HMDB0011769 | 154573074 |
|  | Cer(d18:0/26:1(OH)) |  |  |
| ***Fatty Acids*** |  |  |  |
| Arachidonic acid | AA | HMDB0001043 | 243 |
| Docosahexaenoic acid | DHA | HMDB0002183 | 15608515 |
| Eicosapentaenoic acid | EPA | HMDB0001999 | 446284 |
| Dodecanoic acid | FA(12:0) | HMDB0000638 | 164344202 |
| Myristic acid | FA(14:0) | HMDB0000806 | 73761743 |
| Palmitic acid | FA(16:0) | HMDB0000220 | 165184182 |
| Stearic acid | FA(18:0) | HMDB0000827 | 165210552 |
| Elaidic acid | FA(18:1) | HMDB0000573 | 53481446 |
| Linoleic acid | FA(18:2) | HMDB0000673 | 6440740 |
| 11Z-Eicosenoic acid | FA(20:1) | HMDB0002231 | 131839773 |
| Eicosadienoic acid | FA(20:2) | HMDB0005060 | 52921831 |
| Dihomo-gamma-linolenic acid | FA(20:3) | HMDB0002925 | 164257565 |
| ***Glycerophospholipids*** |  |  |  |
| LysoPC(14:0/0:0) | lysoPC a C14:0 | HMDB0010379 | 460604 |
| LysoPC(16:0/0:0) | lysoPC a C16:0 | HMDB0010382 | 460602 |
| LysoPC(16:1/0:0) | lysoPC a C16:1 | HMDB0010383 | 24779461 |
| LysoPC(17:0/0:0) | lysoPC a C17:0 | HMDB0012108 | 24779463 |
| LysoPC(18:0/0:0) | lysoPC a C18:0 | HMDB0010384 | 497299 |
| LysoPC(18:1/0:0) | lysoPC a C18:1 | HMDB0002815 | 16081932 |
| LysoPC(18:2/0:0) | lysoPC a C18:2 | HMDB0010386 | 11005824 |
| LysoPC(20:3/0:0) | lysoPC a C20:3 | HMDB0010393 | 52924055 |
| LysoPC(20:4/0:0) | lysoPC a C20:4 | HMDB0010395 | 53480469 |
| LysoPC(24:0/0:0) | lysoPC a C24:0 | HMDB0010405 | 24779481 |
| LysoPC(26:0/0:0) | lysoPC a C26:0 | HMDB0029205 | 131750810 |
| LysoPC(26:1/0:0) | lysoPC a C26:1 | HMDB0029220 | 131750814 |
| LysoPC(28:0/0:0) | lysoPC a C28:0 | HMDB0029206 | 131750811 |
| LysoPC(28:1/0:0) | lysoPC a C28:1 | HMDB0029221 | 131750816 |
| Phosphatidylcholine diacyl C24:0 | PC aa C24:0 |  | 24779481 |
| Phosphatidylcholine diacyl C26:0 | PC aa C26:0 |  |  |
| Phosphatidylcholine diacyl C28:1 | PC aa C28:1 | HMDB0007867 | 52922244 |
| Phosphatidylcholine diacyl C30:0 | PC aa C30:0 | HMDB0007869 | 53478615 |
| Phosphatidylcholine diacyl C30:0 | PC aa C32:0 | HMDB0000564 | 53478671 |
| Phosphatidylcholine diacyl C32:1 | PC aa C32:1 | HMDB0007872 | 53478619 |
| Phosphatidylcholine diacyl C32:2 | PC aa C32:2 | HMDB0007874 | 24778624 |
| Phosphatidylcholine diacyl C32:3 | PC aa C32:3 | HMDB0007876 | 52922220 |
| Phosphatidylcholine diacyl C34:1 | PC aa C34:1 | HMDB0007971 | 24778688 |
| Phosphatidylcholine diacyl C34:2 | PC aa C34:2 | HMDB0007973 | 52922228 |
| Phosphatidylcholine diacyl C34:3 | PC aa C34:3 | HMDB0007974 | 52922230 |
| Phosphatidylcholine diacyl C34:4 | PC aa C34:4 | HMDB0007883 | 24778634 |
| Phosphatidylcholine diacyl C36:0 | PC aa C36:0 | HMDB0007886 | 53478705 |
| Phosphatidylcholine diacyl C36:1 | PC aa C36:1 | HMDB0007978 | 53478609 |
| Phosphatidylcholine diacyl C36:2 | PC aa C36:2 | HMDB0007979 | 52922236 |
| Phosphatidylcholine diacyl C36:3 | PC aa C36:3 | HMDB0007980 | 53478825 |
| Phosphatidylcholine diacyl C36:4 | PC aa C36:4 | HMDB0007982 | 52922238 |
| Phosphatidylcholine diacyl C36:5 | PC aa C36:5 | HMDB0007984 | 53478611 |
| Phosphatidylcholine diacyl C36:6 | PC aa C36:6 | HMDB0007892 | 24778639 |
| Phosphatidylcholine diacyl C38:0 | PC aa C38:0 | HMDB0007893 | 53479157 |
| Phosphatidylcholine diacyl C38:3 | PC aa C38:3 | HMDB0008020 | 53478953 |
| Phosphatidylcholine diacyl C38:4 | PC aa C38:4 | HMDB0007988 | 53479267 |
| Phosphatidylcholine diacyl C38:5 | PC aa C38:5 | HMDB0007989 | 53479399 |
| Phosphatidylcholine diacyl C38:6 | PC aa C38:6 | HMDB0007991 | 6441886 |
| Phosphatidylcholine diacyl C40:1 | PC aa C40:1 | HMDB0007993 | 24778745 |
| Phosphatidylcholine diacyl C40:2 | PC aa C40:2 | HMDB0008276 | 52922667 |
| Phosphatidylcholine diacyl C40:3 | PC aa C40:3 | HMDB0008086 | 52922811 |
| Phosphatidylcholine diacyl C40:4 | PC aa C40:4 | HMDB0008054 | 52922931 |
| Phosphatidylcholine diacyl C40:5 | PC aa C40:5 | HMDB0008055 | 24778871 |
| Phosphatidylcholine diacyl C40:6 | PC aa C40:6 | HMDB0008057 | 24778876 |
| Phosphatidylcholine diacyl C42:0 | PC aa C42:0 | HMDB0008058 | 53479481 |
| Phosphatidylcholine diacyl C42:1 | PC aa C42:1 | HMDB0008059 | 24778881 |
| Phosphatidylcholine diacyl C42:2 | PC aa C42:2 | HMDB0008092 | 53478795 |
| Phosphatidylcholine diacyl C42:4 | PC aa C42:4 | HMDB0008191 | 53478867 |
| Phosphatidylcholine diacyl C42:5 | PC aa C42:5 | HMDB0008287 | 53478883 |
| Phosphatidylcholine diacyl C42:6 | PC aa C42:6 | HMDB0008288 | 24779053 |
| Phosphatidylcholine diacyl C30:0 | PC ae C30:0 | HMDB0013341 | 24779275 |
| Phosphatidylcholine diacyl C30:1 | PC ae C30:1 | HMDB0013402 | 24779277 |
| Phosphatidylcholine diacyl C30:2 | PC ae C30:2 | HMDB0013410 | 53481703 |
| Phosphatidylcholine diacyl C32:1 | PC ae C32:1 | HMDB0007896 | 52923742 |
| Phosphatidylcholine diacyl C32:2 | PC ae C32:2 | HMDB0013411 | 53481705 |
| Phosphatidylcholine diacyl C34:0 | PC ae C34:0 | HMDB0013405 | 11803170 |
| Phosphatidylcholine diacyl C34:1 | PC ae C34:1 | HMDB0013426 | 53481707 |
| Phosphatidylcholine diacyl C34:2 | PC ae C34:2 | HMDB0011151 | 6443157 |
| Phosphatidylcholine diacyl C34:3 | PC ae C34:3 | HMDB0013413 | 53481709 |
| Phosphatidylcholine diacyl C36:0 | PC ae C36:0 | HMDB0013406 | 24779326 |
| Phosphatidylcholine diacyl C36:1 | PC ae C36:1 | HMDB0013414 | 53481711 |
| Phosphatidylcholine diacyl C36:2 | PC ae C36:2 | HMDB0011243 | 6443070 |
| Phosphatidylcholine diacyl C36:3 | PC ae C36:3 | HMDB0013425 | 53481721 |
| Phosphatidylcholine diacyl C36:4 | PC ae C36:4 | HMDB0013407 | 53481701 |
| Phosphatidylcholine diacyl C36:5 | PC ae C36:5 | HMDB0011220 | 53481713 |
| Phosphatidylcholine diacyl C38:0 | PC ae C38:0 | HMDB0013408 | 24779301 |
| Phosphatidylcholine diacyl C38:1 | PC ae C38:1 | HMDB0013408 | 53481727 |
| Phosphatidylcholine diacyl C38:2 | PC ae C38:2 | HMDB0013416 | 53481729 |
| Phosphatidylcholine diacyl C38:3 | PC ae C38:3 | HMDB0013431 | 52923834 |
| Phosphatidylcholine diacyl C38:4 | PC ae C38:4 | HMDB0013420 | 6443065 |
| Phosphatidylcholine diacyl C38:5 | PC ae C38:5 | HMDB0011253 | 24779379 |
| Phosphatidylcholine diacyl C38:6 | PC ae C38:6 | HMDB0013409 | 16759366 |
| Phosphatidylcholine diacyl C40:1 | PC ae C40:1 | HMDB0013433 | 53481731 |
| Phosphatidylcholine diacyl C40:2 | PC ae C40:2 | HMDB0013437 | 53481739 |
| Phosphatidylcholine diacyl C40:3 | PC ae C40:3 | HMDB0013445 | 53481745 |
| Phosphatidylcholine diacyl C40:4 | PC ae C40:4 | HMDB0013442 | 52923852 |
| Phosphatidylcholine diacyl C40:4 | PC ae C40:5 | HMDB0013444 | 53481743 |
| Phosphatidylcholine diacyl C40:5 | PC ae C40:6 | HMDB0013422 | 24779341 |
| Phosphatidylcholine diacyl C42:0 | PC ae C42:0 | HMDB0013423 | 53481717 |
| Phosphatidylcholine diacyl C42:1 | PC ae C42:1 | HMDB0013434 | 53481733 |
| Phosphatidylcholine diacyl C42:2 | PC ae C42:2 | HMDB0013438 | 53481741 |
| Phosphatidylcholine diacyl C42:3 | PC ae C42:3 | HMDB0013458 | 53481773 |
| Phosphatidylcholine diacyl C42:4 | PC ae C42:4 | HMDB0013448 | 53481751 |
| Phosphatidylcholine diacyl C42:5 | PC ae C42:5 | HMDB0013451 | 53481757 |
| Phosphatidylcholine diacyl C44:3 | PC ae C44:3 | HMDB0013449 | 53481759 |
| Phosphatidylcholine diacyl C44:4 | PC ae C44:4 | HMDB0013453 | 53481775 |
| Phosphatidylcholine diacyl C44:5 | PC ae C44:5 | HMDB0013456 | 53481767 |
| Phosphatidylcholine diacyl C44:6 | PC ae C44:6 | HMDB0013450 | 53481755 |
| ***Glycosylceramides*** |  |  |  |
| lactosylceramide (d18:1/14:0) | Hex2Cer(d18:1/14:0) | HMDB0012097 | 118796895 |
| Galabiosylceramide (d18:1/16:0) | Hex2Cer(d18:1/16:0) | HMDB0004833 | 20057274 |
| Galabiosylceramide (d18:1/18:0) | Hex2Cer(d18:1/18:0) | HMDB0004834 | 20057275 |
| Galabiosylceramide (d18:1/20:0) | Hex2Cer(d18:1/20:0) | HMDB0004835 | 20057276 |
| Galabiosylceramide (d18:1/22:0) | Hex2Cer(d18:1/22:0) | HMDB0004836 | 20057277 |
| Galabiosylceramide (d18:1/24:0) | Hex2Cer(d18:1/24:0) | HMDB0004840 | 20057281 |
| Galabiosylceramide (d18:1/24:1(15Z)) | Hex2Cer(d18:1/24:1) | HMDB0004837 | 20057278 |
| Galabiosylceramide (d18:1/26:0) | Hex2Cer(d18:1/26:0) | HMDB0004839 | 20057280 |
| Galabiosylceramide (d18:1/26:1(17Z)) | Hex2Cer(d18:1/26:1) | HMDB0004838 | 20057279 |
| Trihexosylceramide (d18:1/16:0) | Hex3Cer(d18:1/16:0) | HMDB0004879 | 20057274 |
| Trihexosylceramide (d18:1/18:0) | Hex3Cer(d18:1/18:0) | HMDB0004880 | 20057275 |
| Trihexosylceramide (d18:1/24:1(15Z)) | Hex3Cer(d18:1/24:1) | HMDB0004883 | 20057278 |
| Trihexosylceramide (d18:1/26:1(17Z)) | Hex3Cer(d18:1/26:1) | HMDB0004884 | 20057279 |
| Trihexosylceramide (d18:1/20:0) | Hex3Cer(d18:1_20:0) | HMDB0004881 | 20057316 |
| Trihexosylceramide (d18:1/22:0) | Hex3Cer(d18:1_22:0) | HMDB0004882 | 20057317 |
|  | HexCer(d16:1/22:0) | HMDB0341523 |  |
|  | HexCer(d16:1/24:0) | HMDB0341544 |  |
| Galactosylceramide (d18:1/14:0) | HexCer(d18:1/14:0) | HMDB0012321 | 53481406 |
| GlcCer(d18:1/16:0) | HexCer(d18:1/16:0) | HMDB0004971 | 53480652 |
| GlcCer(d18:1/18:0) | HexCer(d18:1/18:0) | HMDB0004972 | 53480653 |
| GlcCer(d18:1/9Z-18:1) | HexCer(d18:1/18:1) | HMDB0004970 | 53480658 |
| GlcCer(d18:1/20:0) | HexCer(d18:1/20:0) | HMDB0004973 | 53480654 |
| GlcCer(d18:1/22:0) | HexCer(d18:1/22:0) | HMDB0004974 | 53480655 |
|  | HexCer(d18:1/23:0) | HMDB0341524 |  |
| GlcCer(d18:1/24:0) | HexCer(d18:1/24:0) | HMDB0004978 | 6451121 |
| GlcCer(d18:1/24:1(15Z)) | HexCer(d18:1/24:1) | HMDB0004975 | 53480656 |
| GlcCer(d18:1/26:0) | HexCer(d18:1/26:0) | HMDB0004977 |  |
| GlcCer(d18:1/26:1(17Z)) | HexCer(d18:1/26:1) | HMDB0004976 | 20057358 |
| GalCer(d18:2/16:0) | HexCer(d18:2/16:0) | HMDB0341525 |  |
|  | HexCer(d18:2/18:0) | HMDB0341526 |  |
|  | HexCer(d18:2/20:0) | HMDB0341527 |  |
|  | HexCer(d18:2/22:0) | HMDB0341528 | 138206856 |
|  | HexCer(d18:2/23:0) | HMDB0341529 |  |
|  | HexCer(d18:2/24:0) | HMDB0341530 |  |
| ***Hormones*** |  |  |  |
| (S)-Abscisic acid | AbsAcid | HMDB0035140 | 5702609 |
| Cortisol | Cortisol | HMDB0000063 | 5754 |
| Cortisone | Cortisone | HMDB0002802 | 222786 |
| Dehydroepiandrosterone sulfate | DHEAS | HMDB0001032 | 12594 |
| ***Indoles Derivatives*** |  |  |  |
| Indoleacetic acid | 3-IAA | HMDB0000197 | 802 |
| Indole-3-propionic acid | 3-IPA | HMDB0002302 | 521106 |
| Indoxyl sulfate | Ind-SO4 | HMDB0000682 | 10258 |
| Indole | Indole | HMDB0000738 | 798 |
| ***Nucleobases Related*** |  |  |  |
| Hypoxanthine | Hypoxanthine | HMDB0000157 | 135398638 |
| Xanthine | Xanthine | HMDB0000292 | 1188 |
| ***Sphingolipids*** |  |  |  |
| Hydroxysphingomyeline C14:1 | SM (OH) C14:1 | HMDB0013462 | 53481777 |
| Hydroxysphingomyeline C14:1 | SM (OH) C16:1 | HMDB0013463 | 53481779 |
| Hydroxysphingomyeline C22:1 | SM (OH) C22:1 | HMDB0013466 | 53481785 |
| Hydroxysphingomyeline C22:2 | SM (OH) C22:2 | HMDB0013467 | 53481787 |
| Hydroxysphingomyeline C24:1 | SM (OH) C24:1 | HMDB0013469 | 53481791 |
| Sphingomyelin (d18:1/16:0) | SM C16:0 | HMDB0010169 | 9939941 |
|  | SM C16:1 | HMDB0029216 | 52931145 |
| Sphingomyelin (D18:1/18:0) | SM C18:0 | HMDB0001348 | 6453725 |
| Sphingomyelin (d18:1/18:1) | SM C18:1 | HMDB0012100 | 156998407 |
|  | SM C20:2 |  | 252149946 |
| Sphingomyelin (d18:1/24:0) | SM C24:0 | HMDB0011697 | 44260127 |
| Sphingomyelin (d18:1/24:1) | SM C24:1 | HMDB0012107 | 53481791 |
| Sphingomyelin (d18:1/26:0) | SM C26:0 | HMDB0011698 | 44260129 |
| Sphingomyelin (d18:1/26:1) | SM C26:1 | HMDB0013461 | 44260128 |
| ***Sugars*** |  |  |  |
| D-Glucose | H1 | HMDB0000122 | 5793 |
| ***Triacylglycerols*** |  |  |  |
| Tracylglycerol(46:2) | TG(14:0_32:2) | HMDB0042280 | 131753422 |
| Tracylglycerol(48:0) | TG(14:0_34:0) | HMDB0063486 | 56936565 |
| Tracylglycerol(48:1) | TG(14:0_34:1) | HMDB0042275 | 131756481 |
| Tracylglycerol(48:2) | TG(14:0_34:2) | HMDB0042078 | 131753224 |
| Tracylglycerol(48:3) | TG(14:0_34:3) | HMDB0042073 | 131753219 |
| Tracylglycerol(49:1) | TG(14:0_35:1) | HMDB0042102 | 56937971 |
| Tracylglycerol(49:2) | TG(14:0_35:2) | HMDB0042108 |  |
| Tracylglycerol(50:1) | TG(14:0_36:1) | HMDB0042074 | 131753220 |
| Tracylglycerol(50:2) | TG(14:0_36:2) | HMDB0042081 | 56936574 |
| Tracylglycerol(50:3) | TG(14:0_36:3) | HMDB0042140 | 56938026 |
| Tracylglycerol(50:4) | TG(14:0_36:4) | HMDB0042082 | 56936576 |
| Tracylglycerol(52:4) | TG(14:0_38:4) | HMDB0042142 | 56938033 |
| Tracylglycerol(52:5) | TG(14:0_38:5) | HMDB0042177 | 56938142 |
|  | TG(14:0_40:5) |  |  |
| Tracylglycerol(44:1) | TG(16:0_28:1) | HMDB0043834 |  |
| Tracylglycerol(44:2) | TG(16:0_28:2) | HMDB0044030 | 56936609 |
| TAG(46:1) | TG(16:0_30:2) | HMDB0042996 | 56936667 |
| Tracylglycerol(48:0) | TG(16:0_32:0) | HMDB0005356 | 11147 |
| Tracylglycerol(48:1) | TG(16:0_32:1) | HMDB0005359 | 9543986 |
| Tracylglycerol(48:2) | TG(16:0_32:2) | HMDB0005376 | 9543987 |
| Tracylglycerol(48:3) | TG(16:0_32:3) | HMDB0047778 | 56938422 |
| Tracylglycerol(49:1) | TG(16:0_33:1) | HMDB0043864 |  |
| Tracylglycerol(49:2) | TG(16:0_33:2) | HMDB0043870 |  |
| Tracylglycerol(50:0) | TG(16:0_34:0) | HMDB0063117 | 56938022 |
| Tracylglycerol(50:1) | TG(16:0_34:1) | HMDB0005360 | 56938428 |
| Tracylglycerol(50:2) | TG(16:0_34:2) | HMDB0005362 | 25240356 |
| Tracylglycerol(50:3) | TG(16:0_34:3) | HMDB0005379 | 9544021 |
| Tracylglycerol(50:4) | TG(16:0_34:4) | HMDB0043907 | 56936774 |
| Tracylglycerol(51:1) | TG(16:0_35:1) | HMDB0043028 | 56938807 |
| Tracylglycerol(51:2) | TG(16:0_35:2) | HMDB0011700 | 53481032 |
| Tracylglycerol(51:3) | TG(16:0_35:3) | HMDB0011701 | 53481033 |
| Tracylglycerol(52:2) | TG(16:0_36:2) | HMDB0005369 | 9544069 |
| Tracylglycerol(52:3) | TG(16:0_36:3) | HMDB0005384 | 25240361 |
| Tracylglycerol(52:4) | TG(16:0_36:4) | HMDB0005363 | 9544125 |
| Tracylglycerol(52:5) | TG(16:0_36:5) | HMDB0005380 | 9544150 |
| Tracylglycerol(52:6) | TG(16:0_36:6) | HMDB0042149 | 56938035 |
|  | TG(16:0_37:3) |  |  |
| Tracylglycerol(54:1) | TG(16:0_38:1) | HMDB0005368 | 9544170 |
| Tracylglycerol(54:2) | TG(16:0_38:2) | HMDB0005383 | 9544199 |
| Tracylglycerol(54:3) | TG(16:0_38:3) | HMDB0005389 | 9544231 |
| Tracylglycerol(54:4) | TG(16:0_38:4) | HMDB0005370 | 9544279 |
| Tracylglycerol(54:5) | TG(16:0_38:5) | HMDB0005385 | 9544319 |
| Tracylglycerol(54:6) | TG(16:0_38:6) | HMDB0005391 | 9544362 |
| Tracylglycerol(53:0) | TG(16:0_38:7) | HMDB0063700 | 9544077 |
| Tracylglycerol(56:6) | TG(16:0_40:6) | HMDB0043939 | 9544814 |
| Tracylglycerol(56:7) | TG(16:0_40:7) | HMDB0044135 | 9544886 |
| Tracylglycerol(56:8) | TG(16:0_40:8) | HMDB0005392 | 9544762 |
| Tracylglycerol(44:1) | TG(16:1_28:0) | HMDB0042069 | 56936554 |
| Tracylglycerol(46:2) | TG(16:1_30:1) | HMDB0047771 | 56938416 |
| Tracylglycerol(48:1) | TG(16:1_32:0) | HMDB0005359 | 9543986 |
| Tracylglycerol(48:2) | TG(16:1_32:1) | HMDB0005376 | 9543987 |
| Tracylglycerol(48:3) | TG(16:1_32:2) | HMDB0005432 | 9543989 |
|  | TG(16:1_33:1) |  |  |
| Tracylglycerol(50:1) | TG(16:1_34:0) | HMDB0005374 | 9544004 |
| Tracylglycerol(50:2) | TG(16:1_34:1) | HMDB0005377 | 9544011 |
| Tracylglycerol(50:3) | TG(16:1_34:2) | HMDB0005379 | 9544021 |
| Tracylglycerol(50:4) | TG(16:1_34:3) | HMDB0005435 | 131750394 |
| Tracylglycerol(52:2) | TG(16:1_36:1) | HMDB0005378 | 9544079 |
| Tracylglycerol(52:3) | TG(16:1_36:2) | HMDB0005425 | 9544087 |
| Tracylglycerol(52:4) | TG(16:1_36:3) | HMDB0005440 | 25240364 |
| Tracylglycerol(52:5) | TG(16:1_36:4) | HMDB0005380 | 9544150 |
| Tracylglycerol(52:6) | TG(16:1_36:5) | HMDB0005436 | 9544179 |
| Tracylglycerol(54:4) | TG(16:1_38:3) | HMDB0005445 | 25240372 |
| Tracylglycerol(54:5) | TG(16:1_38:4) | HMDB0005426 | 9544320 |
| Tracylglycerol(54:6) | TG(16:1_38:5) | HMDB0005441 | 9544363 |
|  | TG(17:0_32:1) |  |  |
|  | TG(17:0_34:1) |  |  |
|  | TG(17:0_34:2) |  |  |
|  | TG(17:0_34:3) |  |  |
|  | TG(17:0_36:3) |  |  |
|  | TG(17:0_36:4) |  |  |
|  | TG(17:1_32:1) |  |  |
|  | TG(17:1_34:1) |  |  |
|  | TG(17:1_34:2) |  |  |
|  | TG(17:1_34:3) |  |  |
|  | TG(17:1_36:3) |  |  |
|  | TG(17:1_36:4) |  |  |
|  | TG(17:1_36:5) |  |  |
|  | TG(17:1_38:5) |  |  |
|  | TG(17:1_38:6) |  |  |
|  | TG(17:1_38:7) |  |  |
|  | TG(17:2_34:2) |  |  |
|  | TG(17:2_34:3) |  |  |
|  | TG(17:2_36:2) |  |  |
|  | TG(17:2_36:3) |  |  |
|  | TG(17:2_36:4) |  |  |
|  | TG(17:2_38:5) |  |  |
|  | TG(17:2_38:6) |  |  |
|  | TG(17:2_38:7) |  |  |
| Tracylglycerol(48:0) | TG(18:0_30:0) | HMDB0108000 | 91865742 |
| Tracylglycerol(48:1) | TG(18:0_30:1) | HMDB0044726 | 56938420 |
| Tracylglycerol(50:1) | TG(18:0_32:0) | HMDB0005374 | 9544004 |
| Tracylglycerol(50:1) | TG(18:0_32:1) | HMDB0005374 |  |
| Tracylglycerol(50:2) | TG(18:0_32:2) | HMDB0044889 | 9544010 |
| Tracylglycerol(52:2) | TG(18:0_34:2) | HMDB0005369 | 9544069 |
| Tracylglycerol(52:3) | TG(18:0_34:3) | HMDB0005425 | 9544087 |
| Tracylglycerol(54:1) | TG(18:0_36:1) | HMDB0005368 | 9544170 |
| Tracylglycerol(54:2) | TG(18:0_36:2) | HMDB0005397 | 25240368 |
| Tracylglycerol(54:3) | TG(18:0_36:3) | HMDB0005405 | 9544216 |
| Tracylglycerol(54:4) | TG(18:0_36:4) | HMDB0005370 | 9544279 |
| Tracylglycerol(54:5) | TG(18:0_36:5) | HMDB0005426 | 9544320 |
| Tracylglycerol(56:6) | TG(18:0_38:6) | HMDB0005412 | 9544638 |
| Tracylglycerol(55:0) | TG(18:0_38:7) | HMDB0063278 | 9544197 |
|  | TG(18:1_26:0) |  |  |
|  | TG(18:1_28:1) |  |  |
| Tracylglycerol(48:1) | TG(18:1_30:0) | HMDB0042130 | 131753274 |
| Tracylglycerol(48:2) | TG(18:1_30:1) | HMDB0047772 | 131758614 |
| Tracylglycerol(48:3) | TG(18:1_30:2) | HMDB0047910 | 131758751 |
| Tracylglycerol(49:1) | TG(18:1_31:0) | HMDB0043026 | 131754160 |
| Tracylglycerol(50:1) | TG(18:1_32:0) | HMDB0005360 | 25240460 |
| Tracylglycerol(50:2) | TG(18:1_32:1) | HMDB0005377 | 9544011 |
| Tracylglycerol(50:3) | TG(18:1_32:2) | HMDB0005433 | 25240357 |
| Tracylglycerol(50:4) | TG(18:1_32:3) | HMDB0042347 | 131753489 |
| Tracylglycerol(51:1) | TG(18:1_33:0) | HMDB0043055 | 131754186 |
| Tracylglycerol(51:2) | TG(18:1_33:1) | HMDB0011707 | 53481039 |
| Tracylglycerol(51:3) | TG(18:1_33:2) | HMDB0011708 | 53481040 |
| Tracylglycerol(51:4) | TG(18:1_33:3) | HMDB0011709 | 53481041 |
| Tracylglycerol(52:2) | TG(18:1_34:1) | HMDB0005382 | 6442384 |
| Tracylglycerol(52:3) | TG(18:1_34:2) | HMDB0005384 | 25240361 |
| Tracylglycerol(52:4) | TG(18:1_34:3) | HMDB0005440 | 25240364 |
| Tracylglycerol(52:5) | TG(18:1_34:4) | HMDB0042350 | 131753492 |
| Tracylglycerol(53:3) | TG(18:1_35:2) | HMDB0043237 | 131754364 |
| Tracylglycerol(53:4) | TG(18:1_35:3) | HMDB0043232 | 131754359 |
| Tracylglycerol(54:1) | TG(18:1_36:0) | HMDB0005381 | 9544166 |
| Tracylglycerol(54:2) | TG(18:1_36:1) | HMDB0005383 | 9544199 |
| Tracylglycerol(54:3) | TG(18:1_36:2) | HMDB0005405 | 9544216 |
| Tracylglycerol(54:4) | TG(18:1_36:3) | HMDB0005455 | 25240371 |
| Tracylglycerol(54:5) | TG(18:1_36:4) | HMDB0005385 | 9544319 |
| Tracylglycerol(54:6) | TG(18:1_36:5) | HMDB0005441 | 9544363 |
| Tracylglycerol(54:7) | TG(18:1_36:6) | HMDB0042359 | 131753501 |
| Tracylglycerol(56:6) | TG(18:1_38:5) | HMDB0005456 | 9544639 |
| Tracylglycerol(56:7) | TG(18:1_38:6) | HMDB0005462 | 9544702 |
| Tracylglycerol(55:1) | TG(18:1_38:7) | HMDB0043225 | 131754352 |
| Tracylglycerol(46:2) | TG(18:2_28:0) | HMDB0042076 | 56936559 |
| Tracylglycerol(48:2) | TG(18:2_30:0) | HMDB0043003 | 56936673 |
| Tracylglycerol(48:3) | TG(18:2_30:1) | HMDB0047778 | 56938422 |
| Tracylglycerol(49:2) | TG(18:2_31:0) | HMDB0043032 | 56938800 |
| Tracylglycerol(50:2) | TG(18:2_32:0) | HMDB0005362 | 25240356 |
| Tracylglycerol(50:3) | TG(18:2_32:1) | HMDB0005379 | 9544021 |
| Tracylglycerol(50:4) | TG(18:2_32:2) | HMDB0005435 | 131750394 |
| Tracylglycerol(51:2) | TG(18:2_33:0) | HMDB0011703 | 53481035 |
| Tracylglycerol(51:3) | TG(18:2_33:1) | HMDB0011708 | 53481040 |
| Tracylglycerol(51:4) | TG(18:2_33:2) | HMDB0011711 | 53481043 |
| Tracylglycerol(52:2) | TG(18:2_34:0) | HMDB0005369 | 9544069 |
| Tracylglycerol(52:3) | TG(18:2_34:1) | HMDB0005384 | 25240361 |
| Tracylglycerol(52:4) | TG(18:2_34:2) | HMDB0005390 | 25240363 |
| Tracylglycerol(52:5) | TG(18:2_34:3) | HMDB0005446 | 25240366 |
| Tracylglycerol(52:6) | TG(18:2_34:4) | HMDB0042530 | 56938180 |
| Tracylglycerol(53:3) | TG(18:2_35:1) | HMDB0043405 | 56938954 |
|  | TG(18:2_35:2) |  |  |
| Tracylglycerol(53:5) | TG(18:2_35:3) | HMDB0043406 | 131754527 |
| Tracylglycerol(54:2) | TG(18:2_36:0) | HMDB0005388 | 9544195 |
| Tracylglycerol(54:3) | TG(18:2_36:1) | HMDB0005389 | 9544231 |
| Tracylglycerol(54:4) | TG(18:2_36:2) | HMDB0005411 | 25240370 |
| Tracylglycerol(54:5) | TG(18:2_36:3) | HMDB0005461 | 25240373 |
| Tracylglycerol(54:6) | TG(18:2_36:4) | HMDB0005391 | 9544362 |
| Tracylglycerol(54:7) | TG(18:2_36:5) | HMDB0005447 | 9544410 |
| Tracylglycerol(56:6) | TG(18:2_38:4) | HMDB0005412 | 9544638 |
| Tracylglycerol(56:7) | TG(18:2_38:5) | HMDB0005462 | 9544702 |
| Tracylglycerol(56:8) | TG(18:2_38:6) | HMDB0005475 | 9544769 |
| Tracylglycerol(48:3) | TG(18:3_30:0) | HMDB0043004 | 56936674 |
| Tracylglycerol(50:3) | TG(18:3_32:0) | HMDB0010417 | 25240358 |
| Tracylglycerol(50:4) | TG(18:3_32:1) | HMDB0042377 | 56938152 |
| Tracylglycerol(51:5) | TG(18:3_33:2) | HMDB0043410 | 56938948 |
| Tracylglycerol(52:3) | TG(18:3_34:0) | HMDB0043927 | 56939505 |
| Tracylglycerol(52:4) | TG(18:3_34:1) | HMDB0045896 | 56938600 |
| Tracylglycerol(52:5) | TG(18:3_34:2) | HMDB0044263 | 56939513 |
| Tracylglycerol(52:6) | TG(18:3_34:3) | HMDB0042553 | 131753690 |
| Tracylglycerol(53:5) | TG(18:3_35:2) | HMDB0043440 | 131754560 |
| Tracylglycerol(54:4) | TG(18:3_36:1) | HMDB0045897 | 56939612 |
| Tracylglycerol(54:5) | TG(18:3_36:2) | HMDB0010459 | 53480523 |
| Tracylglycerol(54:6) | TG(18:3_36:3) | HMDB0044287 | 131755346 |
| Tracylglycerol(54:7) | TG(18:3_36:4) | HMDB0010489 | 53480545 |
| Tracylglycerol(56:8) | TG(18:3_38:5) | HMDB0050761 | 56940157 |
| Tracylglycerol(56:9) | TG(18:3_38:6) | HMDB0053582 | 56940294 |
| Tracylglycerol(52:3) | TG(20:0_32:3) | HMDB0042545 | 56938194 |
| Tracylglycerol(52:4) | TG(20:0_32:4) | HMDB0045896 | 56938600 |
| Tracylglycerol(54:1) | TG(20:0_34:1) | HMDB0005381 | 9544166 |
|  | TG(20:1_24:3) |  |  |
|  | TG(20:1_26:1) |  |  |
| Tracylglycerol(50:2) | TG(20:1_30:1) | HMDB0042312 | 56938048 |
|  | TG(20:1_32:0) |  |  |
| Tracylglycerol(52:2) | TG(20:1_32:1) | HMDB0005378 | 9544079 |
| Tracylglycerol(52:3) | TG(20:1_32:2) | HMDB0005434 | 9544099 |
| Tracylglycerol(52:4) | TG(20:1_32:3) | HMDB0042552 | 56938195 |
| Tracylglycerol(54:1) | TG(20:1_34:0) | HMDB0005368 | 9544170 |
| Tracylglycerol(54:2) | TG(20:1_34:1) | HMDB0005383 | 9544199 |
| Tracylglycerol(54:3) | TG(20:1_34:2) | HMDB0005389 | 9544231 |
| Tracylglycerol(54:4) | TG(20:1_34:3) | HMDB0005445 | 25240372 |
| Tracylglycerol(52:2) | TG(20:2_32:0) | HMDB0042168 | 131754084 |
| Tracylglycerol(52:3) | TG(20:2_32:1) | HMDB0042378 | 131753516 |
| Tracylglycerol(54:3) | TG(20:2_34:1) | HMDB0042408 | 131753545 |
| Tracylglycerol(54:4) | TG(20:2_34:2) | HMDB0042588 | 131753725 |
| Tracylglycerol(54:5) | TG(20:2_34:3) | HMDB0044292 | 131755350 |
| Tracylglycerol(54:6) | TG(20:2_34:4) | HMDB0042590 | 131753727 |
| Tracylglycerol(56:7) | TG(20:2_36:5) | HMDB0042593 | 131753730 |
| Tracylglycerol(52:3) | TG(20:3_32:0) | HMDB0042163 | 131753305 |
| Tracylglycerol(52:4) | TG(20:3_32:1) | HMDB0042349 | 131753491 |
| Tracylglycerol(52:5) | TG(20:3_32:2) | HMDB0042523 | 131753660 |
| Tracylglycerol(54:3) | TG(20:3_34:0) | HMDB0042193 | 131753335 |
| Tracylglycerol(54:4) | TG(20:3_34:1) | HMDB0042403 | 131753540 |
| Tracylglycerol(54:5) | TG(20:3_34:2) | HMDB0042583 | 131753720 |
| Tracylglycerol(54:6) | TG(20:3_34:3) | HMDB0042613 | 131753750 |
| Tracylglycerol(54:6) | TG(20:3_36:3) | HMDB0044343 | 131755401 |
| Tracylglycerol(56:7) | TG(20:3_36:4) | HMDB0010492 | 9544695 |
| Tracylglycerol(56:8) | TG(20:3_36:5) | HMDB0042443 | 131753580 |
| Tracylglycerol(50:4) | TG(20:4_30:0) | HMDB0043013 | 131754147 |
| Tracylglycerol(52:4) | TG(20:4_32:0) | HMDB0005363 | 9544125 |
| Tracylglycerol(52:5) | TG(20:4_32:1) | HMDB0005380 | 9544150 |
| Tracylglycerol(52:6) | TG(20:4_32:2) | HMDB0005436 | 9544179 |
| Tracylglycerol(53:6) | TG(20:4_33:2) | HMDB0043413 | 56938957 |
| Tracylglycerol(54:4) | TG(20:4_34:0) | HMDB0005370 | 9544279 |
| Tracylglycerol(54:5) | TG(20:4_34:1) | HMDB0005385 | 9544319 |
| Tracylglycerol(54:6) | TG(20:4_34:2) | HMDB0005391 | 9544362 |
| Tracylglycerol(54:7) | TG(20:4_34:3) | HMDB0005447 | 9544410 |
| Tracylglycerol(55:7) | TG(20:4_35:3) | HMDB0043326 | 131754448 |
| Tracylglycerol(56:6) | TG(20:4_36:2) | HMDB0005412 | 9544638 |
| Tracylglycerol(56:7) | TG(20:4_36:3) | HMDB0005462 | 9544702 |
| Tracylglycerol(56:8) | TG(20:4_36:4) | HMDB0005392 | 9544762 |
| Tracylglycerol(56:9) | TG(20:4_36:5) | HMDB0005448 | 9544831 |
| Tracylglycerol(54:5) | TG(20:5_34:0) | HMDB0042207 | 56938274 |
| Tracylglycerol(54:6) | TG(20:5_34:1) | HMDB0042417 | 56938286 |
| Tracylglycerol(54:7) | TG(20:5_34:2) | HMDB0042597 | 56938286 |
| Tracylglycerol(56:7) | TG(20:5_36:2) | HMDB0010464 | 9544709 |
| Tracylglycerol(56:8) | TG(20:5_36:3) | HMDB0044189 | 131755257 |
| Tracylglycerol(54:4) | TG(22:0_32:4) | HMDB0046658 | 56938607 |
| Tracylglycerol(54:6) | TG(22:1_32:5) | HMDB0048282 | 131759123 |
| Tracylglycerol(54:6) | TG(22:2_32:4) | HMDB0042801 | 56938236 |
|  | TG(22:3_30:2) | HMDB0341566 |  |
| Tracylglycerol(54:4) | TG(22:4_32:0) | HMDB0042172 | 56938148 |
| Tracylglycerol(54:6) | TG(22:4_32:2) | HMDB0042532 | 56938187 |
| Tracylglycerol(56:6) | TG(22:4_34:2) | HMDB0042592 | 131753729 |
| Tracylglycerol(54:5) | TG(22:5_32:0) | HMDB0042173 | 131753315 |
| Tracylglycerol(54:6) | TG(22:5_32:1) | HMDB0044073 | 131755152 |
| Tracylglycerol(56:6) | TG(22:5_34:1) | HMDB0042413 | 131753550 |
| Tracylglycerol(56:7) | TG(22:5_34:2) | HMDB0044269 | 131755330 |
| Tracylglycerol(56:8) | TG(22:5_34:3) | HMDB0044297 | 131755355 |
| Tracylglycerol(54:6) | TG(22:6_32:0) | HMDB0010418 | 9544489 |
| Tracylglycerol(54:7) | TG(22:6_32:1) | HMDB0044079 | 9544548 |
| Tracylglycerol(56:7) | TG(22:6_34:1) | HMDB0042419 | 56938294 |
| Tracylglycerol(56:8) | TG(22:6_34:2) | HMDB0042599 | 131753736 |
| Tracylglycerol(56:9) | TG(22:6_34:3) | HMDB0042449 | 131753586 |
| ***Vitamins and Cofactors*** |  |  |  |
| Choline chloride | Choline | HMDB0000097 | 305 |
|  |  |  |  |
| **Compound** | **Abbreviation** | **Bio ID** | **CID** |
| ***Hormones (others)*** |  |  |  |
| 17-Hydroxycorticosterone | Cortisol | HMDB00063 | 5754 |
| 17beta-Hydroxy-4-androsten-3-one | Testosterone | HMDB00234 | 5791 |
| ***Isotopes*** |  |  |  |
| Nitrogen-15 | ¹⁵N |  |  |
| Carbon-13 | ¹³C |  |  |

**Supplementary Table S2.**Biochemical abbreviation and Plasma concentration (µM) of Acylcarnitines in low (LRFI) and high (HRFI) Residual Feed Intake bulls on Day 0 and 56 of the feed efficiency trial (Mean ± SEM).

|  |  |  | **Day 0** |  |  |  |  |  | **Day 56** |  |  |
| --- | --- | --- | --- | --- | --- | --- | --- | --- | --- | --- | --- |
|  |  | **LRFI** |  | **HRFI** |  |  |  | **LRFI** |  | **HRFI** |  |
| **COMPOUND** | **Mean** | **SEM** | **Mean** | **SEM** | ***P*** |  | **Mean** | **SEM** | **Mean** | **SEM** | ***P*** |
| **C0** | 12.92 | 0.34 | 13.14 | 0.54 | > 0.05 |  | 12.64 | 1.12 | 14.20 | 0.79 | > 0.05 |
| **C2** | 7.85 | 2.89 | 4.35 | 0.29 | > 0.05 |  | 6.30 | 1.01 | 6.41 | 0.99 | > 0.05 |
| **C3** | 0.26 | 0.03 | 0.32 | 0.03 | > 0.05 |  | 0.23 | 0.04 | 0.29 | 0.03 | > 0.05 |
| **C3-DC (C4-OH)** | 0.05 | 0.00 | 0.08 | 0.01 | 0.004 |  | 0.08 | 0.01 | 0.08 | 0.01 | > 0.05 |
| **C3-OH** | 0.27 | 0.02 | 0.26 | 0.02 | > 0.05 |  | 0.26 | 0.04 | 0.34 | 0.04 | > 0.05 |
| **C3:1** | 0.02 | 0.00 | 0.02 | 0.00 | > 0.05 |  | 0.04 | 0.00 | 0.04 | 0.01 | > 0.05 |
| **C4** | 0.26 | 0.02 | 0.28 | 0.02 | > 0.05 |  | 0.24 | 0.03 | 0.28 | 0.02 | > 0.05 |
| **C4:1** | 0.01 | 0.00 | 0.01 | 0.00 | > 0.05 |  | 0.04 | 0.01 | 0.04 | 0.01 | > 0.05 |
| **C5** | 0.13 | 0.01 | 0.16 | 0.01 | > 0.05 |  | 0.10 | 0.01 | 0.09 | 0.01 | > 0.05 |
| **C5-DC (C6-OH)** | 0.02 | 0.00 | 0.02 | 0.00 | > 0.05 |  | 0.01 | 0.00 | 0.01 | 0.00 | > 0.05 |
| **C5-M-DC** | 0.04 | 0.00 | 0.04 | 0.00 | > 0.05 |  | 0.07 | 0.01 | 0.07 | 0.00 | > 0.05 |
| **C5-OH (C3-DC-M)** | 0.06 | 0.00 | 0.06 | 0.00 | > 0.05 |  | 0.06 | 0.00 | 0.06 | 0.00 | > 0.05 |
| **C5:1** | 0.04 | 0.00 | 0.05 | 0.00 | > 0.05 |  | 0.09 | 0.01 | 0.08 | 0.01 | > 0.05 |
| **C5:1-DC** | 0.01 | 0.01 | 0.02 | 0.01 | > 0.05 |  | 0.11 | 0.01 | 0.12 | 0.02 | > 0.05 |
| **C6 (C4:1-DC)** | 0.04 | 0.00 | 0.04 | 0.00 | > 0.05 |  | 0.09 | 0.01 | 0.09 | 0.01 | > 0.05 |
| **C6:1** | 0.02 | 0.00 | 0.02 | 0.00 | > 0.05 |  | 0.01 | 0.00 | 0.01 | 0.00 | > 0.05 |
| **C7-DC** | 0.00 | 0.00 | 0.00 | 0.00 | > 0.05 |  | 0.00 | 0.00 | 0.00 | 0.00 | > 0.05 |
| **C8** | 0.01 | 0.00 | 0.01 | 0.00 | > 0.05 |  | 0.02 | 0.00 | 0.02 | 0.00 | > 0.05 |
| **C9** | 0.01 | 0.00 | 0.01 | 0.00 | > 0.05 |  | 0.03 | 0.00 | 0.02 | 0.00 | > 0.05 |
| **C10** | 0.04 | 0.00 | 0.04 | 0.00 | > 0.05 |  | 0.05 | 0.00 | 0.05 | 0.00 | > 0.05 |
| **C10:1** | 0.04 | 0.00 | 0.03 | 0.00 | > 0.05 |  | 0.03 | 0.00 | 0.03 | 0.00 | > 0.05 |
| **C10:2** | 0.08 | 0.00 | 0.08 | 0.00 | > 0.05 |  | 0.06 | 0.00 | 0.06 | 0.00 | > 0.05 |
| **C12** | 0.03 | 0.00 | 0.03 | 0.00 | > 0.05 |  | 0.04 | 0.00 | 0.04 | 0.00 | > 0.05 |
| **C12-DC** | 0.64 | 0.02 | 0.65 | 0.02 | > 0.05 |  | 0.73 | 0.03 | 0.73 | 0.02 | > 0.05 |
| **C12:1** | 0.02 | 0.00 | 0.02 | 0.00 | > 0.05 |  | 0.02 | 0.00 | 0.02 | 0.00 | > 0.05 |
| **C14** | 0.02 | 0.00 | 0.02 | 0.00 | > 0.05 |  | 0.03 | 0.00 | 0.03 | 0.00 | > 0.05 |
| **C14:1** | 0.02 | 0.00 | 0.02 | 0.00 | > 0.05 |  | 0.02 | 0.00 | 0.02 | 0.00 | > 0.05 |
| **C14:1-OH** | 0.01 | 0.00 | 0.01 | 0.00 | > 0.05 |  | 0.01 | 0.00 | 0.01 | 0.00 | > 0.05 |
| **C14:2** | 0.01 | 0.00 | 0.01 | 0.00 | > 0.05 |  | 0.01 | 0.00 | 0.01 | 0.00 | > 0.05 |
| **C14:2-OH** | 0.01 | 0.00 | 0.01 | 0.00 | > 0.05 |  | 0.01 | 0.00 | 0.01 | 0.00 | > 0.05 |
| **C16** | 0.01 | 0.00 | 0.01 | 0.00 | > 0.05 |  | 0.02 | 0.00 | 0.02 | 0.00 | > 0.05 |
| **C16-OH** | 0.01 | 0.00 | 0.01 | 0.00 | > 0.05 |  | 0.01 | 0.00 | 0.01 | 0.00 | > 0.05 |
| **C16:1** | 0.01 | 0.00 | 0.01 | 0.00 | > 0.05 |  | 0.01 | 0.00 | 0.01 | 0.00 | > 0.05 |
| **C16:1-OH** | 0.01 | 0.00 | 0.01 | 0.00 | > 0.05 |  | 0.01 | 0.00 | 0.01 | 0.00 | > 0.05 |
| **C16:2** | 0.00 | 0.00 | 0.00 | 0.00 | 0.04 |  | 0.01 | 0.00 | 0.00 | 0.00 | > 0.05 |
| **C16:2-OH** | 0.01 | 0.00 | 0.01 | 0.00 | > 0.05 |  | 0.01 | 0.00 | 0.01 | 0.00 | > 0.05 |
| **C18** | 0.03 | 0.00 | 0.02 | 0.00 | > 0.05 |  | 0.03 | 0.00 | 0.03 | 0.00 | > 0.05 |
| **C18:1** | 0.03 | 0.00 | 0.03 | 0.00 | > 0.05 |  | 0.03 | 0.00 | 0.03 | 0.00 | > 0.05 |
| **C18:1-OH** | 0.04 | 0.00 | 0.04 | 0.00 | > 0.05 |  | 0.02 | 0.00 | 0.02 | 0.00 | > 0.05 |
| **C18:2** | 0.00 | 0.00 | 0.00 | 0.00 | > 0.05 |  | 0.00 | 0.00 | 0.00 | 0.00 | > 0.05 |

**Supplementary Table S3.**Biochemical abbreviation and Plasma concentration (µM) of Alkaloids in low (LRFI) and high (HRFI) Residual Feed Intake bulls on Day 0 and 56 of the feed efficiency trial (Mean ± SEM).

|  |  |  | **Day 0** |  | |  | |  | |  | |  | | **Day 56** | |  | |  | |
| --- | --- | --- | --- | --- | --- | --- | --- | --- | --- | --- | --- | --- | --- | --- | --- | --- | --- | --- | --- |
|  |  | **LRFI** |  | **HRFI** | |  | |  | |  | | **LRFI** | |  | | **HRFI** | |  | |
| **COMPOUND** | **Mean** | **SEM** | **Mean** | **SEM** | | ***P*** | |  | | **Mean** | | **SEM** | | **Mean** | | **SEM** | | ***P*** | |
| **Trigonelline** | 0.05 | 0.01 | 0.07 | 0.02 | > 0.05 | |  | | 0.06 | | 0.01 | | 0.06 | | 0.01 | | > 0.05 | |  |

**Supplementary Table S4.**Biochemical abbreviation and Plasma concentration (µM) of Amine Oxides in low (LRFI) and high (HRFI) Residual Feed Intake bulls on Day 0 and 56 of the feed efficiency trial (Mean ± SEM).

|  |  |  | **Day 0** |  | |  | |  | |  | |  | | **Day 56** | |  | |  | |
| --- | --- | --- | --- | --- | --- | --- | --- | --- | --- | --- | --- | --- | --- | --- | --- | --- | --- | --- | --- |
|  |  | **LRFI** |  | **HRFI** | |  | |  | |  | | **LRFI** | |  | | **HRFI** | |  | |
| **COMPOUND** | **Mean** | **SEM** | **Mean** | **SEM** | | ***P*** | |  | | **Mean** | | **SEM** | | **Mean** | | **SEM** | | ***P*** | |
| **TMAO** | 3.03 | 0.68 | 2.95 | 0.48 | > 0.05 | |  | | 2.24 | | 0.47 | | 2.13 | | 0.50 | | > 0.05 | |  |

**Supplementary Table S5.**Biochemical abbreviation and Plasma concentration (µM) of Amino acids in low (LRFI) and high (HRFI) Residual Feed Intake bulls on Day 0 and 56 of the feed efficiency trial (Mean ± SEM).

|  |  |  | **Day 0** |  | |  | |  | |  | |  | | **Day 56** | |  | |  | |
| --- | --- | --- | --- | --- | --- | --- | --- | --- | --- | --- | --- | --- | --- | --- | --- | --- | --- | --- | --- |
|  |  | **LRFI** |  | **HRFI** | |  | |  | |  | | **LRFI** | |  | | **HRFI** | |  | |
| **COMPOUND** | **Mean** | **SEM** | **Mean** | **SEM** | | ***P*** | |  | | **Mean** | | **SEM** | | **Mean** | | **SEM** | | ***P*** | |
| **Ala** | 250.00 | 12.54 | 268.43 | 8.05 | > 0.05 | |  | | 251.04 | | 11.37 | | 254.48 | | 12.27 | | > 0.05 | |  |
| **Arg** | 96.37 | 2.96 | 101.24 | 3.01 | > 0.05 | |  | | 102.69 | | 3.06 | | 100.58 | | 3.26 | | > 0.05 | |  |
| **Asn** | 36.36 | 1.68 | 39.82 | 1.31 | > 0.05 | |  | | 39.60 | | 1.59 | | 39.63 | | 1.35 | | > 0.05 | |  |
| **Asp** | 9.53 | 1.47 | 11.11 | 0.87 | > 0.05 | |  | | 12.61 | | 1.35 | | 11.60 | | 1.60 | | > 0.05 | |  |
| **Cys** | 27.11 | 0.83 | 28.59 | 0.75 | > 0.05 | |  | | 27.23 | | 0.80 | | 27.43 | | 0.84 | | > 0.05 | |  |
| **Gln** | 367.52 | 8.84 | 373.63 | 8.85 | > 0.05 | |  | | 372.33 | | 8.94 | | 376.61 | | 10.00 | | > 0.05 | |  |
| **Glu** | 104.56 | 5.77 | 94.34 | 5.31 | > 0.05 | |  | | 92.30 | | 5.23 | | 90.19 | | 5.44 | | > 0.05 | |  |
| **Gly** | 413.96 | 16.51 | 449.43 | 16.98 | > 0.05 | |  | | 431.21 | | 15.06 | | 421.66 | | 16.81 | | > 0.05 | |  |
| **His** | 71.30 | 2.08 | 78.09 | 1.94 | 0.02 | |  | | 79.34 | | 2.41 | | 76.04 | | 3.12 | | > 0.05 | |  |
| **Ile** | 147.34 | 5.96 | 176.59 | 4.83 | 0.0003 | |  | | 157.25 | | 5.83 | | 168.08 | | 6.38 | | > 0.05 | |  |
| **Leu** | 219.79 | 9.29 | 246.00 | 7.61 | 0.03 | |  | | 231.17 | | 10.42 | | 231.37 | | 8.42 | | > 0.05 | |  |
| **Lys** | 103.42 | 3.83 | 111.22 | 3.44 | > 0.05 | |  | | 110.03 | | 4.88 | | 111.80 | | 3.55 | | > 0.05 | |  |
| **Met** | 29.68 | 1.34 | 32.33 | 1.34 | > 0.05 | |  | | 29.78 | | 1.24 | | 30.56 | | 1.04 | | > 0.05 | |  |
| **Phe** | 64.56 | 2.36 | 69.76 | 2.73 | > 0.05 | |  | | 67.83 | | 3.04 | | 73.28 | | 2.86 | | > 0.05 | |  |
| **Pro** | 87.06 | 2.71 | 89.82 | 2.25 | > 0.05 | |  | | 88.65 | | 2.63 | | 89.22 | | 2.57 | | > 0.05 | |  |
| **Ser** | 86.22 | 5.05 | 93.07 | 4.08 | > 0.05 | |  | | 87.54 | | 4.41 | | 87.08 | | 3.69 | | > 0.05 | |  |
| **Thr** | 82.23 | 3.52 | 91.24 | 3.32 | > 0.05 | |  | | 86.65 | | 3.38 | | 83.70 | | 3.54 | | > 0.05 | |  |
| **Trp** | 55.39 | 2.09 | 59.32 | 1.81 | > 0.05 | |  | | 59.83 | | 2.97 | | 54.91 | | 2.34 | | > 0.05 | |  |
| **Tyr** | 71.85 | 3.17 | 88.69 | 4.02 | 0.02 | |  | | 90.58 | | 4.70 | | 83.40 | | 4.35 | | > 0.05 | |  |
| **Val** | 236.87 | 12.37 | 269.46 | 14.22 | > 0.05 | |  | | 262.33 | | 13.16 | | 262.86 | | 15.16 | | > 0.05 | |  |

**Supplementary Table S6.**Biochemical abbreviation and Plasma concentration (µM) of Amino acids Related in low (LRFI) and high (HRFI) Residual Feed Intake bulls on Day 0 and 56 of the feed efficiency trial (Mean ± SEM).

|  |  |  | **Day 0** |  | |  | |  | |  | |  | | **Day 56** | |  | |  | |
| --- | --- | --- | --- | --- | --- | --- | --- | --- | --- | --- | --- | --- | --- | --- | --- | --- | --- | --- | --- |
|  |  | **LRFI** |  | **HRFI** | |  | |  | |  | | **LRFI** | |  | | **HRFI** | |  | |
| **COMPOUND** | **Mean** | **SEM** | **Mean** | **SEM** | | ***P*** | |  | | **Mean** | | **SEM** | | **Mean** | | **SEM** | | ***P*** | |
| **1-Met-His** | 4.98 | 0.27 | 4.59 | 0.25 | > 0.05 | |  | | 5.36 | | 0.32 | | 4.68 | | 0.24 | | > 0.05 | |  |
| **3-Met-His** | 4.28 | 0.20 | 4.06 | 0.17 | > 0.05 | |  | | 4.72 | | 0.20 | | 4.34 | | 0.19 | | > 0.05 | |  |
| **5-AVA** | 0.42 | 0.04 | 0.50 | 0.06 | > 0.05 | |  | | 0.45 | | 0.04 | | 0.47 | | 0.05 | | > 0.05 | |  |
| **AABA** | 6.27 | 0.35 | 6.45 | 0.42 | > 0.05 | |  | | 6.59 | | 0.48 | | 6.63 | | 0.42 | | > 0.05 | |  |
| **Ac-Orn** | 2.36 | 0.27 | 2.50 | 0.35 | > 0.05 | |  | | 2.06 | | 0.27 | | 2.40 | | 0.22 | | > 0.05 | |  |
| **ADMA** | 1.23 | 0.03 | 1.29 | 0.04 | > 0.05 | |  | | 1.34 | | 0.03 | | 1.24 | | 0.03 | | > 0.05 | |  |
| **alpha-AAA** | 1.47 | 0.08 | 1.35 | 0.06 | > 0.05 | |  | | 1.41 | | 0.07 | | 1.30 | | 0.05 | | > 0.05 | |  |
| **Anserine** | 0.36 | 0.02 | 0.33 | 0.02 | > 0.05 | |  | | 0.34 | | 0.01 | | 0.31 | | 0.02 | | > 0.05 | |  |
| **BABA** | 0.07 | 0.01 | 0.07 | 0.01 | > 0.05 | |  | | 0.07 | | 0.01 | | 0.07 | | 0.01 | | > 0.05 | |  |
| **Betaine** | 53.82 | 4.01 | 47.41 | 4.13 | > 0.05 | |  | | 58.25 | | 4.04 | | 56.66 | | 4.29 | | > 0.05 | |  |
| **c4-OH-Pro** | 0.01 | 0.00 | 0.02 | 0.00 | > 0.05 | |  | | 0.01 | | 0.00 | | 0.01 | | 0.00 | | > 0.05 | |  |
| **Carnosine** | 17.67 | 1.03 | 16.73 | 0.85 | > 0.05 | |  | | 16.46 | | 0.76 | | 15.02 | | 0.94 | | > 0.05 | |  |
| **Cit** | 65.39 | 3.13 | 69.19 | 4.33 | > 0.05 | |  | | 67.89 | | 4.06 | | 72.47 | | 4.52 | | > 0.05 | |  |
| **Creatinine** | 104.62 | 3.23 | 99.83 | 2.55 | > 0.05 | |  | | 108.22 | | 3.08 | | 97.05 | | 2.76 | | 0.01 | |  |
| **Cystine** | 13.38 | 0.76 | 15.12 | 0.84 | > 0.05 | |  | | 12.74 | | 0.83 | | 13.73 | | 0.66 | | > 0.05 | |  |
| **DOPA** | 0.02 | 0.00 | 0.02 | 0.00 | > 0.05 | |  | | 0.02 | | 0.00 | | 0.03 | | 0.00 | | > 0.05 | |  |
| **HArg** | 2.75 | 0.11 | 2.75 | 0.10 | > 0.05 | |  | | 2.71 | | 0.12 | | 2.86 | | 0.13 | | > 0.05 | |  |
| **HCys** | 3.45 | 0.14 | 3.89 | 0.17 | > 0.05 | |  | | 3.56 | | 0.18 | | 3.33 | | 0.16 | | > 0.05 | |  |
| **Kynurenine** | 4.92 | 0.24 | 5.13 | 0.24 | > 0.05 | |  | | 5.25 | | 0.31 | | 5.33 | | 0.17 | | > 0.05 | |  |
| **Met-SO** | 1.63 | 0.09 | 1.64 | 0.11 | > 0.05 | |  | | 1.55 | | 0.11 | | 1.72 | | 0.09 | | > 0.05 | |  |
| **Nitro-Tyr** | 0.46 | 0.02 | 0.44 | 0.02 | > 0.05 | |  | | 0.44 | | 0.02 | | 0.45 | | 0.02 | | > 0.05 | |  |
| **Orn** | 80.28 | 3.21 | 81.48 | 3.74 | > 0.05 | |  | | 82.96 | | 3.85 | | 84.95 | | 3.02 | | > 0.05 | |  |
| **PAG** | 4.36 | 0.42 | 4.86 | 0.54 | > 0.05 | |  | | 5.24 | | 0.53 | | 5.44 | | 0.68 | | > 0.05 | |  |
| **PheAlaBetaine** | 0.00 | 0.00 | 0.00 | 0.00 | > 0.05 | |  | | 0.00 | | 0.00 | | 0.00 | | 0.00 | | > 0.05 | |  |
| **ProBetaine** | 0.36 | 0.03 | 0.34 | 0.03 | > 0.05 | |  | | 0.42 | | 0.05 | | 0.40 | | 0.03 | | > 0.05 | |  |
| **Sarcosine** | 2.95 | 0.12 | 2.96 | 0.11 | > 0.05 | |  | | 3.07 | | 0.12 | | 3.19 | | 0.09 | | > 0.05 | |  |
| **SDMA** | 0.57 | 0.03 | 0.54 | 0.03 | > 0.05 | |  | | 0.58 | | 0.02 | | 0.55 | | 0.02 | | > 0.05 | |  |
| **t4-OH-Pro** | 31.85 | 1.39 | 27.86 | 1.09 | > 0.05 | |  | | 31.02 | | 1.10 | | 28.74 | | 1.30 | | > 0.05 | |  |
| **Taurine** | 32.55 | 2.47 | 35.16 | 2.59 | > 0.05 | |  | | 32.79 | | 2.16 | | 37.55 | | 2.54 | | > 0.05 | |  |
| **TrpBetaine** | 0.01 | 0.00 | 0.01 | 0.00 | > 0.05 | |  | | 0.01 | | 0.00 | | 0.01 | | 0.00 | | > 0.05 | |  |

**Supplementary Table S7.**Biochemical abbreviation and Plasma concentration (µM) of Bile Acids in low (LRFI) and high (HRFI) Residual Feed Intake bulls on Day 0 and 56 of the feed efficiency trial (Mean ± SEM).

|  |  |  | **Day 0** |  | |  | |  | |  | |  | | **Day 56** | |  | |  | |
| --- | --- | --- | --- | --- | --- | --- | --- | --- | --- | --- | --- | --- | --- | --- | --- | --- | --- | --- | --- |
|  |  | **LRFI** |  | **HRFI** | |  | |  | |  | | **LRFI** | |  | | **HRFI** | |  | |
| **COMPOUND** | **Mean** | **SEM** | **Mean** | **SEM** | | ***P*** | |  | | **Mean** | | **SEM** | | **Mean** | | **SEM** | | ***P*** | |
| **CA** | 8.73 | 1.13 | 9.04 | 0.99 | > 0.05 | |  | | 9.01 | | 1.11 | | 10.64 | | 0.97 | | > 0.05 | |  |
| **CDCA** | 0.73 | 0.14 | 1.01 | 0.23 | > 0.05 | |  | | 1.17 | | 0.26 | | 1.15 | | 0.18 | | > 0.05 | |  |
| **DCA** | 1.52 | 0.29 | 1.51 | 0.24 | > 0.05 | |  | | 1.94 | | 0.34 | | 1.99 | | 0.27 | | > 0.05 | |  |
| **GCA** | 5.40 | 0.67 | 3.84 | 0.62 | > 0.05 | |  | | 4.52 | | 0.76 | | 4.82 | | 0.76 | | > 0.05 | |  |
| **GCDCA** | 0.55 | 0.05 | 0.48 | 0.07 | > 0.05 | |  | | 0.61 | | 0.09 | | 0.54 | | 0.07 | | > 0.05 | |  |
| **GDCA** | 1.69 | 0.19 | 1.18 | 0.17 | 0.04 | |  | | 1.49 | | 0.22 | | 1.14 | | 0.14 | | > 0.05 | |  |
| **GLCA** | 0.11 | 0.01 | 0.08 | 0.01 | 0.03 | |  | | 0.10 | | 0.01 | | 0.09 | | 0.01 | | > 0.05 | |  |
| **GLCAS** | 0.00 | 0.00 | 0.00 | 0.00 | > 0.05 | |  | | 0.01 | | 0.00 | | 0.00 | | 0.00 | | > 0.05 | |  |
| **GUDCA** | 0.00 | 0.00 | 0.00 | 0.00 | > 0.05 | |  | | 0.00 | | 0.00 | | 0.00 | | 0.00 | | > 0.05 | |  |
| **TCA** | 1.24 | 0.22 | 0.78 | 0.13 | 0.04 | |  | | 0.96 | | 0.18 | | 0.85 | | 0.13 | | > 0.05 | |  |
| **TCDCA** | 0.21 | 0.02 | 0.25 | 0.04 | > 0.05 | |  | | 0.17 | | 0.02 | | 0.19 | | 0.03 | | > 0.05 | |  |
| **TDCA** | 0.33 | 0.05 | 0.23 | 0.04 | > 0.05 | |  | | 0.35 | | 0.05 | | 0.20 | | 0.02 | | 0.04 | |  |
| **TLCA** | 0.04 | 0.01 | 0.03 | 0.00 | > 0.05 | |  | | 0.04 | | 0.01 | | 0.03 | | 0.00 | | > 0.05 | |  |
| **TMCA** | 0.01 | 0.00 | 0.00 | 0.00 | 0.02 | |  | | 0.01 | | 0.00 | | 0.00 | | 0.00 | | > 0.05 | |  |

**Supplementary Table S8.**Biochemical abbreviation and Plasma concentration (µM) of Biogenic Amines in low (LRFI) and high (HRFI) Residual Feed Intake bulls on Day 0 and 56 of the feed efficiency trial (Mean ± SEM).

|  |  |  | **Day 0** |  | |  | |  | |  | |  | | **Day 56** | |  | |  | |
| --- | --- | --- | --- | --- | --- | --- | --- | --- | --- | --- | --- | --- | --- | --- | --- | --- | --- | --- | --- |
|  |  | **LRFI** |  | **HRFI** | |  | |  | |  | | **LRFI** | |  | | **HRFI** | |  | |
| **COMPOUND** | **Mean** | **SEM** | **Mean** | **SEM** | | ***P*** | |  | | **Mean** | | **SEM** | | **Mean** | | **SEM** | | ***P*** | |
| **beta-Ala** | 1.55 | 0.08 | 1.53 | 0.07 | > 0.05 | |  | | 1.58 | | 0.08 | | 1.73 | | 0.06 | | > 0.05 | |  |
| **Dopamine** | 0.09 | 0.02 | 0.10 | 0.02 | > 0.05 | |  | | 0.11 | | 0.02 | | 0.11 | | 0.02 | | > 0.05 | |  |
| **GABA** | 0.13 | 0.01 | 0.12 | 0.01 | > 0.05 | |  | | 0.13 | | 0.01 | | 0.12 | | 0.01 | | > 0.05 | |  |
| **Histamine** | 0.00 | 0.00 | 0.00 | 0.00 | > 0.05 | |  | | 0.00 | | 0.00 | | 0.00 | | 0.00 | | > 0.05 | |  |
| **PEA** | 0.00 | 0.00 | 0.00 | 0.00 | > 0.05 | |  | | 0.00 | | 0.00 | | 0.00 | | 0.00 | | > 0.05 | |  |
| **Putrescine** | 0.16 | 0.01 | 0.14 | 0.01 | > 0.05 | |  | | 0.14 | | 0.01 | | 0.13 | | 0.01 | | > 0.05 | |  |
| **Serotonin** | 0.13 | 0.01 | 0.14 | 0.01 | > 0.05 | |  | | 0.14 | | 0.01 | | 0.12 | | 0.01 | | > 0.05 | |  |
| **Spermidine** | 0.02 | 0.00 | 0.02 | 0.00 | > 0.05 | |  | | 0.02 | | 0.00 | | 0.03 | | 0.00 | | > 0.05 | |  |
| **Spermine** | 0.05 | 0.01 | 0.05 | 0.01 | > 0.05 | |  | | 0.06 | | 0.01 | | 0.06 | | 0.01 | | > 0.05 | |  |

**Supplementary Table S9.**Biochemical abbreviation and Plasma concentration (µM) of Carboxylic Acids in low (LRFI) and high (HRFI) Residual Feed Intake bulls on Day 0 and 56 of the feed efficiency trial (Mean ± SEM).

|  | |  | |  | | **Day 0** | |  | |  | |  | |  | |  | | **Day 56** | |  | |  |
| --- | --- | --- | --- | --- | --- | --- | --- | --- | --- | --- | --- | --- | --- | --- | --- | --- | --- | --- | --- | --- | --- | --- |
|  | |  | | **LRFI** | |  | | **HRFI** | |  | |  | |  | | **LRFI** | |  | | **HRFI** | |  |
| **COMPOUND** | | **Mean** | | **SEM** | | **Mean** | | **SEM** | | ***P*** | |  | | **Mean** | | **SEM** | | **Mean** | | **SEM** | | ***P*** |
| **AconAcid** | 15.24 | | 1.20 | | 12.90 | | 1.17 | | > 0.05 | |  | | 12.47 | | 1.19 | | 10.87 | | 0.89 | | > 0.05 | |
| **DiCA(12:0)** | 0.28 | | 0.01 | | 0.23 | | 0.02 | | 0.02 | |  | | 0.24 | | 0.02 | | 0.25 | | 0.02 | | > 0.05 | |
| **DiCA(14:0)** | 0.04 | | 0.01 | | 0.04 | | 0.01 | | > 0.05 | |  | | 0.03 | | 0.01 | | 0.04 | | 0.01 | | > 0.05 | |
| **HipAcid** | 54.02 | | 3.69 | | 45.72 | | 3.66 | | > 0.05 | |  | | 49.84 | | 3.93 | | 47.12 | | 3.16 | | > 0.05 | |
| **Lac** | 7319 | | 1025 | | 6255 | | 734 | | > 0.05 | |  | | 6240 | | 796 | | 5394 | | 681 | | > 0.05 | |
| **OH-GlutAcid** | 4.79 | | 0.35 | | 4.42 | | 0.34 | | > 0.05 | |  | | 4.71 | | 0.32 | | 3.84 | | 0.26 | | 0.01 | |
| **Suc** | 9.53 | | 1.22 | | 7.64 | | 0.90 | | > 0.05 | |  | | 7.88 | | 0.94 | | 8.35 | | 0.84 | | > 0.05 | |

**Supplementary Table S10.**Biochemical abbreviation and Plasma concentration (µM) of Ceramides in low (LRFI) and high (HRFI) Residual Feed Intake bulls on Day 0 and 56 of the feed efficiency trial (Mean ± SEM).

|  |  |  | **Day 0** |  | |  | |  | |  |  | | | **Day 56** |  | |  | |  |
| --- | --- | --- | --- | --- | --- | --- | --- | --- | --- | --- | --- | --- | --- | --- | --- | --- | --- | --- | --- |
|  |  | **LRFI** |  | **HRFI** | |  | |  | |  | **LRFI** | | |  | **HRFI** | |  | |  |
| **COMPOUND** | **Mean** | **SEM** | **Mean** | **SEM** | | ***P*** | |  | | **Mean** | **SEM** | | | **Mean** | **SEM** | | ***P*** | |  |
| **Cer(d16:1/18:0)** | 0.05 | 0.00 | 0.05 | 0.00 | > 0.05 | |  | | 0.05 | | | 0.00 | 0.05 | | | 0.00 | | > 0.05 | |
| **Cer(d16:1/20:0)** | 0.08 | 0.00 | 0.09 | 0.00 | > 0.05 | |  | | 0.09 | | | 0.00 | 0.08 | | | 0.00 | | > 0.05 | |
| **Cer(d16:1/22:0)** | 0.06 | 0.00 | 0.06 | 0.00 | > 0.05 | |  | | 0.06 | | | 0.00 | 0.05 | | | 0.00 | | > 0.05 | |
| **Cer(d16:1/23:0)** | 0.06 | 0.01 | 0.05 | 0.01 | > 0.05 | |  | | 0.06 | | | 0.00 | 0.05 | | | 0.00 | | > 0.05 | |
| **Cer(d16:1/24:0)** | 0.09 | 0.00 | 0.10 | 0.01 | > 0.05 | |  | | 0.10 | | | 0.01 | 0.10 | | | 0.00 | | > 0.05 | |
| **Cer(d18:1/14:0)** | 0.07 | 0.00 | 0.07 | 0.00 | > 0.05 | |  | | 0.07 | | | 0.00 | 0.07 | | | 0.00 | | > 0.05 | |
| **Cer(d18:1/16:0)** | 0.20 | 0.02 | 0.21 | 0.03 | > 0.05 | |  | | 0.21 | | | 0.02 | 0.21 | | | 0.02 | | > 0.05 | |
| **Cer(d18:1/18:0(OH))** | 0.43 | 0.02 | 0.45 | 0.02 | > 0.05 | |  | | 0.46 | | | 0.02 | 0.45 | | | 0.02 | | > 0.05 | |
| **Cer(d18:1/18:0)** | 0.06 | 0.01 | 0.06 | 0.01 | > 0.05 | |  | | 0.08 | | | 0.01 | 0.06 | | | 0.01 | | > 0.05 | |
| **Cer(d18:1/18:1)** | 0.03 | 0.00 | 0.03 | 0.00 | > 0.05 | |  | | 0.03 | | | 0.00 | 0.04 | | | 0.00 | | > 0.05 | |
| **Cer(d18:1/20:0(OH))** | 0.34 | 0.03 | 0.37 | 0.03 | > 0.05 | |  | | 0.36 | | | 0.03 | 0.36 | | | 0.04 | | > 0.05 | |
| **Cer(d18:1/20:0)** | 0.02 | 0.00 | 0.02 | 0.00 | > 0.05 | |  | | 0.02 | | | 0.00 | 0.02 | | | 0.00 | | > 0.05 | |
| **Cer(d18:1/22:0)** | 0.08 | 0.01 | 0.09 | 0.01 | > 0.05 | |  | | 0.09 | | | 0.01 | 0.08 | | | 0.01 | | > 0.05 | |
| **Cer(d18:1/23:0)** | 0.23 | 0.02 | 0.19 | 0.01 | 0.04 | |  | | 0.27 | | | 0.02 | 0.21 | | | 0.01 | | 0.02 | |
| **Cer(d18:1/24:0)** | 0.20 | 0.01 | 0.17 | 0.01 | > 0.05 | |  | | 0.21 | | | 0.01 | 0.18 | | | 0.01 | | > 0.05 | |
| **Cer(d18:1/24:1)** | 0.09 | 0.01 | 0.07 | 0.01 | > 0.05 | |  | | 0.11 | | | 0.01 | 0.09 | | | 0.01 | | > 0.05 | |
| **Cer(d18:1/25:0)** | 0.09 | 0.01 | 0.09 | 0.01 | > 0.05 | |  | | 0.09 | | | 0.01 | 0.08 | | | 0.01 | | > 0.05 | |
| **Cer(d18:1/26:0)** | 0.04 | 0.00 | 0.05 | 0.00 | > 0.05 | |  | | 0.05 | | | 0.00 | 0.04 | | | 0.00 | | > 0.05 | |
| **Cer(d18:1/26:1)** | 0.02 | 0.00 | 0.02 | 0.00 | > 0.05 | |  | | 0.02 | | | 0.00 | 0.02 | | | 0.00 | | > 0.05 | |
| **Cer(d18:2/14:0)** | 0.02 | 0.00 | 0.02 | 0.00 | > 0.05 | |  | | 0.02 | | | 0.00 | 0.02 | | | 0.00 | | > 0.05 | |
| **Cer(d18:2/16:0)** | 0.04 | 0.00 | 0.04 | 0.00 | > 0.05 | |  | | 0.04 | | | 0.01 | 0.04 | | | 0.00 | | > 0.05 | |
| **Cer(d18:2/18:0)** | 0.04 | 0.01 | 0.05 | 0.00 | > 0.05 | |  | | 0.04 | | | 0.00 | 0.04 | | | 0.00 | | > 0.05 | |
| **Cer(d18:2/18:1)** | 0.02 | 0.00 | 0.02 | 0.00 | > 0.05 | |  | | 0.02 | | | 0.00 | 0.02 | | | 0.00 | | > 0.05 | |
| **Cer(d18:2/20:0)** | 0.04 | 0.00 | 0.05 | 0.00 | > 0.05 | |  | | 0.04 | | | 0.00 | 0.04 | | | 0.00 | | > 0.05 | |
| **Cer(d18:2/22:0)** | 0.03 | 0.00 | 0.03 | 0.00 | > 0.05 | |  | | 0.03 | | | 0.00 | 0.02 | | | 0.00 | | > 0.05 | |
| **Cer(d18:2/23:0)** | 0.02 | 0.00 | 0.03 | 0.00 | > 0.05 | |  | | 0.03 | | | 0.00 | 0.03 | | | 0.00 | | > 0.05 | |
| **Cer(d18:2/24:0)** | 0.05 | 0.01 | 0.06 | 0.01 | > 0.05 | |  | | 0.05 | | | 0.01 | 0.05 | | | 0.01 | | > 0.05 | |
| **Cer(d18:2/24:1)** | 0.04 | 0.00 | 0.04 | 0.00 | > 0.05 | |  | | 0.04 | | | 0.00 | 0.04 | | | 0.00 | | > 0.05 | |

**Supplementary Table S11.**Biochemical abbreviation and Plasma concentration (µM) of Cholesterol Esters in low (LRFI) and high (HRFI) Residual Feed Intake bulls on Day 0 and 56 of the feed efficiency trial (Mean ± SEM).

|  | |  |  | | **Day 0** | |  | |  | |  | |  |  | | **Day 56** | |  | |  | |  |
| --- | --- | --- | --- | --- | --- | --- | --- | --- | --- | --- | --- | --- | --- | --- | --- | --- | --- | --- | --- | --- | --- | --- |
|  | |  | **LRFI** | |  | | **HRFI** | |  | |  | |  | **LRFI** | |  | | **HRFI** | |  | |  |
| **COMPOUND** | | **Mean** | **SEM** | | **Mean** | | **SEM** | | ***P*** | |  | | **Mean** | **SEM** | | **Mean** | | **SEM** | | ***P*** | |  |
| **CE(14:0)** | 67.56 | | | 8.59 | | 62.64 | | 7.60 | | > 0.05 | |  | 69.03 | | 8.89 | | 73.12 | | 8.57 | | > 0.05 | |
| **CE(14:1)** | 0.93 | | | 0.12 | | 0.94 | | 0.12 | | > 0.05 | |  | 1.02 | | 0.12 | | 1.06 | | 0.13 | | > 0.05 | |
| **CE(15:0)** | 6.20 | | | 0.85 | | 6.39 | | 0.80 | | > 0.05 | |  | 6.39 | | 0.74 | | 6.36 | | 0.73 | | > 0.05 | |
| **CE(15:1)** | 1.28 | | | 0.19 | | 1.29 | | 0.18 | | > 0.05 | |  | 1.24 | | 0.16 | | 1.47 | | 0.21 | | > 0.05 | |
| **CE(16:0)** | 405.76 | | | 54.15 | | 440.00 | | 55.44 | | > 0.05 | |  | 439.37 | | 47.35 | | 443.22 | | 58.72 | | > 0.05 | |
| **CE(16:1)** | 224.78 | | | 30.08 | | 243.91 | | 30.19 | | > 0.05 | |  | 256.41 | | 30.91 | | 271.60 | | 34.83 | | > 0.05 | |
| **CE(17:0)** | 56.36 | | | 8.58 | | 52.26 | | 7.66 | | > 0.05 | |  | 53.04 | | 6.87 | | 51.63 | | 7.62 | | > 0.05 | |
| **CE(17:1)** | 45.00 | | | 5.90 | | 54.97 | | 7.36 | | > 0.05 | |  | 56.70 | | 6.96 | | 58.50 | | 7.27 | | > 0.05 | |
| **CE(18:0)** | 43.30 | | | 5.78 | | 45.41 | | 6.20 | | > 0.05 | |  | 44.73 | | 5.50 | | 51.10 | | 6.68 | | > 0.05 | |
| **CE(18:1)** | 1438.0 | | | 202.4 | | 1425.3 | | 215.0 | | > 0.05 | |  | 1415.3 | | 189.09 | | 1569.4 | | 237.23 | | > 0.05 | |
| **CE(18:2)** | 27164 | | | 4087 | | 24468 | | 3858 | | > 0.05 | |  | 24100 | | 3490 | | 24869 | | 3786 | | > 0.05 | |
| **CE(18:3)** | 2594 | | | 326 | | 2814 | | 426 | | > 0.05 | |  | 2887 | | 385 | | 3292 | | 489 | | > 0.05 | |
| **CE(20:0)** | 35.91 | | | 6.59 | | 30.12 | | 6.67 | | > 0.05 | |  | 34.18 | | 6.03 | | 33.43 | | 7.55 | | > 0.05 | |
| **CE(20:1)** | 14.46 | | | 2.53 | | 13.16 | | 2.66 | | > 0.05 | |  | 14.49 | | 2.21 | | 12.78 | | 2.59 | | > 0.05 | |
| **CE(20:3)** | 92.13 | | | 13.01 | | 107.29 | | 14.32 | | > 0.05 | |  | 103.98 | | 11.39 | | 121.81 | | 15.54 | | > 0.05 | |
| **CE(20:4)** | 567.35 | | | 54.39 | | 720.10 | | 96.36 | | > 0.05 | |  | 752.07 | | 86.07 | | 798.41 | | 111.73 | | > 0.05 | |
| **CE(20:5)** | 297.38 | | | 43.05 | | 332.25 | | 46.33 | | > 0.05 | |  | 327.94 | | 31.62 | | 326.79 | | 43.82 | | > 0.05 | |
| **CE(22:0)** | 0.07 | | | 0.03 | | 0.05 | | 0.02 | | > 0.05 | |  | 0.07 | | 0.03 | | 0.09 | | 0.03 | | > 0.05 | |
| **CE(22:1)** | 1.09 | | | 0.12 | | 1.12 | | 0.12 | | > 0.05 | |  | 1.22 | | 0.10 | | 1.25 | | 0.14 | | > 0.05 | |
| **CE(22:2)** | 0.34 | | | 0.04 | | 0.37 | | 0.05 | | > 0.05 | |  | 0.38 | | 0.04 | | 0.35 | | 0.04 | | > 0.05 | |
| **CE(22:5)** | 3.35 | | | 0.73 | | 2.56 | | 0.71 | | > 0.05 | |  | 4.71 | | 1.12 | | 4.27 | | 1.13 | | > 0.05 | |
| **CE(22:6)** | 39.55 | | | 4.39 | | 36.22 | | 3.56 | | > 0.05 | |  | 41.84 | | 4.55 | | 39.41 | | 4.41 | | > 0.05 | |

**Supplementary Table S12.**Biochemical abbreviation and Plasma concentration (µM) of Cresols in low (LRFI) and high (HRFI) Residual Feed Intake bulls on Day 0 and 56 of the feed efficiency trial (Mean ± SEM).

|  |  |  | **Day 0** |  | |  | |  | |  |  | | **Day 56** | |  | | |  |  |
| --- | --- | --- | --- | --- | --- | --- | --- | --- | --- | --- | --- | --- | --- | --- | --- | --- | --- | --- | --- |
|  |  | **LRFI** |  | **HRFI** | |  | |  | |  | **LRFI** | |  | | **HRFI** | | |  |  |
| **COMPOUND** | **Mean** | **SEM** | **Mean** | **SEM** | | ***P*** | |  | | **Mean** | **SEM** | | **Mean** | | **SEM** | | | ***P*** |  |
| **p-Cresol-SO4** | 48.21 | 3.86 | 47.86 | 4.11 | > 0.05 | |  | | 45.15 | | | 4.25 | | 41.51 | | 3.83 | > 0.05 | | |

**Supplementary Table S13.**Biochemical abbreviation and Plasma concentration (µM) of Diacylglycerols in low (LRFI) and high (HRFI) Residual Feed Intake bulls on Day 0 and 56 of the feed efficiency trial (Mean ± SEM).

|  |  |  | **Day 0** |  | |  | |  | |  |  | | **Day 56** | | |  |  | | |
| --- | --- | --- | --- | --- | --- | --- | --- | --- | --- | --- | --- | --- | --- | --- | --- | --- | --- | --- | --- |
|  |  | **LRFI** |  | **HRFI** | |  | |  | |  | **LRFI** | |  | | | **HRFI** |  | | |
| **COMPOUND** | **Mean** | **SEM** | **Mean** | **SEM** | | ***P*** | |  | | **Mean** | **SEM** | | **Mean** | | | **SEM** | ***P*** | | |
| **DG(14:0_14:0)** | 5.87 | 0.81 | 6.27 | 0.80 | > 0.05 | |  | | 6.28 | | | 0.76 | | 6.78 | 0.85 | | | > 0.05 |  |
| **DG(14:0_18:1)** | 1.71 | 0.24 | 1.72 | 0.22 | > 0.05 | |  | | 1.82 | | | 0.23 | | 1.94 | 0.24 | | | > 0.05 |  |
| **DG(14:0_18:2)** | 3.83 | 0.50 | 3.68 | 0.47 | > 0.05 | |  | | 4.02 | | | 0.51 | | 3.87 | 0.44 | | | > 0.05 |  |
| **DG(14:0_20:0)** | 0.02 | 0.00 | 0.02 | 0.00 | > 0.05 | |  | | 0.02 | | | 0.00 | | 0.01 | 0.00 | | | > 0.05 |  |
| **DG(14:1_18:1)** | 0.15 | 0.02 | 0.18 | 0.02 | > 0.05 | |  | | 0.20 | | | 0.02 | | 0.25 | 0.03 | | | > 0.05 |  |
| **DG(14:1_20:2)** | 0.03 | 0.01 | 0.02 | 0.01 | > 0.05 | |  | | 0.03 | | | 0.01 | | 0.04 | 0.01 | | | > 0.05 |  |
| **DG(16:0_16:0)** | 0.09 | 0.04 | 0.07 | 0.04 | > 0.05 | |  | | 0.14 | | | 0.05 | | 0.26 | 0.06 | | | > 0.05 |  |
| **DG(16:0_16:1)** | 2.64 | 0.47 | 3.47 | 0.56 | > 0.05 | |  | | 2.39 | | | 0.37 | | 2.12 | 0.41 | | | > 0.05 |  |
| **DG(16:0_18:1)** | 1.91 | 0.61 | 1.70 | 0.41 | > 0.05 | |  | | 2.27 | | | 0.51 | | 1.50 | 0.47 | | | > 0.05 |  |
| **DG(16:0_18:2)** | 2.49 | 0.34 | 3.27 | 0.38 | > 0.05 | |  | | 3.25 | | | 0.43 | | 2.65 | 0.32 | | | > 0.05 |  |
| **DG(16:0_20:0)** | 0.00 | 0.00 | 0.00 | 0.00 | > 0.05 | |  | | 0.00 | | | 0.00 | | 0.00 | 0.00 | | | > 0.05 |  |
| **DG(16:0_20:3)** | 0.47 | 0.08 | 0.56 | 0.07 | > 0.05 | |  | | 0.39 | | | 0.04 | | 0.38 | 0.06 | | | > 0.05 |  |
| **DG(16:0_20:4)** | 1.25 | 0.21 | 1.16 | 0.13 | > 0.05 | |  | | 1.08 | | | 0.18 | | 0.83 | 0.16 | | | > 0.05 |  |
| **DG(16:1_18:0)** | 0.30 | 0.04 | 0.33 | 0.04 | > 0.05 | |  | | 0.29 | | | 0.04 | | 0.27 | 0.04 | | | > 0.05 |  |
| **DG(16:1_18:1)** | 0.73 | 0.15 | 1.13 | 0.20 | > 0.05 | |  | | 0.73 | | | 0.16 | | 0.74 | 0.16 | | | > 0.05 |  |
| **DG(16:1_18:2)** | 2.61 | 0.37 | 3.29 | 0.43 | > 0.05 | |  | | 2.41 | | | 0.30 | | 2.16 | 0.29 | | | > 0.05 |  |
| **DG(17:0_17:1)** | 0.12 | 0.01 | 0.16 | 0.02 | > 0.05 | |  | | 0.12 | | | 0.01 | | 0.13 | 0.02 | | | > 0.05 |  |
| **DG(17:0_18:1)** | 0.41 | 0.05 | 0.37 | 0.04 | > 0.05 | |  | | 0.45 | | | 0.05 | | 0.38 | 0.05 | | | > 0.05 |  |
| **DG(18:0_20:4)** | 0.54 | 0.07 | 0.63 | 0.08 | > 0.05 | |  | | 0.63 | | | 0.09 | | 0.49 | 0.07 | | | > 0.05 |  |
| **DG(18:1_18:1)** | 0.47 | 0.06 | 0.55 | 0.07 | > 0.05 | |  | | 0.53 | | | 0.07 | | 0.42 | 0.07 | | | > 0.05 |  |
| **DG(18:1_18:2)** | 0.89 | 0.13 | 1.01 | 0.15 | > 0.05 | |  | | 0.84 | | | 0.10 | | 0.69 | 0.10 | | | > 0.05 |  |
| **DG(18:1_18:3)** | 0.42 | 0.10 | 0.49 | 0.10 | > 0.05 | |  | | 0.37 | | | 0.10 | | 0.27 | 0.08 | | | > 0.05 |  |
| **DG(18:1_18:4)** | 0.36 | 0.06 | 0.48 | 0.06 | > 0.05 | |  | | 0.44 | | | 0.07 | | 0.31 | 0.04 | | | > 0.05 |  |
| **DG(18:1_20:1)** | 0.04 | 0.00 | 0.04 | 0.00 | > 0.05 | |  | | 0.03 | | | 0.00 | | 0.04 | 0.00 | | | > 0.05 |  |
| **DG(18:1_20:2)** | 0.64 | 0.07 | 0.80 | 0.10 | > 0.05 | |  | | 0.64 | | | 0.09 | | 0.51 | 0.07 | | | > 0.05 |  |
| **DG(18:1_20:3)** | 0.12 | 0.02 | 0.10 | 0.01 | > 0.05 | |  | | 0.09 | | | 0.01 | | 0.10 | 0.01 | | | > 0.05 |  |
| **DG(18:1_20:4)** | 0.42 | 0.06 | 0.47 | 0.07 | > 0.05 | |  | | 0.38 | | | 0.06 | | 0.32 | 0.04 | | | > 0.05 |  |
| **DG(18:1_22:5)** | 0.02 | 0.00 | 0.02 | 0.00 | > 0.05 | |  | | 0.01 | | | 0.00 | | 0.01 | 0.00 | | | > 0.05 |  |
| **DG(18:1_22:6)** | 0.16 | 0.03 | 0.19 | 0.04 | > 0.05 | |  | | 0.13 | | | 0.03 | | 0.12 | 0.03 | | | > 0.05 |  |
| **DG(18:2_18:2)** | 3.12 | 0.39 | 3.48 | 0.40 | > 0.05 | |  | | 3.22 | | | 0.52 | | 3.18 | 0.49 | | | > 0.05 |  |
| **DG(18:2_18:3)** | 1.72 | 0.23 | 1.84 | 0.24 | > 0.05 | |  | | 1.52 | | | 0.22 | | 1.18 | 0.16 | | | > 0.05 |  |
| **DG(18:2_18:4)** | 0.95 | 0.16 | 1.14 | 0.15 | > 0.05 | |  | | 0.84 | | | 0.13 | | 0.73 | 0.12 | | | > 0.05 |  |
| **DG(18:2_20:0)** | 0.71 | 0.09 | 0.72 | 0.11 | > 0.05 | |  | | 0.79 | | | 0.10 | | 0.64 | 0.07 | | | > 0.05 |  |
| **DG(18:2_20:4)** | 1.38 | 0.19 | 1.47 | 0.19 | > 0.05 | |  | | 1.02 | | | 0.11 | | 1.04 | 0.16 | | | > 0.05 |  |
| **DG(18:3_18:3)** | 3.69 | 0.53 | 4.54 | 0.52 | > 0.05 | |  | | 3.30 | | | 0.37 | | 3.54 | 0.50 | | | > 0.05 |  |
| **DG(18:3_20:2)** | 0.01 | 0.00 | 0.01 | 0.00 | > 0.05 | |  | | 0.01 | | | 0.00 | | 0.00 | 0.00 | | | > 0.05 |  |
| **DG(21:0_22:6)** | 0.00 | 0.00 | 0.01 | 0.00 | > 0.05 | |  | | 0.01 | | | 0.00 | | 0.00 | 0.00 | | | > 0.05 |  |
| **DG(22:1_22:2)** | 0.03 | 0.01 | 0.03 | 0.01 | > 0.05 | |  | | 0.04 | | | 0.01 | | 0.05 | 0.01 | | | > 0.05 |  |
| **DG-O(14:0_18:2)** | 0.00 | 0.00 | 0.00 | 0.00 | > 0.05 | |  | | 0.00 | | | 0.00 | | 0.00 | 0.00 | | | > 0.05 |  |
| **DG-O(16:0_20:4)** | 0.72 | 0.05 | 0.62 | 0.06 | > 0.05 | |  | | 0.59 | | | 0.06 | | 0.62 | 0.05 | | | > 0.05 |  |
| **DG-O(18:2_18:2)** | 0.24 | 0.02 | 0.23 | 0.02 | > 0.05 | |  | | 0.23 | | | 0.02 | | 0.25 | 0.02 | | | > 0.05 |  |

**Supplementary Table S14.**Biochemical abbreviation and Plasma concentration (µM) of Dihydroceramides in low (LRFI) and high (HRFI) Residual Feed Intake bulls on Day 0 and 56 of the feed efficiency trial (Mean ± SEM).

|  |  |  | **Day 0** |  | |  | |  | |  |  | | **Day 56** | |  | | |  | |
| --- | --- | --- | --- | --- | --- | --- | --- | --- | --- | --- | --- | --- | --- | --- | --- | --- | --- | --- | --- |
|  |  | **LRFI** |  | **HRFI** | |  | |  | |  | **LRFI** | |  | | **HRFI** | | |  | |
| **COMPOUND** | **Mean** | **SEM** | **Mean** | **SEM** | | ***P*** | |  | | **Mean** | **SEM** | | **Mean** | | **SEM** | | | ***P*** | |
| **Cer(d18:0/16:0)** | 83.46 | 4.73 | 82.53 | 4.41 | > 0.05 | |  | | 66.18 | | | 5.02 | | 67.81 | | 5.52 | > 0.05 | |  |
| **Cer(d18:0/18:0(OH))** | 0.62 | 0.04 | 0.60 | 0.04 | > 0.05 | |  | | 0.70 | | | 0.04 | | 0.56 | | 0.04 | 0.02 | |  |
| **Cer(d18:0/18:0)** | 0.03 | 0.01 | 0.04 | 0.01 | > 0.05 | |  | | 0.04 | | | 0.01 | | 0.03 | | 0.01 | > 0.05 | |  |
| **Cer(d18:0/20:0)** | 0.01 | 0.00 | 0.01 | 0.00 | > 0.05 | |  | | 0.02 | | | 0.01 | | 0.02 | | 0.01 | > 0.05 | |  |
| **Cer(d18:0/22:0)** | 0.14 | 0.01 | 0.15 | 0.01 | > 0.05 | |  | | 0.15 | | | 0.01 | | 0.13 | | 0.01 | > 0.05 | |  |
| **Cer(d18:0/24:0)** | 0.30 | 0.03 | 0.31 | 0.02 | > 0.05 | |  | | 0.32 | | | 0.03 | | 0.28 | | 0.02 | > 0.05 | |  |
| **Cer(d18:0/24:1)** | 0.38 | 0.03 | 0.38 | 0.02 | > 0.05 | |  | | 0.39 | | | 0.02 | | 0.37 | | 0.03 | > 0.05 | |  |
| **Cer(d18:0/26:1(OH))** | 1.55 | 0.11 | 1.68 | 0.10 | > 0.05 | |  | | 1.55 | | | 0.11 | | 1.56 | | 0.11 | > 0.05 | |  |

**Supplementary Table S15.**Biochemical abbreviation and Plasma concentration (µM) of Fatty Acids in low (LRFI) and high (HRFI) Residual Feed Intake bulls on Day 0 and 56 of the feed efficiency trial (Mean ± SEM).

|  |  |  | **Day 0** | |  | |  | |  | | |  |  | | **Day 56** | |  | |  | |  |
| --- | --- | --- | --- | --- | --- | --- | --- | --- | --- | --- | --- | --- | --- | --- | --- | --- | --- | --- | --- | --- | --- |
|  |  | **LRFI** |  | | **HRFI** | |  | |  | | |  | **LRFI** | |  | | **HRFI** | |  | |  |
| **COMPOUND** | **Mean** | **SEM** | **Mean** | | **SEM** | | ***P*** | |  | | | **Mean** | **SEM** | | **Mean** | | **SEM** | | ***P*** | |  |
| **AA** | 0.57 | 0.13 | | 0.63 | | 0.13 | | > 0.05 | |  | 0.97 | | | 0.13 | | 0.91 | | 0.14 | | > 0.05 | |
| **DHA** | 0.70 | 0.05 | | 0.65 | | 0.06 | | > 0.05 | |  | 0.58 | | | 0.04 | | 0.58 | | 0.05 | | > 0.05 | |
| **EPA** | 0.16 | 0.02 | | 0.15 | | 0.02 | | > 0.05 | |  | 0.13 | | | 0.02 | | 0.14 | | 0.02 | | > 0.05 | |
| **FA(12:0)** | 810.55 | 302.79 | | 884.21 | | 443.5 | | > 0.05 | |  | 988.88 | | | 458.47 | | 391.63 | | 113.21 | | > 0.05 | |
| **FA(14:0)** | 2049.60 | 831.84 | | 1090.7 | | 224.9 | | > 0.05 | |  | 2517.62 | | | 933.76 | | 897.20 | | 461.27 | | 0.02 | |
| **FA(16:0)** | 196.97 | 4.13 | | 196.48 | | 4.15 | | > 0.05 | |  | 201.62 | | | 4.05 | | 198.15 | | 5.17 | | > 0.05 | |
| **FA(18:0)** | 151.96 | 4.08 | | 157.68 | | 5.65 | | > 0.05 | |  | 169.55 | | | 5.89 | | 159.04 | | 6.19 | | > 0.05 | |
| **FA(18:1)** | 52.92 | 4.64 | | 45.49 | | 3.50 | | > 0.05 | |  | 40.49 | | | 4.48 | | 41.81 | | 4.75 | | > 0.05 | |
| **FA(18:2)** | 8.03 | 0.44 | | 7.78 | | 0.40 | | > 0.05 | |  | 8.26 | | | 0.49 | | 8.02 | | 0.52 | | > 0.05 | |
| **FA(20:1)** | 0.78 | 0.08 | | 0.87 | | 0.11 | | > 0.05 | |  | 0.87 | | | 0.09 | | 0.88 | | 0.09 | | > 0.05 | |
| **FA(20:2)** | 0.67 | 0.09 | | 0.79 | | 0.08 | | > 0.05 | |  | 0.62 | | | 0.07 | | 0.70 | | 0.10 | | > 0.05 | |
| **FA(20:3)** | 0.20 | 0.04 | | 0.28 | | 0.05 | | > 0.05 | |  | 0.32 | | | 0.05 | | 0.33 | | 0.05 | | > 0.05 | |

**Supplementary Table S16.**Biochemical abbreviation and Plasma concentration (µM) of Glycerophospholipids in low (LRFI) and high (HRFI) Residual Feed Intake bulls on Day 0 and 56 of the feed efficiency trial (Mean ± SEM).

|  |  |  | **Day 0** |  | |  | |  | |  | |  | | **Day 56** | |  | |  | |
| --- | --- | --- | --- | --- | --- | --- | --- | --- | --- | --- | --- | --- | --- | --- | --- | --- | --- | --- | --- |
|  |  | **LRFI** |  | **HRFI** | |  | |  | |  | | **LRFI** | |  | | **HRFI** | |  | |
| **COMPOUND** | **Mean** | **SEM** | **Mean** | **SEM** | | ***P*** | |  | | **Mean** | | **SEM** | | **Mean** | | **SEM** | | ***P*** | |
| **lysoPC a C14:0** | 3.10 | 0.13 | 3.21 | 0.14 | > 0.05 | |  | | 3.09 | | 0.12 | | 3.34 | | 0.14 | | > 0.05 | |  |
| **lysoPC a C16:0** | 22.65 | 1.51 | 21.75 | 1.60 | > 0.05 | |  | | 24.22 | | 1.48 | | 22.63 | | 1.80 | | > 0.05 | |  |
| **lysoPC a C16:1** | 1.14 | 0.06 | 1.18 | 0.05 | > 0.05 | |  | | 1.25 | | 0.06 | | 1.22 | | 0.07 | | > 0.05 | |  |
| **lysoPC a C17:0** | 3.52 | 0.27 | 4.12 | 0.37 | > 0.05 | |  | | 4.18 | | 0.32 | | 4.39 | | 0.35 | | > 0.05 | |  |
| **lysoPC a C18:0** | 27.11 | 2.00 | 26.06 | 2.05 | > 0.05 | |  | | 29.82 | | 2.06 | | 28.01 | | 2.56 | | > 0.05 | |  |
| **lysoPC a C18:1** | 10.12 | 0.66 | 9.83 | 0.60 | > 0.05 | |  | | 10.92 | | 0.75 | | 10.89 | | 0.83 | | > 0.05 | |  |
| **lysoPC a C18:2** | 25.80 | 1.90 | 23.33 | 1.94 | > 0.05 | |  | | 27.21 | | 2.43 | | 25.72 | | 2.69 | | > 0.05 | |  |
| **lysoPC a C20:3** | 1.05 | 0.06 | 1.04 | 0.06 | > 0.05 | |  | | 1.01 | | 0.06 | | 0.98 | | 0.07 | | > 0.05 | |  |
| **lysoPC a C20:4** | 1.32 | 0.09 | 1.16 | 0.06 | > 0.05 | |  | | 1.37 | | 0.08 | | 1.33 | | 0.11 | | > 0.05 | |  |
| **lysoPC a C24:0** | 0.13 | 0.01 | 0.11 | 0.01 | > 0.05 | |  | | 0.11 | | 0.01 | | 0.11 | | 0.01 | | > 0.05 | |  |
| **lysoPC a C26:0** | 0.13 | 0.01 | 0.13 | 0.01 | > 0.05 | |  | | 0.15 | | 0.01 | | 0.18 | | 0.01 | | > 0.05 | |  |
| **lysoPC a C26:1** | 0.12 | 0.01 | 0.13 | 0.01 | > 0.05 | |  | | 0.16 | | 0.01 | | 0.16 | | 0.01 | | > 0.05 | |  |
| **lysoPC a C28:0** | 0.48 | 0.04 | 0.45 | 0.04 | > 0.05 | |  | | 0.44 | | 0.04 | | 0.41 | | 0.03 | | > 0.05 | |  |
| **lysoPC a C28:1** | 0.37 | 0.02 | 0.34 | 0.03 | > 0.05 | |  | | 0.37 | | 0.02 | | 0.41 | | 0.03 | | > 0.05 | |  |
| **PC aa C24:0** | 0.03 | 0.00 | 0.03 | 0.00 | > 0.05 | |  | | 0.04 | | 0.00 | | 0.03 | | 0.00 | | > 0.05 | |  |
| **PC aa C26:0** | 0.26 | 0.01 | 0.25 | 0.01 | > 0.05 | |  | | 0.26 | | 0.01 | | 0.26 | | 0.01 | | > 0.05 | |  |
| **PC aa C28:1** | 1.40 | 0.08 | 1.31 | 0.12 | > 0.05 | |  | | 1.51 | | 0.08 | | 1.52 | | 0.11 | | > 0.05 | |  |
| **PC aa C30:0** | 2.68 | 0.11 | 2.56 | 0.17 | > 0.05 | |  | | 3.01 | | 0.15 | | 2.93 | | 0.17 | | > 0.05 | |  |
| **PC aa C32:0** | 11.78 | 0.75 | 10.73 | 0.80 | > 0.05 | |  | | 11.37 | | 0.67 | | 10.93 | | 0.78 | | > 0.05 | |  |
| **PC aa C32:1** | 6.60 | 0.45 | 6.60 | 0.55 | > 0.05 | |  | | 6.84 | | 0.59 | | 6.92 | | 0.50 | | > 0.05 | |  |
| **PC aa C32:2** | 11.28 | 1.20 | 9.87 | 1.20 | > 0.05 | |  | | 10.50 | | 1.06 | | 11.03 | | 1.21 | | > 0.05 | |  |
| **PC aa C32:3** | 82.46 | 9.04 | 72.62 | 11.47 | > 0.05 | |  | | 68.53 | | 7.71 | | 80.02 | | 9.89 | | > 0.05 | |  |
| **PC aa C34:1** | 71.99 | 5.29 | 65.64 | 5.57 | > 0.05 | |  | | 72.83 | | 5.62 | | 73.25 | | 5.75 | | > 0.05 | |  |
| **PC aa C34:2** | 294.90 | 20.90 | 253.09 | 24.69 | > 0.05 | |  | | 272.68 | | 20.06 | | 265.36 | | 22.13 | | > 0.05 | |  |
| **PC aa C34:3** | 21.04 | 1.60 | 20.11 | 2.00 | > 0.05 | |  | | 20.66 | | 1.76 | | 19.90 | | 1.77 | | > 0.05 | |  |
| **PC aa C34:4** | 7.12 | 0.69 | 7.12 | 0.94 | > 0.05 | |  | | 7.35 | | 0.74 | | 7.39 | | 0.76 | | > 0.05 | |  |
| **PC aa C36:0** | 1.03 | 0.20 | 1.19 | 0.16 | > 0.05 | |  | | 1.42 | | 0.30 | | 0.65 | | 0.14 | | > 0.05 | |  |
| **PC aa C36:1** | 153.92 | 12.52 | 132.51 | 14.31 | > 0.05 | |  | | 146.40 | | 12.70 | | 156.95 | | 13.76 | | > 0.05 | |  |
| **PC aa C36:2** | 300.62 | 25.71 | 262.30 | 21.77 | > 0.05 | |  | | 324.85 | | 27.71 | | 278.48 | | 24.53 | | > 0.05 | |  |
| **PC aa C36:3** | 123.45 | 9.24 | 127.81 | 11.04 | > 0.05 | |  | | 135.48 | | 11.77 | | 125.28 | | 10.21 | | > 0.05 | |  |
| **PC aa C36:4** | 32.85 | 2.61 | 30.64 | 3.13 | > 0.05 | |  | | 35.13 | | 3.13 | | 28.73 | | 2.06 | | > 0.05 | |  |
| **PC aa C36:5** | 5.31 | 0.39 | 4.71 | 0.45 | > 0.05 | |  | | 5.00 | | 0.36 | | 4.89 | | 0.40 | | > 0.05 | |  |
| **PC aa C36:6** | 2.92 | 0.25 | 2.49 | 0.26 | > 0.05 | |  | | 2.87 | | 0.22 | | 3.17 | | 0.32 | | > 0.05 | |  |
| **PC aa C38:0** | 0.79 | 0.06 | 0.69 | 0.06 | > 0.05 | |  | | 0.92 | | 0.08 | | 0.71 | | 0.07 | | 0.03 | |  |
| **PC aa C38:3** | 34.06 | 2.77 | 32.82 | 3.18 | > 0.05 | |  | | 35.45 | | 3.15 | | 38.20 | | 3.08 | | > 0.05 | |  |
| **PC aa C38:4** | 40.59 | 2.75 | 39.33 | 3.29 | > 0.05 | |  | | 48.65 | | 3.77 | | 41.51 | | 3.23 | | > 0.05 | |  |
| **PC aa C38:5** | 13.30 | 0.82 | 11.99 | 0.86 | > 0.05 | |  | | 13.14 | | 0.91 | | 12.10 | | 0.85 | | > 0.05 | |  |
| **PC aa C38:6** | 3.41 | 0.18 | 3.15 | 0.23 | > 0.05 | |  | | 3.32 | | 0.23 | | 3.38 | | 0.24 | | > 0.05 | |  |
| **PC aa C40:1** | 0.02 | 0.00 | 0.03 | 0.00 | > 0.05 | |  | | 0.03 | | 0.00 | | 0.02 | | 0.00 | | > 0.05 | |  |
| **PC aa C40:2** | 0.24 | 0.03 | 0.21 | 0.03 | > 0.05 | |  | | 0.22 | | 0.03 | | 0.25 | | 0.02 | | > 0.05 | |  |
| **PC aa C40:3** | 0.51 | 0.05 | 0.57 | 0.05 | > 0.05 | |  | | 0.57 | | 0.07 | | 0.58 | | 0.05 | | > 0.05 | |  |
| **PC aa C40:4** | 6.35 | 0.41 | 6.46 | 0.38 | > 0.05 | |  | | 7.38 | | 0.53 | | 7.33 | | 0.50 | | > 0.05 | |  |
| **PC aa C40:5** | 13.27 | 0.91 | 12.02 | 0.76 | > 0.05 | |  | | 14.81 | | 0.93 | | 12.71 | | 0.87 | | > 0.05 | |  |
| **PC aa C40:6** | 4.99 | 0.35 | 4.37 | 0.30 | > 0.05 | |  | | 5.62 | | 0.41 | | 4.69 | | 0.36 | | > 0.05 | |  |
| **PC aa C42:0** | 0.08 | 0.01 | 0.10 | 0.01 | 0.03 | |  | | 0.09 | | 0.01 | | 0.10 | | 0.01 | | > 0.05 | |  |
| **PC aa C42:1** | 0.15 | 0.01 | 0.15 | 0.01 | > 0.05 | |  | | 0.17 | | 0.01 | | 0.15 | | 0.01 | | > 0.05 | |  |
| **PC aa C42:2** | 0.40 | 0.03 | 0.36 | 0.03 | > 0.05 | |  | | 0.45 | | 0.04 | | 0.39 | | 0.03 | | > 0.05 | |  |
| **PC aa C42:4** | 0.13 | 0.01 | 0.12 | 0.01 | > 0.05 | |  | | 0.13 | | 0.01 | | 0.13 | | 0.01 | | > 0.05 | |  |
| **PC aa C42:5** | 0.22 | 0.02 | 0.19 | 0.02 | > 0.05 | |  | | 0.23 | | 0.02 | | 0.20 | | 0.02 | | > 0.05 | |  |
| **PC aa C42:6** | 0.29 | 0.03 | 0.30 | 0.04 | > 0.05 | |  | | 0.34 | | 0.04 | | 0.37 | | 0.03 | | > 0.05 | |  |
| **PC ae C30:0** | 0.62 | 0.03 | 0.61 | 0.03 | > 0.05 | |  | | 0.66 | | 0.03 | | 0.68 | | 0.04 | | > 0.05 | |  |
| **PC ae C30:1** | 1.43 | 0.16 | 2.06 | 0.26 | > 0.05 | |  | | 1.96 | | 0.19 | | 2.04 | | 0.24 | | > 0.05 | |  |
| **PC ae C30:2** | 0.30 | 0.02 | 0.33 | 0.03 | > 0.05 | |  | | 0.37 | | 0.02 | | 0.37 | | 0.03 | | > 0.05 | |  |
| **PC ae C32:1** | 5.00 | 0.42 | 4.69 | 0.51 | > 0.05 | |  | | 5.19 | | 0.43 | | 5.45 | | 0.43 | | > 0.05 | |  |
| **PC ae C32:2** | 7.52 | 0.63 | 7.66 | 0.96 | > 0.05 | |  | | 9.77 | | 0.88 | | 9.27 | | 0.86 | | > 0.05 | |  |
| **PC ae C34:0** | 6.55 | 0.55 | 5.26 | 0.51 | > 0.05 | |  | | 6.46 | | 0.44 | | 6.55 | | 0.50 | | > 0.05 | |  |
| **PC ae C34:1** | 18.43 | 1.25 | 16.08 | 1.34 | > 0.05 | |  | | 18.14 | | 1.37 | | 18.22 | | 1.37 | | > 0.05 | |  |
| **PC ae C34:2** | 35.39 | 3.02 | 30.13 | 3.25 | > 0.05 | |  | | 33.27 | | 2.63 | | 34.47 | | 2.63 | | > 0.05 | |  |
| **PC ae C34:3** | 47.55 | 5.34 | 42.89 | 5.95 | > 0.05 | |  | | 45.55 | | 4.85 | | 47.57 | | 4.54 | | > 0.05 | |  |
| **PC ae C36:0** | 3.41 | 0.32 | 3.08 | 0.33 | > 0.05 | |  | | 3.53 | | 0.27 | | 3.48 | | 0.30 | | > 0.05 | |  |
| **PC ae C36:1** | 26.07 | 2.11 | 22.98 | 2.02 | > 0.05 | |  | | 27.41 | | 2.51 | | 26.54 | | 2.27 | | > 0.05 | |  |
| **PC ae C36:2** | 95.31 | 9.11 | 82.77 | 8.71 | > 0.05 | |  | | 86.50 | | 6.82 | | 96.37 | | 8.61 | | > 0.05 | |  |
| **PC ae C36:3** | 15.46 | 1.21 | 13.36 | 1.29 | > 0.05 | |  | | 15.43 | | 1.16 | | 14.14 | | 1.03 | | > 0.05 | |  |
| **PC ae C36:4** | 8.81 | 0.71 | 8.86 | 0.96 | > 0.05 | |  | | 10.06 | | 0.86 | | 9.24 | | 0.67 | | > 0.05 | |  |
| **PC ae C36:5** | 6.22 | 0.51 | 5.86 | 0.58 | > 0.05 | |  | | 6.48 | | 0.53 | | 6.23 | | 0.44 | | > 0.05 | |  |
| **PC ae C38:0** | 0.87 | 0.07 | 0.71 | 0.07 | > 0.05 | |  | | 0.84 | | 0.07 | | 0.84 | | 0.07 | | > 0.05 | |  |
| **PC ae C38:1** | 3.05 | 0.31 | 3.12 | 0.44 | > 0.05 | |  | | 3.63 | | 0.28 | | 3.60 | | 0.28 | | > 0.05 | |  |
| **PC ae C38:2** | 10.01 | 0.84 | 9.59 | 0.98 | > 0.05 | |  | | 10.08 | | 0.85 | | 10.11 | | 0.89 | | > 0.05 | |  |
| **PC ae C38:3** | 7.28 | 0.62 | 7.24 | 0.65 | > 0.05 | |  | | 7.85 | | 0.70 | | 7.96 | | 0.66 | | > 0.05 | |  |
| **PC ae C38:4** | 7.95 | 0.62 | 7.11 | 0.59 | > 0.05 | |  | | 7.51 | | 0.51 | | 7.71 | | 0.58 | | > 0.05 | |  |
| **PC ae C38:5** | 5.13 | 0.40 | 4.43 | 0.38 | > 0.05 | |  | | 5.18 | | 0.42 | | 4.84 | | 0.32 | | > 0.05 | |  |
| **PC ae C38:6** | 3.44 | 0.26 | 3.05 | 0.26 | > 0.05 | |  | | 3.26 | | 0.24 | | 3.66 | | 0.32 | | > 0.05 | |  |
| **PC ae C40:1** | 0.18 | 0.02 | 0.14 | 0.02 | > 0.05 | |  | | 0.17 | | 0.03 | | 0.19 | | 0.04 | | > 0.05 | |  |
| **PC ae C40:2** | 1.00 | 0.06 | 0.96 | 0.07 | > 0.05 | |  | | 0.99 | | 0.06 | | 1.08 | | 0.07 | | > 0.05 | |  |
| **PC ae C40:3** | 1.31 | 0.11 | 1.37 | 0.10 | > 0.05 | |  | | 1.37 | | 0.09 | | 1.49 | | 0.11 | | > 0.05 | |  |
| **PC ae C40:4** | 1.77 | 0.16 | 1.91 | 0.13 | > 0.05 | |  | | 2.03 | | 0.14 | | 1.91 | | 0.12 | | > 0.05 | |  |
| **PC ae C40:5** | 3.25 | 0.21 | 2.87 | 0.22 | > 0.05 | |  | | 3.26 | | 0.19 | | 3.25 | | 0.23 | | > 0.05 | |  |
| **PC ae C40:6** | 1.37 | 0.08 | 1.31 | 0.11 | > 0.05 | |  | | 1.38 | | 0.10 | | 1.34 | | 0.10 | | > 0.05 | |  |
| **PC ae C42:0** | 0.03 | 0.00 | 0.04 | 0.00 | > 0.05 | |  | | 0.03 | | 0.00 | | 0.04 | | 0.00 | | 0.02 | |  |
| **PC ae C42:1** | 0.17 | 0.01 | 0.18 | 0.01 | > 0.05 | |  | | 0.18 | | 0.01 | | 0.17 | | 0.01 | | > 0.05 | |  |
| **PC ae C42:2** | 0.23 | 0.02 | 0.20 | 0.02 | > 0.05 | |  | | 0.25 | | 0.02 | | 0.24 | | 0.02 | | > 0.05 | |  |
| **PC ae C42:3** | 0.17 | 0.02 | 0.14 | 0.01 | > 0.05 | |  | | 0.19 | | 0.02 | | 0.18 | | 0.01 | | > 0.05 | |  |
| **PC ae C42:4** | 0.02 | 0.00 | 0.03 | 0.00 | 0.003 | |  | | 0.02 | | 0.00 | | 0.02 | | 0.00 | | > 0.05 | |  |
| **PC ae C42:5** | 0.23 | 0.07 | 0.32 | 0.09 | > 0.05 | |  | | 0.28 | | 0.09 | | 0.36 | | 0.09 | | > 0.05 | |  |
| **PC ae C44:3** | 0.13 | 0.01 | 0.12 | 0.01 | > 0.05 | |  | | 0.15 | | 0.01 | | 0.14 | | 0.01 | | > 0.05 | |  |
| **PC ae C44:4** | 0.10 | 0.01 | 0.10 | 0.01 | > 0.05 | |  | | 0.09 | | 0.02 | | 0.07 | | 0.01 | | > 0.05 | |  |
| **PC ae C44:5** | 0.10 | 0.01 | 0.10 | 0.01 | > 0.05 | |  | | 0.11 | | 0.01 | | 0.11 | | 0.01 | | > 0.05 | |  |
| **PC ae C44:6** | 0.02 | 0.00 | 0.02 | 0.00 | > 0.05 | |  | | 0.02 | | 0.00 | | 0.02 | | 0.00 | | > 0.05 | |  |

**Supplementary Table S17.**Biochemical abbreviation and Plasma concentration (µM) of Glycosylceramides in low (LRFI) and high (HRFI) Residual Feed Intake bulls on Day 0 and 56 of the feed efficiency trial (Mean ± SEM).

|  |  |  | **Day 0** |  | |  | |  | |  |  | | **Day 56** | | |  |  | |  |
| --- | --- | --- | --- | --- | --- | --- | --- | --- | --- | --- | --- | --- | --- | --- | --- | --- | --- | --- | --- |
|  |  | **LRFI** |  | **HRFI** | |  | |  | |  | **LRFI** | |  | | | **HRFI** |  | |  |
| **COMPOUND** | **Mean** | **SEM** | **Mean** | **SEM** | | ***P*** | |  | | **Mean** | **SEM** | | **Mean** | | | **SEM** | ***P*** | |  |
| **Hex2Cer(d18:1/14:0)** | 0.06 | 0.01 | 0.06 | 0.01 | > 0.05 | |  | | 0.06 | | | 0.01 | | 0.06 | 0.01 | | | > 0.05 | |
| **Hex2Cer(d18:1/16:0)** | 0.48 | 0.04 | 0.51 | 0.04 | > 0.05 | |  | | 0.51 | | | 0.04 | | 0.48 | 0.05 | | | > 0.05 | |
| **Hex2Cer(d18:1/18:0)** | 0.07 | 0.01 | 0.06 | 0.01 | > 0.05 | |  | | 0.06 | | | 0.01 | | 0.05 | 0.01 | | | > 0.05 | |
| **Hex2Cer(d18:1/20:0)** | 0.01 | 0.00 | 0.01 | 0.00 | > 0.05 | |  | | 0.01 | | | 0.00 | | 0.01 | 0.00 | | | > 0.05 | |
| **Hex2Cer(d18:1/22:0)** | 0.02 | 0.00 | 0.02 | 0.00 | > 0.05 | |  | | 0.02 | | | 0.00 | | 0.02 | 0.00 | | | > 0.05 | |
| **Hex2Cer(d18:1/24:0)** | 0.03 | 0.00 | 0.03 | 0.00 | > 0.05 | |  | | 0.03 | | | 0.00 | | 0.03 | 0.00 | | | > 0.05 | |
| **Hex2Cer(d18:1/24:1)** | 0.02 | 0.00 | 0.03 | 0.00 | > 0.05 | |  | | 0.03 | | | 0.00 | | 0.02 | 0.00 | | | > 0.05 | |
| **Hex2Cer(d18:1/26:0)** | 0.01 | 0.00 | 0.01 | 0.00 | > 0.05 | |  | | 0.01 | | | 0.00 | | 0.01 | 0.00 | | | > 0.05 | |
| **Hex2Cer(d18:1/26:1)** | 0.01 | 0.00 | 0.01 | 0.00 | > 0.05 | |  | | 0.01 | | | 0.00 | | 0.01 | 0.00 | | | > 0.05 | |
| **Hex3Cer(d18:1/16:0)** | 0.19 | 0.02 | 0.16 | 0.02 | > 0.05 | |  | | 0.21 | | | 0.02 | | 0.18 | 0.02 | | | > 0.05 | |
| **Hex3Cer(d18:1/18:0)** | 0.04 | 0.00 | 0.04 | 0.00 | > 0.05 | |  | | 0.03 | | | 0.00 | | 0.04 | 0.00 | | | > 0.05 | |
| **Hex3Cer(d18:1/24:1)** | 0.05 | 0.01 | 0.05 | 0.00 | > 0.05 | |  | | 0.05 | | | 0.00 | | 0.05 | 0.00 | | | > 0.05 | |
| **Hex3Cer(d18:1/26:1)** | 0.01 | 0.00 | 0.01 | 0.00 | > 0.05 | |  | | 0.01 | | | 0.00 | | 0.01 | 0.00 | | | > 0.05 | |
| **Hex3Cer(d18:1_20:0)** | 0.00 | 0.00 | 0.00 | 0.00 | > 0.05 | |  | | 0.00 | | | 0.00 | | 0.00 | 0.00 | | | > 0.05 | |
| **Hex3Cer(d18:1_22:0)** | 0.02 | 0.00 | 0.02 | 0.00 | > 0.05 | |  | | 0.02 | | | 0.00 | | 0.02 | 0.00 | | | > 0.05 | |
| **HexCer(d16:1/22:0)** | 0.04 | 0.00 | 0.04 | 0.00 | > 0.05 | |  | | 0.04 | | | 0.00 | | 0.04 | 0.00 | | | > 0.05 | |
| **HexCer(d16:1/24:0)** | 0.03 | 0.00 | 0.03 | 0.00 | > 0.05 | |  | | 0.03 | | | 0.00 | | 0.03 | 0.00 | | | > 0.05 | |
| **HexCer(d18:1/14:0)** | 0.02 | 0.00 | 0.03 | 0.00 | > 0.05 | |  | | 0.02 | | | 0.00 | | 0.03 | 0.00 | | | > 0.05 | |
| **HexCer(d18:1/16:0)** | 0.25 | 0.02 | 0.26 | 0.03 | > 0.05 | |  | | 0.27 | | | 0.03 | | 0.24 | 0.03 | | | > 0.05 | |
| **HexCer(d18:1/18:0)** | 0.06 | 0.01 | 0.07 | 0.01 | > 0.05 | |  | | 0.05 | | | 0.01 | | 0.05 | 0.01 | | | > 0.05 | |
| **HexCer(d18:1/18:1)** | 0.17 | 0.01 | 0.17 | 0.01 | > 0.05 | |  | | 0.19 | | | 0.01 | | 0.19 | 0.01 | | | > 0.05 | |
| **HexCer(d18:1/20:0)** | 0.20 | 0.01 | 0.18 | 0.01 | > 0.05 | |  | | 0.21 | | | 0.01 | | 0.21 | 0.01 | | | > 0.05 | |
| **HexCer(d18:1/22:0)** | 0.75 | 0.05 | 0.68 | 0.05 | > 0.05 | |  | | 0.62 | | | 0.06 | | 0.62 | 0.05 | | | > 0.05 | |
| **HexCer(d18:1/23:0)** | 0.35 | 0.03 | 0.29 | 0.03 | > 0.05 | |  | | 0.33 | | | 0.03 | | 0.30 | 0.03 | | | > 0.05 | |
| **HexCer(d18:1/24:0)** | 0.19 | 0.02 | 0.19 | 0.02 | > 0.05 | |  | | 0.16 | | | 0.02 | | 0.19 | 0.01 | | | > 0.05 | |
| **HexCer(d18:1/24:1)** | 0.84 | 0.08 | 0.75 | 0.06 | > 0.05 | |  | | 0.69 | | | 0.06 | | 0.62 | 0.05 | | | > 0.05 | |
| **HexCer(d18:1/26:0)** | 0.07 | 0.01 | 0.07 | 0.00 | > 0.05 | |  | | 0.07 | | | 0.00 | | 0.07 | 0.00 | | | > 0.05 | |
| **HexCer(d18:1/26:1)** | 0.10 | 0.01 | 0.09 | 0.01 | > 0.05 | |  | | 0.10 | | | 0.00 | | 0.09 | 0.01 | | | > 0.05 | |
| **HexCer(d18:2/16:0)** | 0.01 | 0.00 | 0.01 | 0.00 | > 0.05 | |  | | 0.01 | | | 0.00 | | 0.01 | 0.00 | | | > 0.05 | |
| **HexCer(d18:2/18:0)** | 0.03 | 0.00 | 0.03 | 0.00 | > 0.05 | |  | | 0.03 | | | 0.00 | | 0.02 | 0.00 | | | > 0.05 | |
| **HexCer(d18:2/20:0)** | 0.02 | 0.00 | 0.02 | 0.00 | > 0.05 | |  | | 0.02 | | | 0.00 | | 0.02 | 0.00 | | | > 0.05 | |
| **HexCer(d18:2/22:0)** | 0.21 | 0.02 | 0.20 | 0.02 | > 0.05 | |  | | 0.22 | | | 0.01 | | 0.21 | 0.01 | | | > 0.05 | |
| **HexCer(d18:2/23:0)** | 0.14 | 0.01 | 0.12 | 0.01 | > 0.05 | |  | | 0.13 | | | 0.01 | | 0.14 | 0.01 | | | > 0.05 | |
| **HexCer(d18:2/24:0)** | 0.18 | 0.01 | 0.17 | 0.02 | > 0.05 | |  | | 0.16 | | | 0.01 | | 0.16 | 0.01 | | | > 0.05 | |

**Supplementary Table S18.**Biochemical abbreviation and Plasma concentration (µM) of Hormones in low (LRFI) and high (HRFI) Residual Feed Intake bulls on Day 0 and 56 of the feed efficiency trial (Mean ± SEM).

|  |  |  | **Day 0** |  | |  | |  | |  |  | | | **Day 56** |  | | |  |  |
| --- | --- | --- | --- | --- | --- | --- | --- | --- | --- | --- | --- | --- | --- | --- | --- | --- | --- | --- | --- |
|  |  | **LRFI** |  | **HRFI** | |  | |  | |  | **LRFI** | | |  | **HRFI** | | |  |  |
| **COMPOUND** | **Mean** | **SEM** | **Mean** | **SEM** | | ***P*** | |  | | **Mean** | **SEM** | | | **Mean** | **SEM** | | | ***P*** |  |
| **AbsAcid** | 0.01 | 0.00 | 0.02 | 0.00 | > 0.05 | |  | | 0.02 | | | 0.00 | 0.02 | | | 0.00 | > 0.05 | | |
| **Cortisol** | 0.06 | 0.01 | 0.06 | 0.01 | > 0.05 | |  | | 0.06 | | | 0.01 | 0.06 | | | 0.01 | > 0.05 | | |
| **Cortisone** | 0.03 | 0.00 | 0.03 | 0.00 | > 0.05 | |  | | 0.03 | | | 0.00 | 0.04 | | | 0.00 | > 0.05 | | |
| **DHEAS** | 0.02 | 0.00 | 0.02 | 0.00 | > 0.05 | |  | | 0.02 | | | 0.00 | 0.02 | | | 0.00 | > 0.05 | | |

**Supplementary Table S19.**Biochemical abbreviation and Plasma concentration (µM) of Indoles in low (LRFI) and high (HRFI) Residual Feed Intake bulls on Day 0 and 56 of the feed efficiency trial (Mean ± SEM).

|  |  |  | **Day 0** |  | |  | |  | |  |  | | | **Day 56** | |  | |  |  |
| --- | --- | --- | --- | --- | --- | --- | --- | --- | --- | --- | --- | --- | --- | --- | --- | --- | --- | --- | --- |
|  |  | **LRFI** |  | **HRFI** | |  | |  | |  | **LRFI** | | |  | | **HRFI** | |  |  |
| **COMPOUND** | **Mean** | **SEM** | **Mean** | **SEM** | | ***P*** | |  | | **Mean** | **SEM** | | | **Mean** | | **SEM** | | ***P*** |  |
| **3-IAA** | 0.29 | 0.02 | 0.30 | 0.03 | > 0.05 | |  | | 0.29 | | | 0.02 | 0.30 | | 0.02 | | > 0.05 | | |
| **3-IPA** | 0.49 | 0.06 | 0.43 | 0.05 | > 0.05 | |  | | 0.41 | | | 0.06 | 0.44 | | 0.07 | | > 0.05 | | |
| **Ind-SO4** | 2.97 | 0.18 | 3.07 | 0.22 | > 0.05 | |  | | 2.59 | | | 0.20 | 2.69 | | 0.17 | | > 0.05 | | |
| **Indole** | 19.42 | 2.60 | 10.91 | 3.04 | 0.04 | |  | | 12.22 | | | 2.65 | 16.61 | | 4.09 | | > 0.05 | | |

**Supplementary Table S20.**Biochemical abbreviation and Plasma concentration (µM) of Nucleobases Related in low (LRFI) and high (HRFI) Residual Feed Intake bulls on Day 0 and 56 of the feed efficiency trial (Mean ± SEM).

|  |  |  | **Day 0** |  | |  | |  | |  | |  | **Day 56** | |  | |  | |  |
| --- | --- | --- | --- | --- | --- | --- | --- | --- | --- | --- | --- | --- | --- | --- | --- | --- | --- | --- | --- |
|  |  | **LRFI** |  | **HRFI** | |  | |  | |  | | **LRFI** |  | | **HRFI** | |  | |  |
| **COMPOUND** | **Mean** | **SEM** | **Mean** | **SEM** | | ***P*** | |  | | **Mean** | | **SEM** | **Mean** | | **SEM** | | ***P*** | |  |
| **Hypoxanthine** | 0.02 | 0.00 | 0.02 | 0.00 | > 0.05 | |  | | 0.02 | | 0.00 | | | 0.02 | | 0.00 | | > 0.05 | |
| **Xanthine** | 0.07 | 0.00 | 0.07 | 0.00 | > 0.05 | |  | | 0.07 | | 0.00 | | | 0.07 | | 0.00 | | > 0.05 | |

**Supplementary Table S21.**Biochemical abbreviation and Plasma concentration (µM) of Sphingolipids in low (LRFI) and high (HRFI) Residual Feed Intake bulls on Day 0 and 56 of the feed efficiency trial (Mean ± SEM).

|  |  |  | **Day 0** |  | |  | |  | |  | |  | | **Day 56** | |  | |  | |
| --- | --- | --- | --- | --- | --- | --- | --- | --- | --- | --- | --- | --- | --- | --- | --- | --- | --- | --- | --- |
|  |  | **LRFI** |  | **HRFI** | |  | |  | |  | | **LRFI** | |  | | **HRFI** | |  | |
| **COMPOUND** | **Mean** | **SEM** | **Mean** | **SEM** | | ***P*** | |  | | **Mean** | | **SEM** | | **Mean** | | **SEM** | | ***P*** | |
| **SM (OH) C14:1** | 11.74 | 0.46 | 11.14 | 0.61 | > 0.05 | |  | | 11.88 | | 0.46 | | 10.61 | | 0.65 | | > 0.05 | |  |
| **SM (OH) C16:1** | 11.97 | 0.82 | 12.27 | 0.83 | > 0.05 | |  | | 12.12 | | 0.67 | | 11.72 | | 0.89 | | > 0.05 | |  |
| **SM (OH) C22:1** | 10.79 | 0.97 | 8.63 | 1.00 | > 0.05 | |  | | 10.84 | | 0.99 | | 8.87 | | 0.91 | | > 0.05 | |  |
| **SM (OH) C22:2** | 3.95 | 0.32 | 3.23 | 0.28 | > 0.05 | |  | | 4.09 | | 0.36 | | 3.32 | | 0.33 | | > 0.05 | |  |
| **SM (OH) C24:1** | 0.97 | 0.10 | 1.01 | 0.09 | > 0.05 | |  | | 0.94 | | 0.07 | | 0.94 | | 0.10 | | > 0.05 | |  |
| **SM C16:0** | 86.11 | 3.30 | 84.00 | 5.02 | > 0.05 | |  | | 91.34 | | 3.91 | | 81.37 | | 5.15 | | > 0.05 | |  |
| **SM C16:1** | 10.30 | 0.58 | 10.38 | 0.65 | > 0.05 | |  | | 11.10 | | 0.61 | | 10.01 | | 0.70 | | > 0.05 | |  |
| **SM C18:0** | 12.87 | 0.64 | 11.69 | 0.67 | > 0.05 | |  | | 13.83 | | 0.82 | | 11.97 | | 0.88 | | > 0.05 | |  |
| **SM C18:1** | 8.46 | 0.65 | 8.65 | 0.74 | > 0.05 | |  | | 8.65 | | 0.70 | | 8.13 | | 0.83 | | > 0.05 | |  |
| **SM C20:2** | 0.34 | 0.04 | 0.40 | 0.04 | > 0.05 | |  | | 0.32 | | 0.03 | | 0.36 | | 0.03 | | > 0.05 | |  |
| **SM C24:0** | 12.18 | 1.10 | 9.80 | 0.82 | > 0.05 | |  | | 11.85 | | 1.04 | | 10.37 | | 0.98 | | > 0.05 | |  |
| **SM C24:1** | 9.27 | 0.85 | 7.56 | 0.80 | > 0.05 | |  | | 9.02 | | 0.82 | | 7.04 | | 0.68 | | > 0.05 | |  |
| **SM C26:0** | 0.24 | 0.02 | 0.29 | 0.04 | > 0.05 | |  | | 0.26 | | 0.02 | | 0.26 | | 0.04 | | > 0.05 | |  |
| **SM C26:1** | 0.19 | 0.03 | 0.24 | 0.03 | > 0.05 | |  | | 0.26 | | 0.03 | | 0.28 | | 0.03 | | > 0.05 | |  |

**Supplementary Table S22.**Biochemical abbreviation and Plasma concentration (µM) of Sugars in low (LRFI) and high (HRFI) Residual Feed Intake bulls on Day 0 and 56 of the feed efficiency trial (Mean ± SEM).

|  |  |  | **Day 0** |  | |  | |  | |  | |  | | **Day 56** |  | | |  | |
| --- | --- | --- | --- | --- | --- | --- | --- | --- | --- | --- | --- | --- | --- | --- | --- | --- | --- | --- | --- |
|  |  | **LRFI** |  | **HRFI** | |  | |  | |  | | **LRFI** | |  | **HRFI** | | |  | |
| **COMPOUND** | **Mean** | **SEM** | **Mean** | **SEM** | | ***P*** | |  | | **Mean** | | **SEM** | | **Mean** | **SEM** | | | ***P*** | |
| **H1** | 2500.9 | 296.5 | 2491.9 | 305.4 | > 0.05 | |  | | 2349.4 | | 335.8 | | 2492.5 | | | 448.5 | > 0.05 | |  |

**Supplementary Table S23.**Biochemical abbreviation and Plasma concentration (µM) of Triacylglycerols in low (LRFI) and high (HRFI) Residual Feed Intake bulls on Day 0 and 56 of the feed efficiency trial (Mean ± SEM).

|  |  |  | **Day 0** |  | |  | |  | |  | |  | **Day 56** | | |  |  | | |
| --- | --- | --- | --- | --- | --- | --- | --- | --- | --- | --- | --- | --- | --- | --- | --- | --- | --- | --- | --- |
|  |  | **LRFI** |  | **HRFI** | |  | |  | |  | | **LRFI** |  | | | **HRFI** |  | | |
| **COMPOUND** | **Mean** | **SEM** | **Mean** | **SEM** | | ***P*** | |  | | **Mean** | | **SEM** | **Mean** | | | **SEM** | ***P*** | | |
| **TG(14:0_32:2)** | 0.71 | 0.08 | 0.71 | 0.08 | > 0.05 | |  | | 0.83 | | 0.09 | | | 0.94 | 0.12 | | | > 0.05 |  |
| **TG(14:0_34:0)** | 1.32 | 0.15 | 1.36 | 0.15 | > 0.05 | |  | | 1.62 | | 0.16 | | | 1.55 | 0.14 | | | > 0.05 |  |
| **TG(14:0_34:1)** | 1.77 | 0.23 | 1.84 | 0.20 | > 0.05 | |  | | 1.95 | | 0.27 | | | 2.15 | 0.27 | | | > 0.05 |  |
| **TG(14:0_34:2)** | 0.83 | 0.09 | 0.86 | 0.07 | > 0.05 | |  | | 1.00 | | 0.09 | | | 0.81 | 0.10 | | | > 0.05 |  |
| **TG(14:0_34:3)** | 0.09 | 0.03 | 0.07 | 0.04 | > 0.05 | |  | | 0.15 | | 0.06 | | | 0.28 | 0.07 | | | > 0.05 |  |
| **TG(14:0_35:1)** | 0.42 | 0.04 | 0.51 | 0.05 | > 0.05 | |  | | 0.57 | | 0.05 | | | 0.59 | 0.06 | | | > 0.05 |  |
| **TG(14:0_35:2)** | 0.34 | 0.04 | 0.33 | 0.03 | > 0.05 | |  | | 0.32 | | 0.04 | | | 0.30 | 0.03 | | | > 0.05 |  |
| **TG(14:0_36:1)** | 1.57 | 0.19 | 1.56 | 0.18 | > 0.05 | |  | | 1.94 | | 0.22 | | | 1.92 | 0.21 | | | > 0.05 |  |
| **TG(14:0_36:2)** | 1.33 | 0.14 | 1.37 | 0.17 | > 0.05 | |  | | 1.26 | | 0.15 | | | 1.49 | 0.16 | | | > 0.05 |  |
| **TG(14:0_36:3)** | 0.66 | 0.08 | 0.67 | 0.08 | > 0.05 | |  | | 0.67 | | 0.10 | | | 1.00 | 0.11 | | | > 0.05 |  |
| **TG(14:0_36:4)** | 0.59 | 0.06 | 0.53 | 0.06 | > 0.05 | |  | | 0.70 | | 0.08 | | | 0.59 | 0.07 | | | > 0.05 |  |
| **TG(14:0_38:4)** | 0.50 | 0.05 | 0.52 | 0.04 | > 0.05 | |  | | 0.57 | | 0.04 | | | 0.58 | 0.06 | | | > 0.05 |  |
| **TG(14:0_38:5)** | 0.59 | 0.21 | 0.51 | 0.24 | > 0.05 | |  | | 1.37 | | 0.36 | | | 1.61 | 0.35 | | | > 0.05 |  |
| **TG(14:0_40:5)** | 1.40 | 0.10 | 1.66 | 0.11 | > 0.05 | |  | | 1.55 | | 0.13 | | | 1.64 | 0.12 | | | > 0.05 |  |
| **TG(16:0_28:1)** | 0.28 | 0.03 | 0.30 | 0.04 | > 0.05 | |  | | 0.43 | | 0.05 | | | 0.40 | 0.04 | | | > 0.05 |  |
| **TG(16:0_28:2)** | 0.20 | 0.03 | 0.25 | 0.03 | > 0.05 | |  | | 0.24 | | 0.03 | | | 0.23 | 0.03 | | | > 0.05 |  |
| **TG(16:0_30:2)** | 0.30 | 0.04 | 0.37 | 0.03 | > 0.05 | |  | | 0.33 | | 0.04 | | | 0.41 | 0.07 | | | > 0.05 |  |
| **TG(16:0_32:0)** | 4.27 | 0.57 | 5.16 | 0.63 | > 0.05 | |  | | 5.69 | | 0.63 | | | 6.38 | 0.61 | | | > 0.05 |  |
| **TG(16:0_32:1)** | 2.42 | 0.24 | 2.55 | 0.27 | > 0.05 | |  | | 2.97 | | 0.34 | | | 3.11 | 0.33 | | | > 0.05 |  |
| **TG(16:0_32:2)** | 0.97 | 0.10 | 0.76 | 0.09 | > 0.05 | |  | | 1.25 | | 0.13 | | | 1.13 | 0.12 | | | > 0.05 |  |
| **TG(16:0_32:3)** | 0.13 | 0.05 | 0.13 | 0.05 | > 0.05 | |  | | 0.20 | | 0.05 | | | 0.09 | 0.04 | | | > 0.05 |  |
| **TG(16:0_33:1)** | 4.22 | 0.51 | 4.29 | 0.59 | > 0.05 | |  | | 5.08 | | 0.62 | | | 4.81 | 0.41 | | | > 0.05 |  |
| **TG(16:0_33:2)** | 1.44 | 0.19 | 1.22 | 0.14 | > 0.05 | |  | | 1.73 | | 0.21 | | | 1.98 | 0.22 | | | > 0.05 |  |
| **TG(16:0_34:0)** | 5.61 | 0.76 | 8.03 | 1.26 | > 0.05 | |  | | 7.13 | | 0.78 | | | 7.06 | 0.67 | | | > 0.05 |  |
| **TG(16:0_34:1)** | 11.82 | 1.57 | 15.47 | 2.08 | > 0.05 | |  | | 12.45 | | 1.78 | | | 13.77 | 1.68 | | | > 0.05 |  |
| **TG(16:0_34:2)** | 6.65 | 0.71 | 6.60 | 0.98 | > 0.05 | |  | | 6.96 | | 0.88 | | | 7.85 | 0.80 | | | > 0.05 |  |
| **TG(16:0_34:3)** | 1.68 | 0.21 | 1.75 | 0.26 | > 0.05 | |  | | 1.58 | | 0.20 | | | 1.60 | 0.22 | | | > 0.05 |  |
| **TG(16:0_34:4)** | 0.09 | 0.04 | 0.21 | 0.06 | > 0.05 | |  | | 0.16 | | 0.05 | | | 0.09 | 0.04 | | | > 0.05 |  |
| **TG(16:0_35:1)** | 3.11 | 0.34 | 3.46 | 0.48 | > 0.05 | |  | | 2.96 | | 0.36 | | | 3.42 | 0.34 | | | > 0.05 |  |
| **TG(16:0_35:2)** | 1.71 | 0.22 | 2.28 | 0.31 | > 0.05 | |  | | 1.96 | | 0.23 | | | 2.19 | 0.24 | | | > 0.05 |  |
| **TG(16:0_35:3)** | 0.68 | 0.08 | 0.61 | 0.06 | > 0.05 | |  | | 0.57 | | 0.06 | | | 0.62 | 0.06 | | | > 0.05 |  |
| **TG(16:0_36:2)** | 16.93 | 2.05 | 20.41 | 2.90 | > 0.05 | |  | | 20.64 | | 2.77 | | | 16.20 | 1.90 | | | > 0.05 |  |
| **TG(16:0_36:3)** | 9.12 | 1.31 | 8.85 | 1.39 | > 0.05 | |  | | 8.14 | | 1.18 | | | 8.05 | 1.01 | | | > 0.05 |  |
| **TG(16:0_36:4)** | 2.64 | 0.39 | 2.97 | 0.52 | > 0.05 | |  | | 3.11 | | 0.50 | | | 3.34 | 0.40 | | | > 0.05 |  |
| **TG(16:0_36:5)** | 0.84 | 0.14 | 0.86 | 0.18 | > 0.05 | |  | | 1.01 | | 0.17 | | | 0.87 | 0.17 | | | > 0.05 |  |
| **TG(16:0_36:6)** | 0.55 | 0.06 | 0.54 | 0.07 | > 0.05 | |  | | 0.54 | | 0.06 | | | 0.60 | 0.07 | | | > 0.05 |  |
| **TG(16:0_37:3)** | 0.33 | 0.04 | 0.43 | 0.05 | > 0.05 | |  | | 0.43 | | 0.06 | | | 0.45 | 0.04 | | | > 0.05 |  |
| **TG(16:0_38:1)** | 0.50 | 0.06 | 0.67 | 0.07 | > 0.05 | |  | | 0.58 | | 0.05 | | | 0.62 | 0.06 | | | > 0.05 |  |
| **TG(16:0_38:2)** | 0.53 | 0.07 | 0.49 | 0.07 | > 0.05 | |  | | 0.58 | | 0.07 | | | 0.66 | 0.07 | | | > 0.05 |  |
| **TG(16:0_38:3)** | 0.25 | 0.09 | 0.22 | 0.09 | > 0.05 | |  | | 0.30 | | 0.09 | | | 0.21 | 0.08 | | | > 0.05 |  |
| **TG(16:0_38:4)** | 0.62 | 0.09 | 0.68 | 0.11 | > 0.05 | |  | | 0.76 | | 0.11 | | | 0.71 | 0.11 | | | > 0.05 |  |
| **TG(16:0_38:5)** | 0.89 | 0.15 | 1.30 | 0.19 | > 0.05 | |  | | 1.28 | | 0.18 | | | 1.08 | 0.16 | | | > 0.05 |  |
| **TG(16:0_38:6)** | 0.54 | 0.10 | 0.69 | 0.12 | > 0.05 | |  | | 0.77 | | 0.11 | | | 0.73 | 0.10 | | | > 0.05 |  |
| **TG(16:0_38:7)** | 0.94 | 0.13 | 1.14 | 0.16 | > 0.05 | |  | | 1.11 | | 0.18 | | | 0.98 | 0.12 | | | > 0.05 |  |
| **TG(16:0_40:6)** | 0.45 | 0.06 | 0.38 | 0.05 | > 0.05 | |  | | 0.51 | | 0.05 | | | 0.57 | 0.09 | | | > 0.05 |  |
| **TG(16:0_40:7)** | 0.60 | 0.08 | 0.59 | 0.06 | > 0.05 | |  | | 0.48 | | 0.06 | | | 0.69 | 0.08 | | | > 0.05 |  |
| **TG(16:0_40:8)** | 0.44 | 0.06 | 0.45 | 0.04 | > 0.05 | |  | | 0.48 | | 0.05 | | | 0.50 | 0.05 | | | > 0.05 |  |
| **TG(16:1_28:0)** | 0.30 | 0.02 | 0.36 | 0.03 | > 0.05 | |  | | 0.31 | | 0.04 | | | 0.24 | 0.04 | | | > 0.05 |  |
| **TG(16:1_30:1)** | 0.27 | 0.03 | 0.27 | 0.03 | > 0.05 | |  | | 0.27 | | 0.04 | | | 0.28 | 0.04 | | | > 0.05 |  |
| **TG(16:1_32:0)** | 1.20 | 0.16 | 1.45 | 0.20 | > 0.05 | |  | | 1.60 | | 0.25 | | | 1.35 | 0.16 | | | > 0.05 |  |
| **TG(16:1_32:1)** | 0.38 | 0.12 | 0.57 | 0.13 | > 0.05 | |  | | 0.59 | | 0.11 | | | 0.60 | 0.13 | | | > 0.05 |  |
| **TG(16:1_32:2)** | 0.35 | 0.06 | 0.46 | 0.08 | > 0.05 | |  | | 0.41 | | 0.08 | | | 0.45 | 0.07 | | | > 0.05 |  |
| **TG(16:1_33:1)** | 0.57 | 0.09 | 0.72 | 0.09 | > 0.05 | |  | | 0.69 | | 0.09 | | | 0.66 | 0.11 | | | > 0.05 |  |
| **TG(16:1_34:0)** | 2.13 | 0.34 | 2.21 | 0.29 | > 0.05 | |  | | 2.58 | | 0.30 | | | 2.44 | 0.34 | | | > 0.05 |  |
| **TG(16:1_34:1)** | 2.80 | 0.34 | 2.41 | 0.30 | > 0.05 | |  | | 3.11 | | 0.41 | | | 2.77 | 0.42 | | | > 0.05 |  |
| **TG(16:1_34:2)** | 1.24 | 0.19 | 1.51 | 0.21 | > 0.05 | |  | | 1.53 | | 0.25 | | | 1.59 | 0.28 | | | > 0.05 |  |
| **TG(16:1_34:3)** | 0.47 | 0.06 | 0.53 | 0.09 | > 0.05 | |  | | 0.50 | | 0.09 | | | 0.53 | 0.09 | | | > 0.05 |  |
| **TG(16:1_36:1)** | 1.88 | 0.29 | 1.68 | 0.21 | > 0.05 | |  | | 2.33 | | 0.33 | | | 1.95 | 0.27 | | | > 0.05 |  |
| **TG(16:1_36:2)** | 1.77 | 0.27 | 1.85 | 0.26 | > 0.05 | |  | | 2.02 | | 0.29 | | | 2.45 | 0.39 | | | > 0.05 |  |
| **TG(16:1_36:3)** | 1.08 | 0.13 | 1.11 | 0.15 | > 0.05 | |  | | 1.33 | | 0.19 | | | 1.16 | 0.16 | | | > 0.05 |  |
| **TG(16:1_36:4)** | 0.22 | 0.08 | 0.39 | 0.15 | > 0.05 | |  | | 0.42 | | 0.14 | | | 0.45 | 0.15 | | | > 0.05 |  |
| **TG(16:1_36:5)** | 0.19 | 0.08 | 0.37 | 0.12 | > 0.05 | |  | | 0.56 | | 0.14 | | | 0.46 | 0.15 | | | > 0.05 |  |
| **TG(16:1_38:3)** | 0.23 | 0.02 | 0.25 | 0.02 | > 0.05 | |  | | 0.18 | | 0.02 | | | 0.23 | 0.02 | | | > 0.05 |  |
| **TG(16:1_38:4)** | 0.44 | 0.05 | 0.48 | 0.05 | > 0.05 | |  | | 0.53 | | 0.06 | | | 0.50 | 0.05 | | | > 0.05 |  |
| **TG(16:1_38:5)** | 0.43 | 0.19 | 0.78 | 0.25 | > 0.05 | |  | | 0.84 | | 0.23 | | | 0.91 | 0.26 | | | > 0.05 |  |
| **TG(17:0_32:1)** | 0.59 | 0.08 | 0.77 | 0.08 | > 0.05 | |  | | 0.69 | | 0.08 | | | 0.78 | 0.09 | | | > 0.05 |  |
| **TG(17:0_34:1)** | 3.01 | 0.41 | 3.11 | 0.34 | > 0.05 | |  | | 3.13 | | 0.31 | | | 3.05 | 0.38 | | | > 0.05 |  |
| **TG(17:0_34:2)** | 3.96 | 0.48 | 3.99 | 0.57 | > 0.05 | |  | | 4.85 | | 0.63 | | | 4.27 | 0.60 | | | > 0.05 |  |
| **TG(17:0_34:3)** | 0.80 | 0.15 | 0.99 | 0.17 | > 0.05 | |  | | 0.82 | | 0.14 | | | 1.07 | 0.15 | | | > 0.05 |  |
| **TG(17:0_36:3)** | 2.38 | 0.29 | 2.46 | 0.32 | > 0.05 | |  | | 2.21 | | 0.30 | | | 2.89 | 0.38 | | | > 0.05 |  |
| **TG(17:0_36:4)** | 0.59 | 0.10 | 0.58 | 0.09 | > 0.05 | |  | | 0.65 | | 0.08 | | | 0.64 | 0.09 | | | > 0.05 |  |
| **TG(17:1_32:1)** | 0.39 | 0.05 | 0.44 | 0.04 | > 0.05 | |  | | 0.40 | | 0.04 | | | 0.37 | 0.04 | | | > 0.05 |  |
| **TG(17:1_34:1)** | 0.93 | 0.11 | 0.98 | 0.12 | > 0.05 | |  | | 0.75 | | 0.09 | | | 0.89 | 0.11 | | | > 0.05 |  |
| **TG(17:1_34:2)** | 0.53 | 0.07 | 0.60 | 0.08 | > 0.05 | |  | | 0.49 | | 0.07 | | | 0.64 | 0.07 | | | > 0.05 |  |
| **TG(17:1_34:3)** | 0.15 | 0.05 | 0.16 | 0.06 | > 0.05 | |  | | 0.18 | | 0.06 | | | 0.19 | 0.05 | | | > 0.05 |  |
| **TG(17:1_36:3)** | 0.90 | 0.10 | 0.97 | 0.10 | > 0.05 | |  | | 0.68 | | 0.09 | | | 0.79 | 0.10 | | | > 0.05 |  |
| **TG(17:1_36:4)** | 0.54 | 0.17 | 0.41 | 0.15 | > 0.05 | |  | | 0.51 | | 0.16 | | | 0.58 | 0.14 | | | > 0.05 |  |
| **TG(17:1_36:5)** | 0.94 | 0.13 | 0.91 | 0.09 | > 0.05 | |  | | 0.98 | | 0.13 | | | 0.94 | 0.14 | | | > 0.05 |  |
| **TG(17:1_38:5)** | 0.07 | 0.01 | 0.07 | 0.01 | > 0.05 | |  | | 0.08 | | 0.01 | | | 0.08 | 0.01 | | | > 0.05 |  |
| **TG(17:1_38:6)** | 0.09 | 0.01 | 0.08 | 0.01 | > 0.05 | |  | | 0.09 | | 0.01 | | | 0.10 | 0.01 | | | > 0.05 |  |
| **TG(17:1_38:7)** | 0.11 | 0.01 | 0.11 | 0.01 | > 0.05 | |  | | 0.09 | | 0.01 | | | 0.11 | 0.01 | | | > 0.05 |  |
| **TG(17:2_34:2)** | 0.27 | 0.02 | 0.30 | 0.03 | > 0.05 | |  | | 0.27 | | 0.03 | | | 0.28 | 0.03 | | | > 0.05 |  |
| **TG(17:2_34:3)** | 0.82 | 0.07 | 0.73 | 0.07 | > 0.05 | |  | | 0.79 | | 0.08 | | | 0.91 | 0.07 | | | > 0.05 |  |
| **TG(17:2_36:2)** | 0.41 | 0.05 | 0.34 | 0.04 | > 0.05 | |  | | 0.30 | | 0.04 | | | 0.35 | 0.05 | | | > 0.05 |  |
| **TG(17:2_36:3)** | 0.83 | 0.10 | 0.71 | 0.08 | > 0.05 | |  | | 0.71 | | 0.10 | | | 0.74 | 0.10 | | | > 0.05 |  |
| **TG(17:2_36:4)** | 0.69 | 0.07 | 0.61 | 0.06 | > 0.05 | |  | | 0.54 | | 0.06 | | | 0.60 | 0.05 | | | > 0.05 |  |
| **TG(17:2_38:5)** | 0.59 | 0.06 | 0.55 | 0.07 | > 0.05 | |  | | 0.46 | | 0.05 | | | 0.60 | 0.06 | | | > 0.05 |  |
| **TG(17:2_38:6)** | 0.32 | 0.03 | 0.33 | 0.04 | > 0.05 | |  | | 0.28 | | 0.03 | | | 0.35 | 0.03 | | | > 0.05 |  |
| **TG(17:2_38:7)** | 0.05 | 0.01 | 0.05 | 0.01 | > 0.05 | |  | | 0.04 | | 0.01 | | | 0.04 | 0.01 | | | > 0.05 |  |
| **TG(18:0_30:0)** | 2.23 | 0.25 | 2.08 | 0.21 | > 0.05 | |  | | 2.43 | | 0.36 | | | 2.03 | 0.23 | | | > 0.05 |  |
| **TG(18:0_30:1)** | 0.69 | 0.10 | 0.64 | 0.08 | > 0.05 | |  | | 0.71 | | 0.10 | | | 0.77 | 0.09 | | | > 0.05 |  |
| **TG(18:0_32:0)** | 7.72 | 0.57 | 8.27 | 0.72 | > 0.05 | |  | | 7.40 | | 0.80 | | | 7.10 | 0.66 | | | > 0.05 |  |
| **TG(18:0_32:1)** | 3.82 | 0.32 | 3.52 | 0.39 | > 0.05 | |  | | 2.98 | | 0.38 | | | 3.91 | 0.42 | | | > 0.05 |  |
| **TG(18:0_32:2)** | 1.24 | 0.13 | 1.22 | 0.15 | > 0.05 | |  | | 1.29 | | 0.18 | | | 1.27 | 0.13 | | | > 0.05 |  |
| **TG(18:0_34:2)** | 11.49 | 1.11 | 10.61 | 1.00 | > 0.05 | |  | | 10.19 | | 1.15 | | | 12.12 | 1.22 | | | > 0.05 |  |
| **TG(18:0_34:3)** | 1.65 | 0.18 | 1.47 | 0.19 | > 0.05 | |  | | 1.67 | | 0.23 | | | 1.75 | 0.17 | | | > 0.05 |  |
| **TG(18:0_36:1)** | 11.92 | 1.64 | 10.89 | 1.56 | > 0.05 | |  | | 10.87 | | 1.46 | | | 12.69 | 1.32 | | | > 0.05 |  |
| **TG(18:0_36:2)** | 21.02 | 2.84 | 16.72 | 2.92 | > 0.05 | |  | | 20.49 | | 2.98 | | | 20.59 | 2.30 | | | > 0.05 |  |
| **TG(18:0_36:3)** | 7.30 | 0.91 | 6.94 | 1.10 | > 0.05 | |  | | 7.49 | | 1.14 | | | 8.89 | 0.95 | | | > 0.05 |  |
| **TG(18:0_36:4)** | 2.69 | 0.28 | 2.56 | 0.38 | > 0.05 | |  | | 2.48 | | 0.33 | | | 2.41 | 0.32 | | | > 0.05 |  |
| **TG(18:0_36:5)** | 1.07 | 0.34 | 0.54 | 0.25 | > 0.05 | |  | | 1.15 | | 0.34 | | | 1.77 | 0.38 | | | > 0.05 |  |
| **TG(18:0_38:6)** | 1.04 | 0.31 | 0.74 | 0.25 | > 0.05 | |  | | 1.19 | | 0.37 | | | 1.70 | 0.42 | | | > 0.05 |  |
| **TG(18:0_38:7)** | 1.01 | 0.33 | 0.83 | 0.28 | > 0.05 | |  | | 1.20 | | 0.33 | | | 1.77 | 0.51 | | | > 0.05 |  |
| **TG(18:1_26:0)** | 0.21 | 0.02 | 0.23 | 0.03 | > 0.05 | |  | | 0.23 | | 0.03 | | | 0.22 | 0.02 | | | > 0.05 |  |
| **TG(18:1_28:1)** | 0.44 | 0.06 | 0.51 | 0.06 | > 0.05 | |  | | 0.51 | | 0.05 | | | 0.56 | 0.06 | | | > 0.05 |  |
| **TG(18:1_30:0)** | 2.71 | 0.43 | 1.99 | 0.30 | > 0.05 | |  | | 2.23 | | 0.32 | | | 3.32 | 0.36 | | | 0.02 |  |
| **TG(18:1_30:1)** | 0.60 | 0.07 | 0.56 | 0.08 | > 0.05 | |  | | 0.70 | | 0.10 | | | 0.74 | 0.10 | | | > 0.05 |  |
| **TG(18:1_30:2)** | 0.38 | 0.05 | 0.40 | 0.05 | > 0.05 | |  | | 0.39 | | 0.05 | | | 0.48 | 0.05 | | | > 0.05 |  |
| **TG(18:1_31:0)** | 2.97 | 0.40 | 2.88 | 0.43 | > 0.05 | |  | | 2.90 | | 0.36 | | | 3.86 | 0.45 | | | > 0.05 |  |
| **TG(18:1_32:0)** | 11.12 | 1.62 | 9.05 | 1.39 | > 0.05 | |  | | 12.42 | | 1.78 | | | 14.24 | 1.66 | | | > 0.05 |  |
| **TG(18:1_32:1)** | 3.57 | 0.48 | 3.08 | 0.53 | > 0.05 | |  | | 4.29 | | 0.55 | | | 5.09 | 0.71 | | | > 0.05 |  |
| **TG(18:1_32:2)** | 1.02 | 0.13 | 0.80 | 0.11 | > 0.05 | |  | | 1.02 | | 0.15 | | | 1.11 | 0.13 | | | > 0.05 |  |
| **TG(18:1_32:3)** | 0.08 | 0.03 | 0.08 | 0.03 | > 0.05 | |  | | 0.11 | | 0.05 | | | 0.13 | 0.05 | | | > 0.05 |  |
| **TG(18:1_33:0)** | 8.18 | 1.30 | 8.19 | 1.23 | > 0.05 | |  | | 8.28 | | 1.13 | | | 10.05 | 1.23 | | | > 0.05 |  |
| **TG(18:1_33:1)** | 6.99 | 1.01 | 6.68 | 1.26 | > 0.05 | |  | | 7.64 | | 1.28 | | | 8.25 | 1.30 | | | > 0.05 |  |
| **TG(18:1_33:2)** | 1.55 | 0.23 | 1.91 | 0.30 | > 0.05 | |  | | 1.68 | | 0.24 | | | 1.83 | 0.24 | | | > 0.05 |  |
| **TG(18:1_33:3)** | 0.20 | 0.06 | 0.10 | 0.05 | > 0.05 | |  | | 0.15 | | 0.06 | | | 0.23 | 0.06 | | | > 0.05 |  |
| **TG(18:1_34:1)** | 29.14 | 4.40 | 23.49 | 4.11 | > 0.05 | |  | | 33.21 | | 5.15 | | | 32.35 | 4.65 | | | > 0.05 |  |
| **TG(18:1_34:2)** | 8.34 | 1.27 | 7.87 | 1.21 | > 0.05 | |  | | 10.84 | | 1.64 | | | 11.95 | 1.62 | | | > 0.05 |  |
| **TG(18:1_34:3)** | 1.68 | 0.23 | 1.55 | 0.23 | > 0.05 | |  | | 1.57 | | 0.24 | | | 2.11 | 0.32 | | | > 0.05 |  |
| **TG(18:1_34:4)** | 0.65 | 0.07 | 0.72 | 0.07 | > 0.05 | |  | | 0.76 | | 0.08 | | | 0.65 | 0.08 | | | > 0.05 |  |
| **TG(18:1_35:2)** | 1.13 | 0.32 | 1.03 | 0.33 | > 0.05 | |  | | 1.49 | | 0.40 | | | 1.97 | 0.40 | | | > 0.05 |  |
| **TG(18:1_35:3)** | 0.54 | 0.08 | 0.50 | 0.06 | > 0.05 | |  | | 0.61 | | 0.08 | | | 0.58 | 0.09 | | | > 0.05 |  |
| **TG(18:1_36:0)** | 8.97 | 1.39 | 6.73 | 0.93 | > 0.05 | |  | | 8.86 | | 1.19 | | | 10.67 | 1.33 | | | > 0.05 |  |
| **TG(18:1_36:1)** | 20.91 | 3.31 | 16.97 | 3.21 | > 0.05 | |  | | 26.05 | | 4.01 | | | 26.65 | 3.69 | | | > 0.05 |  |
| **TG(18:1_36:2)** | 13.36 | 1.93 | 12.91 | 2.33 | > 0.05 | |  | | 15.38 | | 2.45 | | | 18.80 | 2.76 | | | > 0.05 |  |
| **TG(18:1_36:3)** | 5.73 | 1.02 | 6.17 | 1.21 | > 0.05 | |  | | 6.56 | | 1.11 | | | 7.12 | 1.06 | | | > 0.05 |  |
| **TG(18:1_36:4)** | 2.45 | 0.29 | 1.99 | 0.28 | > 0.05 | |  | | 2.92 | | 0.32 | | | 3.39 | 0.35 | | | > 0.05 |  |
| **TG(18:1_36:5)** | 8.19 | 0.97 | 7.14 | 0.82 | > 0.05 | |  | | 6.94 | | 0.59 | | | 9.10 | 0.96 | | | > 0.05 |  |
| **TG(18:1_36:6)** | 0.46 | 0.13 | 0.28 | 0.09 | > 0.05 | |  | | 0.32 | | 0.14 | | | 0.61 | 0.17 | | | > 0.05 |  |
| **TG(18:1_38:5)** | 27.71 | 3.62 | 24.96 | 3.38 | > 0.05 | |  | | 25.89 | | 2.79 | | | 28.44 | 3.38 | | | > 0.05 |  |
| **TG(18:1_38:6)** | 4.63 | 0.75 | 4.76 | 0.61 | > 0.05 | |  | | 4.54 | | 0.47 | | | 6.11 | 0.92 | | | > 0.05 |  |
| **TG(18:1_38:7)** | 0.46 | 0.07 | 0.40 | 0.08 | > 0.05 | |  | | 0.38 | | 0.06 | | | 0.52 | 0.07 | | | > 0.05 |  |
| **TG(18:2_28:0)** | 0.32 | 0.05 | 0.36 | 0.03 | > 0.05 | |  | | 0.38 | | 0.04 | | | 0.48 | 0.05 | | | > 0.05 |  |
| **TG(18:2_30:0)** | 1.03 | 0.13 | 1.00 | 0.10 | > 0.05 | |  | | 1.08 | | 0.13 | | | 1.09 | 0.11 | | | > 0.05 |  |
| **TG(18:2_30:1)** | 0.35 | 0.05 | 0.40 | 0.04 | > 0.05 | |  | | 0.42 | | 0.05 | | | 0.43 | 0.05 | | | > 0.05 |  |
| **TG(18:2_31:0)** | 1.39 | 0.16 | 1.41 | 0.14 | > 0.05 | |  | | 1.67 | | 0.18 | | | 1.99 | 0.25 | | | > 0.05 |  |
| **TG(18:2_32:0)** | 4.02 | 0.50 | 3.92 | 0.34 | > 0.05 | |  | | 4.26 | | 0.40 | | | 4.26 | 0.35 | | | > 0.05 |  |
| **TG(18:2_32:1)** | 1.25 | 0.16 | 1.28 | 0.13 | > 0.05 | |  | | 1.41 | | 0.17 | | | 1.43 | 0.14 | | | > 0.05 |  |
| **TG(18:2_32:2)** | 0.53 | 0.06 | 0.55 | 0.06 | > 0.05 | |  | | 0.57 | | 0.06 | | | 0.61 | 0.07 | | | > 0.05 |  |
| **TG(18:2_33:0)** | 3.06 | 0.41 | 3.57 | 0.41 | > 0.05 | |  | | 3.15 | | 0.32 | | | 3.32 | 0.42 | | | > 0.05 |  |
| **TG(18:2_33:1)** | 1.43 | 0.20 | 1.59 | 0.18 | > 0.05 | |  | | 1.54 | | 0.18 | | | 2.00 | 0.25 | | | > 0.05 |  |
| **TG(18:2_33:2)** | 0.27 | 0.08 | 0.22 | 0.08 | > 0.05 | |  | | 0.18 | | 0.06 | | | 0.16 | 0.08 | | | > 0.05 |  |
| **TG(18:2_34:0)** | 8.04 | 0.96 | 7.54 | 0.74 | > 0.05 | |  | | 7.27 | | 0.71 | | | 8.56 | 0.93 | | | > 0.05 |  |
| **TG(18:2_34:1)** | 6.61 | 0.81 | 7.11 | 0.64 | > 0.05 | |  | | 7.15 | | 0.72 | | | 7.86 | 0.79 | | | > 0.05 |  |
| **TG(18:2_34:2)** | 3.08 | 0.38 | 3.68 | 0.44 | > 0.05 | |  | | 3.36 | | 0.36 | | | 4.82 | 0.63 | | | > 0.05 |  |
| **TG(18:2_34:3)** | 0.22 | 0.08 | 0.22 | 0.06 | > 0.05 | |  | | 0.31 | | 0.11 | | | 0.26 | 0.08 | | | > 0.05 |  |
| **TG(18:2_34:4)** | 0.28 | 0.03 | 0.31 | 0.03 | > 0.05 | |  | | 0.28 | | 0.03 | | | 0.33 | 0.04 | | | > 0.05 |  |
| **TG(18:2_35:1)** | 1.16 | 0.16 | 1.45 | 0.14 | > 0.05 | |  | | 1.25 | | 0.15 | | | 1.59 | 0.19 | | | > 0.05 |  |
| **TG(18:2_35:2)** | 0.29 | 0.11 | 0.38 | 0.12 | > 0.05 | |  | | 0.48 | | 0.15 | | | 0.43 | 0.15 | | | > 0.05 |  |
| **TG(18:2_35:3)** | 0.42 | 0.05 | 0.37 | 0.06 | > 0.05 | |  | | 0.36 | | 0.05 | | | 0.39 | 0.06 | | | > 0.05 |  |
| **TG(18:2_36:0)** | 3.15 | 0.41 | 2.95 | 0.36 | > 0.05 | |  | | 3.45 | | 0.41 | | | 3.64 | 0.34 | | | > 0.05 |  |
| **TG(18:2_36:1)** | 4.81 | 0.55 | 4.95 | 0.43 | > 0.05 | |  | | 5.09 | | 0.39 | | | 5.59 | 0.58 | | | > 0.05 |  |
| **TG(18:2_36:2)** | 4.55 | 0.62 | 5.03 | 0.49 | > 0.05 | |  | | 4.64 | | 0.45 | | | 5.36 | 0.58 | | | > 0.05 |  |
| **TG(18:2_36:3)** | 1.85 | 0.25 | 1.99 | 0.28 | > 0.05 | |  | | 2.24 | | 0.31 | | | 2.63 | 0.34 | | | > 0.05 |  |
| **TG(18:2_36:4)** | 1.00 | 0.18 | 1.30 | 0.20 | > 0.05 | |  | | 0.96 | | 0.12 | | | 1.17 | 0.16 | | | > 0.05 |  |
| **TG(18:2_36:5)** | 1.16 | 0.12 | 1.32 | 0.14 | > 0.05 | |  | | 1.14 | | 0.09 | | | 1.04 | 0.11 | | | > 0.05 |  |
| **TG(18:2_38:4)** | 1.26 | 0.15 | 1.37 | 0.16 | > 0.05 | |  | | 1.35 | | 0.17 | | | 1.40 | 0.19 | | | > 0.05 |  |
| **TG(18:2_38:5)** | 2.16 | 0.48 | 2.84 | 0.52 | > 0.05 | |  | | 2.15 | | 0.48 | | | 1.62 | 0.50 | | | > 0.05 |  |
| **TG(18:2_38:6)** | 0.76 | 0.09 | 0.77 | 0.08 | > 0.05 | |  | | 0.70 | | 0.10 | | | 0.67 | 0.08 | | | > 0.05 |  |
| **TG(18:3_30:0)** | 0.22 | 0.02 | 0.34 | 0.03 | 0.01 | |  | | 0.29 | | 0.03 | | | 0.30 | 0.04 | | | > 0.05 |  |
| **TG(18:3_32:0)** | 0.72 | 0.07 | 0.72 | 0.07 | > 0.05 | |  | | 0.76 | | 0.10 | | | 0.68 | 0.06 | | | > 0.05 |  |
| **TG(18:3_32:1)** | 0.15 | 0.04 | 0.23 | 0.06 | > 0.05 | |  | | 0.15 | | 0.05 | | | 0.10 | 0.04 | | | > 0.05 |  |
| **TG(18:3_33:2)** | 0.01 | 0.00 | 0.02 | 0.00 | > 0.05 | |  | | 0.01 | | 0.00 | | | 0.01 | 0.00 | | | > 0.05 |  |
| **TG(18:3_34:0)** | 1.57 | 0.18 | 1.32 | 0.14 | > 0.05 | |  | | 1.21 | | 0.16 | | | 1.42 | 0.14 | | | > 0.05 |  |
| **TG(18:3_34:1)** | 1.37 | 0.17 | 1.49 | 0.15 | > 0.05 | |  | | 1.47 | | 0.18 | | | 1.16 | 0.13 | | | > 0.05 |  |
| **TG(18:3_34:2)** | 0.66 | 0.09 | 0.72 | 0.09 | > 0.05 | |  | | 0.76 | | 0.08 | | | 0.78 | 0.08 | | | > 0.05 |  |
| **TG(18:3_34:3)** | 0.15 | 0.06 | 0.24 | 0.07 | > 0.05 | |  | | 0.13 | | 0.05 | | | 0.16 | 0.06 | | | > 0.05 |  |
| **TG(18:3_35:2)** | 0.05 | 0.02 | 0.07 | 0.02 | > 0.05 | |  | | 0.04 | | 0.01 | | | 0.04 | 0.01 | | | > 0.05 |  |
| **TG(18:3_36:1)** | 0.47 | 0.14 | 0.44 | 0.13 | > 0.05 | |  | | 0.37 | | 0.12 | | | 0.31 | 0.11 | | | > 0.05 |  |
| **TG(18:3_36:2)** | 0.76 | 0.09 | 1.01 | 0.11 | > 0.05 | |  | | 0.92 | | 0.09 | | | 0.94 | 0.10 | | | > 0.05 |  |
| **TG(18:3_36:3)** | 0.56 | 0.06 | 0.51 | 0.07 | > 0.05 | |  | | 0.49 | | 0.06 | | | 0.64 | 0.07 | | | > 0.05 |  |
| **TG(18:3_36:4)** | 0.75 | 0.08 | 1.18 | 0.17 | > 0.05 | |  | | 0.90 | | 0.11 | | | 0.85 | 0.12 | | | > 0.05 |  |
| **TG(18:3_38:5)** | 3.69 | 0.55 | 7.05 | 1.32 | > 0.05 | |  | | 5.89 | | 1.05 | | | 3.74 | 0.64 | | | > 0.05 |  |
| **TG(18:3_38:6)** | 0.08 | 0.01 | 0.17 | 0.04 | > 0.05 | |  | | 0.11 | | 0.02 | | | 0.11 | 0.03 | | | > 0.05 |  |
| **TG(20:0_32:3)** | 0.11 | 0.01 | 0.11 | 0.01 | > 0.05 | |  | | 0.10 | | 0.01 | | | 0.11 | 0.01 | | | > 0.05 |  |
| **TG(20:0_32:4)** | 0.08 | 0.02 | 0.11 | 0.03 | > 0.05 | |  | | 0.07 | | 0.03 | | | 0.09 | 0.03 | | | > 0.05 |  |
| **TG(20:0_34:1)** | 0.63 | 0.10 | 0.75 | 0.11 | > 0.05 | |  | | 0.54 | | 0.11 | | | 0.78 | 0.10 | | | > 0.05 |  |
| **TG(20:1_24:3)** | 0.13 | 0.02 | 0.16 | 0.03 | > 0.05 | |  | | 0.16 | | 0.03 | | | 0.15 | 0.03 | | | > 0.05 |  |
| **TG(20:1_26:1)** | 0.00 | 0.00 | 0.00 | 0.00 | > 0.05 | |  | | 0.00 | | 0.00 | | | 0.00 | 0.00 | | | > 0.05 |  |
| **TG(20:1_30:1)** | 0.22 | 0.01 | 0.22 | 0.02 | > 0.05 | |  | | 0.22 | | 0.02 | | | 0.22 | 0.01 | | | > 0.05 |  |
| **TG(20:1_32:0)** | 0.17 | 0.02 | 0.17 | 0.01 | > 0.05 | |  | | 0.20 | | 0.02 | | | 0.21 | 0.01 | | | > 0.05 |  |
| **TG(20:1_32:1)** | 0.17 | 0.05 | 0.11 | 0.05 | > 0.05 | |  | | 0.15 | | 0.05 | | | 0.26 | 0.06 | | | > 0.05 |  |
| **TG(20:1_32:2)** | 0.03 | 0.01 | 0.03 | 0.01 | > 0.05 | |  | | 0.03 | | 0.01 | | | 0.04 | 0.01 | | | > 0.05 |  |
| **TG(20:1_32:3)** | 0.03 | 0.01 | 0.02 | 0.00 | > 0.05 | |  | | 0.03 | | 0.00 | | | 0.03 | 0.01 | | | > 0.05 |  |
| **TG(20:1_34:0)** | 0.37 | 0.04 | 0.41 | 0.04 | > 0.05 | |  | | 0.45 | | 0.03 | | | 0.45 | 0.04 | | | > 0.05 |  |
| **TG(20:1_34:1)** | 0.42 | 0.05 | 0.49 | 0.06 | > 0.05 | |  | | 0.53 | | 0.06 | | | 0.63 | 0.06 | | | > 0.05 |  |
| **TG(20:1_34:2)** | 0.45 | 0.04 | 0.49 | 0.04 | > 0.05 | |  | | 0.46 | | 0.03 | | | 0.40 | 0.03 | | | > 0.05 |  |
| **TG(20:1_34:3)** | 0.04 | 0.01 | 0.03 | 0.01 | > 0.05 | |  | | 0.04 | | 0.01 | | | 0.06 | 0.01 | | | > 0.05 |  |
| **TG(20:2_32:0)** | 0.27 | 0.05 | 0.30 | 0.04 | > 0.05 | |  | | 0.28 | | 0.05 | | | 0.33 | 0.05 | | | > 0.05 |  |
| **TG(20:2_32:1)** | 0.04 | 0.01 | 0.02 | 0.01 | > 0.05 | |  | | 0.04 | | 0.01 | | | 0.05 | 0.01 | | | > 0.05 |  |
| **TG(20:2_34:1)** | 0.10 | 0.04 | 0.08 | 0.03 | > 0.05 | |  | | 0.09 | | 0.03 | | | 0.10 | 0.04 | | | > 0.05 |  |
| **TG(20:2_34:2)** | 0.33 | 0.09 | 0.13 | 0.06 | 0.01 | |  | | 0.12 | | 0.04 | | | 0.41 | 0.10 | | | 0.01 |  |
| **TG(20:2_34:3)** | 0.06 | 0.02 | 0.02 | 0.01 | > 0.05 | |  | | 0.03 | | 0.01 | | | 0.06 | 0.01 | | | > 0.05 |  |
| **TG(20:2_34:4)** | 0.05 | 0.01 | 0.01 | 0.01 | 0.01 | |  | | 0.03 | | 0.01 | | | 0.07 | 0.01 | | | > 0.05 |  |
| **TG(20:2_36:5)** | 0.22 | 0.02 | 0.24 | 0.02 | > 0.05 | |  | | 0.24 | | 0.02 | | | 0.20 | 0.02 | | | > 0.05 |  |
| **TG(20:3_32:0)** | 0.43 | 0.11 | 0.37 | 0.08 | > 0.05 | |  | | 0.30 | | 0.08 | | | 0.60 | 0.11 | | | > 0.05 |  |
| **TG(20:3_32:1)** | 0.15 | 0.02 | 0.14 | 0.01 | > 0.05 | |  | | 0.14 | | 0.02 | | | 0.16 | 0.02 | | | > 0.05 |  |
| **TG(20:3_32:2)** | 0.04 | 0.01 | 0.02 | 0.01 | > 0.05 | |  | | 0.03 | | 0.01 | | | 0.05 | 0.01 | | | > 0.05 |  |
| **TG(20:3_34:0)** | 0.60 | 0.08 | 0.63 | 0.06 | > 0.05 | |  | | 0.52 | | 0.06 | | | 0.56 | 0.07 | | | > 0.05 |  |
| **TG(20:3_34:1)** | 0.50 | 0.05 | 0.55 | 0.06 | > 0.05 | |  | | 0.51 | | 0.07 | | | 0.57 | 0.06 | | | > 0.05 |  |
| **TG(20:3_34:2)** | 0.63 | 0.07 | 0.57 | 0.06 | > 0.05 | |  | | 0.56 | | 0.07 | | | 0.63 | 0.10 | | | > 0.05 |  |
| **TG(20:3_34:3)** | 0.05 | 0.02 | 0.02 | 0.01 | > 0.05 | |  | | 0.05 | | 0.02 | | | 0.07 | 0.02 | | | > 0.05 |  |
| **TG(20:3_36:3)** | 0.60 | 0.07 | 0.72 | 0.06 | > 0.05 | |  | | 0.61 | | 0.06 | | | 0.79 | 0.09 | | | > 0.05 |  |
| **TG(20:3_36:4)** | 0.95 | 0.11 | 1.00 | 0.10 | > 0.05 | |  | | 1.06 | | 0.10 | | | 1.13 | 0.11 | | | > 0.05 |  |
| **TG(20:3_36:5)** | 0.05 | 0.01 | 0.04 | 0.01 | > 0.05 | |  | | 0.05 | | 0.01 | | | 0.08 | 0.01 | | | > 0.05 |  |
| **TG(20:4_30:0)** | 0.13 | 0.02 | 0.13 | 0.02 | > 0.05 | |  | | 0.12 | | 0.01 | | | 0.13 | 0.02 | | | > 0.05 |  |
| **TG(20:4_32:0)** | 0.31 | 0.04 | 0.35 | 0.04 | > 0.05 | |  | | 0.35 | | 0.04 | | | 0.35 | 0.03 | | | > 0.05 |  |
| **TG(20:4_32:1)** | 0.09 | 0.03 | 0.04 | 0.02 | > 0.05 | |  | | 0.09 | | 0.03 | | | 0.15 | 0.04 | | | > 0.05 |  |
| **TG(20:4_32:2)** | 0.12 | 0.03 | 0.07 | 0.03 | > 0.05 | |  | | 0.07 | | 0.02 | | | 0.12 | 0.03 | | | > 0.05 |  |
| **TG(20:4_33:2)** | 0.14 | 0.04 | 0.05 | 0.03 | > 0.05 | |  | | 0.07 | | 0.03 | | | 0.15 | 0.03 | | | > 0.05 |  |
| **TG(20:4_34:0)** | 0.13 | 0.05 | 0.07 | 0.03 | > 0.05 | |  | | 0.11 | | 0.04 | | | 0.31 | 0.09 | | | > 0.05 |  |
| **TG(20:4_34:1)** | 0.41 | 0.11 | 0.17 | 0.07 | > 0.05 | |  | | 0.21 | | 0.08 | | | 0.42 | 0.11 | | | > 0.05 |  |
| **TG(20:4_34:2)** | 1.40 | 0.18 | 1.05 | 0.15 | > 0.05 | |  | | 1.05 | | 0.12 | | | 1.50 | 0.17 | | | > 0.05 |  |
| **TG(20:4_34:3)** | 0.09 | 0.03 | 0.06 | 0.03 | > 0.05 | |  | | 0.09 | | 0.04 | | | 0.13 | 0.03 | | | > 0.05 |  |
| **TG(20:4_35:3)** | 0.06 | 0.00 | 0.06 | 0.00 | > 0.05 | |  | | 0.06 | | 0.01 | | | 0.05 | 0.01 | | | > 0.05 |  |
| **TG(20:4_36:2)** | 4.16 | 0.67 | 3.80 | 0.73 | > 0.05 | |  | | 4.53 | | 0.72 | | | 5.02 | 0.67 | | | > 0.05 |  |
| **TG(20:4_36:3)** | 0.87 | 0.12 | 0.91 | 0.12 | 0.03 | |  | | 0.85 | | 0.11 | | | 1.26 | 0.14 | | | 0.03 |  |
| **TG(20:4_36:4)** | 0.60 | 0.08 | 0.49 | 0.07 | > 0.05 | |  | | 0.57 | | 0.08 | | | 0.84 | 0.11 | | | > 0.05 |  |
| **TG(20:4_36:5)** | 0.42 | 0.13 | 0.29 | 0.12 | > 0.05 | |  | | 0.27 | | 0.10 | | | 0.58 | 0.13 | | | > 0.05 |  |
| **TG(20:5_34:0)** | 0.23 | 0.03 | 0.21 | 0.02 | > 0.05 | |  | | 0.21 | | 0.02 | | | 0.24 | 0.03 | | | > 0.05 |  |
| **TG(20:5_34:1)** | 0.39 | 0.06 | 0.45 | 0.06 | > 0.05 | |  | | 0.37 | | 0.05 | | | 0.41 | 0.05 | | | > 0.05 |  |
| **TG(20:5_34:2)** | 0.14 | 0.05 | 0.08 | 0.03 | > 0.05 | |  | | 0.10 | | 0.04 | | | 0.15 | 0.04 | | | > 0.05 |  |
| **TG(20:5_36:2)** | 0.47 | 0.06 | 0.48 | 0.04 | > 0.05 | |  | | 0.50 | | 0.06 | | | 0.52 | 0.06 | | | > 0.05 |  |
| **TG(20:5_36:3)** | 0.31 | 0.04 | 0.27 | 0.05 | 0.01 | |  | | 0.27 | | 0.03 | | | 0.42 | 0.05 | | | 0.01 |  |
| **TG(22:0_32:4)** | 0.06 | 0.01 | 0.04 | 0.01 | > 0.05 | |  | | 0.05 | | 0.01 | | | 0.06 | 0.01 | | | > 0.05 |  |
| **TG(22:1_32:5)** | 0.00 | 0.00 | 0.00 | 0.00 | > 0.05 | |  | | 0.00 | | 0.00 | | | 0.00 | 0.00 | | | > 0.05 |  |
| **TG(22:2_32:4)** | 0.01 | 0.00 | 0.01 | 0.00 | > 0.05 | |  | | 0.01 | | 0.00 | | | 0.02 | 0.00 | | | > 0.05 |  |
| **TG(22:3_30:2)** | 0.01 | 0.00 | 0.01 | 0.00 | > 0.05 | |  | | 0.01 | | 0.00 | | | 0.01 | 0.00 | | | > 0.05 |  |
| **TG(22:4_32:0)** | 0.20 | 0.02 | 0.16 | 0.02 | > 0.05 | |  | | 0.16 | | 0.02 | | | 0.18 | 0.02 | | | > 0.05 |  |
| **TG(22:4_32:2)** | 0.13 | 0.02 | 0.17 | 0.01 | > 0.05 | |  | | 0.17 | | 0.02 | | | 0.15 | 0.02 | | | > 0.05 |  |
| **TG(22:4_34:2)** | 0.40 | 0.05 | 0.38 | 0.04 | > 0.05 | |  | | 0.44 | | 0.05 | | | 0.50 | 0.05 | | | > 0.05 |  |
| **TG(22:5_32:0)** | 0.36 | 0.04 | 0.28 | 0.03 | > 0.05 | |  | | 0.35 | | 0.04 | | | 0.41 | 0.05 | | | > 0.05 |  |
| **TG(22:5_32:1)** | 0.16 | 0.02 | 0.17 | 0.02 | > 0.05 | |  | | 0.16 | | 0.02 | | | 0.19 | 0.02 | | | > 0.05 |  |
| **TG(22:5_34:1)** | 0.28 | 0.09 | 0.12 | 0.07 | > 0.05 | |  | | 0.20 | | 0.07 | | | 0.33 | 0.10 | | | > 0.05 |  |
| **TG(22:5_34:2)** | 0.32 | 0.11 | 0.17 | 0.10 | > 0.05 | |  | | 0.31 | | 0.10 | | | 0.57 | 0.13 | | | > 0.05 |  |
| **TG(22:5_34:3)** | 0.04 | 0.01 | 0.03 | 0.01 | > 0.05 | |  | | 0.03 | | 0.01 | | | 0.04 | 0.01 | | | > 0.05 |  |
| **TG(22:6_32:0)** | 0.08 | 0.02 | 0.12 | 0.04 | > 0.05 | |  | | 0.10 | | 0.03 | | | 0.15 | 0.04 | | | > 0.05 |  |
| **TG(22:6_32:1)** | 0.16 | 0.01 | 0.17 | 0.02 | > 0.05 | |  | | 0.17 | | 0.02 | | | 0.20 | 0.02 | | | > 0.05 |  |
| **TG(22:6_34:1)** | 0.49 | 0.06 | 0.50 | 0.06 | > 0.05 | |  | | 0.58 | | 0.06 | | | 0.58 | 0.06 | | | > 0.05 |  |
| **TG(22:6_34:2)** | 0.75 | 0.22 | 0.73 | 0.22 | > 0.05 | |  | | 0.58 | | 0.20 | | | 0.74 | 0.23 | | | > 0.05 |  |
| **TG(22:6_34:3)** | 0.23 | 0.02 | 0.26 | 0.02 | > 0.05 | |  | | 0.24 | | 0.03 | | | 0.25 | 0.02 | | | > 0.05 |  |

**Supplementary Table S24.**Biochemical abbreviation and Plasma concentration (µM) of Vitamins and Cofactors in low (LRFI) and high (HRFI) Residual Feed Intake bulls on Day 0 and 56 of the feed efficiency trial (Mean ± SEM).

|  |  |  | **Day 0** |  | |  | |  | |  | |  | **Day 56** | |  | | |  | |
| --- | --- | --- | --- | --- | --- | --- | --- | --- | --- | --- | --- | --- | --- | --- | --- | --- | --- | --- | --- |
|  |  | **LRFI** |  | **HRFI** | |  | |  | |  | | **LRFI** |  | | **HRFI** | | |  | |
| **COMPOUND** | **Mean** | **SEM** | **Mean** | **SEM** | | ***P*** | |  | | **Mean** | | **SEM** | **Mean** | | **SEM** | | | ***P*** | |
| **Choline** | 9.62 | 0.42 | 7.96 | 0.38 | 0.003 | |  | | 9.69 | | 0.39 | | | 8.97 | | 0.52 | > 0.05 | |  |

**Supplementary Table S25.**Biochemical abbreviation and Plasma concentration (nM) of Hormones (others) in low (LRFI) and high (HRFI) Residual Feed Intake bulls on Day 0 and 56 of the feed efficiency trial (Mean ± SEM).

|  |  |  | **Day 0** |  | |  | |  | |  | |  | | **Day 56** | |  | |  | |
| --- | --- | --- | --- | --- | --- | --- | --- | --- | --- | --- | --- | --- | --- | --- | --- | --- | --- | --- | --- |
|  |  | **LRFI** |  | **HRFI** | |  | |  | |  | | **LRFI** | |  | | **HRFI** | |  | |
| **COMPOUND** | **Mean** | **SEM** | **Mean** | **SEM** | | ***P*** | |  | | **Mean** | | **SEM** | | **Mean** | | **SEM** | | ***P*** | |
| **COR** | 32.70 | 3.62 | 28.88 | 3.16 | > 0.05 | |  | | 30.85 | | 3.29 | | 30.02 | | 2.97 | | > 0.05 | |  |
| **TEST** | 13.29 | 0.85 | 11.12 | 1.20 | > 0.05 | |  | | 11.71 | | 1.04 | | 11.75 | | 1.07 | | > 0.05 | |  |

**Supplementary Table S26.**Biochemical abbreviation and Plasma ratio (δ) of Isotopes in low (LRFI) and high (HRFI) Residual Feed Intake bulls on Day 0 and 56 of the feed efficiency trial (Mean ± SEM).

|  |  |  | **Day 0** |  | |  | |  | |  | |  | | **Day 56** |  | | |  |  |
| --- | --- | --- | --- | --- | --- | --- | --- | --- | --- | --- | --- | --- | --- | --- | --- | --- | --- | --- | --- |
|  |  | **LRFI** |  | **HRFI** | |  | |  | |  | | **LRFI** | |  | **HRFI** | | |  |  |
| **COMPOUND** | **Mean** | **SEM** | **Mean** | **SEM** | | ***P*** | |  | | **Mean** | | **SEM** | | **Mean** | **SEM** | | | ***P*** |  |
| **¹⁵N** | 6.04 | 0.14 | 6.07 | 0.09 | > 0.05 | |  | | 6.01 | | 0.12 | | 6.05 | | | 0.08 | > 0.05 | | |
| **¹³C** | -17.02 | 0.24 | -17.04 | 0.16 | > 0.05 | |  | | -17.31 | | 0.18 | | -17.38 | | | 0.10 | > 0.05 | | |
